# Comparative Gene Expression Profiling Identifies Common Molecular Signatures of NF-κB Activation in Canine and Human Diffuse Large B Cell Lymphoma (DLBCL)

**DOI:** 10.1371/journal.pone.0072591

**Published:** 2013-09-04

**Authors:** Manikhandan A. V. Mudaliar, Ross D. Haggart, Gino Miele, Grant Sellar, Karen A. L. Tan, John R. Goodlad, Elspeth Milne, David M. Vail, Ilene Kurzman, Daniel Crowther, David J. Argyle

**Affiliations:** 1 Translational Medicine Research Collaboration, Ninewells Hospital, University of Dundee, Dundee, United Kingdom; 2 Pfizer Inc, Translational Medicine Research Collaboration, Ninewells Hospital, Dundee, United Kingdom; 3 Western General Hospital, Department of Pathology, University of Edinburgh, Edinburgh, United Kingdom; 4 Royal (Dick) School of Veterinary Studies and Roslin Institute, University of Edinburgh, Easter Bush, Midlothian, United Kingdom; 5 University of Wisconsin–Madison School of Veterinary Medicine, University of Wisconsin–Madison, Madison, Wisconsin, United States of America; University of Navarra, Center for Applied Medical Research, Spain

## Abstract

We present the first comparison of global transcriptional changes in canine and human diffuse large B-cell lymphoma (DLBCL), with particular reference to the nuclear factor-kappa B (NF-κB) pathway. Microarray data generated from canine DLBCL and normal lymph nodes were used for differential expression, co-expression and pathway analyses, and compared with analysis of microarray data from human healthy and DLBCL lymph nodes. The comparisons at gene level were performed by mapping the probesets in canine microarrays to orthologous genes in humans and *vice versa*. A considerable number of differentially expressed genes between canine lymphoma and healthy lymph node samples were also found differentially expressed between human DLBCL and healthy lymph node samples. Principal component analysis using a literature-derived NF-κB target gene set mapped to orthologous canine array probesets and human array probesets clearly separated the healthy and cancer samples in both datasets. The analysis demonstrated that for both human and canine DLBCL there is activation of the NF-κB/p65 canonical pathway, indicating that canine lymphoma could be used as a model to study NF-κB-targeted therapeutics for human lymphoma. To validate this, tissue arrays were generated for canine and human NHL and immunohistochemistry was employed to assess NF-κB activation status. In addition, human and canine B-cell lymphoma lines were assessed for NF-κB activity and the effects of NF-κB inhibition.

## Introduction

The use of engineered murine cancer models in cancer drug research and development has been criticized as being inadequate for reflecting spontaneous cancers in humans with respect to latency, genomic instability, heterogeneity, cancer recurrence and metastasis^1^. Where murine models have been invaluable in unravelling the molecular mechanisms underlying the cancer phenotype, in drug development, rarely do drugs that have proved successful in mouse models translate to success in human clinical trials. Recently, there has been increasing evidence that spontaneous cancers in dogs could provide model systems to support human cancer drug development [1]. The genetic diversity, phenotypic heterogeneity, anatomical and physiological similarities with humans, large body size, common living environment and sufficient life span supports the dog as a model system for human oncology. Further, the exponential growth in our understanding and ability to study individual genomes has highlighted the rich conservation of gene sequences between man and the domestic species such as the dog. Coupled with the high incidence of cancer in this species [2]–[5], there is an ideal opportunity to utilize this model of disease.

In dogs, the non-Hodgkin Lymphomas, (NHLs) are the most common haematologic malignancies. In people, they represent 5% of all new cancer cases, the fifth leading cause of cancer death, and the second fastest growing cancer in terms of mortality [6]–[8]. In dogs, NHL accounts for approximately 10% of all malignant tumours (83% of all haematopoietic malignancies [9]). NHL is initially highly responsive to standard chemotherapy in both species, with first-remission rates of approximately 90%; however, drug resistance occurs in most cases, resulting in disease recurrence. In recent years it has become clear that aberrant deregulated nuclear factor kappa B (NF-κB) activation is a major feature of lymphoid malignancies in man.

Constitutively active NF-κB pathway is involved in most lymphoid malignancies, particularly in human diffuse large B cell lymphoma (DLBCL) [10], [11]. However, the expression of NF-κB pathway related genes differ between subtypes of DLBCL, establishing their molecular classification [12]–[15]. The germinal-centre B-cell–like (GCB) subgroup of DLBCL originates from the centroblasts and specifically expresses BCL6 whereas the activated B-cell–like (ABC) subgroup originates from plasmablasts and expresses XBP1, PRDM1 and IRF4 among hundreds of other differentially expressed genes [16]. However, studies involving molecular characterisation of canine large B-cell lymphoma are absent in the literature. This may be due to the inherent difficulty of establishing lymphoma cell lines and the restricted availability of standardized large B-cell canine lymphoma cell lines with stable genotypes in addition to the lack of appropriate canine-specific antibody reagents [17].

We present the first comparison of global transcriptional changes taking place in canine and human DLBCL, specifically, investigating the integrity of the NF-κB pathway as a target for therapy. This analysis was supported by immunohistochemical and cell line studies on NFκB Inhibition. The data indicates that a number of common therapeutic targets exist in canine and human DLBCL and highlights the potential of naturally occurring lymphoma in the dog as a model for therapeutic drug development in humans.

## Materials and Methods

### Canine dataset

All biopsy procedures were approved by institutional ethical review panels (University of Wisconsin-Madison, School of Veterinary Medicine IAUAC and the Royal (Dick) School of Veterinary Studies Veterinary Ethical Review Committee (VERC) and included mandatory written consent from patient owners. Lymph node biopsies were taken, as part of normal diagnostic procedures, from dogs newly diagnosed with lymphoma (naïve) and samples from dogs that had relapsed following standard CHOP chemotherapy. Only dogs with confirmed DLBCL after pathological grading were used (histopathological grading by two independent pathologists and CD3/PAX5 marker analysis). Normal lymph node samples were obtained from canines that were euthanized for non-lymphoma conditions. The samples were snap frozen in liquid nitrogen, transported on dry ice and stored at −80°C prior to RNA extraction.

### Microarray data generation

RNA extraction was performed in two balanced random batches. cDNA probes were generated from the RNA, biotin labelled, hybridised onto the Affymetrix GeneChip® Canine Genome 2.0 array and scanned to obtain data. This dataset, with 33 samples that passed QC, was deposited at Gene Expression Omnibus (GEO) database with the accession number GSE30881.

### Human datasets

Public databases were searched for human DLBCL microarray datasets generated from Affymetrix 2.0 technologies (for technological match with the canine array) and GSE12195 (E-GEOD-12195) dataset from ArrayExpress was selected and downloaded for this study [18]. This dataset has raw data from 83 frozen biopsy samples including 73 DLBCL samples and 10 samples of tonsillar B-cells generated using Affymetrix GeneChip® Human Genome U133 Plus 2.0 arrays.

### Data analysis

The quality of the datasets was assessed by box plots, histograms, RNA degradation plots, array-array intensity correlation plots, principal component analysis plots and QC metrics retrieved by ‘yaqcaffy’ [19] and ‘AffyQCReport’ [20] R/bioconductor packages [21], [22] figure SF 11 in File S3 shows the flow chart of the bioinformatics analysis. Two of the 35 samples in the canine dataset and 28 of the 83 samples in the human dataset did not pass the stringent quality control and were removed from further analysis. Data from 33 (23 DLBCL and 10 healthy) samples in the canine dataset (GSE30881) and 55 (45 DLBCL and 10 healthy) samples in the human dataset (GSE12195) were used for analysis. Supplementary tables (ST 1 and ST 2 in File S1) show the included and excluded samples in canine and human datasets.

### NF-κB target gene expression

The datasets were normalised using RMA normalisation method [23], [24] 120 NF-κB target genes (supplementary table ST 3 in file S1) were derived from literature [18] and were mapped to 199 probesets in the canine array and 259 probesets in the human array using web-based NetAffx- Batch Query tool [25]. Hierarchical clustering and principal component analysis of the datasets using the expression levels of mapped NF-κB probesets were performed. The choice of this this NF-κB target gene set was based on the fact that it was derived from many published sources and comparatively more comprehensive than NF-κB gene sets which are available in different databases.

### Differential expression analysis

Analysis of variance (ANOVA) in the RMA normalised datasets between the cancer and healthy samples were performed using Partek® software [26] (version 6.5) and differentially expressed gene lists were created based on the ANOVA log_2_ fold change >±2 at adjusted *p-value* with FDR <0.05. The gene lists were used as inputs in the DAVID Functional Annotation Tool [27] for Gene-Enrichment and Functional Annotation Analysis. In addition, the gene lists were analysed for NF-κB target gene enrichment using the literature derived NF-κB target genes list.

### Co-expression analysis

For co-expression analysis, expression data from cancer samples alone were used, excluding the data from healthy samples. The datasets were normalised using MAS 5.0 normalisation method in Linux-R/bioconductor and probesets with “P” calls in at least one-third of the dataset were included for the analysis [28] Co-expression matrices were constructed using a rigorous cut-off *p-value* of less than 0.0001 with statistical power >80% between the correlated probesets, visualised in Cytoscape [29] and clustered using MCODE [30] plugin (parameters: Haircut  =  True, Fluff  =  False, Node Score Cutoff  = 0.3, K-Core  = 2). The co-expression clusters were explored in the Ingenuity Pathway Analysis [31] with emphasis on canonical pathways and networks.

### Comparison between canine and human datasets

The similarity between the two species were compared at different levels including the similarity between the differentially expressed genes, Gene Ontology enrichment, NF-κB target gene enrichment, global NF-κB target gene expression signatures, co-expression clusters, canonical pathways and networks. For gene level comparison, the probesets from canine array were mapped to orthologous human genes and probesets in the human array and *vice versa* whereas comparison at other levels were direct.

### Tissue samples and tissue array construction

78 cases of human diffuse large B-cell lymphoma (DLBCL) with formalin fixed paraffin wax embedded biopsy tissue were available, with ethical approval (Ethical approval for the use of archival human biopsy material in this study was granted by Lothian Research Ethics Committee), for study, together with formalin fixed paraffin wax embedded biopsies from 17 cases of treatment naïve canine DLBCL, and 5 cases of post-treatment (relapsed) canine DLBCL as described above. Tissue microarrays were constructed using 2 mm diameter cores (between 1 and 5 per case) cut from all 78 cases of human lymphoma and from 20 cases of canine lymphoma (17 treatment naïve, 3 post-treatment). In two cases of post treatment canine DLBCL, the biopsies were too small for use in tissue microarrays, so whole tissue sections were used instead.

### Immunohistochemistry

Immunohistochemistry for p65/p52 was performed using standard laboratory techniques and appropriate controls (no primary antibody and isotype matched). Briefly, sections (4 μm) of tissue microarrays constructed from formalin fixed, paraffin wax embedded lymph nodes were used. Antigen retrieval was carried out in 0.1 M citrate buffer pH 6.0 110°C for 15 minutes followed by blocking of non-specific binding using the Dako REAL system (Dako, Ely, UK) for 10 minutes at 25°C. After overnight incubation with primary antibody at 25°C, specific binding was visualized using the Envision + System-HRP (Dako) according to the manufacturer's instructions, followed by counterstaining with haematoxylin. Primary antibodies used were NF-κB-P100/p52 (Ser865 rabbit polyclonal, Thermo Fisher Scientific at 1/25) and NF-κB/p65 (Rel A, ab-1 rabbit polyclonal Thermo Fisher Scientific at 1/50). Activation of the canonical and alternative NF-κB pathways was measured using a semi-quantitative technique to assess the degree of nuclear staining with antibodies to p65 and p52 respectively. All immunohistochemistry sections were scored by two independent pathologists (JG and EM). In any one tumour, a score of between 0–4 was attributed on the basis of the percentage of cells staining positively with a particular antibody, calculated over all the available cores for that biopsy; 0 =  completely negative, 1 = 1–25% of tumour cell nuclei positive, 2 = 26–50% of nuclei positive, 3 = 51–75% of nuclei positive and 4 = >75% of nuclei positive. The intensity of the nuclear stain tended to be uniform throughout any one case and was scored as negative (0), weakly positive (1), moderately positive (2) or strongly positive (3). A final nuclear histoscore of between 0 and 12 was then calculated by multiplying the percentage score by the intensity score. It was possible to score all cases of human and canine DLBCL for p65, and all cases of canine DLBCL for p52. Only 77 cases of human DLBCL were assessed for p52 due to missing material in the TMA sections.

### Cell lines and culture conditions

All human cell lines were obtained from the ATCC (LGC standards, Middlesex, U.K) and were certified EBV-negative. JM1 is a pre-B lymphoblastic lymphoma line which was maintained and propagated using Iscove's modified Dulbecco's medium with 4 mM L-glutamine adjusted to contain 1.5 g/L sodium bicarbonate (ATCC-LGC standards, Middlesex, U.K.) and supplemented with 0.05 mM 2-mercaptoethanol (Sigma-Aldrich, Dorset, U.K.), 100 U/ml Penicillin, 100 mg/ml Streptomycin (Invitrogen, Paisley, U.K.) and 10% (v/v) foetal bovine serum (Invitrogen, Paisley, U.K.). Jurkat, Clone E6-1, a T cell lymphoma line, RL, a B cell non-Hodgkin's lymphoma cell line with t (14;18) translocation and Pfeiffer, a diffuse large B cell lymphoma (DLBCL) also with the typical t (14;18) (q32; q21) translocation of follicular lymphomas were all maintained and propagated in RPMI-1640 medium (Invitrogen, Paisley, U.K.) and supplemented as above with Penicillin, Streptomycin and foetal bovine serum. Canine 3132 is a suspension B cell lymphoma culture derived from a dog with multi-centric lymphoma. This line most resembles the human equivalent of lines derived from diffuse large B-cell lymphoma (DLBCL). The canine lymphoma line, 3132, was established from ascitic fluid from a dog with malignant lymphoma [17] and propagated in supplemented RPMI-1640 medium as detailed above.

### Chemotherapeutic reagents and NF-κB/IKK inhibitors

Drugs used for the *in vitro* studies include doxorubicin, which was purchased from Pfizer Ltd (Kent, U.K.) and vincristine sulfate from Mayne Pharma Plc (Warwickshire, U.K.). In Solution NF-κB activation inhibitor and IKK inhibitor VII were sourced from Calbiochem (Nottingham, U.K.).

### MTT assays

Cells were seeded in 96-well plates at 5×10^3^ cells per well and incubated for 24 h at 37°C, 5% CO_2_. Drugs and inhibitors were added in triplicates for each concentration before incubation at 37°C for 72 h. CellTiter 96® AQueous One Solution Reagent (Promega, Southampton, U.K.) was added to each well and incubated for 1 hour at 37°C before absorbance at 490 nm was read. Data was analyzed and IC_50_ values calculated using GraphPad prism 5.0 (La Jolla, U.S.A.) and drug combination index was defined using the Chou and Talalay equation [32].

### Antibodies for western blotting immunodetection (WB)

NF-κB/p65 (Rel A, used 1:200 WB) antibody (RB-1638; used 1:200 WB), NF-κB-p100/p52 (Ser865) antibody (RB-10608; used 1:200 WB), NF-κB-p105/p50 (Ser907) antibody (RB-10611; used 1:200 WB) and Cyclin D1/BCL-1 (SP4) antibody (RM-9104; used 1:200 WB) were obtained from Thermo Fisher Scientific (Cheshire, U.K.), while BCL-2 antibody (sc-492; used 1:200 WB) was sourced from Santa Cruz (Santa Cruz, U.S.A.) and I-κBα [E130] antibody (ab32518; used at 1:10000 WB) and β-actin antibody (ab6276; used 1: 10000 WB) from Abcam (Cambridge, U.K.). The swine anti-rabbit HRP conjugated antibody (used at 1:1000) was purchased from DAKO (Ely, Cambridgeshire, U.K.). ECL™ Western Blotting detection reagents from GE Healthcare/Amersham Biosciences (Buckinghamshire, U.K.) were used.

### Electrophoresis, western blotting and immunodetection

40 mg of protein from whole cell lysates were electrophoresed on 10% (v/v) denaturing polyacrylamide gels and transferred onto Hybond ECL™ membranes (GE Healthcare, Buckinghamshire, U.K.) using standard electrophoresis and western blotting procedures. Membranes were blocked in 5% (w/v) skimmed milk (Sigma-Aldrich, Dorset, U.K.) in phosphate-buffered saline (PBS), incubated at 4°C overnight with antibodies at the appropriate concentrations before washing in phosphate-buffered saline with 0.1% (v/v) NP-40 (Sigma-Aldrich, Dorset, U.K.; PBST), incubation with secondary HRP conjugated antibodies. After further washes in PBST, ECL detection agents were used and Hyperfilm ECL™ (GE Healthcare, Buckinghamshire, U.K.) were exposed for appropriate durations to the membranes.

### Nuclear protein extraction and electrophoretic mobility shift assay (EMSA)

Harvested cells were resuspended in 25 mM HEPES, 5 mM KCl, 0.5 mM MgCl_2_ and gently lysed with the addition of equal volume of 25 mM HEPES, 5mM KCl, 0.5 mM MgCl_2_, 1% (v/v) NP-40, incubated for 15 min with rotation at 4°C before centrifugation and removal of cytoplasmic proteins. Nuclear pellets were washed before adding nuclear lysis buffer (25 mM HEPES, 10% (w/v) sucrose, 350 mM NaCl, 0.01% (v/v) NP-40) for 1 hour with rotation at 4°C before centrifugation to obtain the nuclear extracts. All nuclear extraction solutions contained 1x Roche Mini Complete protease inhibitor cocktail solution (Roche applied science, Burgess Hill, U.K.). 22-mer NF-κB consensus oligonucleotide (Promega, Southampton, U.K.) was labelled according to manufacturer's instructions using the DIG gel-shift kit (Roche Applied Science, Burgess Hill, U.K.). 10 mg of nuclear extracts were used per binding reaction as detailed in the manufacturer's protocol, with some control reactions using NF-κB unlabelled oligonucleotide or non-specific negative control OCT1 consensus oligonucleotide (Promega, Southampton, U.K.) for competition or the NF-κB p65 antibody (Thermo Fisher Scientific, Loughborough, U.K.) for supershift reactions. Reactions were run on 6% (v/v) DNA retardation gels (Invitrogen, Paisley, U.K.) and blotted and cross-linked onto positively charged nylon membranes (Roche Applied Science, Burgess Hill, U.K.) before immunodetection.

## Results

### Global expression profiles of the canine (GSE30881) and Human (GSE12195) dataset

**Figure 1:**
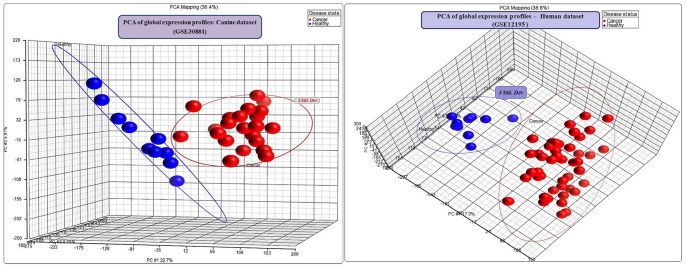
PCA of global gene expression profiles of canine and human datasets. Three dimensional plots of the PCA of canine and human datasets using the first three principal components of global gene expressions. The PCAs show two distinct clusters in both the datasets: the healthy cluster and DLBCL cluster. Blue spheres denote the healthy samples and red spheres denote DLBCL samples. An ellipse was drawn around each of the clusters to mark the limit of the distance of 3 standard deviations from the centre. (A) In the canine dataset (GSE30881), the healthy cluster has all the 10 healthy samples and the cancer cluster has all the 23 canine DLBCL samples. The amounts of variance captured by the first three principal components are 22.7%, 6.97% and 6.75% respectively. (B) In the human dataset (GSE12195), the healthy cluster has all the 10 healthy samples and the cancer cluster has all the 45 human DLBCL samples. The amounts of variance captured by the first three principal components are 17.3%, 14.7% and 6.6% respectively.

To understand the global expression profiles of the canine dataset, principal component analysis (PCA) and hierarchical cluster analysis (HCA) were performed. Using the first three principal components that captured over 36% of the variance in the dataset, the samples were plotted in 3-dimensional space. The PCA plot showed all the 33 samples in the dataset clustered into two distinct clusters, in line with their disease status (Figure 1). Hierarchical clustering of the dataset using Euclidean dissimilarity also showed two distinct top-level clusters separating the DLBCL samples from the healthy samples (data not shown).

Exploratory data analysis (PCA and HCA) was performed to understand global expression profiles of the human dataset. The three dimensional PCA plot, using the first three principal components that captured over 38% of the variance in the dataset showed two distinct clusters: all the 45 DLBCL samples in one cluster and all the 10 healthy samples in another cluster (Figure 1). Predictably, the HCA plot of the human dataset using Euclidean dissimilarity also showed the two sample groups in distinct top level clusters, separating the DLBCL samples from the healthy samples (data not shown).

### Canine and human NF-κB target gene set expression profiles

**Figure 2:**
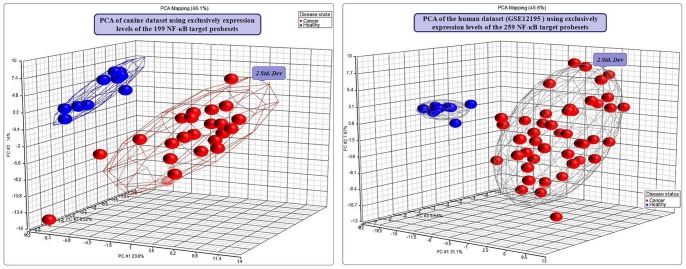
PCA of canine and human datasets using exclusively the expression levels of the NF-κB target genes (probesets). Three dimensional plots of the PCA of canine and human datasets using the first three principal components of the expression levels of the NF-κB target genes (probesets) show clear separation of DLBCL samples from the healthy samples in both the datasets. Blue spheres denote the healthy samples and red spheres denote the DLBCL samples. The ellipsoids drawn around the clusters mark the limit of the 2 standard deviations from the centre in 3-dimensional space. (A) In the canine dataset (GSE30881), the amounts of variance captured by the first three principal components are 23.6%, 14% and 8.52% respectively. (B) In the human dataset (GSE12195), the amounts of variance captured by the first three principal components are 31.6%, 7.87% and 6.54% respectively.

**Figure 3:**
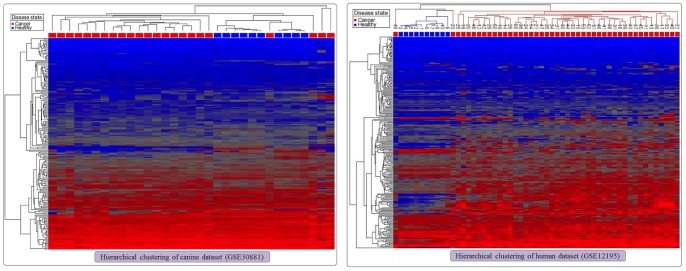
Hierarchical clustering of canine and human datasets using exclusively the expression levels of the NF-κB target genes (probesets). Hierarchical clustering of the canine (A) and human (B) datasets using exclusively the expression levels of the NF-κB target gene set. The samples are arranged in the columns (blue squares denote healthy and red squares denote DLBCL) and the probesets are in the rows. The dendrograms are drawn using Euclidean distances with average linkage method. (A) In the canine dataset (GSE30881), the 199 NF-κB target probesets separate the dataset into three top-level clusters. While the first and the third clusters have exclusively of DLBCL samples, the second cluster has all the healthy samples with two DLBCL samples. (B) In the human dataset (GSE12195), the 259 NF-κB target probesets separate the dataset into two top-level clusters. The first cluster has 12 samples that include all the healthy samples and two DLBCL samples, while the second cluster is solely of 43 DLBCL samples. The numbers above each column refer to sample identification numbers.

We investigated the global behaviour of a set of genes previously identified as the target gene set for the NF-κB pathway in our canine expression data. The healthy canine and DLBCL samples formed two separate clusters in the PCA plot using the expression levels of the NF-κB target 199-probesets (Figure 2). Significantly, the DLBCL and normal samples are separated by the first principal component, which captures 23.6% of the variance. This suggests the disruption of the NF-κB pathway in canine DLBCL. Three DLBCL samples were located with the healthy samples on the basis of the first principal component, but they were removed from the healthy cluster due to their incongruence in second and third principal components. Hierarchical clustering of the canine dataset using the expression levels of the same NF-κB target 199-probesets separated the datasets into three top-level clusters: two DLBCL clusters and one healthy cluster (Figure 3). While the two exclusive DLBCL clusters had 19 and 2 samples, the healthy cluster had all the healthy samples with two remaining DLBCL samples. A PCA plot using the 259 NF-κB target probesets on the human dataset showed clustering of the samples into DLBCL and healthy groups (Figure 2). The separation of the clusters was mainly by the first principal component that captured 31.1% of the variance. Further, HCA plot using the same 259 NF-κB target probesets demonstrated two top level clusters, one entirely made up of DLBCL and the other having all the healthy samples with two DLBCL samples (Figure 3).

### Differential expression patterns in canine and human data set

The difference in the expression patterns of genes between the healthy and DLBCL samples in the canine dataset was analysed using one-way ANOVA. Using an adjusted *p-value* with FDR less than 0.05 and an absolute log_2_ fold change of greater than 2, 3286 probesets were found to be differentially expressed between the healthy and DLBCL samples (data not shown). Of these, 926 probesets were up-regulated and 2360 probesets were down-regulated in canine DLBCL. The important lymphoma specific up-regulated genes include Dihydrofolate reductase (DHFR); CD20 (MS4A1), Myelocytomatosis viral oncogene (MYC), DNA Polymerase-alpha 1 (POLA1), DNA Polymerase-epsilon (POLE), Ribonucleotide reductase M1 (RRM1), DNA Topoisomerase II alpha (TOP2A) and Thymidylate synthase (TYMS). Similarly, one-way ANOVA was performed to understand the difference in the expression patterns of genes between DLBCL samples and healthy samples in the human dataset. Using a FDR adjusted *p-value* of less than 0.05 and absolute log_2_ fold change of greater than 2, 4388 probesets differentially expressed between the healthy and DLBCL samples were identified from the ANOVA results (data not shown). Of these, 2564 probesets were up-regulated and 1824 probesets were down-regulated in DLBCL.

Functional annotation of the up-regulated and down-regulated probesets in canine DLBCL using the DAVID Functional Annotation Tool showed involvement of many cancer-related pathways, as would be expected. The gene ontology and pathways enrichment analysis results for the differentially expressed probesets in canine DLBCL using DAVID are given in the supplementary tables (ST 5–8 in File S2), but the principal results are summarised in Table 1. Functional annotation of the differentially expressed probesets between human healthy and DLBCL using DAVID also showed involvement of cancer-related pathways and gene ontologies. The gene ontology and pathways enrichment analysis results for the up-regulated and down-regulated probesets in DLBCL are given in the supplementary tables (ST 4–8 in File S2) and the top results are given in Table 1.

**Table 1:** Top 20 Gene Ontology (BP) Enrichment in the differentially expressed probesets in canine DLBCL and human DLBCL (ranking based on FDR in the DAVID functional annotation chart).

Canine DLBCL	Human DLBCL
M phase	immune response
cell cycle phase	response to wounding
mitosis	inflammatory response
nuclear division	positive regulation of immune system process
cell cycle	regulation of apoptosis
M phase of mitotic cell cycle	regulation of programmed cell death
cell division	regulation of cell death
organelle fission	regulation of cell proliferation
mitotic cell cycle	defense response
cell cycle process	cell activation
chromosome segregation	regulation of cell migration
regulation of leukocyte activation	regulation of cell motion
regulation of cell activation	blood vessel development
positive regulation of leukocyte activation	vasculature development
positive regulation of cell activation	regulation of cell activation
regulation of lymphocyte activation	taxis
regulation of immune effector process	chemotaxis
positive regulation of lymphocyte activation	leukocyte activation
positive regulation of immune system process	regulation of locomotion
immune response	positive regulation of signal transduction

Ingenuity Pathways Analysis of the up-regulated and down-regulated probesets in canine DLBCL also showed enrichment of cancer related bio functions and canonical pathways (figures SF 3–6 in File S3). The notable canonical pathways enriched in the up-regulated probesets are related to cell cycle regulation that includes mitotic roles of Polo-like kinase pathway (*p-value* 1.3E-08, ratio 14/58), BRCA1 in DNA damage response pathway (*p-value* 1.91E-08, ratio 13/61), cell-cycle checkpoint control pathway (*p-value* 9.65E-08, ratio 10/35), ATM signalling pathway (*p-value* 1.07E-07, ratio 12/53) and p53 signalling pathway. Similarly, the notable bio functions in the up-regulated probesets include cell cycle, genetic disorder, DNA replication, recombination and repair and cellular movement. The main canonical pathways enriched in the down-regulated probesets are related to cellular immune response that includes crosstalk between dendritic cells and natural killer cells (*p-value* 7.39E-06, ratio 16/97), T helper cell differentiation (*p-value* 3.28E-07, ratio 15/70) and IL-12 signalling pathway. The main bio functions enriched in the down-regulated probesets are proliferation of normal cells, cell-to-cell signalling and interaction, immune response, cellular development and apoptosis. Ingenuity Pathways Analysis of the up-regulated and down-regulated probesets in human DLBCL also showed enrichment of DLBCL related bio functions and canonical pathways. Some of the important signalling pathways enriched in the differentially expressed probesets in DLBCL are IL-10, p53, IL-6, NF-κB, IL-2, PI3K, BRCA1 in DNA damage response, NOTCH and PI3K/AKT signalling pathways, which are common to the canine system.

**Figure 4:**
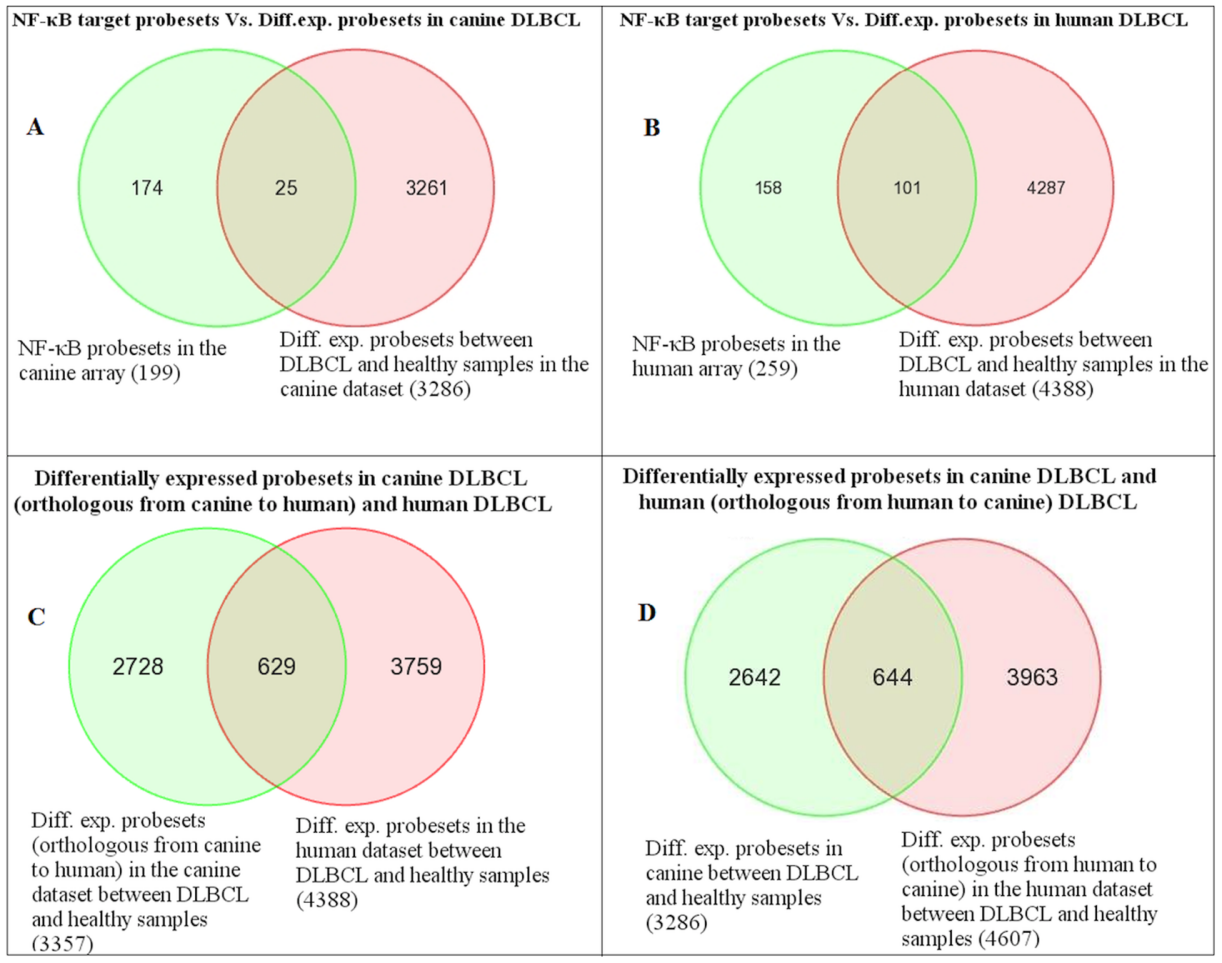
Comparison of differentially expressed probesets in canine and human DLBCLs. Venn diagrams comparing the NF-κB target genes in the differentially expressed probesets (between DLBCL and healthy) of canine DLBCL and human DLBCL and comparison of the number of the differentially expressed probesets of canine DLBCL and human DLBCL. The differentially expressed probesets in the datasets were identified by one-way ANOVA (DLBCL Vs. healthy) of the expression values; selecting probesets with log_2_ fold change over 2 with FDR adjusted *p-value* less than 0.05. (A) 25 NF-κB target probsets (17 NF-κB target genes out of the 120 genes) present in the differentially expressed probesets of canine DLBCL. (B) 101 NF-κB target probsets (54 NF-κB target genes out of the 120 genes) present in the differentially expressed probesets of human DLBCL. (C) Comparison of canine array probesets converted to orthologous human array probesets. (D) Comparison of human array probesets converted to orthologous canine array probesets.

We compared the 3286 differentially expressed canine probesets against the NF-κB target 199-orthologous probesets and found 25 NF-κB target orthologous probesets corresponding to 17 genes were present in the differentially expressed probesets (Table 2). Interestingly, IPA analysis for the NF-κB signalling showed CD40LG, LCK, LTBR and TNFSF11 were present in the down-regulated probesets and EIF2AK2 and MYD88 were present in the up-regulated probesets of canine DLBCL. As we looked for differential expression of NF-κB target genes in the human data, we compared the 4388 differentially expressed probesets against the NF-κB target 259 probesets and found 101 NF-κB target probesets corresponding to 54 genes in the differentially expressed probesets (Table 3 Figure 4). Further, Ingenuity Pathway analysis of the differentially expressed probesets showed up-regulation of IRAK3, LTBR and TNFSF13B and down-regulation of LCK, MAP3K7 and TNFRSF17 in addition to our NF-κB target gene set.

**Table 2:** NF-κB target genes in the differentially expressed gene set of the canine DLBCL.

Sr. No.	Gene Symbol	Gene Title	FDR adjusted *p-value*	Log_2_ fold change
1	BUB1B	budding uninhibited by benzimidazoles 1 homolog beta (yeast)	2.33E-09	5.8804
2	TPMT	thiopurine S-methyltransferase	8.62E-08	2.60776
3	PRKCD	protein kinase C, delta	6.20E-09	2.16004
4	NCF2	neutrophil cytosolic factor 2	6.53E-06	2.15934
5	CD83	CD83 molecule	4.12E-05	−2.09458
6	CXCL13	chemokine (C-X-C motif) ligand 13	0.00991254	−2.25478
7	ICAM1	intercellular adhesion molecule 1	2.89E-06	−2.26415
8	PTPN3	protein tyrosine phosphatase, non-receptor type 3	3.05E-09	−2.39125
9	EGR1	early growth response 1	0.00919512	−2.15779
10	DLA-79	MHC class Ib	0.00362251	−2.05517
11	HSPA1L	heat shock 70kDa protein 1-like	9.32E-07	−2.87401
12	CD36	CD36 molecule (thrombospondin receptor)	0.00858191	−3.62725
13	RGS1	regulator of G-protein signaling 1	5.13E-05	−4.05241
14	IL8	interleukin 8	0.0034537	−4.40177
15	IL12B	interleukin 12B (natural killer cell stimulatory factor 2, cytotoxic lymphocyte maturation factor 2, p40)	4.92E-16	−5.29585
16	IL2	interleukin 2	7.44E-10	−8.65752
17	CD40LG	CD40 ligand	4.89E-12	−7.82082

**Table 3:** NF-κB target genes in the differentially expressed gene set of the human DLBCL.

Sr. No.	Gene Symbol	Gene Title	p-value	Log_2_ fold change
1	CXCL9	chemokine (C-X-C motif) ligand 9	1.21E-19	69.72
2	CXCL10	chemokine (C-X-C motif) ligand 10	3.46E-16	27.93
3	CXCL13	chemokine (C-X-C motif) ligand 13	2.43E-09	12.64
4	SDC4	syndecan 4	3.37E-17	12.39
5	CCL2	chemokine (C-C motif) ligand 2	1.19E-15	12.18
6	STAT1	signal transducer and activator of transcription 1, 91kDa	1.83E-11	10.58
7	IL32	interleukin 32	2.14E-18	9.28
8	SLAMF7	SLAM family member 7	1.02E-09	9.22
9	BCL2	B-cell CLL/lymphoma 2	2.78E-09	9.05
10	CD44	CD44 molecule (Indian blood group)	4.44E-10	7.20
11	SOD2	superoxide dismutase 2, mitochondrial	1.84E-14	7.13
12	ELL2	elongation factor, RNA polymerase II, 2	8.50E-12	6.69
13	CCL3	chemokine (C-C motif) ligand 3	8.87E-10	6.56
14	RGS1	regulator of G-protein signaling 1	2.52E-07	6.28
15	CCL4	chemokine (C-C motif) ligand 4	1.51E-11	6.02
16	CCND2	cyclin D2	4.51E-07	5.41
17	DUSP1	dual specificity phosphatase 1	4.97E-07	5.04
18	IER3	immediate early response 3	1.18E-11	4.79
19	ID2	inhibitor of DNA binding 2	4.39E-12	4.28
20	VIM	vimentin	6.14E-15	4.03
21	IL10	interleukin 10	6.57E-07	3.81
22	IRF4	interferon regulatory factor 4	3.50E-05	3.81
23	LITAF	lipopolysaccharide-induced TNF factor	2.99E-12	3.76
24	PECAM1	platelet/endothelial cell adhesion molecule	4.02E-10	3.68
25	CCR7	chemokine (C-C motif) receptor 7	0.00131648	3.50
26	NCF2	neutrophil cytosolic factor 2	3.92E-05	3.41
27	CD36	CD36 molecule (thrombospondin receptor)	7.49E-06	3.38
28	BATF	basic leucine zipper transcription factor, ATF-like	3.20E-06	3.15
29	IL15RA	interleukin 15 receptor, alpha	4.08E-13	3.04
30	CFLAR	CASP8 and FADD-like apoptosis regulator	5.72E-08	2.84
31	FNDC3A	fibronectin type III domain containing 3A	1.83E-12	2.83
32	AHR	aryl hydrocarbon receptor	1.87E-06	2.61
33	CX3CL1	chemokine (C-X3-C motif) ligand 1	4.28E-06	2.56
34	ICAM1	intercellular adhesion molecule 1	3.95E-08	2.53
35	PTPN1	protein tyrosine phosphatase, non-receptor type 1	1.59E-05	2.44
36	IRF1	interferon regulatory factor 1	3.83E-05	2.44
37	CXCL2	chemokine (C-X-C motif) ligand 2	0.0045885	2.41
38	IL2RA	interleukin 2 receptor, alpha	6.66E-05	2.36
39	TNFAIP3	tumor necrosis factor, alpha-induced protein 3	3.52E-06	2.35
40	JUNB	jun B proto-oncogene	1.84E-07	2.29
41	PIM2	pim-2 oncogene	2.69E-05	2.25
42	IL6	interleukin 6 (interferon, beta 2)	0.00388045	2.11
43	NFKB2	nuclear factor of kappa light polypeptide gene enhancer in B-cells 2	2.00E-09	2.08
44	CD69	CD69 molecule	0.0170441	2.07
45	RFTN1	raftlin, lipid raft linker 1	1.77E-05	-2.02
46	CEP110	centrosomal protein 110kDa	2.10E-07	−2.06
47	MAP3K1	mitogen-activated protein kinase kinase kinase 1	7.79E-05	−2.20
48	WTAP	Wilms tumor 1 associated protein	1.25E-10	−2.25
49	SLC2A5	solute carrier family 2, member 5	0.0017411	−2.32
50	CD40	CD40 molecule, TNF receptor superfamily member 5	4.59E-07	−2.49
51	IL8	interleukin 8	5.73E-05	−2.93
52	RRAS2	related RAS viral (r-ras) oncogene homolog 2	1.31E-08	−4.87
53	BANK1	B-cell scaffold protein with ankyrin repeats 1	0.000318229	−5.07
54	REL	v-rel reticuloendotheliosis viral oncogene homolog (avian)	2.87E-15	−5.43

### Co-expression patterns in canine and human DLBC lymphoma

A gene co-expression network of canine DLBCL was built using the microarray expression data from the 23 canine DLBCL samples and 45 human DLBCL samples. For canine, expression values of 26,154 probesets that were “present” in at least 8 DLBCL samples were used for building the network and using a very strict Pearson correlation coefficient of 0.7999934 (*p-value* 0.0001 with a power of 80%), as a measure of co-expression between the probesets, 16852 nodes (probesets) having 120,270 edges were selected for the co-expression network (figure SF1 in File S3). Many highly interconnected co-expressed clusters were derived from the network and found to be enriched with cell cycle regulation, immune response, TGF-β, Wnt/β-catenin and ERK/MAPK signalling pathways (Figure SF 10 in File S3). Gene co-expression network of human DLBCL was built using the quality control passed, MAS5.0 normalised 45 DLBCL samples in the human dataset. Expression values of 25931 probesets that were “present” in at least 15 samples were used for building the network. Using a very strict Pearson correlation coefficient of 0.6312203 (*p-value* 0.0001 with a power of 80%), as a measure of co-expression between the probesets, 21,435 nodes (probesets) having 421,777 edges were selected for the co-expression network (figure SF 1 in File S3). Many highly interconnected co-expressed clusters were derived from the network (figure SF 2 in File S3) and analysed in DAVID and IPA.

### Comparative analysis of differentially expressed genes in canine DLBCL and human DLBCL

Comparison of the two species was achieved by mapping the canine chip probesets on to orthologous human chip probesets and *vice versa* as described earlier^29^. The comparisons of the number of differentially expressed probesets in both the datasets are presented in Figure 4. 644 orthologous probesets were common between the differentially expressed genes of canine DLBCL and human DLBCL. When we looked at the differentially expressed probesets in both the diseases for NF-κB target genes, the human DLBCL had 54 NF-κB target genes (out of the 120 genes list) and the canine DLBCL had 17 NF-κB target genes (Figure 4, Table 2 and 3). Ingenuity pathway analysis of differentially expressed probesets in canine DLBCL and human DLBCL also showed enrichment of NF-κB signalling pathway in DCBL of both species (figure SF 9 in File S3). Comparison of signalling pathways enriched in the up-regulated and the down-regulated probesets of canine DLBCL and human DLBCL showed enrichment of many lymphoma specific pathways in both canine DLBCL and human DLBCL (figures SF 7 and 8 in File S3).

### Commonly co-regulated pathways in canine DLBCL and human DLBCL

**Figure 5:**
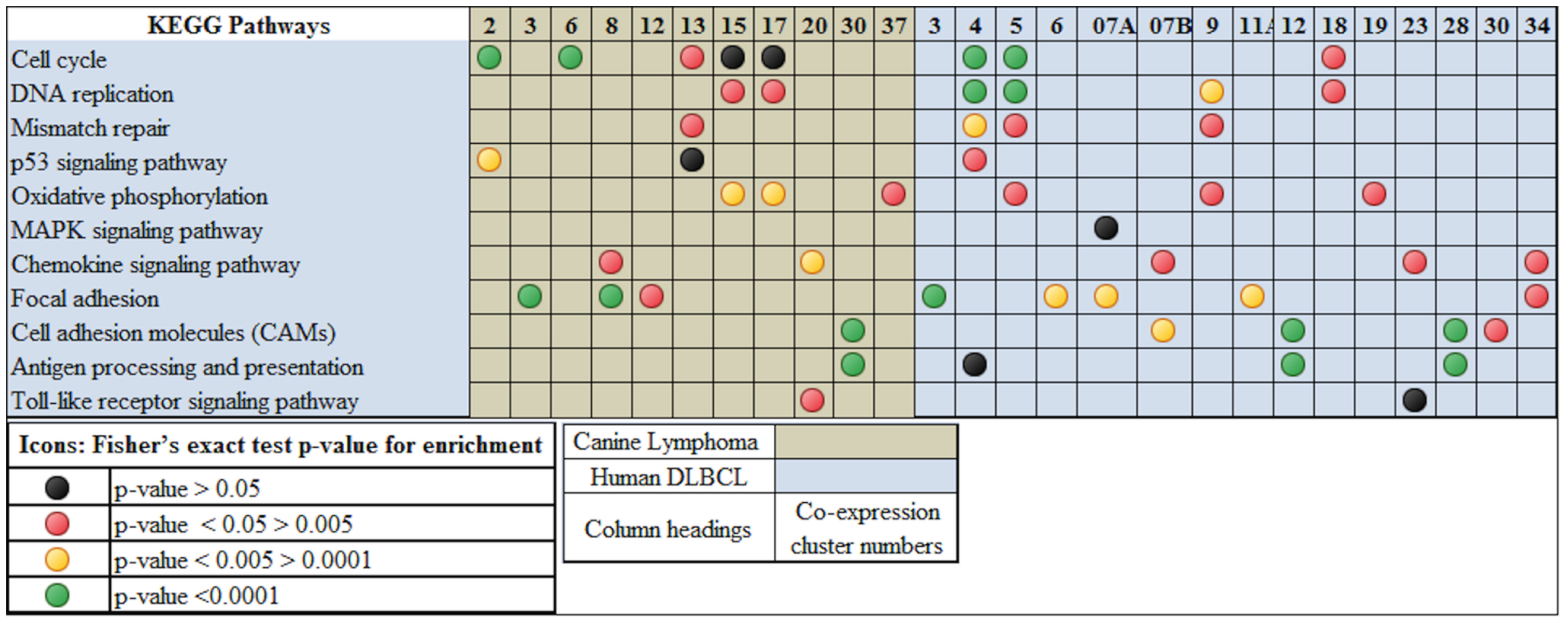
Comparison of enrichment of KEGG pathways in the co-expressed clusters of canine DLBCL and human DLBCL. The highly connected gene clusters identified in the co-expression networks of canine DLBCL and human DLBCL were analysed for enrichment of KEGG pathways using DAVID functional annotation tool. The results from the analysis of each cluster are compiled and the *p-values* of the enrichment score computed by Fisher's exact test are represented graphically as coloured icons.

**Figure 6:**
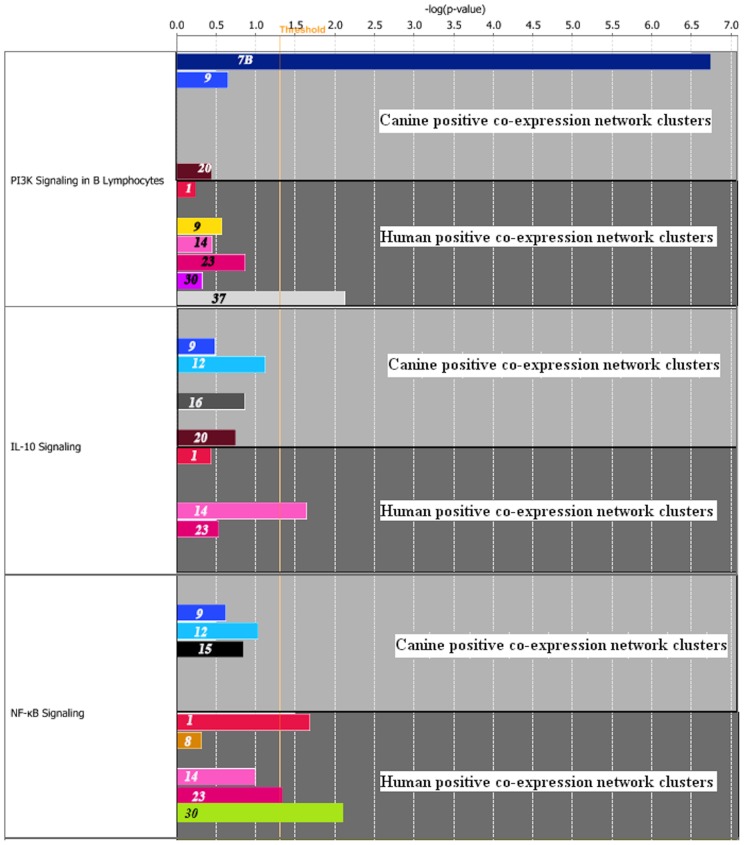
Comparison of enrichment of signalling pathways in the co-expression clusters of canine DLBCL and human DLBCL using IPA. The highly connected gene clusters identified in the co-expression networks of canine DLBCL and human DLBCL were analysed for enrichment of lymphoma related signalling pathways using IPA tool. The result of the comparison analysis is represented graphically. The numbers on the bars denote the co-expression cluster numbers while the length of the bars show their significance, negative log of the *p-value* for the pathway computed by Fisher's exact test, in the relevant pathway.

The co-expression clusters obtained from the positive co-expression networks were analysed for enrichment of signalling pathways using DAVID and comparison work flow of the IPA (Figures 5 and 6). We could find many signalling pathways that are important in the pathogenesis of lymphoma enriched in the co-expressed clusters of DCBL in both species. As the genes present in the densely connected co-expressed clusters are generally co-regulated and expressed in similar fashion, we can safely assume that the enriched pathways in these clusters are active and related to each other. The notable signalling pathways that are enriched in co-expressed clusters of DCBL in both species: PI3K signalling in B Lymphocytes, NF-κB, p53, PI3K, JAK/Stat and PI3K/AKT signalling pathways as well as upregulation of IL-10, IL-6, and IL-2.

### Analysis of NF-κB pathways in canine and human tissue sections demonstrates activation of both canonical and alternative pathways

**Figure 7:**
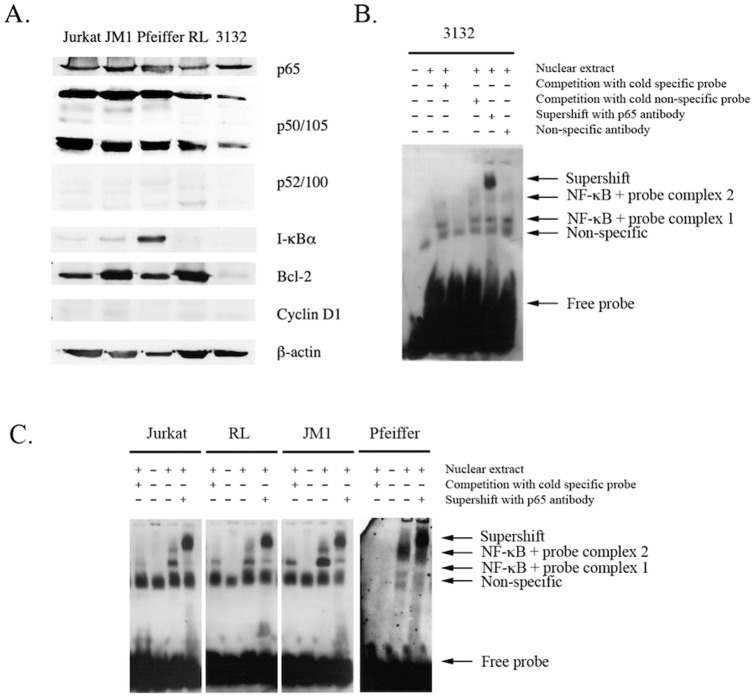
In vivo and in vitro Validation of NF-κB Status in Canine and Human DLBCL. NF-κB expression and activation in human T and B cell lymphoma cell lines and canine B cell lymphoma cell line. Whole cell lysates from Jurkat, JM1, Pfeiffer, RL and 3132 cells were eletrophoresed and immunoblotted for the detection of expression of signaling components of the NF-κB pathway (A). Nuclear protein from human T (Jurkat) and B (RL, JM1, Pfeiffer) cell lymphoma lines as well as canine B (3132) cell lymphoma line, were extracted. EMSAs were performed on these samples using non-radioactive DIG-labeled NF-κB consensus oligonucleotide probes in binding reactions. Specificity was tested by competition using unlabeled (cold) specific and non-specific probes and “supershift” using specific antibody for NF-κB p65/RelA and a non-specific antibody (p53). Samples were then subjected to electrophoresis in DNA retardation gels, before transfer onto nylon membranes and chemiluminescence detection of the DIG-labels. EMSAs were performed using nuclear extracts of canine (B) and human (C) lymphoma cell lines. Bands are indicated by arrows and annotated. ‘+’ and ‘−‘ refer to components being added or omitted respectively, to standard gel shift binding reactions.

To support the array date, we performed immunohistochemical analysis of human and canine lymphoma samples (Figure 7). All cases of human DLBCL showed at least low levels of nuclear staining for p65; range of histoscores  = 1–12, mean  = 6.2, median  = 6.25. In just over 40% of cases (33/78), there was more intense nuclear staining in at least 50% of nuclei (histoscore ≥6), suggesting increased activation of the classic, canonical NF-κB pathway. Similar patterns of staining were seen in treatment naïve and post-treatment canine DLBCL. However, although no completely negative cases were seen, in most only weak nuclear positivity was identified: histoscore range for treatment naïve cases  = 1.5–3, mean  = 2.18, and median  = 2.0; histoscore range for post-treatment cases  = 2–4, mean  = 3.0, median  = 3.0. Staining for nuclear p52 in human DLBCL was generally at lower levels than for p65. In 9/77 cases staining was entirely negative, and moderate or strong nuclear staining in >50% of tumour cell nuclei (histoscore ≥6) was only seen in 8/77; histoscore range  = 0–8.5, mean  = 2.24, median  = 1.50. Conversely, nuclear staining for p52 was generally more intense than for p65 in canine DLBCL. All cases showed at least weak staining in most nuclei, and moderate or strong nuclear staining in >50% of cells was identified in 15/17 treatment naïve DLBCL and 4/5 post-treatment canine DLBCL; for treatment naïve cases histoscore range  = 6–10, mean  = 7.68, median  = 8.0; post-treatment histoscore range  = 4–12, mean  = 7.2, median  = 6.0.

**Figure 8:**
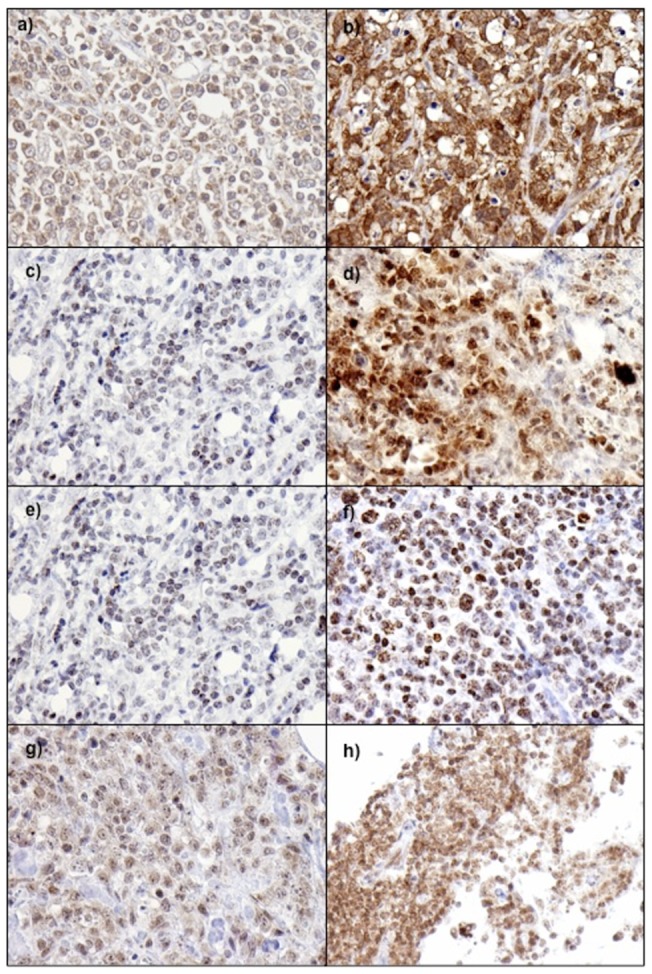
Illustrative photomicrographs showing different nuclear staining intensities for p65 and p52. p65 staining in human DLBCL; weak (a), strong (b). p65 staining in canine DLBCL weak (c), strong (d). p52 staining in human DLBCL; weak (e), strong (f). p52 staining in canine DLBCL; weak (g), strong (h).

Using western blot analysis we demonstrate that all human lymphoma lines and canine 3132 line express p65 and p50/105 subunits but little or none of the p52/100 subunit, indicating the classical canonical pathway is activated in the lines tested (Figure 8A). None of these lines are known to harbor the t(11;14) translocation and consequently do not upregulate expression of cyclin D1 as demonstrated in Fig. 5A. Pfeiffer cells express high levels of I-κBα but also have high constitutive levels of p65 and p50. Nuclear extracts were shown to contain translocated NF-κB p65 complexes that bound to κB motifs in the NF-κB oligonucleotides in nuclear extracts of the canine 3132 B cell lymphoma line (lane 2, Fig. 8B). Specificity of bands (complexed 1 and 2) indicated was shown by the reduction of signal by competition with cold unlabeled NF-κB consensus oligonucleotide bands (lane 3, Fig. 8B) but not with non-specific cold Oct2A probe (lane 4, Fig. 8B). “Supershift” of bands occurred in the presence of the NF-κB p65 antibody (lane 5, Fig. 8B) but not the p53 antibody (lane 6, Fig. 8B). Comparable NF-κB translocation and binding to κB motifs were also demonstrated in nuclear extracts of human Jurkat, RL, JM1 and Pfeiffer lines (lanes 3, 7, 11, 15, Fig. 8C). Specificity of bands (complexes 1 and 2) indicated were shown by the reduction or absence of signal by competition with cold unlabelled NF-κB consensus oligonucleotide bands (lanes 1, 5, 9, 13, Fig. 8C) and “supershift” of bands in the presence of NF-κB p65 antibody (lanes 4, 8, 12, 16 Fig. 8C). 2 NF-κB-probe complexes were observed, with complex 2 being the primary band. The non-specific band probably originated from the commercial preparation of the NF-κB consensus oligonucleotides as the “labelled probe only” lanes (lanes 2, 6, 10, 14, Fig. 8C) demonstrate the presence of this band but not in any of the lanes with the kit controls that utilized labelled Oct2A probe and Oct2A factor (not shown).

### NF-κB inhibition combined with classical chemotherapy treatment on cell viability and NF-κB activation

**Figure 9:**
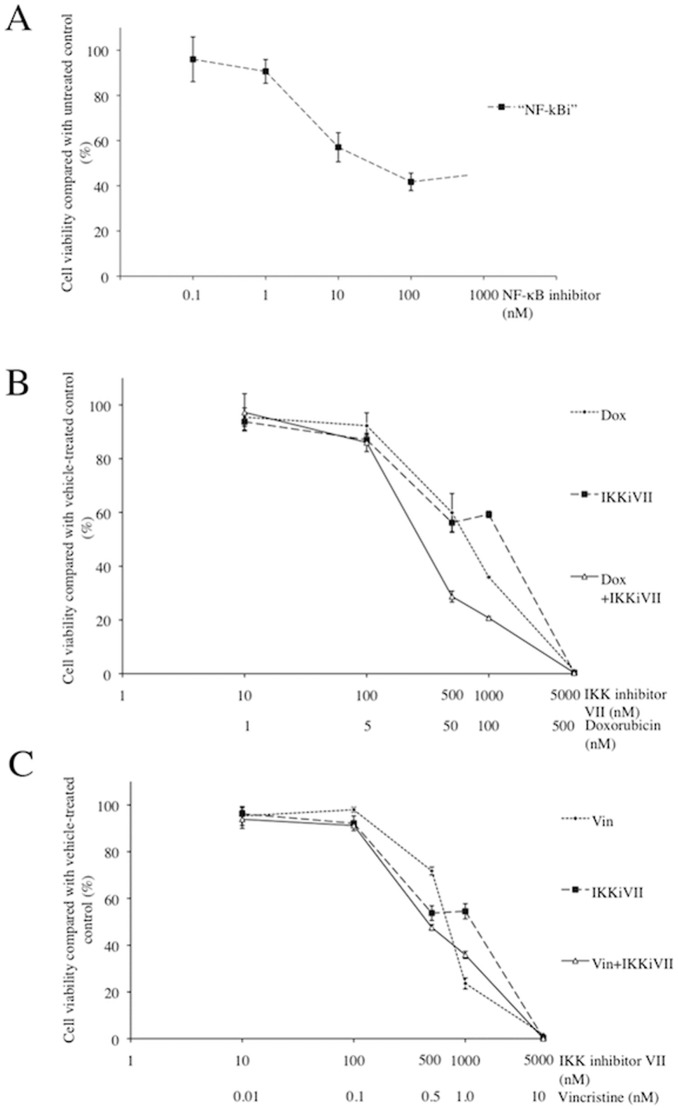
Sensitivity of 3132 cells to NF-κB inhibitor, IKK inhibitor VII, doxorubicin and vincristine. Cell viability of 3132 cells were measured post-treatment with NF-κB inhibitor (A), doxorubicin (B) or vincristine (C) in the presence/absence of IKK inhibitor VII or with IKK inhibitor VII only, using Promega CellTiter 96® AQ_ueous_ One Solution Cell Proliferation MTT assays. Data from doxorubicin and vincristine single treatments are depicted in B and C respectively as small closed diamond points on short dashed lines while IKK inhibitor VII (B and C) and NF-κB inhibitor (A) single treatments are represented as large closed square points on long dashed lines. Combination treatments of doxorubicin (B) or vincristine (C) with IKK inhibitor VII are presented as open triangular points on solid lines.

The array data and immunohistochemistry support the hypothesis that the NK-κB pathway is a potential target in lymphoma in both species. To support this conclusion, we performed a number of drug and cytotoxicity assays on human and canine lymphoma cell lines targeting this pathway. Utilizing an MTT assay, dose response curves and IC_50_ values were generated for doxorubcin and vincristine for all cell lines (data not shown). The IC_50_ value of the NF-κB inhibitor on 3132 cells was calculated to be 10nM. This NF-κB inhibitor is not thought to be cytotoxic even at a high dose of 10mM. The drug only killed 50% of 3132 cells at 10nM and further increases in the doses of NF-κB inhibitor up to 1mM did not decrease viability of 3132 cells (Figure 9A).

The IKK inhibitor VII exhibited a cytotoxic effect on 3132 cells with an IC_50_ of about 0.7 mM-1mM and enhanced the cytotoxic effects of doxorubicin (Fig. 9B). However, IKK inhibitor VII antagonized the cytotoxic effects of vincristine (Fig. 9C). Using the Chou and Talalay formula to calculate the combination index [32] from IC_50_ values obtained using GraphPad Prism 5, IKK inhibitor VII was found to be moderately to slightly synergistic with doxorubicin (CI  = 0.87) but antagonistic to the action of vincristine (CI  = 1.99) in 3132 cells.

**Figure 10:**
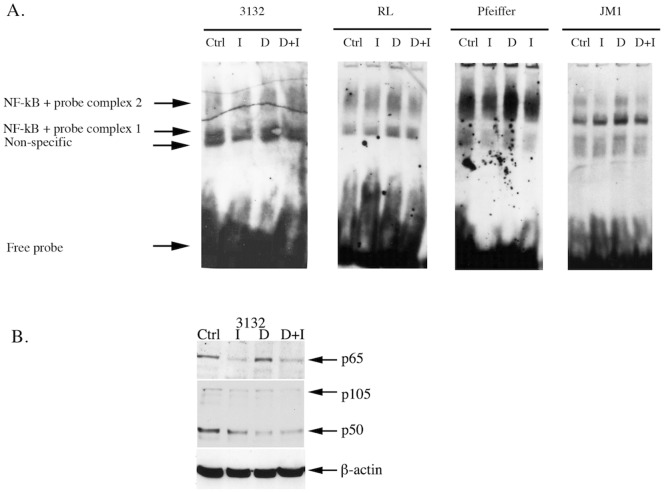
The effect of doxorubicin and IKK inhibitor VII on the activation of NF-κB in human B cell lymphoma cell lines and canine B cell lymphoma cell lines. Nuclear protein from human B (RL, JM1, Pfeiffer) cell lymphoma lines as well as canine B (3132) cell lymphoma line treated with doxorubicin (A) and/or IKK inhibitor VII (B), were extracted. EMSAs were performed on these samples using non-radioactive DIG-labelled NF-κB consensus oligonucleotide probes in binding reactions. Samples were then subjected to electrophoresis in DNA retardation gels, before transfer onto nylon membranes and chemiluminescence detection of the DIG-labels. Bands are indicated by arrows and annotated. ‘+’ and ‘−’ refer to components being added or omitted respectively, to standard gel shift binding reactions. Expression of classical pathway NF-κB subunits in 3132 nuclear extracts when 3132 cells are treated with doxorubicin and/or IKK inhibitor VII at IC_50_ doses was detected by western blotting of whole cell lysates (B).

When IKK inhibitor VII was added to cultures of 3132, Pfeiffer, JM1 and RL cell lines, constitutive nuclear NF-κB binding activity was reduced compared with controls (Fig. 10). Combined treatments with IKK inhibitor VII and doxorubicin attenuated and downregulated NF-κB binding activity compared with treatment with doxorubicin alone, the latter of which up-regulates constitutive NF-κB binding activity. The EMSA profile demonstrated by RL cells was similar to that of 3132 cells. There was greater activation of NF-κB in Pfeiffer cells (especially with respect to complex 2) in comparison with the other lines. JM1 pre-B lymphoma cells also demonstrated nuclear NF-κB activity, which is up-regulated in the presence of traditional chemotherapeutics and attenuated by co-treatment with IKK inhibitor VII. In addition to nuclear translocation and activation, there was downregulation of overall expression of p65 upon IKK inhibitor VII treatment of 3132 cells and attenuation of the increased p65 expression upon doxorubicin treatment (Fig. 10A).

## Discussion

While mortality and incidence for most cancers are decreasing, the age-standardised incidence for NHL increased by more than a third (35%) in the twenty-year period between 1988–2007 in the U.K. and canine and human NHL is the most common haematological malignancy in dogs and humans respectively [6]–[10]. In this study, we sought to establish whether canine lymphoma is a valid model for human non-Hodgkin's lymphoma (NHL), specifically focusing on the NF-κB pathway. Dysregulation of the NF-κB pathway has been consistently observed in human lymphoid malignancies [33]–[36] the main mechanisms of dysregulation involving chromosomal mutations (e.g. p100) resulting in pathway activation; BCL10, MALT1 and CARMA1 dependent activation of IKK [37]; and viral protein stimulation of the IKK pathway [33], [38]. In activated B-cell-like diffuse large B-cell lymphoma, CARD11-dependent chronic B-cell receptor signalling is the mechanism by which NF-κB is constitutively activated, thus preventing apoptosis [39] Finally, up-regulation of the anti-apoptotic Bcl-2 family proteins (e.g. through activation of the NF-κB pathway) has been shown to be a cause of chemotherapy resistance [40].

In this study we show that array data in human and canine lymphoma samples demonstrates activation of NF-κB pathways, and is further supported by immunohistochemical data and drug studies on cell lines. We performed expression analysis on canine samples and then compared the data to human DLBC lymphoma expression data available through standard databases. For all canine patients RNA of sufficient quantity and quality was retrieved for analysis. Principal component analysis of global expression for canine DLBCL demonstrated separation of samples into two distinct clusters: a healthy cluster (10 samples) and lymphoma cluster (23 samples). However, at this global level PCA did not separate samples that were either naïve or were at relapse (figure SF 12 in File S3).

For the canine samples, we compared the 3286 differentially expressed probesets against the NF-κB target 199-orthologous probesets and found 25 NF-κB target orthologous probesets corresponding to 17 genes were present in the differentially expressed probesets. In addition to the core NF-κB gene sets, IPA analysis for NF-κB signalling showed CD40LG, LCK, LTBR and TNFSF11 were present in the down-regulated probesets and EIF2AK2 and MYD88 were present in the up-regulated probesets of canine DLBCL. These results provide evidence that the classical/canonical pathway is activated in canine DLBCL rather than the alternative pathway. Specifically, for down-regulated genes: LTBR is a receptor for LTbeta in the NF-κB alternative pathway in humans and CD40LG (or CD40L) can be a ligand for NF-κB alternative pathway, both indicating canonical pathway activation in lymphoma rather than alternative pathway activation for NF-κB [41]. Interestingly, loss of LCK expression has been linked to resistance to apoptosis in B cell tumours and down-regulation of TNFS11 fits with the diagnosis of NHL in these dogs [42]. For human DLBCL, it is interesting to note that LCK is also down-regulated as for canine DLBCL. However, LTBR is up-regulated, which may reflect a different initiating event that may cause activation of the alternative pathway.

For up-regulated canine genes, EIF2AK2 encodes for double stranded RNA-activated protein kinase, which phosphorylates IKK. IKK phosphorylates I-κB for degradation, allowing NF-κB translocation to the nucleus [43]. MYD88 is involved in channelling activation of the p65 NF-κB pathway via TLR stimulation and is considered a major player in certain forms of human DLBC lymphoma [44]. This may reflect that canine lymphoma could result from chronic B-cell inflammatory stimulation or even viral infection. Further analysis of the human data suggests an important role for NOTCH signalling. Hes1, a canonical Notch target and transcriptional repressor, is responsible for sustaining IKK activation in T-ALL. In addition, Notch-1 can increase NF-κB activity through a variety of mechanisms. There is evidence in some B cell malignancies (e.g. B-CLL) that Notch signalling plays a critical role in cell survival and apoptosis resistance and suggests that it could be a novel potential therapeutic target.

Both *in vivo* IHC data and *in vitro* cell line data indicate that the NF-κB pathway is constitutively activated in human and canine NHL. Conventional lymphoma chemotherapeutic drugs such as doxorubicin and vincristine can inadvertently exacerbate the disease and contribute to chemoresistance by upregulating NF-κB activation. The custom tissue array data for both human and canine lines suggest that both canonical and alternate pathways are activated based upon both p65 and p52 staining. This is in contrast to the cell line data where the canonical pathway predominates. This is possibly explained by the heterogeneity of the tissue arrays compared to cell lines. The immunohistochemistry did suggest subtle differences in expression patterns between human and canine samples that again could indicate the heterogeneity in tissue samples and the lack of molecular subtype classification system in the canine samples. However, biologically the canine and human lines have activation of this pathway and respond similar to drug treatments. All human and canine cell lines tested in this study indicate that high levels of the p65 subunit and the p50/105 subunit are expressed whilst the p52/100 subunit is virtually undetectable, suggesting that the classical canonical NF-κB pathway is predominantly activated in these non-Hodgkin's lymphoma lines rather than the alternative pathway. In contrast to the NF-κB inhibitor, IKK inhibitor VII was able to reduce 3132, RL, JM1 and Pfeiffer cell viabilities (Supplementary data in File S4) down to levels of 5% and below, demonstrating that the IKK complex/subunits may have NF-κB-independent consequences on cell survival, specificities of inhibitors notwithstanding. Further, IKK inhibitor VII is able to potentiate cell killing in conjunction with doxorubicin and vincristine in human and canine B-cell lymphoma by inhibiting NF-κB activation as well as IKK-dependent NF-κB-independent pathways. In addition, it is able to act synergistically with doxorubicin, a chemotherapeutic drug that activates the IKK complex through protein kinase C. From these studies, IKK inhibitors show promise as therapeutic agents for targeting aberrant NF-κB activation in canine and human NHL, possibly reducing acquired chemoresistance thus leading to enhanced patient survival. The results in this study also support observations made by Gaurnier-Hausser *et al* in which canonical NF-kappa B activity was evaluated by electrophoretic mobility shift assays and immunoblot analyses, and NF-kappa B target gene expression was measured by quantitative real time PCR [45]. In that study constitutive canonical NF-kappa B activity and increased NF-kappa B target gene expression were detected in primary DLBCL tissue, as with the current study. Using a NEMO-binding domain peptide the authors demonstrated that dogs with relapsed DLBCL inhibited NF-kappa B target gene expression and reduced tumor burden. This work underscored the dog as a translational model for human DLBCL.

While this study focused specifically on NF-κB activity between human and canine lymphoma, we also explored differentially expressed genes in canine lymphoma and non-lymphoma. The difference in the expression patterns of genes between the healthy and DLBCL samples in the canine dataset highlighted important lymphoma-specific up-regulated genes including DHFR, MS4A1, MYC, POLA1, POLE, RRM1, TOP2A and TYMS. These gene sets are of particular importance in lymphoma for a number of reasons, including their role in drug resistance or as novel therapeutic targets. As an example, amplification of the DHFR has been linked to resistance of lymphoid malignancies to antimetabolite chemotherapy drugs such as methotrexate [46]. In addition, Pralatrexate, a potent DHFR inhibitor is currently showing promise in Phase II clinical trials for human non-Hodgkin lymphoma [47]. Identification of DHFR during molecular diagnosis, may help to guide drug selection processes. As a further example with relation to drug resistance, The RRM1gene encodes the regulatory subunit of ribonucleotide reductase, an essential enzyme that catalyzes the reduction of ribonucleoside diphosphates to the corresponding deoxyribonucleotides. RRM1 is involved in carcinogenesis, tumor progression, and the response of non–small-cell lung cancer to treatment. It is the molecular target of gemcitabine (2′,2′-difluorodeoxycytidine), an antimetabolite with activity in several malignancies including lymphoma. Overexpression of RRM1 in canine lymphoma may suggest resistance to these classes of drugs. Interestingly, TOP2A expression (and up regulation in this current study) has been linked to sensitivity of tumours to anthracyclines such as doxorubicin. The amplification of TOP2A in breast cancer predicts increased sensitivity to anthracylines in women [48]. The results from the canine lymphoma samples suggest why anthracylines are considered to be the most important component of treatment regimes in dogs. As well as indicators of drug resistance, expression of some genes may also reflect markers of poor prognosis. In this study we identified upregulation of cMyc. Myc is considered to be a key oncogene transcription factor involved in cell cycle control and cell proliferation. In humans, approximately 5% to 10% of diffuse large B-cell lymphomas harbor a MYC oncogene rearrangement and is considered to be a negative prognostic marker [49].

In terms of potential therapeutic targets, MS4A1 (also referred to as CD20) has been demonstrated as a strong therapeutic target in human DLBCL. Rituximab is a chimeric monocloncal antibody targeted to CD20 that has demonstrated efficacy in the treatment of human DLBCL in combination with the CHOP protocol [50]. Rituximab does not efficiently bind canine CD20 or provide any therapeutic benefit in the dog. However, this finding adds support for groups currently trying to make monoclonal antibodies against canine CD20 for both diagnostic and therapeutic purposes. In this study we also identified upregulation of TYMS, which is also involved in responsiveness to chemotherapeutic drugs. TYMS catalyzes the methylation of deoxyuridylate to deoxythymidylate using 5,10-methylenetetrahydrofolate (methylene-THF) as a cofactor. This function maintains the dTMP (thymidine-5-prime monophosphate) pool critical for DNA replication and repair. The enzyme has been of interest as a target for cancer chemotherapeutic agents. It is considered to be the primary site of action for 5-fluorouracil, 5-fluoro-2-prime-deoxyuridine, and some folate analogs.

For canine and human samples, the co-expression clusters obtained from the positive co-expression networks were analysed for enrichment of signalling pathways using DAVID and comparison work flow of the IPA. The notable signalling pathways that are enriched in co-expressed clusters of both canine and human diseases are: NF-κB, p53, JAK/Stat and PI3K/AKT signalling pathways. In addition, up-regulation of IL-10, IL-6, and IL-2 supports a role for inflammatory pathways maintaining the malignant phenotype and suggest that there may be common therapeutic targets in both species. Interestingly the PI3/AKT pathway also has a major influence on NF-κB signalling, playing a critical role in NF-κB-dependent survival in DLBCL. Taken together, the conclusions from the global expression profiles for canine and human DLBCL are:

- In canine DLBCL there are a number of up-regulated genes that have therapeutic and prognostic importance and may influence drug choices during therapy.
- There is activation of the classical/canonical NF-κB pathway in canine lymphoma.
- Human and canine DLBCL share pathways, which have potential therapeutic implications including NF-κB, PI3/AKT, Notch and JAK/STAT.
- Canine lymphoma may represent a natural model of human disease and may offer a system to rapidly advance novel compounds to the clinic.

## Conclusion

It has been suggested that the dog may provide a more valid model for human cancer drug development as both species share comparable responsiveness to chemotherapy and development of drug resistance, in addition to comparable molecular and histological features [1]–[5]. The results from this study support these observations and complement the recent study by Frantz et al [51]. The prevalence of naturally occurring canine lymphoma is sufficient for clinical trials with multi-modality protocols being feasible in animals of this size. Information gained on drug activity, toxicity, dose regimen, biomarker development and use in combination therapies in dogs, can be employed in the development of novel therapies in human cancer management, the translation of biological concepts in cancer to *in vivo* models and the generation of new information about cancer.

## Supporting Information

File S1 ST 1: Description of the canine dataset (GSE30881). ST 2: Description of the human dataset (GSE12195). ST 3: NF-κB target genes adopted from Compagno *et al* [18]. ST 4: Differentially expressed probesets in canine DLBCL.
(DOC)

File S2 ST 5: Gene Ontology (BP) enrichment in the up-regulated probesets in canine DLBCL (DAVID functional annotation chart, ranked on FDR). ST 6: Gene Ontology (BP) enrichment in the down-regulated probesets in canine DLBCL (DAVID functional annotation chart, ranked on FDR). ST 7: Kegg pathway hits in the up-regulated probesets in canine DLBCL (DAVID functional annotation chart, ranked on FDR). ST 8: Kegg pathway hits in the down-regulated probesets in canine DLBCL (DAVID functional annotation chart, ranked on FDR).
(DOC)

File S3 SF 1: Positive co-expression networks of canine and human DLBCLs. SF 2: Clusters identified from the co-expression network of human DLBCL. SF 3: Ingenuity Pathway Analysis results showing the top canonical pathways enriched in the up-regulated probesets of canine DLBCL. SF 4: Ingenuity Pathway Analysis results showing the top canonical pathways enriched in the down-regulated probesets of canine DLBCL. SF 5: Ingenuity Pathway Analysis results showing the top bio functions enriched in the up-regulated probesets of canine DLBCL. SF 6: Ingenuity Pathway Analysis results showing the top bio functions enriched in the down-regulated probesets of canine DLBCL. SF 7: Comparison of enrichment of KEGG pathways in the differentially expressed probesets of canine DLBCL and human DLBCL. SF 8: Comparison of enrichment of signalling pathways in the differentially expressed probesets of canine DLBCL and human DLBCL in IPA. SF 9: Comparison of enrichment of NF-κB signalling pathway in differentially expressed genes in canine lymphoma and human DLBCL using IPA. SF 10: Clusters identified from the co-expression network of canine DLBCL. SF 11: Flow chart of the bioinformatics analysis. SF 12: Hierarchical clustering and principal components analysis of the canine dataset GSE30881 showing the clustering of normal, naïve and relapsed samples.
(DOC)

File S4 Supplemental data 4: GSEA Report for Dataset Canine_GSE30881_RMA_cg_2.
(PDF)
