# Supplementary material for: Comparative Gene Expression Profiling Identifies Common Molecular Signatures of NF-κB Activation in Canine and Human Diffuse Large B Cell Lymphoma (DLBCL)
Source: PLoS One. 2013 Sep 4;8(9):e72591. doi: 10.1371/journal.pone.0072591 (PMC3762807; doi:10.1371/journal.pone.0072591)
Supplement: File S1 — ST 1: Description of the canine dataset (GSE30881). ST 2: Description of the human dataset (GSE12195). ST 3: NF-κB target genes adopted from Compagno et al [18]. ST 4: Differentially expressed probesets in canine DLBCL. (DOC) [file pone.0072591.s001.doc]

**Supplemental Data 1**

**ST 1: Description of the canine dataset (GSE30881)**

| **Sr. No.** | **Array Data File** | **Case ID** | **Diagnosis** | **Naïve or relapsed** | **Days since last chemotherapy** | **Age** (yrs) | **Sex** | **RIN** | **Included or excluded in the analysis** |
| --- | --- | --- | --- | --- | --- | --- | --- | --- | --- |
| 1 | GSM766024.CEL | 124200 | DLBCL | Naïve | 0 | 2.7 | F/S | 9.6 | Included |
| 2 | GSM766025.CEL | 127003 | DLBCL | Naïve | 0 | 4.7 | M/N | 9.6 | Included |
| 3 | GSM766026.CEL | 123709 | DLBCL | Relapsed | 82 | 11.8 | F/S | 9.3 | Included |
| 4 | GSM766027.CEL | 116012 | DLBCL | Relapsed | 672 | 8 | M/N | 9.8 | Included |
| 5 | GSM766028.CEL | 127718 | DLBCL | Naïve | 0 | 12.6 | F/S | 9.8 | Included |
| 6 | GSM766029.CEL | 93419 | DLBCL | Naïve | 0 | 7.3 | M | 9.8 | Included |
| 7 | Not Applicable | 113925 | DLBCL | Relapsed | 600 | 10.7 | M/N | 9.1 | Excluded |
| 8 | GSM766030.CEL | 129126 | DLBCL | Naïve | 0 | 7.2 | F/S | 10 | Included |
| 9 | GSM766031.CEL | 121504 | DLBCL | Relapsed | 32 | 10.6 | M/N | 9.9 | Included |
| 10 | GSM766032.CEL | 128335 | DLBCL | Naïve | 0 | 8.1 | F/S | 9.8 | Included |
| 11 | GSM766033.CEL | 123939 | DLBCL | Naïve | 0 | 5.7 | M/N | 10 | Included |
| 12 | GSM766034.CEL | 123939 | DLBCL | Relapsed | 152 | 6.4 | M/N | 9.9 | Included |
| 13 | GSM766035.CEL | 123541 | DLBCL | Relapsed | 123 | 8.7 | M/N | 9.3 | Included |
| 14 | GSM766036.CEL | 128943 | DLBCL | Naïve | 0 | 9.1 | M | 9.5 | Included |
| 15 | GSM766037.CEL | 124246 | DLBCL | Naïve | 0 | 5.9 | M/N | 10 | Included |
| 16 | GSM766038.CEL | 126747 | DLBCL | Naïve | 0 | 12 | M/N | 9.8 | Included |
| 17 | Not Applicable | 121449 | DLBCL | Relapsed | 14 | 8.7 | F | 7.5 | Excluded |
| 18 | GSM766039.CEL | 123454 | DLBCL | Relapsed | 76 | 10.8 | M/N | 9.1 | Included |
| 19 | GSM766040.CEL | 127555 | DLBCL | Naïve | 0 | 5.4 | F/S | 9.8 | Included |
| 20 | GSM766041.CEL | 124359 | DLBCL | Naïve | 0 | 10.1 | M/N | 9.7 | Included |
| 21 | GSM766042.CEL | 125073 | DLBCL | Naïve | 0 | 8.2 | F/S | 9.7 | Included |
| 22 | GSM766043.CEL | 127887 | DLBCL | Naïve | 0 | 6.2 | M/N | 8.9 | Included |
| 23 | GSM766044.CEL | 127793 | DLBCL | Naïve | 0 | 8.8 | F/S | 9.3 | Included |
| 24 | GSM766045.CEL | 121197 | DLBCL | Relapsed | 13 | 8 | M/N | 9.6 | Included |
| 25 | GSM766046.CEL | 125898 | DLBCL | Naïve | 0 | 13.3 | F/S | 9.8 | Included |
| 26 | GSM766047.CEL | Dog1 | Normal |  | 0 | 0 |  | 9.4 | Included |
| 27 | GSM766048.CEL | Dog1 | Normal |  | 0 | 0 |  | 9.2 | Included |
| 28 | GSM766049.CEL | Dog1 | Normal |  | 0 | 0 |  | 9.2 | Included |
| 29 | GSM766050.CEL | Dog1 | Normal |  | 0 | 0 |  | 8.8 | Included |
| 30 | GSM766051.CEL | U71 | Normal |  | 0 | 0 |  | 7.7 | Included |
| 31 | GSM766052.CEL | U72 | Normal |  | 0 | 0 |  | 6 | Included |
| 32 | GSM766053.CEL | U78 | Normal |  | 0 | 0 |  | 7.2 | Included |
| 33 | GSM766054.CEL | U74 | Normal |  | 0 | 0 |  | 6.8 | Included |
| 34 | GSM766055.CEL | U73 | Normal |  | 0 | 0 |  | 5.8 | Included |
| 35 | GSM766056.CEL | U75 | Normal |  | 0 | 0 |  | 7.1 | Included |

Table showing the relationship of microarray data files in the canine dataset (GSE30881) with patient clinical information, extracted RNA quality (RNA integrity number (RIN)) and whether the files were included or excluded in the analysis after quality control. Sr. Nos. 7 and 17 were excluded from the analysis for quality reasons and thus not deposited in GEO.

**ST 2: Description of the human dataset (GSE12195)**

| **Sr. No.** | **Array Data File** | **Diagnosis** | **Included or excluded in the analysis** | **Available DLBCL subtype information** | **Predicted subtype (Wright *et al^19^.* genes)** | **Predicted subtype (Blenk *et al.^36^* genes)** |
| --- | --- | --- | --- | --- | --- | --- |
| 1 | GSM306875.CEL | Normal | Included | Normal | - | - |
| 2 | GSM306876.CEL | Healthy | Included | Normal | - | - |
| 3 | GSM306877.CEL | Healthy | Included | Normal | - | - |
| 4 | GSM306878.CEL | Healthy | Included | Normal | - | - |
| 5 | GSM306879.CEL | Healthy | Included | Normal | - | - |
| 6 | GSM306880.CEL | Healthy | Included | Normal | - | - |
| 7 | GSM306881.CEL | Healthy | Included | Normal | - | - |
| 8 | GSM306882.CEL | Healthy | Included | Normal | - | - |
| 9 | GSM306883.CEL | Healthy | Included | Normal | - | - |
| 10 | GSM306884.CEL | Healthy | Included | Normal | - | - |
| 11 | GSM306886.CEL | DLBCL | Included | Not Available | ABC | ABC |
| 12 | GSM306887.CEL | DLBCL | Included | Not Classified | ABC | ABC |
| 13 | GSM306888.CEL | DLBCL | Included | Not Available | ABC | ABC |
| 14 | GSM306889.CEL | DLBCL | Included | Not Available | ABC | ABC |
| 15 | GSM306890.CEL | DLBCL | Included | Not Available | ABC | ABC |
| 16 | GSM306892.CEL | DLBCL | Included | Not Available | ABC | GCB |
| 17 | GSM306893.CEL | DLBCL | Included | ABC | ABC | ABC |
| 18 | GSM306894.CEL | DLBCL | Included | ABC | ABC | ABC |
| 19 | GSM306895.CEL | DLBCL | Included | ABC | ABC | ABC |
| 20 | GSM306898.CEL | DLBCL | Included | GCB | GCB | GCB |
| 21 | GSM306899.CEL | DLBCL | Included | ABC | ABC | ABC |
| 22 | GSM306900.CEL | DLBCL | Included | ABC | ABC | ABC |
| 23 | GSM306902.CEL | DLBCL | Included | GCB | GCB | GCB |
| 24 | GSM306903.CEL | DLBCL | Included | Not Available | GCB | GCB |
| 25 | GSM306904.CEL | DLBCL | Included | ABC | ABC | ABC |
| 26 | GSM306905.CEL | DLBCL | Included | ABC | ABC | ABC |
| 27 | GSM306906.CEL | DLBCL | Included | Not Available | ABC | ABC |
| 28 | GSM306908.CEL | DLBCL | Included | Not Available | ABC | ABC |
| 29 | GSM306909.CEL | DLBCL | Included | Not Available | GCB | GCB |
| 30 | GSM306910.CEL | DLBCL | Included | ABC | ABC | ABC |
| 31 | GSM306911.CEL | DLBCL | Included | Not Available | GCB | GCB |
| 32 | GSM306912.CEL | DLBCL | Included | Not Available | ABC | ABC |
| 33 | GSM306914.CEL | DLBCL | Included | Not Available | ABC | ABC |
| 34 | GSM306915.CEL | DLBCL | Included | ABC | ABC | ABC |
| 35 | GSM306919.CEL | DLBCL | Included | GCB | GCB | GCB |
| 36 | GSM306920.CEL | DLBCL | Included | Not Available | GCB | ABC |
| 37 | GSM306921.CEL | DLBCL | Included | GCB | GCB | GCB |
| 38 | GSM306922.CEL | DLBCL | Included | Not Available | GCB | GCB |
| 39 | GSM306923.CEL | DLBCL | Included | GCB | GCB | GCB |
| 40 | GSM306924.CEL | DLBCL | Included | ABC | ABC | ABC |
| 41 | GSM306925.CEL | DLBCL | Included | ABC | ABC | ABC |
| 42 | GSM306926.CEL | DLBCL | Included | Not Available | ABC | ABC |
| 43 | GSM306927.CEL | DLBCL | Included | Not Available | ABC | ABC |
| 44 | GSM306928.CEL | DLBCL | Included | Not Available | ABC | ABC |
| 45 | GSM306930.CEL | DLBCL | Included | Not Available | GCB | GCB |
| 46 | GSM306931.CEL | DLBCL | Included | Not Available | GCB | GCB |
| 47 | GSM306932.CEL | DLBCL | Included | Not Available | ABC | GCB |
| 48 | GSM306933.CEL | DLBCL | Included | GCB | GCB | GCB |
| 49 | GSM306934.CEL | DLBCL | Included | Not Available | GCB | GCB |
| 50 | GSM306935.CEL | DLBCL | Included | Not Available | GCB | GCB |
| 51 | GSM306936.CEL | DLBCL | Included | ABC | ABC | ABC |
| 52 | GSM306937.CEL | DLBCL | Included | Not Available | GCB | GCB |
| 53 | GSM306938.CEL | DLBCL | Included | ABC | ABC | ABC |
| 54 | GSM306939.CEL | DLBCL | Included | Not Classified | GCB | GCB |
| 55 | GSM306940.CEL | DLBCL | Included | Not Available | GCB | GCB |
| 56 | GSM306891.CEL | DLBCL | Excluded | - | - | - |
| 57 | GSM306896.CEL | DLBCL | Excluded | - | - | - |
| 58 | GSM306897.CEL | DLBCL | Excluded | - | - | - |
| 59 | GSM306907.CEL | DLBCL | Excluded | - | - | - |
| 60 | GSM306913.CEL | DLBCL | Excluded | - | - | - |
| 61 | GSM306916.CEL | DLBCL | Excluded | - | - | - |
| 62 | GSM306918.CEL | DLBCL | Excluded | - | - | - |
| 63 | GSM306944.CEL | DLBCL | Excluded | - | - | - |
| 64 | GSM306946.CEL | DLBCL | Excluded | - | - | - |
| 65 | GSM306947.CEL | DLBCL | Excluded | - | - | - |
| 66 | GSM306948.CEL | DLBCL | Excluded | - | - | - |
| 67 | GSM306949.CEL | DLBCL | Excluded | - | - | - |
| 68 | GSM387565.CEL | DLBCL | Excluded | - | - | - |
| 69 | GSM387566.CEL | DLBCL | Excluded | - | - | - |
| 70 | GSM387567.CEL | DLBCL | Excluded | - | - | - |
| 71 | GSM387568.CEL | DLBCL | Excluded | - | - | - |
| 72 | GSM387569.CEL | DLBCL | Excluded | - | - | - |
| 73 | GSM387570.CEL | DLBCL | Excluded | - | - | - |
| 74 | GSM387571.CEL | DLBCL | Excluded | - | - | - |
| 75 | GSM387572.CEL | DLBCL | Excluded | - | - | - |
| 76 | GSM387573.CEL | DLBCL | Excluded | - | - | - |
| 77 | GSM387574.CEL | DLBCL | Excluded | - | - | - |
| 78 | GSM387575.CEL | DLBCL | Excluded | - | - | - |
| 79 | GSM387576.CEL | DLBCL | Excluded | - | - | - |
| 80 | GSM387577.CEL | DLBCL | Excluded | - | - | - |
| 81 | GSM387578.CEL | DLBCL | Excluded | - | - | - |
| 82 | GSM387579.CEL | DLBCL | Excluded | - | - | - |
| 83 | GSM387580.CEL | DLBCL | Excluded | - | - | - |

Table showing the microarray data files in the downloaded human dataset (GSE12195) with the available DLBCL subtype information, whether the files were included or excluded in our analysis after quality control and predicted DLBCL subtypes using statistical models based on Wright *et al.^19^* and Blenk *et al.^36^* classifier genes.

**ST 3: NF-κB target genes adopted from Compagno *et al* [18]**

| **Sr. No.** | **Gene Symbol** | **Sr. No.** | **Gene Symbol** | **Sr. No.** | **Gene Symbol** | **Sr. No.** | **Gene Symbol** |
| --- | --- | --- | --- | --- | --- | --- | --- |
| 1 | AHR | 31 | CXCL10 | 61 | IL4RA | 91 | PTGS2 |
| 2 | BANK1 | 32 | CXCL13 | 62 | IL4RB | 92 | PTPN1 |
| 3 | BATF | 33 | CXCL2 | 63 | IL6 | 93 | PTPN3 |
| 4 | BCL2 | 34 | CXCL9 | 64 | IL8 | 94 | RASGRP1 |
| 5 | BCL2A1 | 35 | CXCR7 | 65 | IRF1 | 95 | REL |
| 6 | BCL2L1 | 36 | DUSP1 | 66 | IRF4 | 96 | RELB |
| 7 | BIRC2 | 37 | DUSP2 | 67 | JUNB | 97 | RET |
| 8 | BIRC3 | 38 | EBI2 | 68 | KLF10 | 98 | RFTN1 |
| 9 | BUB1B | 39 | EBI3 | 69 | LITAF | 99 | RGS1 |
| 10 | C6ORF32 | 40 | EGR1 | 70 | LSP1 | 100 | RRAS2 |
| 11 | CCL2 | 41 | ELL2 | 71 | LTA | 101 | SDC4 |
| 12 | CCL22 | 42 | EMR1 | 72 | LYN | 102 | SELL |
| 13 | CCL3 | 43 | FAS | 73 | MAP3K1 | 103 | SLAMF7 |
| 14 | CCL4 | 44 | FCER | 74 | MAP3K8 | 104 | SLC2A5 |
| 15 | CCND2 | 45 | FGF12 | 75 | MIRN155 | 105 | SMAD7 |
| 16 | CCR4 | 46 | FNDC3A | 76 | MYB | 106 | SMARCA2 |
| 17 | CCR7 | 47 | GADD45B | 77 | NCF2 | 107 | SOCS2 |
| 18 | CD36 | 48 | HLA-F | 78 | NCL | 108 | SOD2 |
| 19 | CD40 | 49 | HSPA1L | 79 | NFKB1 | 109 | SPI1 |
| 20 | CD40LG | 50 | ICAM1 | 80 | NFKB2 | 110 | STAT1 |
| 21 | CD44 | 51 | ID2 | 81 | NFKBIA | 111 | STAT5A |
| 22 | CD69 | 52 | IER2 | 82 | NFKBIE | 112 | STX4 |
| 23 | CD82 | 53 | IER3 | 83 | PASK | 113 | TNF |
| 24 | CD83 | 54 | IL10 | 84 | PBEF1 | 114 | TNFAIP3 |
| 25 | CEP110 | 55 | IL12B | 85 | PECAM1 | 115 | TNIP2 |
| 26 | CFLAR | 56 | IL15RA | 86 | PIM1 | 116 | TPMT |
| 27 | CISH | 57 | IL1B | 87 | PIM2 | 117 | TRAF1 |
| 28 | CSF2 | 58 | IL2 | 88 | PLEK | 118 | VIM |
| 29 | CX3CL1 | 59 | IL2RA | 89 | PRKCD | 119 | WTAP |
| 30 | CXCL1 | 60 | IL32 | 90 | PRPF4B | 120 | ZFP36L1 |

**ST 4: Differentially expressed probesets in canine DLBCL**

| **Sr.No.** | **Probeset ID** | **Gene Symbol** | **Gene Title** | **FDR adjusted *p-value* (DLBCL Vs. Healthy)** | **Fold-Change** |
| --- | --- | --- | --- | --- | --- |
| 1 | CfaAffx.4239.1.S1_s_at | GSTA4 /// LOC606977 | glutathione S-transferase A4 /// similar to glutathione S-transferase A4 | 0.000479512 | 10.70 |
| 2 | Cfa.562.1.A1_at | --- | --- | 0.000334306 | 9.86 |
| 3 | CfaAffx.13574.1.S1_at | ACCN5 | amiloride-sensitive cation channel 5, intestinal | 1.34E-07 | 7.68 |
| 4 | Cfa.461.1.A1_at | --- | --- | 0.000116793 | 6.69 |
| 5 | CfaAffx.19526.1.S1_at | --- | --- | 3.93E-11 | 6.50 |
| 6 | CfaAffx.20516.1.S1_at | SMPX | small muscle protein, X-linked | 0.000738292 | 6.42 |
| 7 | CfaAffx.27551.1.S1_s_at | LOC490493 | similar to Myosin-9 (Myosin heavy chain, nonmuscle IIa) (Nonmuscle myosin heavy | 0.00177129 | 6.08 |
| 8 | CfaAffx.16661.1.S1_s_at | SLC38A11 | solute carrier family 38, member 11 | 0.000734301 | 6.06 |
| 9 | Cfa.11842.1.A1_s_at | GTSF1 | gametocyte specific factor 1 | 0.0146645 | 5.99 |
| 10 | Cfa.2397.1.S1_at | --- | --- | 0.000524271 | 5.91 |
| 11 | CfaAffx.14101.1.S1_s_at | BUB1B | budding uninhibited by benzimidazoles 1 homolog beta (yeast) | 2.33E-09 | 5.88 |
| 12 | Cfa.20586.1.S1_at | LOC478793 | similar to CG14853-PB, isoform B | 1.38E-05 | 5.79 |
| 13 | CfaAffx.10657.1.S1_at | GTSF1 | gametocyte specific factor 1 | 0.0146025 | 5.70 |
| 14 | Cfa.13083.1.A1_at | TTN | titin | 0.000445649 | 5.68 |
| 15 | Cfa.19154.1.S1_a_at | LOC608996 | similar to thymidylate kinase family LPS-inducible member | 0.00020144 | 5.50 |
| 16 | CfaAffx.25509.1.S1_at | NCAPG | non-SMC condensin I complex, subunit G | 1.69E-08 | 5.48 |
| 17 | CfaAffx.16824.1.S1_at | MAP2K6 | mitogen-activated protein kinase kinase 6 | 1.06E-08 | 5.44 |
| 18 | CfaAffx.9073.1.S1_s_at | LRP1B | low density lipoprotein receptor-related protein 1B | 0.00290413 | 5.40 |
| 19 | CfaAffx.27691.1.S1_s_at | ADORA2B | adenosine A2B receptor | 5.72E-13 | 5.38 |
| 20 | CfaAffx.19534.1.S1_at | CENPF | centromere protein F, 350/400ka (mitosin) | 2.43E-10 | 5.26 |
| 21 | CfaAffx.19540.1.S1_at | LOC480027 | similar to CENP-F kinetochore protein (Centromere protein F) (Mitosin) (AH antig | 2.81E-09 | 5.20 |
| 22 | CfaAffx.5187.1.S1_s_at | TTK | TTK protein kinase | 3.35E-09 | 5.17 |
| 23 | CfaAffx.13249.1.S1_at | SFRP2 | secreted frizzled-related protein 2 | 0.00211145 | 5.16 |
| 24 | Cfa.10747.1.S1_at | BCL2L14 | BCL2-like 14 (apoptosis facilitator) | 8.20E-13 | 5.14 |
| 25 | CfaAffx.26906.1.S1_at | PRR11 | proline rich 11 | 9.00E-10 | 5.11 |
| 26 | CfaAffx.24692.1.S1_at | TOP2A | topoisomerase (DNA) II alpha 170kDa | 4.55E-10 | 5.10 |
| 27 | CfaAffx.21183.1.S1_at | DNA2 | DNA replication helicase 2 homolog (yeast) | 2.00E-11 | 5.09 |
| 28 | CfaAffx.24158.1.S1_at | EXO1 | exonuclease 1 | 3.26E-09 | 5.03 |
| 29 | Cfa.11488.1.A1_at | --- | --- | 6.72E-08 | 5.02 |
| 30 | CfaAffx.21714.1.S1_s_at | FANCM | Fanconi anemia, complementation group M | 4.88E-09 | 4.94 |
| 31 | CfaAffx.26824.1.S1_s_at | KIF23 | kinesin family member 23 | 5.17E-10 | 4.93 |
| 32 | CfaAffx.20627.1.S1_at | PHEX | phosphate regulating endopeptidase homolog, X-linked | 0.00011504 | 4.90 |
| 33 | CfaAffx.8051.1.S1_at | LOC607509 /// PHYH | similar to phytanoyl-CoA hydroxylase precursor /// phytanoyl-CoA 2-hydroxylase | 0.00232996 | 4.88 |
| 34 | CfaAffx.31279.1.S1_at | DEPDC1 | DEP domain containing 1 | 2.96E-08 | 4.85 |
| 35 | CfaAffx.17007.1.S1_s_at | LOC487888 | similar to dynamin binding protein | 0.000210267 | 4.81 |
| 36 | CfaAffx.11466.1.S1_at | --- | --- | 3.14E-08 | 4.78 |
| 37 | Cfa.1200.1.S1_s_at | SFRP2 | secreted frizzled-related protein 2 | 0.00177243 | 4.75 |
| 38 | Cfa.7899.1.A1_at | PHEX | phosphate regulating endopeptidase homolog, X-linked | 0.000198826 | 4.75 |
| 39 | CfaAffx.6465.1.S1_s_at | ORC6L | origin recognition complex, subunit 6 like (yeast) | 6.32E-12 | 4.75 |
| 40 | Cfa.1822.1.A1_at | --- | --- | 0.00214847 | 4.71 |
| 41 | Cfa.322.1.S1_s_at | TPX2 | TPX2, microtubule-associated, homolog (Xenopus laevis) | 1.89E-10 | 4.70 |
| 42 | CfaAffx.20514.1.S1_s_at | --- | --- | 2.28E-10 | 4.68 |
| 43 | CfaAffx.2093.1.S1_at | GTSE1 | G-2 and S-phase expressed 1 | 1.44E-08 | 4.67 |
| 44 | Cfa.6008.1.A1_at | --- | --- | 1.24E-07 | 4.67 |
| 45 | CfaAffx.6762.1.S1_s_at | PLK4 | polo-like kinase 4 (Drosophila) | 5.50E-10 | 4.65 |
| 46 | Cfa.10306.1.S1_at | OLFM1 | olfactomedin 1 | 0.00243939 | 4.65 |
| 47 | CfaAffx.12638.1.S1_at | LOC477773 | hypothetical LOC477773 | 3.86E-08 | 4.63 |
| 48 | Cfa.20053.1.S1_s_at | KIF15 | kinesin family member 15 | 1.76E-11 | 4.57 |
| 49 | Cfa.502.1.A1_at | --- | --- | 6.17E-05 | 4.55 |
| 50 | CfaAffx.28375.1.S1_at | LOC612464 | hypothetical protein LOC612464 | 1.31E-06 | 4.54 |
| 51 | Cfa.1732.1.A1_at | --- | --- | 1.38E-07 | 4.53 |
| 52 | CfaAffx.14834.1.S1_at | CCNE2 | cyclin E2 | 1.10E-12 | 4.52 |
| 53 | CfaAffx.14145.1.S1_at | CDCA2 | cell division cycle associated 2 | 8.18E-10 | 4.48 |
| 54 | CfaAffx.24153.1.S1_s_at | SLC6A12 | solute carrier family 6 (neurotransmitter transporter, betaine/GABA), member 12 | 0.000758364 | 4.45 |
| 55 | CfaAffx.21576.1.S1_at | CDCA5 | cell division cycle associated 5 | 2.20E-11 | 4.43 |
| 56 | CfaAffx.16998.1.S1_at | LOC487888 | similar to dynamin binding protein | 0.00012459 | 4.43 |
| 57 | CfaAffx.13116.1.S1_at | MND1 | meiotic nuclear divisions 1 homolog (S. cerevisiae) | 7.82E-11 | 4.42 |
| 58 | Cfa.8552.1.A1_at | ORC1L | origin recognition complex, subunit 1-like (yeast) | 2.40E-08 | 4.42 |
| 59 | CfaAffx.25509.1.S1_s_at | NCAPG | non-SMC condensin I complex, subunit G | 1.15E-07 | 4.40 |
| 60 | CfaAffx.6499.1.S1_at | SHCBP1 | SHC SH2-domain binding protein 1 | 2.07E-08 | 4.39 |
| 61 | CfaAffx.24713.1.S1_at | CDC6 | cell division cycle 6 homolog (S. cerevisiae) | 6.74E-09 | 4.38 |
| 62 | Cfa.20194.1.S1_s_at | NUF2 | NUF2, NDC80 kinetochore complex component, homolog (S. cerevisiae) | 2.34E-08 | 4.34 |
| 63 | Cfa.103.1.A1_s_at | ASPM | asp (abnormal spindle) homolog, microcephaly associated (Drosophila) | 9.23E-09 | 4.34 |
| 64 | Cfa.1559.1.A1_at | --- | --- | 2.63E-08 | 4.31 |
| 65 | Cfa.15293.1.A1_at | KIF23 | kinesin family member 23 | 8.29E-09 | 4.30 |
| 66 | CfaAffx.20664.1.S1_at | BCL2L14 | BCL2-like 14 (apoptosis facilitator) | 2.11E-11 | 4.30 |
| 67 | CfaAffx.20375.1.S1_s_at | CDCA7 | cell division cycle associated 7 | 2.18E-09 | 4.30 |
| 68 | Cfa.13636.1.A1_s_at | MAP2K6 | mitogen-activated protein kinase kinase 6 | 6.61E-08 | 4.28 |
| 69 | CfaAffx.14867.1.S1_at | E2F8 | E2F transcription factor 8 | 4.06E-09 | 4.27 |
| 70 | CfaAffx.7218.1.S1_at | LOC607795 /// LOC609459 /// LOC609645 /// LOC609653 /// LOC609663 | similar to Rho GTPase activating protein 20 /// similar to Rho GTPase activating | 1.30E-09 | 4.26 |
| 71 | CfaAffx.20006.1.S1_at | CDK1 | cyclin-dependent kinase 1 | 3.67E-09 | 4.26 |
| 72 | CfaAffx.4092.1.S1_s_at | LOC481325 /// UBE2C | similar to ubiquitin-conjugating enzyme E2C /// ubiquitin-conjugating enzyme E2C | 1.98E-08 | 4.26 |
| 73 | CfaAffx.14530.1.S1_s_at | CASC5 | cancer susceptibility candidate 5 | 5.69E-10 | 4.24 |
| 74 | CfaAffx.24158.1.S1_s_at | EXO1 | exonuclease 1 | 1.80E-09 | 4.24 |
| 75 | CfaAffx.20507.1.S1_s_at | --- | --- | 1.13E-10 | 4.23 |
| 76 | Cfa.10049.1.A1_s_at | OXSM | 3-oxoacyl-ACP synthase, mitochondrial | 0.000665197 | 4.21 |
| 77 | CfaAffx.6465.1.S1_at | ORC6L | origin recognition complex, subunit 6 like (yeast) | 2.28E-11 | 4.18 |
| 78 | CfaAffx.21784.1.S1_at | KIF15 | kinesin family member 15 | 6.97E-11 | 4.18 |
| 79 | Cfa.1437.1.S1_at | CDC6 | cell division cycle 6 homolog (S. cerevisiae) | 3.83E-09 | 4.17 |
| 80 | Cfa.19571.1.S1_s_at | NUSAP1 | nucleolar and spindle associated protein 1 | 2.88E-10 | 4.15 |
| 81 | CfaAffx.7631.1.S1_s_at | CENPA | centromere protein A | 2.26E-08 | 4.15 |
| 82 | CfaAffx.22478.1.S1_s_at | CDC45 | cell division cycle 45 homolog (S. cerevisiae) | 3.30E-09 | 4.15 |
| 83 | CfaAffx.23012.1.S1_at | WHSC1 | Wolf-Hirschhorn syndrome candidate 1 | 3.47E-09 | 4.13 |
| 84 | CfaAffx.13120.1.S1_s_at | MND1 | meiotic nuclear divisions 1 homolog (S. cerevisiae) | 3.41E-10 | 4.12 |
| 85 | CfaAffx.23135.1.S1_s_at | DLGAP5 | discs, large (Drosophila) homolog-associated protein 5 | 9.79E-07 | 4.12 |
| 86 | CfaAffx.20664.1.S1_s_at | BCL2L14 | BCL2-like 14 (apoptosis facilitator) | 1.27E-12 | 4.10 |
| 87 | CfaAffx.18871.1.S1_at | NEK2 | NIMA (never in mitosis gene a)-related kinase 2 | 6.85E-09 | 4.09 |
| 88 | CfaAffx.27691.1.S1_at | --- | --- | 3.82E-11 | 4.07 |
| 89 | CfaAffx.24818.1.S1_s_at | TMEM138 | transmembrane protein 138 | 3.66E-09 | 4.06 |
| 90 | Cfa.9178.1.S1_at | --- | --- | 2.08E-06 | 4.06 |
| 91 | CfaAffx.9657.1.S1_s_at | SGOL1 | shugoshin-like 1 (S. pombe) | 5.40E-11 | 4.06 |
| 92 | CfaAffx.21719.1.S1_s_at | FANCM | Fanconi anemia, complementation group M | 1.11E-07 | 4.05 |
| 93 | CfaAffx.26239.1.S1_at | ERCC6L | excision repair cross-complementing rodent repair deficiency, complementation gr | 1.10E-07 | 4.05 |
| 94 | Cfa.8552.1.A1_s_at | ORC1L | origin recognition complex, subunit 1-like (yeast) | 2.38E-08 | 4.05 |
| 95 | Cfa.18508.1.S1_s_at | CCNB2 | cyclin B2 | 4.24E-09 | 4.04 |
| 96 | CfaAffx.11803.1.S1_at | PMCH | pro-melanin-concentrating hormone | 3.09E-08 | 4.04 |
| 97 | CfaAffx.6762.1.S1_at | PLK4 | polo-like kinase 4 (Drosophila) | 7.18E-10 | 4.03 |
| 98 | Cfa.5602.1.A1_at | --- | --- | 2.31E-07 | 4.02 |
| 99 | Cfa.19154.2.S1_at | LOC608996 | similar to thymidylate kinase family LPS-inducible member | 9.30E-05 | 4.00 |
| 100 | Cfa.17141.1.S1_at | KIF15 | kinesin family member 15 | 7.31E-10 | 3.99 |
| 101 | CfaAffx.28601.1.S1_s_at | SPAG5 | sperm associated antigen 5 | 2.72E-08 | 3.99 |
| 102 | CfaAffx.11492.1.S1_s_at | TPX2 | TPX2, microtubule-associated, homolog (Xenopus laevis) | 2.25E-07 | 3.98 |
| 103 | CfaAffx.6560.1.S1_at | ORC1L | origin recognition complex, subunit 1-like (yeast) | 7.47E-09 | 3.98 |
| 104 | Cfa.20734.1.S1_at | OLFM1 | olfactomedin 1 | 0.0113037 | 3.98 |
| 105 | CfaAffx.11468.1.S1_at | --- | --- | 1.07E-09 | 3.97 |
| 106 | CfaAffx.16862.1.S1_s_at | NHEDC1 | Na+/H+ exchanger domain containing 1 | 5.63E-05 | 3.95 |
| 107 | CfaAffx.13518.1.S1_s_at | NUSAP1 | nucleolar and spindle associated protein 1 | 8.83E-10 | 3.93 |
| 108 | Cfa.18367.1.S1_at | TMPO | thymopoietin | 1.76E-08 | 3.92 |
| 109 | CfaAffx.31154.1.S1_at | CENPT | centromere protein T | 8.38E-09 | 3.91 |
| 110 | Cfa.7659.1.A1_at | POLR1C | polymerase (RNA) I polypeptide C, 30kDa | 9.95E-12 | 3.89 |
| 111 | CfaAffx.30637.1.S1_at | ATMIN | ATM interactor | 1.16E-10 | 3.89 |
| 112 | Cfa.20006.1.S1_s_at | LOC479820 | similar to exonuclease NEF-sp | 2.87E-09 | 3.86 |
| 113 | CfaAffx.670.1.S1_s_at | LOC608996 | similar to thymidylate kinase family LPS-inducible member | 5.51E-05 | 3.85 |
| 114 | CfaAffx.11796.1.S1_at | LOC612196 | hypothetical protein LOC612196 | 3.91E-07 | 3.84 |
| 115 | CfaAffx.7215.1.S1_at | LOC476098 | similar to Rho GTPase activating protein 20 | 5.16E-09 | 3.83 |
| 116 | CfaAffx.10679.1.S1_at | IFITM5 | interferon induced transmembrane protein 5 | 0.000426653 | 3.83 |
| 117 | Cfa.13816.1.A1_at | TMEM138 | transmembrane protein 138 | 2.30E-08 | 3.82 |
| 118 | Cfa.521.1.A1_at | CENPA | centromere protein A | 2.71E-06 | 3.81 |
| 119 | CfaAffx.24933.1.S1_at | USP18 | ubiquitin specific peptidase 18 | 2.26E-05 | 3.81 |
| 120 | CfaAffx.12118.1.S1_s_at | KIF11 | kinesin family member 11 | 8.24E-09 | 3.80 |
| 121 | Cfa.11379.1.A1_at | ORC6L | origin recognition complex, subunit 6 like (yeast) | 1.39E-10 | 3.79 |
| 122 | CfaAffx.21443.1.S1_at | RTP4 | receptor (chemosensory) transporter protein 4 | 2.57E-05 | 3.75 |
| 123 | CfaAffx.18799.1.S1_at | SFXN4 | sideroflexin 4 | 9.77E-09 | 3.75 |
| 124 | CfaAffx.21794.1.S1_at | KIF15 | kinesin family member 15 | 4.70E-07 | 3.74 |
| 125 | Cfa.12090.1.A1_at | FOXM1 | forkhead box M1 | 1.23E-08 | 3.73 |
| 126 | Cfa.16245.1.S1_s_at | CCNB3 | cyclin B3 | 4.67E-07 | 3.72 |
| 127 | CfaAffx.30639.1.S1_s_at | ATMIN | ATM interactor | 1.33E-09 | 3.71 |
| 128 | Cfa.1928.1.S1_at | SHCBP1 | SHC SH2-domain binding protein 1 | 6.86E-08 | 3.71 |
| 129 | CfaAffx.21717.1.S1_at | FANCM | Fanconi anemia, complementation group M | 1.53E-07 | 3.70 |
| 130 | CfaAffx.9351.1.S1_s_at | --- | --- | 0.000359217 | 3.68 |
| 131 | Cfa.12793.1.A1_at | --- | --- | 1.08E-09 | 3.67 |
| 132 | Cfa.1126.1.S1_s_at | CASC5 | cancer susceptibility candidate 5 | 1.30E-08 | 3.66 |
| 133 | CfaAffx.10536.1.S1_at | BRCA2 | breast cancer 2, early onset | 1.73E-12 | 3.66 |
| 134 | CfaAffx.16434.1.S1_s_at | UBE2T | ubiquitin-conjugating enzyme E2T (putative) | 2.13E-11 | 3.66 |
| 135 | Cfa.20663.1.A1_s_at | CDK1 | cyclin-dependent kinase 1 | 2.11E-08 | 3.65 |
| 136 | CfaAffx.17007.1.S1_at | LOC487888 | similar to dynamin binding protein | 0.00125957 | 3.64 |
| 137 | Cfa.13636.1.A1_at | MAP2K6 | mitogen-activated protein kinase kinase 6 | 4.47E-07 | 3.64 |
| 138 | CfaAffx.23129.1.S1_at | DLGAP5 | discs, large (Drosophila) homolog-associated protein 5 | 1.91E-06 | 3.63 |
| 139 | Cfa.6235.1.A1_s_at | LOC481810 | similar to putative c-Myc-responsive isoform 1 | 2.32E-09 | 3.63 |
| 140 | Cfa.1128.2.S1_s_at | LOC480469 | similar to Importin alpha-2 subunit (Karyopherin alpha-2 subunit) (SRP1-alpha) ( | 1.85E-09 | 3.63 |
| 141 | Cfa.19972.1.S1_s_at | BCL2L14 | BCL2-like 14 (apoptosis facilitator) | 5.95E-13 | 3.62 |
| 142 | Cfa.18946.1.S1_s_at | TOP2A | topoisomerase (DNA) II alpha 170kDa | 2.37E-08 | 3.62 |
| 143 | Cfa.19552.1.S1_at | LOC610710 | similar to HCV NS5A-transactivated protein 9 (L5) | 5.27E-09 | 3.61 |
| 144 | CfaAffx.23580.1.S1_at | RAD51AP1 | RAD51 associated protein 1 | 3.91E-11 | 3.60 |
| 145 | CfaAffx.5338.1.S1_at | SLC4A4 | solute carrier family 4, sodium bicarbonate cotransporter, member 4 | 0.00115698 | 3.60 |
| 146 | Cfa.19618.1.S1_s_at | DKC1 | dyskeratosis congenita 1, dyskerin | 5.22E-11 | 3.60 |
| 147 | CfaAffx.5153.1.S1_at | ELOVL4 | elongation of very long chain fatty acids (FEN1/Elo2, SUR4/Elo3, yeast)-like 4 | 0.000500028 | 3.59 |
| 148 | CfaAffx.17012.1.S1_at | LOC487888 | similar to dynamin binding protein | 0.00213798 | 3.59 |
| 149 | Cfa.21191.1.S1_a_at | OAS1 | 2',5'-oligoadenylate synthetase 1, 40/46kDa | 0.0012283 | 3.58 |
| 150 | Cfa.8371.1.A1_at | --- | --- | 1.42E-05 | 3.58 |
| 151 | Cfa.1437.1.S1_s_at | CDC6 | cell division cycle 6 homolog (S. cerevisiae) | 1.09E-10 | 3.58 |
| 152 | Cfa.11939.1.A1_s_at | CCNB2 | cyclin B2 | 1.53E-07 | 3.57 |
| 153 | Cfa.1126.1.S1_at | CASC5 | cancer susceptibility candidate 5 | 3.35E-08 | 3.56 |
| 154 | CfaAffx.16008.1.S1_at | KIF18A | kinesin family member 18A | 3.37E-11 | 3.56 |
| 155 | Cfa.18038.1.S1_at | LOC476098 /// LOC607795 /// LOC609459 /// LOC609653 | similar to Rho GTPase activating protein 20 /// similar to Rho GTPase activating | 9.24E-08 | 3.56 |
| 156 | CfaAffx.3983.1.S1_at | TFPI2 | tissue factor pathway inhibitor 2 | 0.000496774 | 3.55 |
| 157 | Cfa.1979.1.A1_at | --- | --- | 0.0127724 | 3.55 |
| 158 | CfaAffx.14246.1.S1_at | --- | --- | 5.04E-08 | 3.53 |
| 159 | Cfa.231.1.A1_at | CDCA2 | cell division cycle associated 2 | 1.29E-08 | 3.53 |
| 160 | CfaAffx.13286.1.S1_at | ESCO2 | establishment of cohesion 1 homolog 2 (S. cerevisiae) | 1.99E-09 | 3.53 |
| 161 | CfaAffx.26906.1.S1_s_at | PRR11 | proline rich 11 | 2.77E-09 | 3.52 |
| 162 | CfaAffx.12575.1.S1_at | LOC486254 | similar to Kinetochore-associated protein 1 (Rough deal homolog) (hRod) (HsROD) | 3.27E-09 | 3.52 |
| 163 | CfaAffx.20529.1.S1_at | NUF2 | NUF2, NDC80 kinetochore complex component, homolog (S. cerevisiae) | 1.56E-07 | 3.51 |
| 164 | CfaAffx.6951.1.S1_at | STIL | SCL/TAL1 interrupting locus | 6.61E-10 | 3.51 |
| 165 | Cfa.13157.1.A1_at | --- | --- | 1.29E-08 | 3.51 |
| 166 | Cfa.13004.1.A1_at | --- | --- | 2.20E-06 | 3.50 |
| 167 | CfaAffx.21784.1.S1_s_at | KIF15 | kinesin family member 15 | 9.41E-11 | 3.50 |
| 168 | Cfa.20057.1.S1_s_at | KIF18A | kinesin family member 18A | 6.59E-11 | 3.49 |
| 169 | CfaAffx.3459.1.S1_at | MCM5 | minichromosome maintenance complex component 5 | 4.82E-09 | 3.49 |
| 170 | CfaAffx.16456.1.S1_s_at | OASL | 2'-5'-oligoadenylate synthetase-like | 3.04E-05 | 3.49 |
| 171 | CfaAffx.26729.1.S1_s_at | EML5 | echinoderm microtubule associated protein like 5 | 2.91E-07 | 3.49 |
| 172 | Cfa.231.1.A1_s_at | CDCA2 | cell division cycle associated 2 | 4.82E-08 | 3.49 |
| 173 | CfaAffx.1324.1.S1_at | LOC483997 | similar to DUF729 domain containing 1 | 6.65E-10 | 3.48 |
| 174 | CfaAffx.12124.1.S1_s_at | AHCY | adenosylhomocysteinase | 2.69E-11 | 3.47 |
| 175 | CfaAffx.21142.1.S1_s_at | --- | --- | 0.00175373 | 3.46 |
| 176 | CfaAffx.14862.1.S1_at | E2F8 | E2F transcription factor 8 | 6.24E-07 | 3.46 |
| 177 | CfaAffx.11791.1.S1_s_at | LOC612196 | hypothetical protein LOC612196 | 3.31E-09 | 3.45 |
| 178 | CfaAffx.16356.1.S1_s_at | HSPD1 /// LOC608756 | heat shock 60kDa protein 1 (chaperonin) /// similar to 60 kDa heat shock protein | 1.51E-12 | 3.45 |
| 179 | CfaAffx.10818.1.S1_s_at | NCAPH | non-SMC condensin I complex, subunit H | 3.53E-11 | 3.45 |
| 180 | CfaAffx.11953.1.S1_at | CENPK | centromere protein K | 7.34E-08 | 3.45 |
| 181 | CfaAffx.18536.1.S1_s_at | HSD11B1 | hydroxysteroid (11-beta) dehydrogenase 1 | 8.63E-06 | 3.45 |
| 182 | CfaAffx.16440.1.S1_at | OASL | 2'-5'-oligoadenylate synthetase-like | 9.24E-05 | 3.44 |
| 183 | CfaAffx.27461.1.S1_s_at | LOC479820 | similar to exonuclease NEF-sp | 3.04E-09 | 3.44 |
| 184 | CfaAffx.24198.1.S1_at | --- | --- | 1.86E-06 | 3.43 |
| 185 | CfaAffx.16862.1.S1_at | NHEDC1 | Na+/H+ exchanger domain containing 1 | 0.000686512 | 3.42 |
| 186 | Cfa.18904.1.S1_s_at | IFI44 | interferon-induced protein 44 | 3.22E-05 | 3.41 |
| 187 | CfaAffx.26141.1.S1_s_at | LOC610710 | similar to HCV NS5A-transactivated protein 9 (L5) | 8.73E-09 | 3.41 |
| 188 | CfaAffx.4317.1.S1_s_at | CENPP | centromere protein P | 2.92E-09 | 3.40 |
| 189 | CfaAffx.6235.1.S1_s_at | CLSPN | claspin homolog (Xenopus laevis) | 4.01E-09 | 3.39 |
| 190 | Cfa.344.1.A1_at | --- | --- | 2.54E-10 | 3.39 |
| 191 | CfaAffx.28162.1.S1_at | NDC80 | NDC80 homolog, kinetochore complex component (S. cerevisiae) | 2.96E-10 | 3.38 |
| 192 | CfaAffx.8071.1.S1_at | MCM10 | minichromosome maintenance complex component 10 | 2.09E-08 | 3.37 |
| 193 | CfaAffx.5147.1.S1_s_at | LOC481374 | hypothetical LOC481374 | 9.19E-07 | 3.36 |
| 194 | Cfa.1437.2.A1_at | --- | --- | 7.17E-09 | 3.36 |
| 195 | CfaAffx.27455.1.S1_s_at | LOC479820 | similar to exonuclease NEF-sp | 5.70E-09 | 3.36 |
| 196 | Cfa.21191.2.S1_a_at | OAS1 | 2',5'-oligoadenylate synthetase 1, 40/46kDa | 0.00185907 | 3.36 |
| 197 | CfaAffx.10629.1.S1_at | --- | --- | 8.51E-06 | 3.35 |
| 198 | Cfa.5484.1.A1_at | E2F7 | E2F transcription factor 7 | 1.63E-08 | 3.34 |
| 199 | CfaAffx.13079.1.S1_s_at | ARHGAP11A | Rho GTPase activating protein 11A | 2.01E-09 | 3.33 |
| 200 | CfaAffx.11580.1.S1_at | DEPDC1B | DEP domain containing 1B | 1.57E-09 | 3.33 |
| 201 | CfaAffx.11729.1.S1_at | CKAP2L | cytoskeleton associated protein 2-like | 2.44E-07 | 3.32 |
| 202 | CfaAffx.20503.1.S1_at | --- | --- | 3.87E-09 | 3.32 |
| 203 | Cfa.14994.1.A1_at | --- | --- | 0.0146604 | 3.32 |
| 204 | CfaAffx.13625.1.S1_at | DSN1 | DSN1, MIND kinetochore complex component, homolog (S. cerevisiae) | 1.31E-11 | 3.31 |
| 205 | Cfa.4549.1.A1_at | BIRC5 | baculoviral IAP repeat-containing 5 | 1.42E-07 | 3.31 |
| 206 | Cfa.1437.2.A1_x_at | --- | --- | 7.73E-09 | 3.30 |
| 207 | CfaAffx.16513.1.S1_s_at | CHEK1 | CHK1 checkpoint homolog (S. pombe) | 2.59E-10 | 3.28 |
| 208 | Cfa.9312.1.A1_at | --- | --- | 0.0003583 | 3.28 |
| 209 | Cfa.422.1.A1_at | --- | --- | 0.00783891 | 3.28 |
| 210 | Cfa.3933.1.A1_at | --- | --- | 0.00498816 | 3.27 |
| 211 | Cfa.1476.1.A1_at | NCAPG | non-SMC condensin I complex, subunit G | 2.00E-07 | 3.26 |
| 212 | Cfa.11113.1.A1_at | --- | --- | 6.60E-05 | 3.26 |
| 213 | CfaAffx.18128.1.S1_s_at | FANCI | Fanconi anemia, complementation group I | 4.30E-14 | 3.25 |
| 214 | CfaAffx.26219.1.S1_x_at | CKS1B | CDC28 protein kinase regulatory subunit 1B | 8.78E-12 | 3.24 |
| 215 | CfaAffx.20444.1.S1_at | GPRC5D | G protein-coupled receptor, family C, group 5, member D | 0.0101823 | 3.24 |
| 216 | CfaAffx.22581.1.S1_at | CDCA3 | cell division cycle associated 3 | 2.20E-06 | 3.23 |
| 217 | CfaAffx.8781.1.S1_s_at | NCAPG2 | non-SMC condensin II complex, subunit G2 | 1.73E-09 | 3.22 |
| 218 | CfaAffx.23752.1.S1_s_at | ECT2 | epithelial cell transforming sequence 2 oncogene | 6.17E-07 | 3.22 |
| 219 | Cfa.8472.1.A1_at | CDKN3 | cyclin-dependent kinase inhibitor 3 | 7.17E-07 | 3.21 |
| 220 | CfaAffx.6693.1.S1_at | LARP1B | La ribonucleoprotein domain family, member 1B | 6.69E-10 | 3.21 |
| 221 | Cfa.5737.1.A1_s_at | --- | --- | 2.46E-08 | 3.21 |
| 222 | CfaAffx.2699.1.S1_at | --- | --- | 4.57E-07 | 3.20 |
| 223 | CfaAffx.27495.1.S1_at | CPLX3 | complexin 3 | 2.18E-08 | 3.19 |
| 224 | Cfa.1521.1.S1_at | NUSAP1 | nucleolar and spindle associated protein 1 | 2.29E-09 | 3.19 |
| 225 | Cfa.19487.1.S1_at | LOC485338 | similar to G2/mitotic-specific cyclin B1 | 6.37E-06 | 3.18 |
| 226 | Cfa.10248.1.S1_at | HSD11B1 | hydroxysteroid (11-beta) dehydrogenase 1 | 1.66E-05 | 3.18 |
| 227 | Cfa.9868.1.A1_at | BRCA2 | breast cancer 2, early onset | 2.06E-11 | 3.17 |
| 228 | CfaAffx.22931.1.S1_at | CDKN3 | cyclin-dependent kinase inhibitor 3 | 6.03E-08 | 3.17 |
| 229 | CfaAffx.23941.1.S1_s_at | --- | --- | 2.46E-09 | 3.16 |
| 230 | Cfa.11725.1.A1_at | LOC479820 | similar to exonuclease NEF-sp | 9.46E-08 | 3.15 |
| 231 | Cfa.13994.1.S1_at | TFPI2 | tissue factor pathway inhibitor 2 | 0.000822391 | 3.15 |
| 232 | CfaAffx.11656.1.S1_at | SKA3 | spindle and kinetochore associated complex subunit 3 | 5.54E-07 | 3.14 |
| 233 | Cfa.19990.1.S1_at | LOC478258 | similar to chromosome 15 open reading frame 23 | 9.29E-09 | 3.13 |
| 234 | CfaAffx.14641.1.S1_s_at | NIT2 | nitrilase family, member 2 | 8.73E-06 | 3.13 |
| 235 | CfaAffx.19432.1.S1_at | ECE2 | endothelin converting enzyme 2 | 1.31E-09 | 3.11 |
| 236 | CfaAffx.1746.1.S1_at | FBXO43 | F-box protein 43 | 6.75E-09 | 3.10 |
| 237 | CfaAffx.19067.1.S1_s_at | DTL | denticleless homolog (Drosophila) | 1.11E-10 | 3.10 |
| 238 | Cfa.10757.1.S1_s_at | ISG15 | ISG15 ubiquitin-like modifier | 0.00168774 | 3.10 |
| 239 | Cfa.18723.1.S1_s_at | --- | --- | 1.23E-06 | 3.10 |
| 240 | Cfa.20704.1.S1_at | UBE2T | ubiquitin-conjugating enzyme E2T (putative) | 2.00E-09 | 3.10 |
| 241 | CfaAffx.4233.1.S1_at | LONRF2 | LON peptidase N-terminal domain and ring finger 2 | 0.000413102 | 3.09 |
| 242 | Cfa.21349.1.S1_s_at | TXLNB | taxilin beta | 0.000100215 | 3.09 |
| 243 | Cfa.20090.1.S1_at | --- | --- | 3.52E-06 | 3.09 |
| 244 | CfaAffx.6608.1.S1_s_at | ARMC2 | armadillo repeat containing 2 | 0.00237757 | 3.08 |
| 245 | CfaAffx.15986.1.S1_s_at | GCN1L1 | GCN1 general control of amino-acid synthesis 1-like 1 (yeast) | 0.00688566 | 3.07 |
| 246 | CfaAffx.28831.1.S1_s_at | LOC492149 | similar to CG4497-PA | 6.98E-08 | 3.07 |
| 247 | CfaAffx.8734.1.S1_s_at | FANCD2 | Fanconi anemia, complementation group D2 | 9.12E-11 | 3.06 |
| 248 | CfaAffx.26712.1.S1_at | EML5 | echinoderm microtubule associated protein like 5 | 3.03E-09 | 3.06 |
| 249 | CfaAffx.13149.1.S1_s_at | PTPRJ | protein tyrosine phosphatase, receptor type, J | 5.64E-08 | 3.06 |
| 250 | Cfa.19806.1.S1_s_at | SLC16A1 | solute carrier family 16, member 1 (monocarboxylic acid transporter 1) | 2.10E-07 | 3.05 |
| 251 | CfaAffx.14604.1.S1_s_at | LOC609892 | similar to Trifunctional purine biosynthetic protein adenosine-3 | 2.44E-08 | 3.04 |
| 252 | CfaAffx.7108.1.S1_at | CCNA2 | cyclin A2 | 4.03E-06 | 3.04 |
| 253 | CfaAffx.9490.1.S1_s_at | SLC23A1 | solute carrier family 23 (nucleobase transporters), member 1 | 1.72E-06 | 3.04 |
| 254 | Cfa.19154.1.S1_at | LOC608996 | similar to thymidylate kinase family LPS-inducible member | 0.000105287 | 3.03 |
| 255 | CfaAffx.5344.1.S1_at | SLC4A4 | solute carrier family 4, sodium bicarbonate cotransporter, member 4 | 0.00174991 | 3.03 |
| 256 | CfaAffx.8309.1.S1_s_at | DIAPH3 | diaphanous homolog 3 (Drosophila) | 4.96E-08 | 3.03 |
| 257 | CfaAffx.7499.1.S1_s_at | MASTL | microtubule associated serine/threonine kinase-like | 8.28E-09 | 3.01 |
| 258 | CfaAffx.17004.1.S1_at | CEP72 | centrosomal protein 72kDa | 8.53E-08 | 3.01 |
| 259 | CfaAffx.15042.1.S1_s_at | SPP1 | secreted phosphoprotein 1 | 0.00569495 | 3.01 |
| 260 | Cfa.9662.1.A1_at | --- | --- | 3.07E-08 | 3.01 |
| 261 | Cfa.13111.1.A1_at | --- | --- | 8.91E-08 | 3.01 |
| 262 | CfaAffx.12419.1.S1_s_at | LOC485338 | similar to G2/mitotic-specific cyclin B1 | 6.30E-06 | 3.01 |
| 263 | Cfa.10757.1.S1_at | ISG15 | ISG15 ubiquitin-like modifier | 0.0003983 | 3.00 |
| 264 | Cfa.4343.1.S1_s_at | MCM5 | minichromosome maintenance complex component 5 | 1.83E-08 | 3.00 |
| 265 | Cfa.16671.1.S1_at | --- | --- | 2.63E-08 | 2.99 |
| 266 | CfaAffx.30398.1.S1_s_at | OLFM1 | olfactomedin 1 | 0.00102863 | 2.99 |
| 267 | Cfa.3066.1.S1_s_at | NDC80 | NDC80 homolog, kinetochore complex component (S. cerevisiae) | 4.42E-10 | 2.99 |
| 268 | CfaAffx.9145.1.S1_at | KYNU | kynureninase (L-kynurenine hydrolase) | 2.26E-05 | 2.99 |
| 269 | Cfa.20663.1.A1_at | CDK1 | cyclin-dependent kinase 1 | 2.78E-07 | 2.98 |
| 270 | CfaAffx.18587.1.S1_at | LOC609269 | similar to RIKEN cDNA 5730590G19-like | 2.21E-08 | 2.98 |
| 271 | CfaAffx.24518.1.S1_at | FADS1 | fatty acid desaturase 1 | 5.57E-08 | 2.98 |
| 272 | Cfa.17965.1.S1_s_at | PAICS | phosphoribosylaminoimidazole carboxylase, phosphoribosylaminoimidazole succinoca | 4.03E-11 | 2.97 |
| 273 | Cfa.10452.1.S1_s_at | CDCA2 | cell division cycle associated 2 | 1.04E-09 | 2.97 |
| 274 | CfaAffx.19651.1.S1_s_at | STRAP | serine/threonine kinase receptor associated protein | 3.99E-08 | 2.97 |
| 275 | CfaAffx.14370.1.S1_at | LOC478258 | similar to chromosome 15 open reading frame 23 | 5.57E-09 | 2.97 |
| 276 | Cfa.19423.1.S1_at | LOC490715 | hypothetical LOC490715 | 0.000221116 | 2.96 |
| 277 | CfaAffx.4188.1.S1_s_at | MCM3 | minichromosome maintenance complex component 3 | 3.46E-13 | 2.96 |
| 278 | Cfa.16493.1.A1_at | RPS20 | ribosomal protein S20 | 6.70E-10 | 2.95 |
| 279 | Cfa.19165.1.S1_s_at | NEK2 | NIMA (never in mitosis gene a)-related kinase 2 | 2.79E-08 | 2.95 |
| 280 | Cfa.10486.1.A1_at | --- | --- | 2.66E-09 | 2.95 |
| 281 | Cfa.15269.1.A1_at | WHSC1 | Wolf-Hirschhorn syndrome candidate 1 | 2.90E-09 | 2.95 |
| 282 | CfaAffx.7879.1.S1_at | LOC485160 | hypothetical LOC485160 | 1.55E-06 | 2.94 |
| 283 | CfaAffx.28972.1.S1_at | PRKAA2 | protein kinase, AMP-activated, alpha 2 catalytic subunit | 3.70E-07 | 2.93 |
| 284 | Cfa.14211.1.A1_at | --- | --- | 0.000977945 | 2.93 |
| 285 | CfaAffx.6627.1.S1_at | LOC475018 | similar to Protein C6orf182 homolog | 1.92E-08 | 2.93 |
| 286 | Cfa.14178.1.S1_at | SLC5A6 | solute carrier family 5 (sodium-dependent vitamin transporter), member 6 | 1.97E-11 | 2.92 |
| 287 | CfaAffx.18190.1.S1_s_at | --- | --- | 0.00797908 | 2.92 |
| 288 | CfaAffx.18494.1.S1_at | --- | --- | 3.78E-08 | 2.91 |
| 289 | CfaAffx.13263.1.S1_at | RACGAP1 | Rac GTPase activating protein 1 | 2.87E-08 | 2.91 |
| 290 | Cfa.19915.1.S1_at | GALNTL5 | UDP-N-acetyl-alpha-D-galactosamine:polypeptide N-acetylgalactosaminyltransferase | 3.54E-05 | 2.90 |
| 291 | CfaAffx.24036.1.S1_at | MYO5C | myosin VC | 1.74E-05 | 2.90 |
| 292 | CfaAffx.10071.1.S1_s_at | EIF2AK2 | eukaryotic translation initiation factor 2-alpha kinase 2 | 7.56E-05 | 2.88 |
| 293 | CfaAffx.1382.1.S1_at | TXLNB | taxilin beta | 4.26E-05 | 2.88 |
| 294 | Cfa.16228.1.S1_s_at | MSH2 | mutS homolog 2, colon cancer, nonpolyposis type 1 (E. coli) | 2.95E-09 | 2.88 |
| 295 | CfaAffx.24091.1.S1_at | OPN3 | opsin 3 | 2.01E-06 | 2.86 |
| 296 | CfaAffx.16935.1.S1_at | TRIP13 | thyroid hormone receptor interactor 13 | 1.84E-09 | 2.85 |
| 297 | CfaAffx.25584.1.S1_at | LOC608702 | similar to Spermidine synthase (Putrescine aminopropyltransferase) (SPDSY) | 1.86E-07 | 2.85 |
| 298 | CfaAffx.11803.1.S1_s_at | PMCH | pro-melanin-concentrating hormone | 2.01E-08 | 2.85 |
| 299 | Cfa.11525.1.A1_at | --- | --- | 9.88E-09 | 2.85 |
| 300 | Cfa.19508.1.A1_at | LOC477353 | similar to ankyrin repeat domain 26 | 0.000811759 | 2.84 |
| 301 | CfaAffx.31148.1.S1_at | IFI44 | interferon-induced protein 44 | 0.000166718 | 2.83 |
| 302 | Cfa.19854.1.S1_at | PRMT3 | protein arginine methyltransferase 3 | 2.65E-14 | 2.83 |
| 303 | Cfa.6991.1.A1_at | --- | --- | 1.38E-05 | 2.83 |
| 304 | CfaAffx.5845.1.S1_at | CDCA8 | cell division cycle associated 8 | 1.66E-09 | 2.83 |
| 305 | CfaAffx.28874.1.S1_at | LOC609907 | hypothetical protein LOC609907 | 5.31E-08 | 2.82 |
| 306 | Cfa.9124.1.A1_at | --- | --- | 0.000520045 | 2.81 |
| 307 | CfaAffx.20855.1.S1_at | RNF2 | ring finger protein 2 | 2.28E-10 | 2.81 |
| 308 | Cfa.15094.1.S1_at | NME1 /// NME2 | non-metastatic cells 1, protein (NM23A) expressed in /// non-metastatic cells 2, | 2.80E-08 | 2.81 |
| 309 | Cfa.15460.1.A1_at | CENPP | centromere protein P | 3.55E-07 | 2.80 |
| 310 | CfaAffx.20340.1.S1_at | LOC609308 | hypothetical protein LOC609308 | 3.29E-09 | 2.80 |
| 311 | Cfa.15622.1.A1_s_at | RND1 | Rho family GTPase 1 | 2.05E-06 | 2.80 |
| 312 | Cfa.18892.1.S1_s_at | IFI44L | interferon-induced protein 44-like | 0.00269933 | 2.80 |
| 313 | Cfa.16512.1.S1_s_at | CDKN3 | cyclin-dependent kinase inhibitor 3 | 1.55E-07 | 2.80 |
| 314 | Cfa.10704.1.S1_at | --- | --- | 2.69E-08 | 2.80 |
| 315 | CfaAffx.1910.1.S1_s_at | SLC25A32 | solute carrier family 25, member 32 | 2.63E-09 | 2.80 |
| 316 | Cfa.4967.1.A1_at | --- | --- | 1.92E-08 | 2.79 |
| 317 | Cfa.18569.1.S1_s_at | LOC608051 | similar to tubulin, alpha 2 isoform 2 | 0.0149714 | 2.78 |
| 318 | CfaAffx.12430.1.S1_at | CENPH | centromere protein H | 1.95E-09 | 2.78 |
| 319 | CfaAffx.18174.1.S1_at | --- | --- | 0.0167642 | 2.77 |
| 320 | CfaAffx.16240.1.S1_s_at | ALDH5A1 | aldehyde dehydrogenase 5 family, member A1 | 2.10E-09 | 2.77 |
| 321 | Cfa.11769.1.A1_s_at | LOC475733 | similar to protein containing single MORN motif in testis | 4.10E-09 | 2.76 |
| 322 | CfaAffx.13280.1.S1_at | PBK | PDZ binding kinase | 8.06E-08 | 2.76 |
| 323 | CfaAffx.1747.1.S1_at | FBXO5 | F-box protein 5 | 8.61E-06 | 2.76 |
| 324 | Cfa.18120.1.S1_s_at | SEH1L | SEH1-like (S. cerevisiae) | 2.30E-12 | 2.76 |
| 325 | CfaAffx.4093.1.S1_at | CENPQ | centromere protein Q | 6.07E-08 | 2.76 |
| 326 | CfaAffx.6059.1.S1_at | RRM2 | ribonucleotide reductase M2 | 1.88E-05 | 2.76 |
| 327 | CfaAffx.4735.1.S1_at | UBE2S | ubiquitin-conjugating enzyme E2S | 4.11E-06 | 2.76 |
| 328 | Cfa.7415.1.A1_at | LOC478258 | similar to chromosome 15 open reading frame 23 | 4.80E-07 | 2.76 |
| 329 | CfaAffx.21313.1.S1_at | CCR2 | chemokine (C-C motif) receptor 2 | 7.03E-05 | 2.75 |
| 330 | CfaAffx.26921.1.S1_at | CENPI | centromere protein I | 2.17E-09 | 2.75 |
| 331 | CfaAffx.7006.1.S1_s_at | MCM2 | minichromosome maintenance complex component 2 | 4.22E-09 | 2.74 |
| 332 | Cfa.21627.1.S1_s_at | MTHFD1 | methylenetetrahydrofolate dehydrogenase (NADP+ dependent) 1, methenyltetrahydrof | 1.36E-10 | 2.73 |
| 333 | CfaAffx.13861.1.S1_s_at | ATP6V0D2 | ATPase, H+ transporting, lysosomal 38kDa, V0 subunit d2 | 0.00603342 | 2.73 |
| 334 | CfaAffx.28075.1.S1_at | LOC607417 | similar to Thymidylate synthase (TS) (TSase) | 1.23E-11 | 2.73 |
| 335 | CfaAffx.23072.1.S1_s_at | LOC608051 | similar to tubulin, alpha 2 isoform 2 | 0.0131129 | 2.73 |
| 336 | CfaAffx.16308.1.S1_s_at | TUBA3E | tubulin, alpha 3e | 3.45E-08 | 2.73 |
| 337 | CfaAffx.15377.1.S1_at | HERC5 | hect domain and RLD 5 | 0.00205679 | 2.72 |
| 338 | Cfa.3422.1.A1_at | --- | --- | 0.000110941 | 2.72 |
| 339 | Cfa.2871.1.A1_at | LOC481374 | hypothetical LOC481374 | 6.23E-06 | 2.72 |
| 340 | Cfa.11435.1.A1_at | --- | --- | 0.000716375 | 2.72 |
| 341 | CfaAffx.22661.1.S1_s_at | GRK4 | G protein-coupled receptor kinase 4 | 0.00149956 | 2.71 |
| 342 | Cfa.8441.1.A1_at | --- | --- | 1.15E-06 | 2.71 |
| 343 | Cfa.20105.1.S1_s_at | PRMT5 | protein arginine methyltransferase 5 | 5.59E-11 | 2.71 |
| 344 | CfaAffx.5747.1.S1_at | UTP11L | UTP11-like, U3 small nucleolar ribonucleoprotein, (yeast) | 0.000835271 | 2.70 |
| 345 | CfaAffx.15121.1.S1_at | IFIT1 | interferon-induced protein with tetratricopeptide repeats 1 | 0.00544636 | 2.70 |
| 346 | CfaAffx.8177.1.S1_at | SLC5A6 | solute carrier family 5 (sodium-dependent vitamin transporter), member 6 | 3.97E-11 | 2.69 |
| 347 | CfaAffx.15301.1.S1_at | UBE2C | ubiquitin-conjugating enzyme E2C | 3.46E-07 | 2.68 |
| 348 | Cfa.19631.1.S1_at | PRC1 | protein regulator of cytokinesis 1 | 3.57E-06 | 2.68 |
| 349 | Cfa.2696.1.S1_at | --- | --- | 1.18E-11 | 2.68 |
| 350 | CfaAffx.27290.1.S1_at | --- | --- | 1.16E-08 | 2.67 |
| 351 | CfaAffx.13617.1.S1_s_at | TROAP | trophinin associated protein (tastin) | 2.37E-07 | 2.67 |
| 352 | CfaAffx.15493.1.S1_s_at | LOC484905 | similar to anti-silencing function 1B | 5.88E-10 | 2.67 |
| 353 | Cfa.9276.1.A1_at | --- | --- | 5.99E-06 | 2.67 |
| 354 | Cfa.20160.1.S1_at | FANCD2 | Fanconi anemia, complementation group D2 | 7.76E-10 | 2.67 |
| 355 | CfaAffx.31150.1.S1_at | IFI44L | interferon-induced protein 44-like | 0.00199893 | 2.67 |
| 356 | Cfa.19990.1.S1_s_at | LOC478258 | similar to chromosome 15 open reading frame 23 | 1.43E-07 | 2.67 |
| 357 | CfaAffx.8850.1.S1_s_at | CDC20 | cell division cycle 20 homolog (S. cerevisiae) | 8.07E-07 | 2.66 |
| 358 | CfaAffx.7956.1.S1_at | --- | --- | 0.00952977 | 2.66 |
| 359 | CfaAffx.4364.1.S1_s_at | MELK | maternal embryonic leucine zipper kinase | 3.41E-10 | 2.66 |
| 360 | CfaAffx.13612.1.S1_at | TROAP | trophinin associated protein (tastin) | 2.00E-06 | 2.65 |
| 361 | CfaAffx.20589.1.S1_at | DNAJC12 | DnaJ (Hsp40) homolog, subfamily C, member 12 | 0.000862625 | 2.65 |
| 362 | Cfa.20597.1.S1_s_at | KIF22 | kinesin family member 22 | 4.73E-07 | 2.65 |
| 363 | CfaAffx.11945.1.S1_s_at | RPP14 | ribonuclease P/MRP 14kDa subunit | 3.84E-10 | 2.65 |
| 364 | CfaAffx.3455.1.S1_s_at | LOC476320 | similar to CG11596-PA, isoform A | 5.05E-09 | 2.65 |
| 365 | CfaAffx.5147.1.S1_at | LOC481374 | hypothetical LOC481374 | 1.21E-06 | 2.65 |
| 366 | CfaAffx.13588.1.S1_at | TDO2 | tryptophan 2,3-dioxygenase | 0.000186232 | 2.64 |
| 367 | Cfa.1603.1.A1_at | PCYT1A | phosphate cytidylyltransferase 1, choline, alpha | 3.15E-07 | 2.64 |
| 368 | CfaAffx.5562.1.S1_at | LOC481385 | hypothetical LOC481385 | 1.47E-08 | 2.64 |
| 369 | CfaAffx.6631.1.S1_s_at | LOC475018 | similar to Protein C6orf182 homolog | 5.49E-08 | 2.64 |
| 370 | CfaAffx.18859.1.S1_at | --- | --- | 4.09E-11 | 2.64 |
| 371 | CfaAffx.2299.1.S1_s_at | TTLL1 | tubulin tyrosine ligase-like family, member 1 | 1.41E-09 | 2.64 |
| 372 | CfaAffx.5545.1.S1_at | EPGN | epithelial mitogen homolog (mouse) | 3.95E-09 | 2.63 |
| 373 | Cfa.19031.1.S1_s_at | ATG4C | ATG4 autophagy related 4 homolog C (S. cerevisiae) | 2.78E-07 | 2.63 |
| 374 | CfaAffx.20342.1.S1_s_at | LOC609308 | hypothetical protein LOC609308 | 6.63E-09 | 2.63 |
| 375 | Cfa.19423.1.S1_s_at | LOC490715 | hypothetical LOC490715 | 0.000246353 | 2.62 |
| 376 | CfaAffx.8304.1.S1_at | DIAPH3 | diaphanous homolog 3 (Drosophila) | 9.92E-07 | 2.62 |
| 377 | CfaAffx.16869.1.S1_at | SPATA17 | spermatogenesis associated 17 | 4.20E-05 | 2.62 |
| 378 | CfaAffx.5317.1.S1_at | DCK | deoxycytidine kinase | 6.84E-06 | 2.62 |
| 379 | Cfa.9240.1.S1_at | SPP1 | secreted phosphoprotein 1 | 0.0203707 | 2.62 |
| 380 | Cfa.7427.1.A1_s_at | GRK4 | G protein-coupled receptor kinase 4 | 0.00221318 | 2.61 |
| 381 | CfaAffx.4220.1.S1_at | PPAT | phosphoribosyl pyrophosphate amidotransferase | 2.28E-08 | 2.61 |
| 382 | CfaAffx.21924.1.S1_at | POU2AF1 | POU class 2 associating factor 1 | 7.93E-05 | 2.61 |
| 383 | CfaAffx.15972.1.S1_at | LOC607371 | hypothetical protein LOC607371 | 6.12E-07 | 2.61 |
| 384 | CfaAffx.1913.1.S1_s_at | SLC25A32 | solute carrier family 25, member 32 | 3.98E-08 | 2.61 |
| 385 | Cfa.33.2.S1_s_at | TPMT | thiopurine S-methyltransferase | 8.62E-08 | 2.61 |
| 386 | CfaAffx.14735.1.S1_at | MYBL2 | v-myb myeloblastosis viral oncogene homolog (avian)-like 2 | 1.23E-06 | 2.61 |
| 387 | Cfa.18463.1.S1_s_at | AURKA | aurora kinase A | 7.25E-07 | 2.60 |
| 388 | CfaAffx.15427.1.S1_s_at | PRMT3 | protein arginine methyltransferase 3 | 8.45E-13 | 2.60 |
| 389 | CfaAffx.14483.1.S1_s_at | RAD54B | RAD54 homolog B (S. cerevisiae) | 1.67E-07 | 2.60 |
| 390 | Cfa.11046.1.A1_s_at | OMG | oligodendrocyte myelin glycoprotein | 0.000475515 | 2.60 |
| 391 | CfaAffx.13594.1.S1_at | CTSK | cathepsin K | 1.29E-05 | 2.60 |
| 392 | Cfa.13912.1.A1_at | RRM2 | ribonucleotide reductase M2 | 1.62E-05 | 2.60 |
| 393 | Cfa.8051.1.A1_at | --- | --- | 1.65E-09 | 2.60 |
| 394 | Cfa.18211.1.A1_s_at | LOC480414 | similar to CG11638-PA | 1.28E-06 | 2.60 |
| 395 | CfaAffx.987.1.S1_at | TXNL4A | thioredoxin-like 4A | 1.78E-09 | 2.59 |
| 396 | CfaAffx.2723.1.S1_s_at | CDC25C | cell division cycle 25 homolog C (S. pombe) | 1.58E-07 | 2.59 |
| 397 | CfaAffx.17382.1.S1_at | KIF14 | kinesin family member 14 | 2.78E-05 | 2.59 |
| 398 | Cfa.17139.1.S1_at | SREBF2 | sterol regulatory element binding transcription factor 2 | 0.000478866 | 2.59 |
| 399 | CfaAffx.25113.1.S1_at | PLEK2 | pleckstrin 2 | 0.000264077 | 2.58 |
| 400 | CfaAffx.27569.1.S1_at | KLHL14 | kelch-like 14 (Drosophila) | 0.00580854 | 2.58 |
| 401 | CfaAffx.28602.1.S1_s_at | ATG4C | ATG4 autophagy related 4 homolog C (S. cerevisiae) | 4.39E-08 | 2.58 |
| 402 | Cfa.21474.1.S1_s_at | WDR46 | WD repeat domain 46 | 4.33E-11 | 2.58 |
| 403 | Cfa.1885.1.A1_at | --- | --- | 1.28E-06 | 2.58 |
| 404 | Cfa.13211.1.A1_at | TDO2 | tryptophan 2,3-dioxygenase | 0.000255776 | 2.57 |
| 405 | Cfa.3012.1.A1_at | --- | --- | 0.016983 | 2.57 |
| 406 | CfaAffx.2381.1.S1_at | KIFC1 | kinesin family member C1 | 4.18E-07 | 2.57 |
| 407 | CfaAffx.20019.1.S1_s_at | LOC488399 | similar to methionine aminopeptidase 1D | 4.15E-08 | 2.57 |
| 408 | CfaAffx.4706.1.S1_at | RIMKLA | ribosomal modification protein rimK-like family member A | 2.51E-05 | 2.56 |
| 409 | Cfa.6748.1.A1_at | WHSC1 | Wolf-Hirschhorn syndrome candidate 1 | 1.00E-08 | 2.56 |
| 410 | CfaAffx.4424.1.S1_at | GRHPR | glyoxylate reductase/hydroxypyruvate reductase | 1.57E-07 | 2.56 |
| 411 | Cfa.20748.1.S1_s_at | GNL2 | guanine nucleotide binding protein-like 2 (nucleolar) | 5.75E-11 | 2.56 |
| 412 | CfaAffx.15482.1.S1_s_at | DNAH5 | dynein, axonemal, heavy chain 5 | 0.00442269 | 2.56 |
| 413 | Cfa.11882.1.A1_at | LOC481385 | hypothetical LOC481385 | 3.66E-09 | 2.55 |
| 414 | CfaAffx.28412.1.S1_s_at | OXCT1 | 3-oxoacid CoA transferase 1 | 1.97E-07 | 2.55 |
| 415 | Cfa.21183.1.S1_s_at | OAS1 | 2',5'-oligoadenylate synthetase 1, 40/46kDa | 7.08E-05 | 2.54 |
| 416 | Cfa.15745.1.A1_at | SFXN4 | sideroflexin 4 | 3.39E-05 | 2.54 |
| 417 | Cfa.18685.1.S1_s_at | MTHFD1 | methylenetetrahydrofolate dehydrogenase (NADP+ dependent) 1, methenyltetrahydrof | 1.10E-08 | 2.54 |
| 418 | Cfa.13716.1.A1_a_at | LOC479395 | hypothetical LOC479395 | 0.00588269 | 2.54 |
| 419 | CfaAffx.8348.1.S1_at | DDX18 | DEAD (Asp-Glu-Ala-Asp) box polypeptide 18 | 3.02E-09 | 2.54 |
| 420 | Cfa.16245.1.S1_at | CCNB3 | cyclin B3 | 3.95E-06 | 2.54 |
| 421 | Cfa.15799.1.S1_s_at | FUT7 | fucosyltransferase 7 (alpha (1,3) fucosyltransferase) | 0.000416135 | 2.53 |
| 422 | Cfa.20058.1.S1_at | SERPINB5 | serpin peptidase inhibitor, clade B (ovalbumin), member 5 | 2.06E-10 | 2.53 |
| 423 | Cfa.17226.1.S1_at | SLC5A6 | Solute carrier family 5 (sodium-dependent vitamin transporter), member 6 | 1.19E-10 | 2.53 |
| 424 | CfaAffx.29135.1.S1_s_at | --- | --- | 1.80E-06 | 2.53 |
| 425 | CfaAffx.21393.1.S1_at | A2ML1 | alpha-2-macroglobulin-like 1 | 0.00608469 | 2.53 |
| 426 | Cfa.19427.1.S1_s_at | CDC7 | cell division cycle 7 homolog (S. cerevisiae) | 1.97E-08 | 2.52 |
| 427 | CfaAffx.18282.1.S1_s_at | ATRNL1 | attractin-like 1 | 0.000163428 | 2.52 |
| 428 | CfaAffx.2353.1.S1_at | RRP7A | ribosomal RNA processing 7 homolog A (S. cerevisiae) | 2.69E-06 | 2.52 |
| 429 | Cfa.4009.1.A1_at | --- | --- | 9.79E-07 | 2.52 |
| 430 | Cfa.5876.1.A1_at | POLA1 | polymerase (DNA directed), alpha 1, catalytic subunit | 2.65E-05 | 2.52 |
| 431 | Cfa.5378.1.A1_at | --- | --- | 7.02E-07 | 2.52 |
| 432 | Cfa.7182.1.S1_s_at | OAZ3 | ornithine decarboxylase antizyme 3 | 0.00225602 | 2.52 |
| 433 | CfaAffx.25421.1.S1_at | LOC611683 | similar to Protein C14orf112 precursor | 8.55E-09 | 2.51 |
| 434 | Cfa.17732.1.S1_s_at | PMS1 | PMS1 postmeiotic segregation increased 1 (S. cerevisiae) | 8.21E-08 | 2.51 |
| 435 | CfaAffx.14811.1.S1_at | PMS1 | PMS1 postmeiotic segregation increased 1 (S. cerevisiae) | 2.05E-11 | 2.51 |
| 436 | CfaAffx.16943.1.S1_s_at | LOC478498 | similar to centromere protein E | 9.60E-07 | 2.51 |
| 437 | Cfa.1028.1.A1_s_at | PAICS | phosphoribosylaminoimidazole carboxylase, phosphoribosylaminoimidazole succinoca | 4.87E-10 | 2.51 |
| 438 | CfaAffx.4185.1.S1_at | MCM3 | minichromosome maintenance complex component 3 | 2.34E-12 | 2.50 |
| 439 | CfaAffx.27008.1.S1_at | SENP8 | SUMO/sentrin specific peptidase family member 8 | 1.78E-11 | 2.50 |
| 440 | CfaAffx.15004.1.S1_s_at | TMEM169 | transmembrane protein 169 | 1.91E-07 | 2.50 |
| 441 | CfaAffx.15427.1.S1_at | PRMT3 | protein arginine methyltransferase 3 | 2.84E-11 | 2.50 |
| 442 | CfaAffx.7664.1.S1_s_at | CACNB2 | calcium channel, voltage-dependent, beta 2 subunit | 0.000205841 | 2.50 |
| 443 | CfaAffx.29929.1.S1_at | ACOT7 | acyl-CoA thioesterase 7 | 0.00101121 | 2.49 |
| 444 | Cfa.15181.1.A1_at | DKC1 | dyskeratosis congenita 1, dyskerin | 2.80E-09 | 2.49 |
| 445 | CfaAffx.18502.1.S1_at | AURKA | aurora kinase A | 2.77E-07 | 2.49 |
| 446 | CfaAffx.7759.1.S1_at | LOC482790 | similar to zinc finger, BED domain containing 4 | 0.000347058 | 2.48 |
| 447 | Cfa.13658.1.A1_at | --- | --- | 0.000113856 | 2.48 |
| 448 | Cfa.7394.1.A1_at | --- | --- | 0.00314187 | 2.48 |
| 449 | Cfa.3370.1.S1_at | LOC476320 | similar to CG11596-PA, isoform A | 7.61E-11 | 2.48 |
| 450 | CfaAffx.12634.1.S1_s_at | LOC403525 | hypothetical protein LOC403525 | 7.71E-12 | 2.48 |
| 451 | CfaAffx.14683.1.S1_s_at | ESRP1 | epithelial splicing regulatory protein 1 | 0.000617746 | 2.48 |
| 452 | CfaAffx.26479.1.S1_x_at | NME2 | non-metastatic cells 2, protein (NM23B) expressed in | 4.93E-08 | 2.48 |
| 453 | CfaAffx.12767.1.S1_at | BLK | B lymphoid tyrosine kinase | 1.37E-06 | 2.47 |
| 454 | CfaAffx.4202.1.S1_s_at | AASDH | aminoadipate-semialdehyde dehydrogenase | 1.54E-07 | 2.47 |
| 455 | CfaAffx.26814.1.S1_at | SPC24 | SPC24, NDC80 kinetochore complex component, homolog (S. cerevisiae) | 4.75E-07 | 2.47 |
| 456 | CfaAffx.17004.1.S1_s_at | CEP72 | centrosomal protein 72kDa | 5.99E-07 | 2.47 |
| 457 | Cfa.6235.1.A1_at | LOC481810 | similar to putative c-Myc-responsive isoform 1 | 2.98E-08 | 2.47 |
| 458 | CfaAffx.28412.1.S1_at | OXCT1 | 3-oxoacid CoA transferase 1 | 8.56E-07 | 2.46 |
| 459 | CfaAffx.6880.1.S1_s_at | DHFR | dihydrofolate reductase | 5.84E-11 | 2.46 |
| 460 | Cfa.21134.1.S1_s_at | GTPBP4 | GTP binding protein 4 | 8.87E-10 | 2.46 |
| 461 | Cfa.9305.1.A1_at | --- | --- | 3.17E-06 | 2.46 |
| 462 | CfaAffx.24086.1.S1_at | KMO | kynurenine 3-monooxygenase (kynurenine 3-hydroxylase) | 5.17E-05 | 2.46 |
| 463 | CfaAffx.14645.1.S1_at | NIT2 | nitrilase family, member 2 | 0.000470209 | 2.45 |
| 464 | Cfa.6200.1.A1_at | LOC606957 | similar to XIAP associated factor-1 isoform 2 | 0.0014082 | 2.45 |
| 465 | Cfa.18211.1.A1_at | LOC480414 | similar to CG11638-PA | 0.000193332 | 2.45 |
| 466 | CfaAffx.23014.1.S1_s_at | WHSC1 | Wolf-Hirschhorn syndrome candidate 1 | 1.48E-10 | 2.45 |
| 467 | CfaAffx.16314.1.S1_s_at | GMNN | geminin, DNA replication inhibitor | 7.48E-08 | 2.45 |
| 468 | CfaAffx.13280.1.S1_s_at | PBK | PDZ binding kinase | 3.84E-08 | 2.45 |
| 469 | Cfa.11149.1.A1_at | --- | --- | 8.62E-08 | 2.44 |
| 470 | CfaAffx.21322.1.S1_at | DDX21 | DEAD (Asp-Glu-Ala-Asp) box polypeptide 21 | 2.68E-08 | 2.44 |
| 471 | Cfa.15826.1.S1_s_at | BIRC5 | baculoviral IAP repeat-containing 5 | 1.15E-05 | 2.44 |
| 472 | CfaAffx.9496.1.S1_at | --- | --- | 0.00024472 | 2.44 |
| 473 | CfaAffx.10362.1.S1_s_at | POLE | polymerase (DNA directed), epsilon | 1.15E-07 | 2.44 |
| 474 | CfaAffx.24419.1.S1_s_at | ADIPOR2 | adiponectin receptor 2 | 7.57E-07 | 2.44 |
| 475 | Cfa.13618.1.A1_at | MAP2K6 | mitogen-activated protein kinase kinase 6 | 1.31E-05 | 2.43 |
| 476 | Cfa.21021.1.S1_s_at | SLC35F5 | solute carrier family 35, member F5 | 0.00123631 | 2.43 |
| 477 | CfaAffx.8348.1.S1_s_at | DDX18 | DEAD (Asp-Glu-Ala-Asp) box polypeptide 18 | 4.18E-11 | 2.43 |
| 478 | CfaAffx.22400.1.S1_s_at | CENPL | centromere protein L | 4.44E-07 | 2.43 |
| 479 | CfaAffx.22615.1.S1_at | SLC35F2 | solute carrier family 35, member F2 | 0.000105058 | 2.43 |
| 480 | CfaAffx.20671.1.S1_at | --- | --- | 4.56E-06 | 2.43 |
| 481 | CfaAffx.18859.1.S1_s_at | --- | --- | 3.41E-11 | 2.42 |
| 482 | Cfa.19413.1.S1_s_at | EIF3B | eukaryotic translation initiation factor 3, subunit B | 3.55E-10 | 2.42 |
| 483 | Cfa.18640.1.S1_s_at | ACSL5 | acyl-CoA synthetase long-chain family member 5 | 2.73E-06 | 2.42 |
| 484 | CfaAffx.18978.1.S1_at | INTS7 | integrator complex subunit 7 | 2.21E-11 | 2.42 |
| 485 | Cfa.21191.1.S1_at | OAS1 | 2',5'-oligoadenylate synthetase 1, 40/46kDa | 0.00988083 | 2.42 |
| 486 | Cfa.11984.1.A1_at | MIS12 | MIS12, MIND kinetochore complex component, homolog (S. pombe) | 3.16E-06 | 2.42 |
| 487 | Cfa.3414.1.A1_at | MYBL2 | v-myb myeloblastosis viral oncogene homolog (avian)-like 2 | 4.05E-05 | 2.41 |
| 488 | CfaAffx.16245.1.S1_s_at | ALDH5A1 | aldehyde dehydrogenase 5 family, member A1 | 4.35E-09 | 2.41 |
| 489 | Cfa.10070.1.A1_at | --- | --- | 4.38E-08 | 2.41 |
| 490 | CfaAffx.30321.1.S1_s_at | FANCA | Fanconi anemia, complementation group A | 7.98E-07 | 2.41 |
| 491 | CfaAffx.2298.1.S1_at | TTLL1 | tubulin tyrosine ligase-like family, member 1 | 6.52E-09 | 2.41 |
| 492 | Cfa.14321.1.A1_at | BTBD3 | BTB (POZ) domain containing 3 | 0.00149734 | 2.41 |
| 493 | Cfa.20573.1.S1_x_at | CBR4 | carbonyl reductase 4 | 2.23E-10 | 2.41 |
| 494 | CfaAffx.22779.1.S1_s_at | CEP152 | centrosomal protein 152kDa | 5.55E-10 | 2.41 |
| 495 | CfaAffx.17880.1.S1_s_at | --- | --- | 2.82E-10 | 2.40 |
| 496 | CfaAffx.18433.1.S1_at | SPC25 | SPC25, NDC80 kinetochore complex component, homolog (S. cerevisiae) | 1.36E-07 | 2.40 |
| 497 | CfaAffx.24500.1.S1_s_at | FADS1 /// LOC612278 | fatty acid desaturase 1 /// similar to fatty acid desaturase 1 | 5.41E-07 | 2.40 |
| 498 | CfaAffx.29312.1.S1_at | NMRAL1 | NmrA-like family domain containing 1 | 3.50E-11 | 2.40 |
| 499 | Cfa.454.1.A1_at | --- | --- | 2.50E-07 | 2.40 |
| 500 | CfaAffx.29216.1.S1_at | HMGB3 | high-mobility group box 3 | 0.0020165 | 2.40 |
| 501 | Cfa.140.1.S1_s_at | BRCA1 | breast cancer 1, early onset | 2.60E-10 | 2.40 |
| 502 | CfaAffx.24242.1.S1_s_at | PPPDE1 | PPPDE peptidase domain containing 1 | 0.000231324 | 2.40 |
| 503 | CfaAffx.5614.1.S1_s_at | MDN1 | MDN1, midasin homolog (yeast) | 7.38E-07 | 2.40 |
| 504 | CfaAffx.10024.1.S1_s_at | PCNA | proliferating cell nuclear antigen | 3.83E-09 | 2.39 |
| 505 | CfaAffx.11943.1.S1_at | RPP14 | ribonuclease P/MRP 14kDa subunit | 1.12E-11 | 2.39 |
| 506 | Cfa.5293.1.A1_at | --- | --- | 4.55E-05 | 2.39 |
| 507 | Cfa.3066.1.S1_at | NDC80 | NDC80 homolog, kinetochore complex component (S. cerevisiae) | 9.54E-08 | 2.39 |
| 508 | Cfa.20120.1.S1_s_at | WHSC1 | Wolf-Hirschhorn syndrome candidate 1 | 3.36E-10 | 2.39 |
| 509 | Cfa.1832.1.A1_at | TIMM8A | translocase of inner mitochondrial membrane 8 homolog A (yeast) | 4.52E-11 | 2.39 |
| 510 | Cfa.2667.1.S1_s_at | CACNB2 | calcium channel, voltage-dependent, beta 2 subunit | 0.00442213 | 2.38 |
| 511 | Cfa.18708.1.S1_s_at | CCT5 | chaperonin containing TCP1, subunit 5 (epsilon) | 1.18E-12 | 2.38 |
| 512 | CfaAffx.12890.1.S1_at | LOC482649 | similar to CG3558-PA, isoform A | 0.00137609 | 2.38 |
| 513 | CfaAffx.16351.1.S1_s_at | IFIH1 | interferon induced with helicase C domain 1 | 0.000123293 | 2.38 |
| 514 | CfaAffx.12444.1.S1_s_at | MLF1IP | MLF1 interacting protein | 4.57E-06 | 2.38 |
| 515 | Cfa.10744.1.S1_s_at | KIF2C | kinesin family member 2C | 6.80E-08 | 2.38 |
| 516 | Cfa.806.1.A1_at | --- | --- | 0.0144299 | 2.38 |
| 517 | Cfa.19941.1.S1_s_at | MCM4 | minichromosome maintenance complex component 4 | 5.28E-10 | 2.37 |
| 518 | Cfa.6778.1.A1_at | --- | --- | 7.03E-08 | 2.37 |
| 519 | Cfa.17790.1.S1_s_at | MED28 | mediator complex subunit 28 | 1.29E-08 | 2.37 |
| 520 | Cfa.8213.1.A1_at | --- | --- | 7.87E-05 | 2.37 |
| 521 | Cfa.15418.1.A1_s_at | CBR4 | carbonyl reductase 4 | 1.15E-08 | 2.37 |
| 522 | Cfa.18891.1.S1_s_at | SYK | spleen tyrosine kinase | 3.26E-06 | 2.37 |
| 523 | CfaAffx.2129.1.S1_s_at | FBLN1 | fibulin 1 | 0.0208533 | 2.37 |
| 524 | CfaAffx.26925.1.S1_s_at | CENPI | centromere protein I | 2.26E-07 | 2.37 |
| 525 | CfaAffx.18839.1.S1_s_at | SLC30A1 | solute carrier family 30 (zinc transporter), member 1 | 3.00E-08 | 2.37 |
| 526 | Cfa.9412.1.A1_at | IMMT | inner membrane protein, mitochondrial | 1.09E-07 | 2.37 |
| 527 | Cfa.10885.1.A1_at | LOC490715 | hypothetical LOC490715 | 0.000363048 | 2.37 |
| 528 | CfaAffx.23853.1.S1_at | LOC490715 | hypothetical LOC490715 | 8.78E-06 | 2.37 |
| 529 | CfaAffx.1679.1.S1_at | NIPAL2 | NIPA-like domain containing 2 | 0.000215551 | 2.36 |
| 530 | CfaAffx.21146.1.S1_s_at | PIKFYVE | phosphoinositide kinase, FYVE finger containing | 6.17E-07 | 2.36 |
| 531 | CfaAffx.7030.1.S1_at | CENPO | centromere protein O | 5.31E-09 | 2.36 |
| 532 | Cfa.20811.1.S1_s_at | TM9SF2 | transmembrane 9 superfamily member 2 | 0.000563101 | 2.36 |
| 533 | CfaAffx.8884.1.S1_s_at | CLIP4 | CAP-GLY domain containing linker protein family, member 4 | 0.00445629 | 2.36 |
| 534 | Cfa.19949.1.S1_s_at | STRBP | spermatid perinuclear RNA binding protein | 9.52E-07 | 2.36 |
| 535 | Cfa.13288.1.A1_at | GRK4 | G protein-coupled receptor kinase 4 | 0.000232665 | 2.36 |
| 536 | Cfa.4343.1.S1_at | MCM5 | minichromosome maintenance complex component 5 | 1.94E-06 | 2.36 |
| 537 | Cfa.20573.1.S1_at | CBR4 | carbonyl reductase 4 | 1.87E-10 | 2.35 |
| 538 | CfaAffx.15349.1.S1_at | HERC6 | hect domain and RLD 6 | 1.12E-05 | 2.35 |
| 539 | Cfa.21133.1.S1_s_at | HSPA9 | heat shock 70kDa protein 9 (mortalin) | 1.95E-09 | 2.35 |
| 540 | Cfa.12738.1.A1_at | --- | --- | 0.00163598 | 2.35 |
| 541 | CfaAffx.6721.1.S1_at | --- | --- | 1.22E-08 | 2.35 |
| 542 | Cfa.14564.2.S1_at | BLVRB | Biliverdin reductase B (flavin reductase (NADPH)) | 5.62E-05 | 2.34 |
| 543 | Cfa.8727.1.A1_at | LOC610885 | hypothetical protein LOC610885 | 0.000166265 | 2.34 |
| 544 | CfaAffx.26723.1.S1_s_at | EML5 | echinoderm microtubule associated protein like 5 | 1.63E-05 | 2.34 |
| 545 | Cfa.8783.1.S1_s_at | PTTG1 | pituitary tumor-transforming 1 | 4.81E-05 | 2.34 |
| 546 | CfaAffx.3613.1.S1_at | --- | --- | 9.45E-05 | 2.34 |
| 547 | CfaAffx.24001.1.S1_s_at | GNB5 | guanine nucleotide binding protein (G protein), beta 5 | 5.10E-06 | 2.33 |
| 548 | CfaAffx.7248.1.S1_at | LOC607137 | similar to CG9119-PA | 1.26E-07 | 2.33 |
| 549 | CfaAffx.140.1.S1_at | GEMIN4 | gem (nuclear organelle) associated protein 4 | 2.33E-08 | 2.33 |
| 550 | Cfa.17404.1.S1_s_at | ACSL5 | acyl-CoA synthetase long-chain family member 5 | 6.67E-07 | 2.33 |
| 551 | CfaAffx.11852.1.S1_at | IPO7 | importin 7 | 1.11E-09 | 2.33 |
| 552 | Cfa.13076.1.A1_at | --- | --- | 0.000686744 | 2.33 |
| 553 | CfaAffx.6693.1.S1_s_at | LARP1B | La ribonucleoprotein domain family, member 1B | 1.61E-07 | 2.33 |
| 554 | Cfa.8417.1.A1_at | --- | --- | 4.68E-06 | 2.33 |
| 555 | CfaAffx.10985.1.S1_s_at | HSD17B12 | hydroxysteroid (17-beta) dehydrogenase 12 | 1.04E-09 | 2.33 |
| 556 | CfaAffx.26488.1.S1_at | LOC478349 | similar to Zwilch | 1.95E-08 | 2.33 |
| 557 | CfaAffx.25740.1.S1_s_at | KIF4A | kinesin family member 4A | 1.36E-07 | 2.32 |
| 558 | CfaAffx.13934.1.S1_at | RND1 | Rho family GTPase 1 | 4.69E-05 | 2.32 |
| 559 | Cfa.7107.1.A1_at | ZWINT | ZW10 interactor | 7.41E-06 | 2.32 |
| 560 | Cfa.14476.1.S1_at | LOC486352 | similar to mitochondrial protein 18 kDa isoform a | 7.66E-06 | 2.32 |
| 561 | CfaAffx.28602.1.S1_at | ATG4C | ATG4 autophagy related 4 homolog C (S. cerevisiae) | 5.25E-07 | 2.32 |
| 562 | CfaAffx.4065.1.S1_at | LOC481829 | similar to CG11212-PA | 0.0195003 | 2.32 |
| 563 | CfaAffx.25205.1.S1_s_at | MAD2L1 | MAD2 mitotic arrest deficient-like 1 (yeast) | 5.11E-08 | 2.31 |
| 564 | CfaAffx.14116.1.S1_at | OAS2 | 2'-5'-oligoadenylate synthetase 2, 69/71kDa | 0.00721048 | 2.31 |
| 565 | CfaAffx.13599.1.S1_s_at | CTSK | cathepsin K | 8.01E-06 | 2.31 |
| 566 | Cfa.7916.1.A1_at | --- | --- | 5.99E-06 | 2.31 |
| 567 | Cfa.20116.1.S1_s_at | ANLN | anillin, actin binding protein | 0.00013794 | 2.31 |
| 568 | CfaAffx.9471.1.S1_s_at | RNF213 | ring finger protein 213 | 1.24E-05 | 2.31 |
| 569 | Cfa.871.2.S1_s_at | --- | --- | 9.57E-06 | 2.30 |
| 570 | Cfa.20812.1.S1_s_at | LARP1 | La ribonucleoprotein domain family, member 1 | 0.000486644 | 2.30 |
| 571 | CfaAffx.4735.1.S1_s_at | UBE2S | ubiquitin-conjugating enzyme E2S | 1.87E-05 | 2.30 |
| 572 | CfaAffx.28468.1.S1_at | CD70 | CD70 molecule | 0.00722733 | 2.30 |
| 573 | CfaAffx.14972.1.S1_at | LY86 | lymphocyte antigen 86 | 4.72E-07 | 2.30 |
| 574 | Cfa.3042.1.A1_at | --- | --- | 1.72E-06 | 2.30 |
| 575 | Cfa.11769.1.A1_at | LOC475733 | similar to protein containing single MORN motif in testis | 3.92E-05 | 2.29 |
| 576 | CfaAffx.23284.1.S1_s_at | NCAPD2 | non-SMC condensin I complex, subunit D2 | 4.63E-09 | 2.29 |
| 577 | CfaAffx.6968.1.S1_at | PUS7 | pseudouridylate synthase 7 homolog (S. cerevisiae) | 3.20E-09 | 2.29 |
| 578 | CfaAffx.26219.1.S1_s_at | CKS1B | CDC28 protein kinase regulatory subunit 1B | 2.46E-10 | 2.29 |
| 579 | CfaAffx.17556.1.S1_at | KCNMB3 | potassium large conductance calcium-activated channel, subfamily M beta member 3 | 0.000720862 | 2.29 |
| 580 | Cfa.2817.1.A1_at | --- | --- | 0.00282961 | 2.29 |
| 581 | Cfa.18283.1.S1_s_at | IARS | isoleucyl-tRNA synthetase | 1.55E-11 | 2.29 |
| 582 | CfaAffx.14226.1.S1_s_at | DDX60 | DEAD (Asp-Glu-Ala-Asp) box polypeptide 60 | 0.0213862 | 2.29 |
| 583 | CfaAffx.1910.1.S1_at | SLC25A32 | solute carrier family 25, member 32 | 2.07E-07 | 2.28 |
| 584 | Cfa.16407.1.A1_at | --- | --- | 1.19E-05 | 2.28 |
| 585 | CfaAffx.13493.1.S1_s_at | --- | --- | 0.000311166 | 2.28 |
| 586 | Cfa.33.1.S1_s_at | TPMT | thiopurine S-methyltransferase | 7.17E-08 | 2.28 |
| 587 | Cfa.16949.2.S1_at | --- | --- | 5.77E-07 | 2.28 |
| 588 | CfaAffx.15522.1.S1_s_at | BID | BH3 interacting domain death agonist | 2.59E-08 | 2.28 |
| 589 | Cfa.18077.1.S1_s_at | MCM4 | minichromosome maintenance complex component 4 | 7.02E-10 | 2.28 |
| 590 | Cfa.11546.1.A1_at | LOC607087 | hypothetical protein LOC607087 | 6.84E-09 | 2.28 |
| 591 | Cfa.12817.1.A1_at | --- | --- | 0.000295743 | 2.28 |
| 592 | CfaAffx.23845.1.S1_s_at | HAUS8 | HAUS augmin-like complex, subunit 8 | 6.02E-10 | 2.28 |
| 593 | CfaAffx.2413.1.S1_at | SREBF2 | sterol regulatory element binding transcription factor 2 | 2.28E-05 | 2.28 |
| 594 | Cfa.7110.1.A1_s_at | NDUFAF2 | NADH dehydrogenase (ubiquinone) 1 alpha subcomplex, assembly factor 2 | 4.30E-08 | 2.28 |
| 595 | CfaAffx.7212.1.S1_s_at | LOC491373 /// LOC607456 /// MAD2L1 | similar to Mitotic spindle assembly checkpoint protein MAD2A (MAD2-like 1) (HsMA | 7.61E-07 | 2.28 |
| 596 | Cfa.2892.1.A1_at | EIF5A2 | eukaryotic translation initiation factor 5A2 | 1.58E-07 | 2.27 |
| 597 | CfaAffx.9290.1.S1_at | --- | --- | 4.18E-07 | 2.27 |
| 598 | Cfa.19944.1.S1_s_at | HSPA4L | heat shock 70kDa protein 4-like | 2.39E-07 | 2.27 |
| 599 | Cfa.5556.1.A1_s_at | LOC607417 | similar to Thymidylate synthase (TS) (TSase) | 4.58E-10 | 2.27 |
| 600 | CfaAffx.17067.1.S1_at | BPNT1 | 3'(2'), 5'-bisphosphate nucleotidase 1 | 9.75E-10 | 2.27 |
| 601 | Cfa.7047.1.A1_at | --- | --- | 2.06E-07 | 2.27 |
| 602 | CfaAffx.19003.1.S1_s_at | SLMO2 | slowmo homolog 2 (Drosophila) | 7.51E-11 | 2.27 |
| 603 | CfaAffx.28782.1.S1_s_at | MBNL3 | muscleblind-like 3 (Drosophila) | 0.000299111 | 2.26 |
| 604 | CfaAffx.3765.1.S1_s_at | COMMD8 | COMM domain containing 8 | 2.62E-10 | 2.26 |
| 605 | Cfa.15181.1.A1_s_at | DKC1 | dyskeratosis congenita 1, dyskerin | 7.97E-10 | 2.25 |
| 606 | CfaAffx.20026.1.S1_s_at | CDC25A | cell division cycle 25 homolog A (S. pombe) | 2.87E-06 | 2.25 |
| 607 | CfaAffx.809.1.S1_at | MARS2 | methionyl-tRNA synthetase 2, mitochondrial | 4.13E-09 | 2.25 |
| 608 | CfaAffx.859.1.S1_s_at | MIS12 /// OR16B11 | MIS12, MIND kinetochore complex component, homolog (S. pombe) /// olfactory rece | 6.52E-06 | 2.25 |
| 609 | Cfa.17414.1.S1_at | --- | --- | 3.14E-08 | 2.25 |
| 610 | Cfa.13970.1.S1_at | EIF5A2 | eukaryotic translation initiation factor 5A2 | 4.70E-08 | 2.25 |
| 611 | Cfa.9812.1.A1_at | --- | --- | 3.24E-07 | 2.25 |
| 612 | CfaAffx.12827.1.S1_s_at | LOC608882 | similar to basic FGF-repressed Zic binding protein isoform a | 9.89E-09 | 2.25 |
| 613 | Cfa.13028.1.A1_at | --- | --- | 0.000189459 | 2.25 |
| 614 | CfaAffx.20883.1.S1_at | REXO2 | REX2, RNA exonuclease 2 homolog (S. cerevisiae) | 3.49E-08 | 2.25 |
| 615 | CfaAffx.14676.1.S1_at | ESRP1 | epithelial splicing regulatory protein 1 | 0.000540191 | 2.25 |
| 616 | Cfa.1780.1.S1_at | FEN1 | flap structure-specific endonuclease 1 | 6.34E-09 | 2.24 |
| 617 | CfaAffx.12909.1.S1_at | HELLS | helicase, lymphoid-specific | 2.23E-08 | 2.24 |
| 618 | CfaAffx.19596.1.S1_s_at | ZNF593 | zinc finger protein 593 | 4.55E-08 | 2.24 |
| 619 | Cfa.13539.1.A1_at | --- | --- | 6.89E-07 | 2.24 |
| 620 | CfaAffx.26595.1.S1_s_at | NUP210L | nucleoporin 210kDa-like | 7.36E-09 | 2.24 |
| 621 | CfaAffx.1243.1.S1_at | STX7 | syntaxin 7 | 1.24E-09 | 2.24 |
| 622 | Cfa.4558.1.S1_s_at | HMGA1 | high mobility group AT-hook 1 | 5.83E-07 | 2.24 |
| 623 | Cfa.13143.1.A1_at | --- | --- | 1.17E-06 | 2.24 |
| 624 | Cfa.17199.1.S1_at | PDK3 | pyruvate dehydrogenase kinase, isozyme 3 | 3.28E-08 | 2.24 |
| 625 | CfaAffx.27154.1.S1_at | BRIP1 | BRCA1 interacting protein C-terminal helicase 1 | 8.20E-08 | 2.24 |
| 626 | Cfa.17691.1.S1_at | CHORDC1 | cysteine and histidine-rich domain (CHORD)-containing 1 | 4.95E-08 | 2.24 |
| 627 | Cfa.18914.1.S1_s_at | MS4A1 | membrane-spanning 4-domains, subfamily A, member 1 | 0.00395669 | 2.23 |
| 628 | CfaAffx.18042.1.S1_s_at | LOC607237 | similar to CG10581-PA | 5.08E-10 | 2.23 |
| 629 | Cfa.8940.1.A1_s_at | COQ7 | coenzyme Q7 homolog, ubiquinone (yeast) | 2.98E-06 | 2.23 |
| 630 | Cfa.5302.1.A1_at | --- | --- | 0.00685392 | 2.23 |
| 631 | Cfa.9123.1.S1_at | --- | --- | 5.20E-05 | 2.23 |
| 632 | CfaAffx.7811.1.S1_s_at | MMACHC | methylmalonic aciduria (cobalamin deficiency) cblC type, with homocystinuria | 6.32E-08 | 2.23 |
| 633 | CfaAffx.24998.1.S1_at | DOK3 | docking protein 3 | 1.92E-09 | 2.22 |
| 634 | CfaAffx.17119.1.S1_s_at | ACSL5 | acyl-CoA synthetase long-chain family member 5 | 9.08E-09 | 2.22 |
| 635 | CfaAffx.7091.1.S1_x_at | NME1 | non-metastatic cells 1, protein (NM23A) expressed in | 2.01E-06 | 2.22 |
| 636 | CfaAffx.13633.1.S1_at | E2F5 | E2F transcription factor 5, p130-binding | 0.00191123 | 2.22 |
| 637 | CfaAffx.23010.1.S1_s_at | EIF5A2 | eukaryotic translation initiation factor 5A2 | 2.60E-09 | 2.22 |
| 638 | CfaAffx.23720.1.S1_at | ANKLE1 | ankyrin repeat and LEM domain containing 1 | 1.63E-07 | 2.22 |
| 639 | Cfa.10684.2.A1_s_at | PFKP | phosphofructokinase, platelet | 1.62E-08 | 2.22 |
| 640 | CfaAffx.23204.1.S1_at | MED28 | mediator complex subunit 28 | 1.03E-09 | 2.22 |
| 641 | CfaAffx.18595.1.S1_s_at | LOC483236 /// PPIL3 | similar to peptidylprolyl isomerase-like protein 3 isoform PPIL3b /// peptidylpr | 3.78E-06 | 2.22 |
| 642 | CfaAffx.26309.1.S1_at | HMMR | hyaluronan-mediated motility receptor (RHAMM) | 7.45E-05 | 2.22 |
| 643 | CfaAffx.29979.1.S1_at | GNG13 | guanine nucleotide binding protein (G protein), gamma 13 | 8.74E-05 | 2.22 |
| 644 | Cfa.21178.1.S1_s_at | CSE1L | CSE1 chromosome segregation 1-like (yeast) | 1.32E-07 | 2.22 |
| 645 | Cfa.8960.1.A1_at | AHCY | adenosylhomocysteinase | 5.45E-12 | 2.22 |
| 646 | CfaAffx.23062.1.S1_at | WDHD1 | WD repeat and HMG-box DNA binding protein 1 | 7.72E-09 | 2.22 |
| 647 | CfaAffx.25383.1.S1_at | DCTPP1 | dCTP pyrophosphatase 1 | 2.42E-07 | 2.21 |
| 648 | Cfa.6964.1.A1_s_at | PFKL | phosphofructokinase, liver | 0.000549156 | 2.21 |
| 649 | Cfa.20627.1.S1_s_at | RSL1D1 | ribosomal L1 domain containing 1 | 5.77E-09 | 2.21 |
| 650 | CfaAffx.22817.1.S1_at | GNPNAT1 | glucosamine-phosphate N-acetyltransferase 1 | 1.82E-07 | 2.21 |
| 651 | Cfa.21063.1.S1_at | LOC609701 | similar to Protein C21orf45 | 4.07E-05 | 2.21 |
| 652 | Cfa.19911.1.S1_at | ERO1LB | ERO1-like beta (S. cerevisiae) | 3.34E-08 | 2.21 |
| 653 | Cfa.14073.1.A1_at | --- | --- | 3.27E-05 | 2.21 |
| 654 | Cfa.19718.1.S1_at | DPH3 | DPH3, KTI11 homolog (S. cerevisiae) | 1.13E-08 | 2.21 |
| 655 | CfaAffx.28278.1.S1_at | ATAD5 | ATPase family, AAA domain containing 5 | 7.91E-08 | 2.21 |
| 656 | CfaAffx.13588.1.S1_s_at | TDO2 | tryptophan 2,3-dioxygenase | 5.73E-05 | 2.21 |
| 657 | Cfa.20760.1.S1_at | TRIM44 | tripartite motif-containing 44 | 2.74E-08 | 2.20 |
| 658 | CfaAffx.242.1.S1_s_at | LOC475685 | similar to CG14903-PA | 8.14E-08 | 2.20 |
| 659 | Cfa.4696.1.A1_at | --- | --- | 0.0119908 | 2.20 |
| 660 | CfaAffx.23314.1.S1_s_at | LOC611419 | hypothetical protein LOC611419 | 1.99E-10 | 2.20 |
| 661 | Cfa.4721.1.A1_at | --- | --- | 2.99E-05 | 2.20 |
| 662 | CfaAffx.21914.1.S1_s_at | POLE2 | polymerase (DNA directed), epsilon 2 (p59 subunit) | 4.31E-05 | 2.20 |
| 663 | Cfa.21066.1.S1_s_at | CINP | cyclin-dependent kinase 2 interacting protein | 8.33E-09 | 2.20 |
| 664 | CfaAffx.28332.1.S1_s_at | LOC609090 | hypothetical protein LOC609090 | 1.33E-09 | 2.20 |
| 665 | Cfa.5529.1.S1_at | LOC479165 | similar to Dihydrofolate reductase | 0.00215313 | 2.19 |
| 666 | CfaAffx.11755.1.S1_at | --- | --- | 3.56E-05 | 2.19 |
| 667 | CfaAffx.7499.1.S1_at | MASTL | microtubule associated serine/threonine kinase-like | 4.15E-08 | 2.19 |
| 668 | Cfa.1854.1.A1_at | --- | --- | 5.14E-05 | 2.19 |
| 669 | CfaAffx.29976.1.S1_at | NOL9 | nucleolar protein 9 | 1.92E-07 | 2.19 |
| 670 | CfaAffx.7443.1.S1_at | ACAD9 | acyl-CoA dehydrogenase family, member 9 | 7.66E-09 | 2.19 |
| 671 | Cfa.11686.1.A1_at | --- | --- | 3.10E-07 | 2.19 |
| 672 | CfaAffx.17277.1.S1_at | SGOL2 | shugoshin-like 2 (S. pombe) | 1.73E-07 | 2.19 |
| 673 | Cfa.20156.1.S1_s_at | SNX2 | sorting nexin 2 | 3.61E-08 | 2.19 |
| 674 | CfaAffx.21663.1.S1_at | LANCL1 | LanC lantibiotic synthetase component C-like 1 (bacterial) | 3.78E-09 | 2.19 |
| 675 | CfaAffx.24074.1.S1_at | FH | fumarate hydratase | 3.93E-11 | 2.18 |
| 676 | CfaAffx.10457.1.S1_at | NOC4L | nucleolar complex associated 4 homolog (S. cerevisiae) | 1.32E-08 | 2.18 |
| 677 | Cfa.9508.1.A1_at | --- | --- | 3.74E-05 | 2.18 |
| 678 | CfaAffx.24419.1.S1_at | ADIPOR2 | adiponectin receptor 2 | 6.39E-07 | 2.18 |
| 679 | Cfa.1964.1.A1_at | --- | --- | 0.00113386 | 2.18 |
| 680 | CfaAffx.17386.1.S1_s_at | UNG | uracil-DNA glycosylase | 2.58E-10 | 2.18 |
| 681 | CfaAffx.738.1.S1_s_at | GINS1 | GINS complex subunit 1 (Psf1 homolog) | 5.90E-12 | 2.18 |
| 682 | CfaAffx.20485.1.S1_at | --- | --- | 1.52E-06 | 2.18 |
| 683 | CfaAffx.11305.1.S1_at | AACS | acetoacetyl-CoA synthetase | 3.92E-06 | 2.17 |
| 684 | CfaAffx.25150.1.S1_s_at | EIF3B | eukaryotic translation initiation factor 3, subunit B | 1.70E-09 | 2.17 |
| 685 | Cfa.20573.1.S1_s_at | CBR4 | carbonyl reductase 4 | 3.32E-10 | 2.17 |
| 686 | CfaAffx.7190.1.S1_at | SAE1 | SUMO1 activating enzyme subunit 1 | 2.42E-10 | 2.17 |
| 687 | Cfa.20388.1.S1_s_at | RUVBL2 | RuvB-like 2 (E. coli) | 1.21E-09 | 2.17 |
| 688 | CfaAffx.13794.1.S1_s_at | MTHFD2 | methylenetetrahydrofolate dehydrogenase (NADP+ dependent) 2, methenyltetrahydrof | 4.22E-08 | 2.17 |
| 689 | Cfa.18131.1.S1_s_at | HSPH1 | heat shock 105kDa/110kDa protein 1 | 9.03E-05 | 2.17 |
| 690 | CfaAffx.24173.1.S1_s_at | TTC25 | tetratricopeptide repeat domain 25 | 5.54E-05 | 2.17 |
| 691 | Cfa.13270.1.S1_at | CDT1 | chromatin licensing and DNA replication factor 1 | 1.12E-06 | 2.16 |
| 692 | CfaAffx.30291.1.S1_at | LOC489647 | similar to Epithelial-cadherin precursor (E-cadherin) (Uvomorulin) (Cadherin-1) | 0.0107652 | 2.16 |
| 693 | CfaAffx.13758.1.S1_s_at | PRKCD | protein kinase C, delta | 6.20E-09 | 2.16 |
| 694 | Cfa.20214.1.S1_at | LOC607137 | similar to CG9119-PA | 5.18E-07 | 2.16 |
| 695 | CfaAffx.3418.1.S1_at | --- | --- | 0.00180248 | 2.16 |
| 696 | CfaAffx.20621.1.S1_s_at | NCF2 | neutrophil cytosolic factor 2 | 6.53E-06 | 2.16 |
| 697 | Cfa.15410.2.A1_at | NOP56 | NOP56 ribonucleoprotein homolog (yeast) | 9.92E-06 | 2.16 |
| 698 | CfaAffx.24182.1.S1_at | HSH2D | hematopoietic SH2 domain containing | 7.68E-06 | 2.16 |
| 699 | CfaAffx.7811.1.S1_at | MMACHC | methylmalonic aciduria (cobalamin deficiency) cblC type, with homocystinuria | 3.33E-08 | 2.16 |
| 700 | Cfa.11397.1.A1_at | --- | --- | 1.34E-05 | 2.16 |
| 701 | CfaAffx.18054.1.S1_s_at | LOC480469 | similar to Importin alpha-2 subunit (Karyopherin alpha-2 subunit) (SRP1-alpha) ( | 3.73E-08 | 2.16 |
| 702 | Cfa.12790.1.A1_at | --- | --- | 1.05E-06 | 2.16 |
| 703 | CfaAffx.30194.1.S1_at | WDR77 | WD repeat domain 77 | 3.12E-08 | 2.15 |
| 704 | Cfa.13884.1.A1_at | --- | --- | 0.00589248 | 2.15 |
| 705 | Cfa.7110.1.A1_at | NDUFAF2 | NADH dehydrogenase (ubiquinone) 1 alpha subcomplex, assembly factor 2 | 1.38E-08 | 2.15 |
| 706 | CfaAffx.22766.1.S1_at | LOC490959 | similar to WNK lysine deficient protein kinase 4 | 0.00245681 | 2.15 |
| 707 | CfaAffx.1580.1.S1_s_at | MTHFD1L | methylenetetrahydrofolate dehydrogenase (NADP+ dependent) 1-like | 1.34E-06 | 2.15 |
| 708 | Cfa.85.1.S1_s_at | RAD51 | RAD51 homolog (RecA homolog, E. coli) (S. cerevisiae) | 3.88E-06 | 2.15 |
| 709 | Cfa.3786.1.S1_s_at | MYC | v-myc myelocytomatosis viral oncogene homolog (avian) | 5.79E-05 | 2.15 |
| 710 | CfaAffx.1736.1.S1_at | RGS22 | regulator of G-protein signaling 22 | 0.00160679 | 2.15 |
| 711 | Cfa.13388.1.S1_at | SLC44A1 | solute carrier family 44, member 1 | 4.98E-06 | 2.14 |
| 712 | Cfa.18695.1.S1_s_at | RPA1 | replication protein A1, 70kDa | 1.05E-07 | 2.14 |
| 713 | Cfa.301.1.A1_at | LOC475096 /// NSMCE2 | hypothetical LOC475096 /// non-SMC element 2, MMS21 homolog (S. cerevisiae) | 0.000890257 | 2.14 |
| 714 | CfaAffx.21535.1.S1_at | KIF18B | kinesin family member 18B | 6.36E-08 | 2.14 |
| 715 | Cfa.1474.1.A1_s_at | GTF3A | general transcription factor IIIA | 1.36E-10 | 2.14 |
| 716 | Cfa.3983.1.A1_s_at | IGSF10 | immunoglobulin superfamily, member 10 | 0.00168179 | 2.14 |
| 717 | CfaAffx.29976.1.S1_s_at | NOL9 | nucleolar protein 9 | 3.60E-08 | 2.14 |
| 718 | Cfa.10593.1.A1_at | SYNCRIP | synaptotagmin binding, cytoplasmic RNA interacting protein | 5.41E-07 | 2.14 |
| 719 | CfaAffx.24044.1.S1_at | PPIF | peptidylprolyl isomerase F | 1.23E-05 | 2.14 |
| 720 | CfaAffx.16501.1.S1_s_at | CBS | cystathionine-beta-synthase | 4.32E-05 | 2.14 |
| 721 | CfaAffx.13881.1.S1_at | ERP29 | endoplasmic reticulum protein 29 | 1.18E-10 | 2.14 |
| 722 | CfaAffx.3408.1.S1_at | --- | --- | 7.24E-06 | 2.14 |
| 723 | Cfa.14564.2.S1_x_at | BLVRB | biliverdin reductase B (flavin reductase (NADPH)) | 0.000151143 | 2.13 |
| 724 | Cfa.4878.1.A1_at | --- | --- | 0.000177138 | 2.13 |
| 725 | CfaAffx.28317.1.S1_at | TNFRSF13B | tumor necrosis factor receptor superfamily, member 13B | 6.80E-05 | 2.13 |
| 726 | Cfa.10400.1.A1_at | SNHG3-RCC1 | SNHG3-RCC1 readthrough transcript | 1.03E-07 | 2.13 |
| 727 | CfaAffx.3728.1.S1_s_at | POLR1C | polymerase (RNA) I polypeptide C, 30kDa | 7.16E-11 | 2.13 |
| 728 | CfaAffx.19718.1.S1_s_at | LOC486352 | similar to mitochondrial protein 18 kDa isoform a | 2.12E-06 | 2.13 |
| 729 | CfaAffx.555.1.S1_s_at | --- | --- | 2.06E-07 | 2.13 |
| 730 | CfaAffx.19429.1.S1_s_at | WDR12 | WD repeat domain 12 | 2.33E-11 | 2.13 |
| 731 | CfaAffx.20621.1.S1_at | NCF2 | neutrophil cytosolic factor 2 | 8.63E-06 | 2.13 |
| 732 | Cfa.19483.1.S1_s_at | SF3B3 | splicing factor 3b, subunit 3, 130kDa | 2.72E-07 | 2.13 |
| 733 | Cfa.17790.1.S1_at | MED28 | mediator complex subunit 28 | 3.41E-09 | 2.13 |
| 734 | CfaAffx.28830.1.S1_at | LOC492149 | similar to CG4497-PA | 4.76E-06 | 2.13 |
| 735 | CfaAffx.27660.1.S1_at | DSG2 | desmoglein 2 | 7.76E-07 | 2.12 |
| 736 | CfaAffx.15384.1.S1_s_at | NCAPD3 | non-SMC condensin II complex, subunit D3 | 4.43E-08 | 2.12 |
| 737 | CfaAffx.18599.1.S1_at | PARP14 | poly (ADP-ribose) polymerase family, member 14 | 0.000173808 | 2.12 |
| 738 | CfaAffx.16037.1.S1_at | SFXN2 | sideroflexin 2 | 8.45E-07 | 2.12 |
| 739 | Cfa.15516.1.A1_at | TMEM93 | transmembrane protein 93 | 1.31E-08 | 2.12 |
| 740 | Cfa.16355.1.S1_s_at | AURKB | aurora kinase B | 1.39E-06 | 2.12 |
| 741 | Cfa.18911.1.S1_s_at | SLC20A1 | solute carrier family 20 (phosphate transporter), member 1 | 3.09E-08 | 2.12 |
| 742 | CfaAffx.16226.1.S1_at | MS4A7 | membrane-spanning 4-domains, subfamily A, member 7 | 0.00957243 | 2.12 |
| 743 | CfaAffx.8359.1.S1_at | XRCC2 | X-ray repair complementing defective repair in Chinese hamster cells 2 | 2.09E-08 | 2.12 |
| 744 | CfaAffx.31271.1.S1_at | LRRC40 | leucine rich repeat containing 40 | 8.53E-09 | 2.12 |
| 745 | CfaAffx.23073.1.S1_at | --- | --- | 9.20E-05 | 2.12 |
| 746 | CfaAffx.28267.1.S1_s_at | LOC480615 | hypothetical LOC480615 | 6.41E-11 | 2.12 |
| 747 | CfaAffx.825.1.S1_at | --- | --- | 3.45E-05 | 2.12 |
| 748 | Cfa.11699.1.A1_at | ACOT7 | acyl-CoA thioesterase 7 | 0.00323376 | 2.12 |
| 749 | Cfa.11565.1.A1_at | RASAL2 | RAS protein activator like 2 | 3.84E-05 | 2.12 |
| 750 | CfaAffx.10864.1.S1_at | MCM4 | minichromosome maintenance complex component 4 | 3.93E-09 | 2.12 |
| 751 | Cfa.17913.1.S1_s_at | USP14 | ubiquitin specific peptidase 14 (tRNA-guanine transglycosylase) | 6.72E-11 | 2.12 |
| 752 | CfaAffx.23130.1.S1_at | NOP2 | NOP2 nucleolar protein homolog (yeast) | 8.78E-07 | 2.11 |
| 753 | CfaAffx.13791.1.S1_at | MTHFD2 | methylenetetrahydrofolate dehydrogenase (NADP+ dependent) 2, methenyltetrahydrof | 3.69E-11 | 2.11 |
| 754 | CfaAffx.30683.1.S1_at | WDR34 | WD repeat domain 34 | 1.39E-06 | 2.11 |
| 755 | CfaAffx.7382.1.S1_s_at | POLR2D | polymerase (RNA) II (DNA directed) polypeptide D | 6.82E-07 | 2.11 |
| 756 | CfaAffx.26418.1.S1_s_at | LOC480136 | similar to Protein C1orf43 (Hepatitis C virus NS5A-transactivated protein 4) (NI | 3.28E-12 | 2.11 |
| 757 | CfaAffx.30647.1.S1_at | SASS6 | spindle assembly 6 homolog (C. elegans) | 2.57E-10 | 2.11 |
| 758 | Cfa.18175.1.S1_at | GINS1 | GINS complex subunit 1 (Psf1 homolog) | 2.57E-13 | 2.11 |
| 759 | CfaAffx.30492.1.S1_at | GINS2 | GINS complex subunit 2 (Psf2 homolog) | 2.18E-08 | 2.11 |
| 760 | CfaAffx.17999.1.S1_s_at | PCNXL2 | pecanex-like 2 (Drosophila) | 0.00356015 | 2.11 |
| 761 | CfaAffx.14162.1.S1_at | ENOPH1 | enolase-phosphatase 1 | 2.48E-09 | 2.11 |
| 762 | CfaAffx.15979.1.S1_at | E2F3 | E2F transcription factor 3 | 4.94E-10 | 2.10 |
| 763 | CfaAffx.15627.1.S1_s_at | LOC477695 /// LOC488369 | similar to mago-nashi homolog /// similar to mago-nashi homolog | 7.66E-09 | 2.10 |
| 764 | CfaAffx.5524.1.S1_s_at | MTHFD2L | methylenetetrahydrofolate dehydrogenase (NADP+ dependent) 2-like | 3.63E-07 | 2.10 |
| 765 | Cfa.17148.1.S1_s_at | RFC4 | replication factor C (activator 1) 4, 37kDa | 4.85E-11 | 2.10 |
| 766 | Cfa.1670.2.S1_a_at | COMMD8 | COMM domain containing 8 | 1.23E-09 | 2.10 |
| 767 | Cfa.8028.1.S1_x_at | SNRPF | small nuclear ribonucleoprotein polypeptide F | 3.42E-07 | 2.10 |
| 768 | Cfa.9833.1.S1_at | LOC611683 | Similar to Protein C14orf112 precursor | 9.06E-10 | 2.10 |
| 769 | Cfa.16850.1.S1_at | LOC483799 | similar to cytochrome b, ascorbate dependent 3 protein | 0.000114371 | 2.10 |
| 770 | Cfa.18531.1.S1_s_at | IPO5 | importin 5 | 4.19E-07 | 2.10 |
| 771 | CfaAffx.14021.1.S1_at | LOC609701 | similar to Protein C21orf45 | 7.19E-07 | 2.10 |
| 772 | CfaAffx.10117.1.S1_s_at | CARS2 | cysteinyl-tRNA synthetase 2, mitochondrial (putative) | 5.04E-09 | 2.10 |
| 773 | CfaAffx.6738.1.S1_s_at | CCT2 | chaperonin containing TCP1, subunit 2 (beta) | 1.33E-10 | 2.10 |
| 774 | CfaAffx.7092.1.S1_s_at | RUVBL1 | RuvB-like 1 (E. coli) | 4.03E-07 | 2.10 |
| 775 | CfaAffx.30405.1.S1_s_at | CDT1 | chromatin licensing and DNA replication factor 1 | 4.50E-07 | 2.10 |
| 776 | Cfa.18187.1.S1_at | LOC610565 | hypothetical protein LOC610565 | 4.44E-08 | 2.09 |
| 777 | CfaAffx.915.1.S1_s_at | HIGD1A | HIG1 hypoxia inducible domain family, member 1A | 9.64E-06 | 2.09 |
| 778 | Cfa.19148.1.S1_s_at | RRM1 | ribonucleotide reductase M1 | 2.64E-07 | 2.09 |
| 779 | CfaAffx.9810.1.S1_at | CKAP2 | cytoskeleton associated protein 2 | 7.86E-08 | 2.09 |
| 780 | Cfa.21020.1.S1_s_at | DNAJC2 | DnaJ (Hsp40) homolog, subfamily C, member 2 | 6.55E-09 | 2.09 |
| 781 | CfaAffx.9926.1.S1_at | LOC611593 | hypothetical protein LOC611593 | 8.22E-09 | 2.09 |
| 782 | CfaAffx.12012.1.S1_at | GTF2H3 | general transcription factor IIH, polypeptide 3, 34kDa | 3.26E-10 | 2.09 |
| 783 | Cfa.14990.2.A1_at | UBFD1 | ubiquitin family domain containing 1 | 2.99E-06 | 2.09 |
| 784 | CfaAffx.19734.1.S1_at | LHPP | phospholysine phosphohistidine inorganic pyrophosphate phosphatase | 5.23E-07 | 2.09 |
| 785 | CfaAffx.23951.1.S1_s_at | PMS2 | PMS2 postmeiotic segregation increased 2 (S. cerevisiae) | 3.74E-07 | 2.09 |
| 786 | CfaAffx.8672.1.S1_s_at | MCM6 | minichromosome maintenance complex component 6 | 1.13E-08 | 2.09 |
| 787 | Cfa.12070.1.A1_at | --- | --- | 1.44E-05 | 2.09 |
| 788 | CfaAffx.29929.1.S1_s_at | ACOT7 | acyl-CoA thioesterase 7 | 0.00161312 | 2.09 |
| 789 | CfaAffx.28518.1.S1_at | TMEM199 | transmembrane protein 199 | 9.71E-07 | 2.09 |
| 790 | Cfa.10201.2.S1_a_at | RPL22 | ribosomal protein L22 | 4.87E-08 | 2.09 |
| 791 | Cfa.24.1.S1_at | --- | --- | 5.47E-10 | 2.09 |
| 792 | CfaAffx.7251.1.S1_s_at | LOC607137 | similar to CG9119-PA | 9.35E-08 | 2.09 |
| 793 | CfaAffx.20407.1.S1_s_at | OLA1 | Obg-like ATPase 1 | 1.55E-10 | 2.09 |
| 794 | CfaAffx.2487.1.S1_s_at | LOC475096 /// NSMCE2 | hypothetical LOC475096 /// non-SMC element 2, MMS21 homolog (S. cerevisiae) | 0.000272186 | 2.09 |
| 795 | Cfa.18212.1.S1_s_at | DDX47 | DEAD (Asp-Glu-Ala-Asp) box polypeptide 47 | 1.96E-08 | 2.08 |
| 796 | Cfa.6825.1.A1_at | ENOPH1 | enolase-phosphatase 1 | 2.44E-09 | 2.08 |
| 797 | Cfa.7465.1.A1_at | SNHG3-RCC1 | SNHG3-RCC1 readthrough transcript | 6.02E-10 | 2.08 |
| 798 | Cfa.13509.1.A1_at | --- | --- | 0.000115575 | 2.08 |
| 799 | CfaAffx.16115.1.S1_at | LOC477808 | hypothetical LOC477808 | 2.83E-08 | 2.08 |
| 800 | CfaAffx.5487.1.S1_s_at | BUB3 | budding uninhibited by benzimidazoles 3 homolog (yeast) | 2.01E-10 | 2.08 |
| 801 | CfaAffx.8366.1.S1_s_at | MYD88 | myeloid differentiation primary response gene (88) | 7.99E-08 | 2.08 |
| 802 | Cfa.9573.2.A1_a_at | RPL35 | ribosomal protein L35 | 2.22E-06 | 2.08 |
| 803 | Cfa.13257.1.A1_at | FADS1 /// LOC612278 | fatty acid desaturase 1 /// similar to fatty acid desaturase 1 | 3.90E-07 | 2.08 |
| 804 | CfaAffx.19917.1.S1_at | LOC611388 /// UROS | similar to Uroporphyrinogen-III synthase (UROS) (Uroporphyrinogen-III cosyntheta | 5.82E-06 | 2.08 |
| 805 | CfaAffx.4198.1.S1_at | AASDH | aminoadipate-semialdehyde dehydrogenase | 3.49E-07 | 2.08 |
| 806 | CfaAffx.13793.1.S1_s_at | RBL1 | retinoblastoma-like 1 (p107) | 1.63E-07 | 2.08 |
| 807 | Cfa.18563.1.S1_at | --- | --- | 5.22E-07 | 2.08 |
| 808 | Cfa.10201.2.S1_at | RPL22 | ribosomal protein L22 | 1.72E-06 | 2.08 |
| 809 | Cfa.20904.1.S1_s_at | ACAT2 | acetyl-CoA acetyltransferase 2 | 1.76E-08 | 2.07 |
| 810 | CfaAffx.8836.1.S1_s_at | WDR43 | WD repeat domain 43 | 1.13E-10 | 2.07 |
| 811 | Cfa.14605.1.A1_at | --- | --- | 1.29E-08 | 2.07 |
| 812 | CfaAffx.12272.1.S1_s_at | CCNE1 | cyclin E1 | 1.41E-05 | 2.07 |
| 813 | CfaAffx.9059.1.S1_at | THAP1 | THAP domain containing, apoptosis associated protein 1 | 1.63E-05 | 2.07 |
| 814 | CfaAffx.600.1.S1_at | STYX | serine/threonine/tyrosine interacting protein | 1.02E-07 | 2.07 |
| 815 | Cfa.18367.2.S1_s_at | TMPO | thymopoietin | 7.05E-08 | 2.07 |
| 816 | CfaAffx.8007.1.S1_s_at | MKI67IP | MKI67 (FHA domain) interacting nucleolar phosphoprotein | 3.10E-08 | 2.07 |
| 817 | CfaAffx.29948.1.S1_at | UBL4A | ubiquitin-like 4A | 1.24E-08 | 2.07 |
| 818 | Cfa.7553.1.A1_at | LOC477720 | similar to dual-specificity tyrosine-(Y)-phosphorylation regulated kinase 4 | 0.00711074 | 2.07 |
| 819 | Cfa.18187.1.S1_s_at | LOC610565 | hypothetical protein LOC610565 | 2.62E-07 | 2.07 |
| 820 | Cfa.1030.1.S1_at | ADIPOR2 | adiponectin receptor 2 | 7.86E-06 | 2.07 |
| 821 | Cfa.2613.1.S1_at | --- | --- | 5.04E-10 | 2.06 |
| 822 | Cfa.18010.1.S1_s_at | CHEK2 | CHK2 checkpoint homolog (S. pombe) | 1.26E-06 | 2.06 |
| 823 | Cfa.1086.2.A1_a_at | GMNN | geminin, DNA replication inhibitor | 1.09E-06 | 2.06 |
| 824 | CfaAffx.8435.1.S1_at | EXOSC5 | exosome component 5 | 3.25E-08 | 2.06 |
| 825 | CfaAffx.5686.1.S1_s_at | NT5C3 | 5'-nucleotidase, cytosolic III | 7.52E-06 | 2.06 |
| 826 | Cfa.17304.1.S1_s_at | --- | --- | 1.93E-06 | 2.06 |
| 827 | Cfa.19888.1.A1_at | CEP72 | centrosomal protein 72kDa | 2.19E-06 | 2.06 |
| 828 | CfaAffx.22493.1.S1_s_at | SERPINI1 | serpin peptidase inhibitor, clade I (neuroserpin), member 1 | 7.07E-06 | 2.06 |
| 829 | CfaAffx.28600.1.S1_at | SKP2 | S-phase kinase-associated protein 2 (p45) | 9.49E-06 | 2.06 |
| 830 | CfaAffx.20005.1.S1_at | LOC488399 | similar to methionine aminopeptidase 1D | 1.08E-05 | 2.06 |
| 831 | Cfa.18370.1.S1_s_at | BDH1 | 3-hydroxybutyrate dehydrogenase, type 1 | 0.00016594 | 2.06 |
| 832 | Cfa.7313.1.A1_at | PBX3 | pre-B-cell leukemia homeobox 3 | 1.83E-07 | 2.06 |
| 833 | Cfa.7693.1.A1_at | --- | --- | 6.29E-06 | 2.06 |
| 834 | CfaAffx.6801.1.S1_at | HSPA4L | heat shock 70kDa protein 4-like | 1.48E-05 | 2.06 |
| 835 | CfaAffx.30854.1.S1_at | LOC490155 | similar to C53D5.1a | 0.000520516 | 2.06 |
| 836 | Cfa.4244.1.S1_at | LOC609990 | similar to Cytochrome c oxidase polypeptide VIIc, mitochondrial precursor | 2.16E-08 | 2.06 |
| 837 | CfaAffx.19613.1.S1_at | IRGM | immunity-related GTPase family, M | 0.000480929 | 2.05 |
| 838 | CfaAffx.9018.1.S1_s_at | PITRM1 | pitrilysin metallopeptidase 1 | 6.36E-06 | 2.05 |
| 839 | Cfa.5737.1.A1_at | --- | --- | 1.49E-07 | 2.05 |
| 840 | Cfa.1585.2.S1_s_at | NAA15 | N(alpha)-acetyltransferase 15, NatA auxiliary subunit | 1.58E-09 | 2.05 |
| 841 | CfaAffx.9503.1.S1_at | NAP1L1 | nucleosome assembly protein 1-like 1 | 1.00E-07 | 2.05 |
| 842 | Cfa.21042.1.S1_s_at | MAK16 | MAK16 homolog (S. cerevisiae) | 7.76E-09 | 2.05 |
| 843 | Cfa.11557.1.A1_at | GRHPR | glyoxylate reductase/hydroxypyruvate reductase | 4.61E-06 | 2.05 |
| 844 | Cfa.11620.1.A1_s_at | CKAP2 | cytoskeleton associated protein 2 | 1.44E-08 | 2.05 |
| 845 | CfaAffx.20764.1.S1_at | WDR76 | WD repeat domain 76 | 4.60E-06 | 2.05 |
| 846 | Cfa.21053.1.S1_s_at | OXCT1 | 3-oxoacid CoA transferase 1 | 2.82E-06 | 2.05 |
| 847 | Cfa.12159.1.A1_at | PRELID1 | PRELI domain containing 1 | 4.29E-12 | 2.05 |
| 848 | Cfa.11475.1.S1_s_at | --- | --- | 0.00241576 | 2.05 |
| 849 | CfaAffx.24083.1.S1_s_at | DHX58 | DEXH (Asp-Glu-X-His) box polypeptide 58 | 0.000295992 | 2.04 |
| 850 | Cfa.10357.1.A1_at | --- | --- | 1.01E-08 | 2.04 |
| 851 | CfaAffx.7350.1.S1_s_at | CHORDC1 | cysteine and histidine-rich domain (CHORD)-containing 1 | 3.22E-07 | 2.04 |
| 852 | CfaAffx.4435.1.S1_s_at | TOMM5 | translocase of outer mitochondrial membrane 5 homolog (yeast) | 4.53E-09 | 2.04 |
| 853 | Cfa.19539.1.S1_s_at | LOC474524 | similar to CG9987-PA | 9.10E-10 | 2.04 |
| 854 | CfaAffx.8836.1.S1_at | WDR43 | WD repeat domain 43 | 1.46E-09 | 2.04 |
| 855 | CfaAffx.2390.1.S1_at | ATAD2 | ATPase family, AAA domain containing 2 | 4.42E-08 | 2.04 |
| 856 | Cfa.11696.1.A1_s_at | FH | fumarate hydratase | 2.84E-10 | 2.04 |
| 857 | Cfa.4893.1.A1_at | --- | --- | 1.21E-05 | 2.04 |
| 858 | CfaAffx.4345.1.S1_s_at | MRPS10 | mitochondrial ribosomal protein S10 | 8.45E-07 | 2.04 |
| 859 | Cfa.4582.1.A1_at | --- | --- | 0.0198851 | 2.04 |
| 860 | Cfa.5379.1.A1_at | DDX21 | DEAD (Asp-Glu-Ala-Asp) box polypeptide 21 | 2.86E-07 | 2.04 |
| 861 | Cfa.16830.1.S1_at | OXCT1 | 3-oxoacid CoA transferase 1 | 5.90E-06 | 2.04 |
| 862 | Cfa.12075.1.A1_at | LOC480882 | similar to Brain protein 44-like protein | 0.00508315 | 2.04 |
| 863 | CfaAffx.7190.1.S1_s_at | SAE1 | SUMO1 activating enzyme subunit 1 | 7.86E-10 | 2.04 |
| 864 | CfaAffx.5541.1.S1_at | MTHFD2L | methylenetetrahydrofolate dehydrogenase (NADP+ dependent) 2-like | 1.25E-06 | 2.04 |
| 865 | CfaAffx.19230.1.S1_s_at | --- | --- | 3.00E-08 | 2.04 |
| 866 | Cfa.19642.1.S1_s_at | DHX15 | DEAH (Asp-Glu-Ala-His) box polypeptide 15 | 2.64E-10 | 2.04 |
| 867 | CfaAffx.27040.1.S1_at | UBFD1 | ubiquitin family domain containing 1 | 4.29E-07 | 2.04 |
| 868 | Cfa.239.1.S1_at | LOC487697 | similar to EURL protein homolog | 0.000130133 | 2.04 |
| 869 | CfaAffx.21869.1.S1_at | RANBP1 | RAN binding protein 1 | 4.43E-08 | 2.04 |
| 870 | CfaAffx.26316.1.S1_s_at | HMMR | hyaluronan-mediated motility receptor (RHAMM) | 8.17E-05 | 2.03 |
| 871 | CfaAffx.12172.1.S1_s_at | LOC477353 /// LOC491592 /// LOC607770 | similar to ankyrin repeat domain 26 /// similar to ankyrin repeat domain 26 /// | 0.0010447 | 2.03 |
| 872 | CfaAffx.28810.1.S1_s_at | HPRT1 | hypoxanthine phosphoribosyltransferase 1 (Lesch-Nyhan syndrome) | 4.41E-10 | 2.03 |
| 873 | Cfa.10662.1.S1_at | NCAPG | non-SMC condensin I complex, subunit G | 2.60E-09 | 2.03 |
| 874 | Cfa.19013.1.S1_s_at | --- | --- | 6.55E-08 | 2.03 |
| 875 | CfaAffx.11699.1.S1_s_at | KIF20B | kinesin family member 20B | 2.97E-07 | 2.03 |
| 876 | Cfa.1634.2.A1_s_at | RAB3IP | RAB3A interacting protein (rabin3) | 1.19E-05 | 2.03 |
| 877 | Cfa.21016.1.S1_s_at | LOC607614 | hypothetical protein LOC607614 | 7.44E-09 | 2.03 |
| 878 | CfaAffx.19735.1.S1_s_at | --- | --- | 1.85E-08 | 2.03 |
| 879 | CfaAffx.18224.1.S1_at | ASPHD2 | aspartate beta-hydroxylase domain containing 2 | 1.94E-05 | 2.03 |
| 880 | CfaAffx.17151.1.S1_s_at | MRPS35 | mitochondrial ribosomal protein S35 | 6.44E-09 | 2.03 |
| 881 | Cfa.18811.1.S1_s_at | DSE | dermatan sulfate epimerase | 0.000161872 | 2.03 |
| 882 | CfaAffx.6803.1.S1_at | AMD1 | adenosylmethionine decarboxylase 1 | 1.27E-07 | 2.03 |
| 883 | CfaAffx.7003.1.S1_at | NT5DC1 | 5'-nucleotidase domain containing 1 | 9.54E-09 | 2.03 |
| 884 | CfaAffx.8399.1.S1_at | TSEN2 | tRNA splicing endonuclease 2 homolog (S. cerevisiae) | 1.01E-06 | 2.03 |
| 885 | CfaAffx.27763.1.S1_at | TAF4B | TAF4b RNA polymerase II, TATA box binding protein (TBP)-associated factor, 105kD | 9.15E-06 | 2.03 |
| 886 | CfaAffx.805.1.S1_s_at | UBE2E3 | ubiquitin-conjugating enzyme E2E 3 (UBC4/5 homolog, yeast) | 0.000220476 | 2.03 |
| 887 | CfaAffx.31060.1.S1_at | TTLL11 | tubulin tyrosine ligase-like family, member 11 | 5.40E-06 | 2.03 |
| 888 | CfaAffx.25175.1.S1_at | PRELID1 | PRELI domain containing 1 | 1.09E-10 | 2.03 |
| 889 | Cfa.16935.1.S1_at | BNIP1 | BCL2/adenovirus E1B 19kDa interacting protein 1 | 1.15E-07 | 2.02 |
| 890 | Cfa.4547.1.A1_at | FASLG | Fas ligand (TNF superfamily, member 6) | 0.00200109 | 2.02 |
| 891 | Cfa.8295.1.S1_at | RPP14 | ribonuclease P/MRP 14kDa subunit | 1.91E-07 | 2.02 |
| 892 | CfaAffx.4190.1.S1_at | MCM3 | minichromosome maintenance complex component 3 | 2.82E-08 | 2.02 |
| 893 | Cfa.3318.1.S1_s_at | MSH2 | mutS homolog 2, colon cancer, nonpolyposis type 1 (E. coli) | 5.49E-08 | 2.02 |
| 894 | Cfa.20109.1.S1_s_at | LOC476337 | similar to D19Bwg1357e protein | 1.35E-09 | 2.02 |
| 895 | CfaAffx.21694.1.S1_s_at | RASAL2 | RAS protein activator like 2 | 1.07E-06 | 2.02 |
| 896 | Cfa.2035.1.S1_at | --- | --- | 0.00101246 | 2.02 |
| 897 | Cfa.14312.1.A1_at | --- | --- | 0.00342305 | 2.02 |
| 898 | CfaAffx.30288.1.S1_s_at | UBE4B | ubiquitination factor E4B (UFD2 homolog, yeast) | 1.46E-11 | 2.02 |
| 899 | CfaAffx.13272.1.S1_s_at | ALDH18A1 | aldehyde dehydrogenase 18 family, member A1 | 9.43E-09 | 2.02 |
| 900 | CfaAffx.1090.1.S1_at | MMP19 | matrix metallopeptidase 19 | 1.43E-05 | 2.02 |
| 901 | CfaAffx.16952.1.S1_at | ABCA5 | ATP-binding cassette, sub-family A (ABC1), member 5 | 0.00122068 | 2.02 |
| 902 | CfaAffx.17729.1.S1_at | CMKLR1 | chemokine-like receptor 1 | 4.19E-06 | 2.01 |
| 903 | CfaAffx.13019.1.S1_s_at | PLSCR1 | phospholipid scramblase 1 | 0.000317446 | 2.01 |
| 904 | CfaAffx.9765.1.S1_at | LOC607948 | similar to THO complex subunit 4 (Tho4) (Ally of AML-1 and LEF-1) (Transcription | 4.95E-11 | 2.01 |
| 905 | CfaAffx.22774.1.S1_at | CEP152 | centrosomal protein 152kDa | 2.86E-06 | 2.01 |
| 906 | CfaAffx.3845.1.S1_s_at | TEC | tec protein tyrosine kinase | 2.78E-08 | 2.01 |
| 907 | Cfa.8997.1.A1_at | MMACHC | methylmalonic aciduria (cobalamin deficiency) cblC type, with homocystinuria | 1.64E-06 | 2.01 |
| 908 | Cfa.19800.1.S1_s_at | ABCE1 | ATP-binding cassette, sub-family E (OABP), member 1 | 1.39E-09 | 2.01 |
| 909 | Cfa.12632.1.A1_at | --- | --- | 1.98E-05 | 2.01 |
| 910 | CfaAffx.2403.1.S1_s_at | WDYHV1 | WDYHV motif containing 1 | 9.46E-06 | 2.01 |
| 911 | CfaAffx.11115.1.S1_at | GTF3A | general transcription factor IIIA | 2.21E-09 | 2.01 |
| 912 | Cfa.13171.1.A1_at | --- | --- | 0.00181805 | 2.01 |
| 913 | Cfa.14947.1.A1_at | LOC475685 | similar to CG14903-PA | 2.68E-07 | 2.01 |
| 914 | Cfa.21126.1.S1_s_at | EPRS | glutamyl-prolyl-tRNA synthetase | 8.53E-08 | 2.01 |
| 915 | CfaAffx.2198.1.S1_at | CCR6 | chemokine (C-C motif) receptor 6 | 0.000604256 | 2.01 |
| 916 | Cfa.17885.1.S1_s_at | DNM1L | dynamin 1-like | 5.48E-08 | 2.01 |
| 917 | Cfa.2715.1.S1_s_at | PUS7L | pseudouridylate synthase 7 homolog (S. cerevisiae)-like | 3.50E-09 | 2.00 |
| 918 | Cfa.8187.1.A1_at | --- | --- | 5.75E-07 | 2.00 |
| 919 | Cfa.18955.1.S1_at | RNF213 | ring finger protein 213 | 6.55E-05 | 2.00 |
| 920 | CfaAffx.26450.1.S1_s_at | UBLCP1 | ubiquitin-like domain containing CTD phosphatase 1 | 2.04E-08 | 2.00 |
| 921 | Cfa.9528.1.A1_at | --- | --- | 2.04E-06 | 2.00 |
| 922 | CfaAffx.28722.1.S1_at | RAD1 | RAD1 homolog (S. pombe) | 1.93E-06 | 2.00 |
| 923 | CfaAffx.1546.1.S1_at | NUP43 | nucleoporin 43kDa | 3.16E-09 | 2.00 |
| 924 | Cfa.867.1.A1_at | --- | --- | 0.000414586 | 2.00 |
| 925 | Cfa.1336.1.S1_s_at | PRMT1 | protein arginine methyltransferase 1 | 2.40E-13 | 2.00 |
| 926 | Cfa.580.1.A1_at | TMX3 | thioredoxin-related transmembrane protein 3 | 8.01E-08 | 2.00 |
| 927 | Cfa.12446.1.A1_at | --- | --- | 3.51E-10 | -2.00 |
| 928 | Cfa.7763.1.S1_at | LYRM1 | LYR motif containing 1 | 5.68E-10 | -2.00 |
| 929 | CfaAffx.13735.1.S1_at | SSBP2 | single-stranded DNA binding protein 2 | 6.88E-05 | -2.00 |
| 930 | CfaAffx.1905.1.S1_s_at | --- | --- | 0.00233917 | -2.00 |
| 931 | Cfa.19971.1.S1_at | --- | --- | 2.01E-05 | -2.00 |
| 932 | CfaAffx.9677.1.S1_s_at | PLCB4 | phospholipase C, beta 4 | 1.17E-09 | -2.00 |
| 933 | Cfa.20314.1.S1_s_at | ARHGAP29 | Rho GTPase activating protein 29 | 0.000209317 | -2.00 |
| 934 | CfaAffx.4073.1.S1_s_at | FHL2 | four and a half LIM domains 2 | 0.0167565 | -2.00 |
| 935 | Cfa.14236.1.A1_at | --- | --- | 0.00307954 | -2.01 |
| 936 | Cfa.4306.1.S1_s_at | S100A6 | S100 calcium binding protein A6 | 4.14E-05 | -2.01 |
| 937 | CfaAffx.12560.1.S1_s_at | SERPING1 | serpin peptidase inhibitor, clade G (C1 inhibitor), member 1 | 0.00358773 | -2.01 |
| 938 | Cfa.19257.1.A1_at | --- | --- | 0.000358381 | -2.01 |
| 939 | Cfa.4759.1.A1_at | --- | --- | 0.000337712 | -2.01 |
| 940 | CfaAffx.500.1.S1_x_at | --- | --- | 2.61E-07 | -2.01 |
| 941 | CfaAffx.16367.1.S1_s_at | LOC488247 | similar to C27H2.3 | 1.11E-05 | -2.01 |
| 942 | Cfa.3117.2.A1_a_at | SERPING1 | serpin peptidase inhibitor, clade G (C1 inhibitor), member 1 | 0.00158838 | -2.01 |
| 943 | Cfa.11451.1.A1_at | --- | --- | 1.39E-05 | -2.01 |
| 944 | Cfa.7741.1.A1_at | --- | --- | 2.92E-05 | -2.01 |
| 945 | Cfa.12275.1.A1_at | LOC608733 | similar to CG14182-PA | 3.52E-05 | -2.01 |
| 946 | Cfa.2671.3.A1_x_at | EPB41L3 | erythrocyte membrane protein band 4.1-like 3 | 0.000579471 | -2.01 |
| 947 | CfaAffx.1502.1.S1_at | SASH1 | SAM and SH3 domain containing 1 | 7.82E-07 | -2.01 |
| 948 | CfaAffx.6815.1.S1_at | SLC16A10 | solute carrier family 16, member 10 (aromatic amino acid transporter) | 9.97E-05 | -2.01 |
| 949 | CfaAffx.18891.1.S1_at | GRK5 | G protein-coupled receptor kinase 5 | 1.62E-05 | -2.01 |
| 950 | Cfa.10838.1.A1_at | --- | --- | 3.69E-05 | -2.01 |
| 951 | Cfa.5832.1.A1_a_at | --- | --- | 0.000395771 | -2.01 |
| 952 | CfaAffx.17255.1.S1_at | BCL9 | B-cell CLL/lymphoma 9 | 0.000202811 | -2.01 |
| 953 | Cfa.6192.1.A1_at | --- | --- | 1.02E-09 | -2.01 |
| 954 | CfaAffx.20384.1.S1_at | TAGLN | transgelin | 0.00561104 | -2.01 |
| 955 | CfaAffx.9248.1.S1_at | P4HA3 | prolyl 4-hydroxylase, alpha polypeptide III | 4.37E-08 | -2.01 |
| 956 | CfaAffx.7554.1.S1_s_at | BEX4 | brain expressed, X-linked 4 | 0.0154996 | -2.01 |
| 957 | Cfa.5027.1.A1_at | --- | --- | 5.83E-06 | -2.01 |
| 958 | Cfa.13941.1.A1_at | --- | --- | 4.39E-07 | -2.01 |
| 959 | CfaAffx.2878.1.S1_s_at | LOC474698 | similar to catenin (cadherin-associated protein), alpha 1, 102kDa | 7.50E-07 | -2.01 |
| 960 | Cfa.9043.1.A1_at | NRG1 | neuregulin 1 | 0.013992 | -2.01 |
| 961 | CfaAffx.15365.1.S1_s_at | NAV2 | neuron navigator 2 | 1.78E-06 | -2.01 |
| 962 | CfaAffx.10305.1.S1_at | ARAP3 | ArfGAP with RhoGAP domain, ankyrin repeat and PH domain 3 | 9.57E-08 | -2.01 |
| 963 | Cfa.20994.1.S1_s_at | LOC610426 | similar to R17.3 | 0.00115658 | -2.01 |
| 964 | Cfa.17439.1.S1_at | TNFRSF21 | tumor necrosis factor receptor superfamily, member 21 | 7.06E-08 | -2.01 |
| 965 | Cfa.2765.1.A1_at | LOC610479 | hypothetical protein LOC610479 | 2.82E-05 | -2.01 |
| 966 | Cfa.15086.1.A1_at | MOBKL2B | MOB1, Mps One Binder kinase activator-like 2B (yeast) | 0.0176309 | -2.01 |
| 967 | Cfa.3957.1.A1_at | FADS3 | fatty acid desaturase 3 | 1.79E-07 | -2.02 |
| 968 | Cfa.2004.1.S1_at | --- | --- | 2.42E-07 | -2.02 |
| 969 | Cfa.11775.1.A1_at | --- | --- | 2.80E-06 | -2.02 |
| 970 | Cfa.14966.1.A1_at | --- | --- | 9.82E-07 | -2.02 |
| 971 | CfaAffx.20382.1.S1_at | NPL | N-acetylneuraminate pyruvate lyase (dihydrodipicolinate synthase) | 0.000378669 | -2.02 |
| 972 | CfaAffx.6155.1.S1_at | TSPAN12 | tetraspanin 12 | 0.00030665 | -2.02 |
| 973 | Cfa.4373.1.A1_at | TSPAN7 | tetraspanin 7 | 1.57E-05 | -2.02 |
| 974 | Cfa.14693.1.S1_at | --- | --- | 4.71E-09 | -2.02 |
| 975 | Cfa.5439.1.A1_at | --- | --- | 0.00149803 | -2.02 |
| 976 | Cfa.19110.1.S1_s_at | Sep-11 | septin 11 | 0.000815697 | -2.02 |
| 977 | Cfa.7796.1.A1_at | --- | --- | 1.14E-06 | -2.02 |
| 978 | CfaAffx.11455.1.S1_at | LOC485825 | similar to cytochrome c oxidase subunit IV isoform 2 precursor | 9.23E-05 | -2.02 |
| 979 | Cfa.4276.1.S1_at | LOC610645 | hypothetical protein LOC610645 | 4.49E-08 | -2.02 |
| 980 | CfaAffx.22221.1.S1_s_at | C1S | complement component 1, s subcomponent | 0.000235364 | -2.02 |
| 981 | CfaAffx.18723.1.S1_at | PDIA5 | protein disulfide isomerase family A, member 5 | 0.000828075 | -2.02 |
| 982 | Cfa.8104.1.S1_at | CARHSP1 | calcium regulated heat stable protein 1, 24kDa | 0.000228618 | -2.02 |
| 983 | Cfa.9325.1.A1_s_at | FXYD6 | FXYD domain containing ion transport regulator 6 | 4.69E-07 | -2.02 |
| 984 | Cfa.9115.1.A1_at | NTNG1 | netrin G1 | 3.43E-09 | -2.02 |
| 985 | Cfa.15582.1.A1_at | --- | --- | 0.00117955 | -2.02 |
| 986 | Cfa.7645.1.A1_at | --- | --- | 2.95E-07 | -2.02 |
| 987 | Cfa.12268.1.A1_at | --- | --- | 1.02E-09 | -2.02 |
| 988 | CfaAffx.4441.1.S1_at | LOC611701 | hypothetical protein LOC611701 | 5.58E-06 | -2.02 |
| 989 | Cfa.14600.1.A1_at | NXN | nucleoredoxin | 0.000495637 | -2.02 |
| 990 | Cfa.13321.1.A1_at | --- | --- | 2.20E-05 | -2.02 |
| 991 | Cfa.10897.1.A1_at | HHATL | hedgehog acyltransferase-like | 0.00764464 | -2.02 |
| 992 | CfaAffx.17965.1.S1_s_at | CLCN4 | chloride channel 4 | 2.75E-09 | -2.02 |
| 993 | CfaAffx.23810.1.S1_at | DLG5 | discs, large homolog 5 (Drosophila) | 3.28E-06 | -2.02 |
| 994 | CfaAffx.8253.1.S1_s_at | KRTCAP3 | keratinocyte associated protein 3 | 1.71E-10 | -2.02 |
| 995 | CfaAffx.15778.1.S1_at | CMBL | carboxymethylenebutenolidase homolog (Pseudomonas) | 0.00149452 | -2.02 |
| 996 | Cfa.39.1.S1_s_at | IL4 | interleukin 4 | 4.41E-07 | -2.02 |
| 997 | CfaAffx.8426.1.S1_at | NCKAP5 | NCK-associated protein 5 | 3.69E-08 | -2.02 |
| 998 | Cfa.4335.1.S1_at | NISCH | nischarin | 5.91E-09 | -2.02 |
| 999 | Cfa.19836.1.S1_at | LOC476359 | similar to RIKEN cDNA 1110007C09 | 7.24E-05 | -2.02 |
| 1000 | CfaAffx.28670.1.S1_at | LOC611589 | similar to fibrous sheath interacting protein 2 | 2.81E-05 | -2.02 |
| 1001 | Cfa.21163.1.S1_s_at | MFAP3L | microfibrillar-associated protein 3-like | 0.000607282 | -2.02 |
| 1002 | CfaAffx.247.1.S1_at | LOC607255 | similar to Ig lambda chain V-I region BL2 precursor | 2.34E-05 | -2.02 |
| 1003 | CfaAffx.9175.1.S1_s_at | RBMS3 | RNA binding motif, single stranded interacting protein 3 | 7.43E-05 | -2.02 |
| 1004 | Cfa.4374.1.S1_s_at | SELM | selenoprotein M | 0.000219753 | -2.03 |
| 1005 | Cfa.21612.2.S1_a_at | LOC476036 | similar to vascular endothelial growth factor B | 3.35E-08 | -2.03 |
| 1006 | CfaAffx.9807.1.S1_x_at | --- | --- | 0.00127051 | -2.03 |
| 1007 | CfaAffx.30694.1.S1_s_at | SNX7 | sorting nexin 7 | 0.000814461 | -2.03 |
| 1008 | CfaAffx.28671.1.S1_at | LOC490554 | similar to CG8486-PA, isoform A | 0.000311415 | -2.03 |
| 1009 | Cfa.10478.2.A1_at | --- | --- | 8.26E-11 | -2.03 |
| 1010 | Cfa.11289.1.A1_at | --- | --- | 4.23E-07 | -2.03 |
| 1011 | Cfa.16449.1.A1_at | --- | --- | 0.000174193 | -2.03 |
| 1012 | Cfa.13279.1.A1_at | --- | --- | 0.000175414 | -2.03 |
| 1013 | Cfa.2070.1.S1_at | --- | --- | 0.00922937 | -2.03 |
| 1014 | CfaAffx.10706.1.S1_at | MITF | microphthalmia-associated transcription factor | 1.41E-05 | -2.03 |
| 1015 | Cfa.9545.1.A1_at | NOVA1 | neuro-oncological ventral antigen 1 | 0.000302926 | -2.03 |
| 1016 | CfaAffx.6156.1.S1_s_at | TSPAN12 | tetraspanin 12 | 0.00102777 | -2.03 |
| 1017 | Cfa.18839.1.S1_at | --- | --- | 0.000100607 | -2.03 |
| 1018 | Cfa.5554.1.A1_at | --- | --- | 0.00619845 | -2.03 |
| 1019 | Cfa.19494.1.S1_at | --- | --- | 7.97E-05 | -2.03 |
| 1020 | Cfa.14495.2.S1_at | CFI | complement factor I | 0.00203039 | -2.03 |
| 1021 | Cfa.12195.4.A1_at | --- | --- | 0.00393176 | -2.03 |
| 1022 | CfaAffx.12653.1.S1_s_at | --- | --- | 0.00746056 | -2.03 |
| 1023 | Cfa.10241.1.A1_at | --- | --- | 0.000450992 | -2.03 |
| 1024 | Cfa.14256.1.A1_at | --- | --- | 1.91E-08 | -2.03 |
| 1025 | Cfa.2424.1.S1_at | SVEP1 | sushi, von Willebrand factor type A, EGF and pentraxin domain containing 1 | 0.0124758 | -2.03 |
| 1026 | Cfa.9525.1.A1_at | --- | --- | 2.98E-11 | -2.03 |
| 1027 | Cfa.11904.1.A1_at | VWA5B2 | von Willebrand factor A domain containing 5B2 | 1.17E-10 | -2.03 |
| 1028 | Cfa.10182.1.A1_at | --- | --- | 0.0013162 | -2.03 |
| 1029 | Cfa.11717.1.A1_at | LOC612065 | similar to basement membrane-induced gene | 0.00211198 | -2.04 |
| 1030 | CfaAffx.7315.1.S1_at | THNSL1 | threonine synthase-like 1 (S. cerevisiae) | 0.00901921 | -2.04 |
| 1031 | Cfa.15457.1.A1_at | SCUBE3 | signal peptide, CUB domain, EGF-like 3 | 0.000121776 | -2.04 |
| 1032 | Cfa.3709.1.S1_s_at | PLA2G7 | phospholipase A2, group VII (platelet-activating factor acetylhydrolase, plasma) | 0.0163609 | -2.04 |
| 1033 | CfaAffx.29825.1.S1_s_at | CIRBP | cold inducible RNA binding protein | 8.94E-08 | -2.04 |
| 1034 | CfaAffx.22373.1.S1_at | PVRIG | poliovirus receptor related immunoglobulin domain containing | 0.000245626 | -2.04 |
| 1035 | CfaAffx.5703.1.S1_at | MACF1 | microtubule-actin crosslinking factor 1 | 6.86E-08 | -2.04 |
| 1036 | Cfa.15223.1.A1_at | --- | --- | 0.0143387 | -2.04 |
| 1037 | CfaAffx.19566.1.S1_at | LMO3 | LIM domain only 3 (rhombotin-like 2) | 0.00304217 | -2.04 |
| 1038 | Cfa.2637.1.A1_at | --- | --- | 0.00236094 | -2.04 |
| 1039 | CfaAffx.9700.1.S1_at | PLCB4 | phospholipase C, beta 4 | 5.03E-07 | -2.04 |
| 1040 | CfaAffx.20869.1.S1_s_at | CLEC7A | C-type lectin domain family 7, member A | 4.33E-05 | -2.04 |
| 1041 | Cfa.5360.1.A1_at | ANO3 | anoctamin 3 | 6.85E-07 | -2.04 |
| 1042 | CfaAffx.22612.1.S1_s_at | MECOM | MDS1 and EVI1 complex locus | 0.000307099 | -2.04 |
| 1043 | Cfa.14142.1.A1_at | --- | --- | 7.24E-05 | -2.04 |
| 1044 | Cfa.6030.1.A1_at | --- | --- | 0.0205352 | -2.04 |
| 1045 | Cfa.12514.1.A1_at | BAG3 | BCL2-associated athanogene 3 | 1.49E-05 | -2.04 |
| 1046 | Cfa.18912.1.S1_at | LOC611771 | hypothetical protein LOC611771 | 0.00127744 | -2.04 |
| 1047 | Cfa.6663.1.A1_at | CDKN2D | cyclin-dependent kinase inhibitor 2D (p19, inhibits CDK4) | 0.000132721 | -2.04 |
| 1048 | Cfa.3572.1.S1_s_at | ME1 | malic enzyme 1, NADP(+)-dependent, cytosolic | 0.00649913 | -2.04 |
| 1049 | CfaAffx.24405.1.S1_at | CCPG1 | cell cycle progression 1 | 2.05E-05 | -2.04 |
| 1050 | Cfa.458.2.S1_s_at | CDH11 | cadherin 11, type 2, OB-cadherin (osteoblast) | 1.08E-06 | -2.04 |
| 1051 | Cfa.4008.1.A1_at | LARGE | like-glycosyltransferase | 3.51E-05 | -2.04 |
| 1052 | Cfa.1230.1.S1_at | GPM6A | glycoprotein M6A | 8.73E-05 | -2.05 |
| 1053 | Cfa.12979.1.S1_at | --- | --- | 3.01E-05 | -2.05 |
| 1054 | Cfa.7055.1.A1_at | --- | --- | 0.000143486 | -2.05 |
| 1055 | CfaAffx.9991.1.S1_s_at | FGFR1 | fibroblast growth factor receptor 1 | 0.00155396 | -2.05 |
| 1056 | Cfa.12566.1.A1_at | --- | --- | 5.95E-06 | -2.05 |
| 1057 | Cfa.21306.1.S1_at | TSPAN13 | tetraspanin 13 | 1.21E-05 | -2.05 |
| 1058 | Cfa.20703.1.S1_s_at | ZYX | zyxin | 3.69E-07 | -2.05 |
| 1059 | Cfa.14614.1.A1_a_at | --- | --- | 2.53E-06 | -2.05 |
| 1060 | Cfa.3117.1.S1_at | SERPING1 | serpin peptidase inhibitor, clade G (C1 inhibitor), member 1 | 0.00364829 | -2.05 |
| 1061 | Cfa.18362.1.S1_at | LCP2 | lymphocyte cytosolic protein 2 (SH2 domain containing leukocyte protein of 76kDa | 3.86E-05 | -2.05 |
| 1062 | CfaAffx.9762.1.S1_s_at | TMTC4 | transmembrane and tetratricopeptide repeat containing 4 | 0.00529594 | -2.05 |
| 1063 | Cfa.20601.1.S1_at | LOC609596 | similar to Tetranectin precursor (TN) (Plasminogen-kringle 4 binding protein) | 0.00283122 | -2.05 |
| 1064 | Cfa.17933.1.S1_s_at | DLA-79 | MHC class Ib | 0.00362251 | -2.06 |
| 1065 | CfaAffx.30696.1.S1_at | SNX7 | sorting nexin 7 | 0.00370239 | -2.06 |
| 1066 | Cfa.14797.1.A1_at | ZNF169 | zinc finger protein 169 | 6.80E-07 | -2.06 |
| 1067 | Cfa.5095.1.A1_s_at | LOC474640 | similar to Small conductance calcium-activated potassium channel protein 2 (SK2) | 6.62E-07 | -2.06 |
| 1068 | Cfa.7443.1.S1_at | DCHS1 | dachsous 1 (Drosophila) | 2.01E-12 | -2.06 |
| 1069 | Cfa.10802.1.S1_at | LOC490693 | similar to pleckstrin homology domain containing, family C (with FERM domain) me | 6.18E-06 | -2.06 |
| 1070 | Cfa.8099.1.A1_at | --- | --- | 1.34E-10 | -2.06 |
| 1071 | Cfa.5699.1.A1_at | --- | --- | 0.0087193 | -2.06 |
| 1072 | Cfa.13073.1.A1_x_at | PLP2 | proteolipid protein 2 (colonic epithelium-enriched) | 3.34E-05 | -2.06 |
| 1073 | Cfa.16788.1.A1_at | --- | --- | 4.75E-05 | -2.06 |
| 1074 | Cfa.9591.1.A1_at | --- | --- | 2.13E-08 | -2.06 |
| 1075 | CfaAffx.7353.1.S1_s_at | MYO3A | myosin IIIA | 0.000292359 | -2.06 |
| 1076 | CfaAffx.5433.1.S1_s_at | ZNF483 | zinc finger protein 483 | 1.87E-09 | -2.06 |
| 1077 | Cfa.14609.1.A1_at | RHBDF1 | rhomboid 5 homolog 1 (Drosophila) | 2.37E-07 | -2.06 |
| 1078 | CfaAffx.9972.1.S1_s_at | COLQ | collagen-like tail subunit (single strand of homotrimer) of asymmetric acetylcho | 8.07E-05 | -2.06 |
| 1079 | CfaAffx.814.1.S1_at | LOC608983 | similar to T-cell receptor beta chain V region YT35 precursor | 7.04E-07 | -2.06 |
| 1080 | CfaAffx.12654.1.S1_s_at | RBP4 | retinol binding protein 4, plasma | 0.00876361 | -2.06 |
| 1081 | Cfa.10859.1.S1_at | LOC488247 | similar to C27H2.3 | 4.15E-06 | -2.06 |
| 1082 | Cfa.12238.1.A1_a_at | TAGLN | transgelin | 0.00135997 | -2.06 |
| 1083 | CfaAffx.28442.1.S1_s_at | TRIP10 | thyroid hormone receptor interactor 10 | 2.00E-09 | -2.06 |
| 1084 | Cfa.102.1.S1_s_at | PLA2G15 | phospholipase A2, group XV | 9.72E-07 | -2.06 |
| 1085 | CfaAffx.8197.1.S1_s_at | CTDSPL | CTD (carboxy-terminal domain, RNA polymerase II, polypeptide A) small phosphatas | 0.000390721 | -2.06 |
| 1086 | Cfa.3162.1.A1_at | --- | --- | 5.95E-08 | -2.06 |
| 1087 | Cfa.11018.1.A1_at | --- | --- | 2.55E-07 | -2.06 |
| 1088 | Cfa.9886.1.A1_at | --- | --- | 0.000119799 | -2.06 |
| 1089 | Cfa.2580.1.A1_at | --- | --- | 0.00022169 | -2.07 |
| 1090 | Cfa.15179.1.S1_at | --- | --- | 2.82E-08 | -2.07 |
| 1091 | Cfa.5397.1.A1_at | --- | --- | 3.90E-05 | -2.07 |
| 1092 | CfaAffx.23576.1.S1_s_at | PLVAP | plasmalemma vesicle associated protein | 0.000773677 | -2.07 |
| 1093 | Cfa.14344.1.A1_at | --- | --- | 3.18E-11 | -2.07 |
| 1094 | Cfa.9875.1.A1_at | LOC475877 | similar to Amphiphysin | 0.000392151 | -2.07 |
| 1095 | Cfa.15611.1.A1_at | --- | --- | 1.87E-06 | -2.07 |
| 1096 | Cfa.15078.1.S1_s_at | PCOLCE | procollagen C-endopeptidase enhancer | 7.64E-06 | -2.07 |
| 1097 | CfaAffx.11702.1.S1_at | LOC607366 | similar to family with sequence similarity 3, member D | 5.06E-07 | -2.07 |
| 1098 | Cfa.12875.1.A1_at | --- | --- | 0.000113581 | -2.07 |
| 1099 | CfaAffx.12482.1.S1_at | CTNND1 | catenin (cadherin-associated protein), delta 1 | 3.61E-05 | -2.07 |
| 1100 | Cfa.17396.1.S1_a_at | N4BP2L1 | NEDD4 binding protein 2-like 1 | 4.10E-05 | -2.07 |
| 1101 | CfaAffx.4230.1.S1_at | NPR2 | natriuretic peptide receptor B/guanylate cyclase B (atrionatriuretic peptide rec | 9.69E-07 | -2.07 |
| 1102 | Cfa.3550.2.A1_at | MGST1 | microsomal glutathione S-transferase 1 | 0.0197655 | -2.07 |
| 1103 | Cfa.15173.2.S1_s_at | --- | --- | 0.00264767 | -2.07 |
| 1104 | Cfa.14082.1.A1_at | --- | --- | 1.89E-07 | -2.07 |
| 1105 | Cfa.9681.1.A1_at | --- | --- | 0.000131879 | -2.07 |
| 1106 | CfaAffx.27161.1.S1_at | LOC608733 | similar to CG14182-PA | 4.27E-07 | -2.07 |
| 1107 | CfaAffx.6619.1.S1_s_at | --- | --- | 2.26E-08 | -2.07 |
| 1108 | CfaAffx.4021.1.S1_s_at | KIT | v-kit Hardy-Zuckerman 4 feline sarcoma viral oncogene homolog | 0.00564384 | -2.07 |
| 1109 | CfaAffx.22164.1.S1_s_at | LGALS3 | lectin, galactoside-binding, soluble, 3 | 0.00666826 | -2.08 |
| 1110 | Cfa.9911.1.A1_at | --- | --- | 1.70E-09 | -2.08 |
| 1111 | CfaAffx.16313.1.S1_s_at | CPNE8 | copine VIII | 0.00157387 | -2.08 |
| 1112 | CfaAffx.14658.1.S1_s_at | SERPINB9 | serpin peptidase inhibitor, clade B (ovalbumin), member 9 | 3.94E-08 | -2.08 |
| 1113 | Cfa.3007.1.A1_at | --- | --- | 0.00724867 | -2.08 |
| 1114 | Cfa.5871.1.A1_at | ANKRD35 | ankyrin repeat domain 35 | 2.17E-07 | -2.08 |
| 1115 | Cfa.16912.1.S1_s_at | SH3GL3 | SH3-domain GRB2-like 3 | 0.000217524 | -2.08 |
| 1116 | Cfa.2428.1.A1_at | --- | --- | 2.56E-06 | -2.08 |
| 1117 | CfaAffx.22339.1.S1_s_at | IGFBP5 | insulin-like growth factor binding protein 5 | 0.000957391 | -2.08 |
| 1118 | Cfa.11452.2.A1_s_at | SMTN | smoothelin | 1.86E-06 | -2.08 |
| 1119 | CfaAffx.29885.1.S1_s_at | --- | --- | 8.26E-16 | -2.08 |
| 1120 | CfaAffx.16048.1.S1_s_at | ADCY2 | adenylate cyclase 2 (brain) | 0.00770121 | -2.08 |
| 1121 | Cfa.11196.1.A1_at | --- | --- | 7.45E-09 | -2.08 |
| 1122 | Cfa.13869.1.A1_at | FGR | Gardner-Rasheed feline sarcoma viral (v-fgr) oncogene homolog | 0.000122421 | -2.08 |
| 1123 | Cfa.4088.1.A1_s_at | EPHX1 | epoxide hydrolase 1, microsomal (xenobiotic) | 1.53E-06 | -2.08 |
| 1124 | CfaAffx.19879.1.S1_s_at | PTPRO | protein tyrosine phosphatase, receptor type, O | 6.16E-05 | -2.08 |
| 1125 | CfaAffx.26785.1.S1_s_at | NPR1 | natriuretic peptide receptor A/guanylate cyclase A (atrionatriuretic peptide rec | 4.06E-09 | -2.08 |
| 1126 | Cfa.8000.1.A1_at | MAP3K9 | mitogen-activated protein kinase kinase kinase 9 | 1.40E-08 | -2.08 |
| 1127 | Cfa.8911.1.A1_at | --- | --- | 0.00843631 | -2.08 |
| 1128 | Cfa.13560.1.A1_at | --- | --- | 0.00171557 | -2.08 |
| 1129 | Cfa.5832.1.A1_at | --- | --- | 0.000508225 | -2.08 |
| 1130 | Cfa.14225.1.A1_s_at | PPP1R15A | protein phosphatase 1, regulatory (inhibitor) subunit 15A | 2.84E-05 | -2.08 |
| 1131 | CfaAffx.12264.1.S1_s_at | MRVI1 | murine retrovirus integration site 1 homolog | 4.60E-12 | -2.08 |
| 1132 | CfaAffx.26924.1.S1_at | GDPD1 | glycerophosphodiester phosphodiesterase domain containing 1 | 5.45E-06 | -2.08 |
| 1133 | Cfa.14520.1.A1_at | RBP5 | retinol binding protein 5, cellular | 0.00076544 | -2.08 |
| 1134 | CfaAffx.23334.1.S1_x_at | LOC486394 /// LOC491405 | similar to Ig lambda chain V-III region LOI /// similar to immunoglobulin iota c | 0.000441387 | -2.08 |
| 1135 | Cfa.9915.1.A1_at | --- | --- | 0.000115906 | -2.08 |
| 1136 | CfaAffx.6983.1.S1_at | LAMA4 | laminin, alpha 4 | 0.000882523 | -2.09 |
| 1137 | Cfa.8595.3.A1_at | PGCP | plasma glutamate carboxypeptidase | 6.99E-06 | -2.09 |
| 1138 | Cfa.12674.1.A1_s_at | --- | --- | 0.000979794 | -2.09 |
| 1139 | Cfa.1882.1.A1_at | --- | --- | 1.63E-08 | -2.09 |
| 1140 | Cfa.1379.1.S1_at | C1QA | complement component 1, q subcomponent, A chain | 0.00192808 | -2.09 |
| 1141 | Cfa.12149.1.S1_at | ANXA5 | annexin A5 | 0.00037812 | -2.09 |
| 1142 | Cfa.15371.1.A1_at | --- | --- | 1.43E-05 | -2.09 |
| 1143 | Cfa.13762.1.A1_at | --- | --- | 0.00777429 | -2.09 |
| 1144 | Cfa.19358.1.S1_s_at | TMEFF1 | transmembrane protein with EGF-like and two follistatin-like domains 1 | 0.00142712 | -2.09 |
| 1145 | CfaAffx.13799.1.S1_at | --- | --- | 0.000490149 | -2.09 |
| 1146 | Cfa.4700.1.A1_at | --- | --- | 1.01E-05 | -2.09 |
| 1147 | Cfa.15703.1.A1_at | RNF144B | ring finger protein 144B | 4.66E-07 | -2.09 |
| 1148 | Cfa.12316.1.A1_at | ARHGEF3 | Rho guanine nucleotide exchange factor (GEF) 3 | 0.000459506 | -2.09 |
| 1149 | CfaAffx.5597.1.S1_s_at | ANKRD6 | ankyrin repeat domain 6 | 0.00419154 | -2.09 |
| 1150 | Cfa.15298.1.A1_at | --- | --- | 5.15E-05 | -2.09 |
| 1151 | Cfa.12195.6.A1_at | PDLIM1 | PDZ and LIM domain 1 | 2.74E-05 | -2.09 |
| 1152 | CfaAffx.19799.1.S1_s_at | --- | --- | 7.28E-06 | -2.09 |
| 1153 | Cfa.9685.2.S1_a_at | TSC22D1 | TSC22 domain family, member 1 | 4.83E-05 | -2.09 |
| 1154 | Cfa.3463.1.S1_s_at | --- | --- | 1.04E-06 | -2.09 |
| 1155 | Cfa.2671.3.A1_a_at | EPB41L3 | erythrocyte membrane protein band 4.1-like 3 | 0.000330305 | -2.09 |
| 1156 | Cfa.6604.1.A1_at | --- | --- | 8.98E-05 | -2.09 |
| 1157 | CfaAffx.15487.1.S1_s_at | CD83 | CD83 molecule | 4.12E-05 | -2.09 |
| 1158 | Cfa.14069.1.A1_at | --- | --- | 2.83E-06 | -2.10 |
| 1159 | CfaAffx.24504.1.S1_s_at | FADS3 | fatty acid desaturase 3 | 2.28E-07 | -2.10 |
| 1160 | Cfa.19519.1.S1_at | --- | --- | 9.04E-05 | -2.10 |
| 1161 | Cfa.8107.1.A1_at | --- | --- | 7.30E-07 | -2.10 |
| 1162 | Cfa.1851.2.S1_s_at | --- | --- | 1.29E-07 | -2.10 |
| 1163 | CfaAffx.26780.1.S1_s_at | DOCK6 | dedicator of cytokinesis 6 | 1.46E-10 | -2.10 |
| 1164 | Cfa.13746.1.A1_at | --- | --- | 3.20E-06 | -2.10 |
| 1165 | Cfa.9837.1.A1_at | BEX4 | brain expressed, X-linked 4 | 0.00947062 | -2.10 |
| 1166 | Cfa.2750.1.A1_at | --- | --- | 9.26E-06 | -2.10 |
| 1167 | CfaAffx.24358.1.S1_s_at | NSUN7 | NOP2/Sun domain family, member 7 | 1.38E-08 | -2.10 |
| 1168 | CfaAffx.9168.1.S1_at | DSTN | destrin (actin depolymerizing factor) | 0.000284564 | -2.10 |
| 1169 | Cfa.2898.1.A1_s_at | PTPRM | protein tyrosine phosphatase, receptor type, M | 9.13E-08 | -2.10 |
| 1170 | Cfa.14919.1.A1_at | --- | --- | 0.0098544 | -2.10 |
| 1171 | Cfa.20952.1.S1_s_at | CD200 | CD200 molecule | 1.42E-05 | -2.10 |
| 1172 | CfaAffx.24486.1.S1_at | SLC16A11 | solute carrier family 16, member 11 (monocarboxylic acid transporter 11) | 0.000213961 | -2.10 |
| 1173 | Cfa.1382.1.A1_at | --- | --- | 5.75E-05 | -2.10 |
| 1174 | Cfa.13859.1.A1_at | --- | --- | 1.40E-05 | -2.10 |
| 1175 | Cfa.1185.1.A1_at | --- | --- | 1.70E-08 | -2.10 |
| 1176 | CfaAffx.22302.1.S1_at | LOC610934 | hypothetical protein LOC610934 | 5.11E-05 | -2.10 |
| 1177 | Cfa.3868.1.S1_at | SELE | selectin E | 0.00324559 | -2.10 |
| 1178 | CfaAffx.10969.1.S1_at | --- | --- | 0.000377875 | -2.10 |
| 1179 | Cfa.14088.1.A1_at | LOC610231 | similar to CG10584-PA | 5.46E-10 | -2.10 |
| 1180 | Cfa.3660.1.A1_s_at | NOS2 | nitric oxide synthase 2, inducible | 8.11E-06 | -2.10 |
| 1181 | CfaAffx.6402.1.S1_s_at | FLT3LG | fms-related tyrosine kinase 3 ligand | 1.92E-13 | -2.10 |
| 1182 | CfaAffx.11386.1.S1_at | LOC610510 | similar to nuclear receptor interacting protein 3 | 0.000140741 | -2.10 |
| 1183 | Cfa.15377.1.A1_s_at | FOXO1 | forkhead box O1 | 1.04E-06 | -2.10 |
| 1184 | CfaAffx.9679.1.S1_at | LIN7A | lin-7 homolog A (C. elegans) | 2.30E-05 | -2.10 |
| 1185 | Cfa.16298.1.A1_at | --- | --- | 2.48E-07 | -2.10 |
| 1186 | Cfa.16688.1.A1_at | ago61 | glycosyltransferase | 2.75E-10 | -2.10 |
| 1187 | Cfa.14767.1.A1_at | --- | --- | 3.46E-05 | -2.11 |
| 1188 | Cfa.13236.1.A1_at | --- | --- | 0.00058246 | -2.11 |
| 1189 | CfaAffx.17132.1.S1_at | PLCL1 | phospholipase C-like 1 | 2.53E-06 | -2.11 |
| 1190 | Cfa.1805.1.S1_at | SEMA4F | sema domain, immunoglobulin domain (Ig), transmembrane domain (TM) and short cyt | 1.70E-05 | -2.11 |
| 1191 | CfaAffx.29458.1.S1_s_at | --- | --- | 3.53E-09 | -2.11 |
| 1192 | Cfa.8752.1.A1_at | --- | --- | 3.08E-08 | -2.11 |
| 1193 | CfaAffx.18886.1.S1_s_at | ABCC9 | ATP-binding cassette, sub-family C (CFTR/MRP), member 9 | 1.61E-08 | -2.11 |
| 1194 | Cfa.14267.1.S1_at | CFB | complement factor B | 2.57E-05 | -2.11 |
| 1195 | Cfa.4743.1.A1_at | --- | --- | 2.99E-06 | -2.11 |
| 1196 | Cfa.8312.1.A1_at | --- | --- | 2.30E-08 | -2.11 |
| 1197 | Cfa.12297.1.A1_at | --- | --- | 6.92E-07 | -2.11 |
| 1198 | Cfa.2038.1.S1_at | --- | --- | 1.26E-06 | -2.11 |
| 1199 | Cfa.8947.1.A1_at | --- | --- | 2.16E-11 | -2.11 |
| 1200 | Cfa.12666.1.A1_at | --- | --- | 0.0179882 | -2.11 |
| 1201 | Cfa.5286.1.A1_at | LOC478967 | similar to Mg87 protein | 0.00398764 | -2.11 |
| 1202 | Cfa.12168.1.A1_s_at | LOC478419 | similar to Protein FAM3B precursor (Cytokine-like protein 2-21) | 0.000152986 | -2.11 |
| 1203 | CfaAffx.455.1.S1_s_at | LOC477558 | similar to Glutathione S-transferase theta 2 (GST class-theta 2) | 0.0031742 | -2.12 |
| 1204 | CfaAffx.13190.1.S1_s_at | ZFHX4 | zinc finger homeobox 4 | 0.00133391 | -2.12 |
| 1205 | Cfa.13680.1.A1_at | --- | --- | 5.85E-07 | -2.12 |
| 1206 | Cfa.164.1.A1_at | --- | --- | 5.77E-05 | -2.12 |
| 1207 | CfaAffx.23074.1.S1_at | PRRX1 | paired related homeobox 1 | 6.81E-05 | -2.12 |
| 1208 | Cfa.19458.1.S1_at | OLFML1 | olfactomedin-like 1 | 1.68E-05 | -2.12 |
| 1209 | Cfa.7132.1.A1_at | SLC2A9 | solute carrier family 2 (facilitated glucose transporter), member 9 | 1.99E-05 | -2.12 |
| 1210 | Cfa.4088.1.A1_at | EPHX1 | epoxide hydrolase 1, microsomal (xenobiotic) | 1.10E-06 | -2.12 |
| 1211 | CfaAffx.26534.1.S1_at | TOM1L1 | target of myb1 (chicken)-like 1 | 7.08E-06 | -2.12 |
| 1212 | Cfa.9214.1.A1_at | JAZF1 | JAZF zinc finger 1 | 0.000285357 | -2.12 |
| 1213 | CfaAffx.13101.1.S1_s_at | LOC475470 | similar to tripartite motif protein TRIM2 | 0.00275926 | -2.12 |
| 1214 | CfaAffx.11174.1.S1_s_at | PPAP2A | phosphatidic acid phosphatase type 2A | 3.43E-06 | -2.12 |
| 1215 | Cfa.3696.1.S1_s_at | GBGT1 | globoside alpha-1,3-N-acetylgalactosaminyltransferase 1 | 5.15E-06 | -2.12 |
| 1216 | Cfa.20143.1.S1_at | ATL1 | atlastin GTPase 1 | 0.000139859 | -2.12 |
| 1217 | Cfa.687.1.A1_at | GNPTG | N-acetylglucosamine-1-phosphate transferase, gamma subunit | 1.14E-05 | -2.12 |
| 1218 | Cfa.14621.1.S1_s_at | FLNA | filamin A, alpha | 9.15E-10 | -2.12 |
| 1219 | Cfa.7131.1.A1_at | --- | --- | 0.000182393 | -2.12 |
| 1220 | CfaAffx.17206.1.S1_s_at | PPFIBP1 | PTPRF interacting protein, binding protein 1 (liprin beta 1) | 4.39E-05 | -2.12 |
| 1221 | CfaAffx.30792.1.S1_s_at | ARHGAP29 | Rho GTPase activating protein 29 | 3.39E-07 | -2.12 |
| 1222 | CfaAffx.1121.1.S1_s_at | ZNF532 | zinc finger protein 532 | 1.12E-06 | -2.12 |
| 1223 | CfaAffx.12463.1.S1_at | LIX1 | Lix1 homolog (chicken) | 0.00601834 | -2.12 |
| 1224 | CfaAffx.4511.1.S1_at | LOC484264 | similar to zinc finger protein 91 (HPF7, HTF10) | 1.62E-06 | -2.12 |
| 1225 | CfaAffx.4141.1.S1_s_at | NFIL3 | nuclear factor, interleukin 3 regulated | 0.00571158 | -2.12 |
| 1226 | Cfa.12131.1.A1_at | CCBL1 | cysteine conjugate-beta lyase, cytoplasmic | 3.92E-07 | -2.12 |
| 1227 | CfaAffx.9219.1.S1_at | UGGT2 | UDP-glucose glycoprotein glucosyltransferase 2 | 1.47E-07 | -2.12 |
| 1228 | CfaAffx.22727.1.S1_s_at | TFPI | tissue factor pathway inhibitor (lipoprotein-associated coagulation inhibitor) | 0.0144867 | -2.12 |
| 1229 | Cfa.11164.1.A1_at | --- | --- | 2.40E-07 | -2.12 |
| 1230 | CfaAffx.5049.1.S1_x_at | ZNF91 | zinc finger protein 91 | 6.33E-05 | -2.12 |
| 1231 | Cfa.11011.1.A1_at | --- | --- | 0.000997524 | -2.12 |
| 1232 | CfaAffx.23910.1.S1_at | MAEL | maelstrom homolog (Drosophila) | 0.0199694 | -2.12 |
| 1233 | Cfa.11119.1.A1_at | --- | --- | 5.70E-09 | -2.12 |
| 1234 | CfaAffx.20968.1.S1_at | LOC486392 /// LOC491364 /// LOC491494 /// LOC607125 /// LOC607213 /// LOC612135 | similar to Ig lambda chain V region 4A precursor /// similar to Ig lambda chain | 0.00018013 | -2.12 |
| 1235 | CfaAffx.28417.1.S1_at | ARHGAP28 | Rho GTPase activating protein 28 | 3.17E-07 | -2.12 |
| 1236 | Cfa.17073.1.S1_at | IGFBP4 | insulin-like growth factor binding protein 4 | 0.000289363 | -2.12 |
| 1237 | CfaAffx.2165.1.S1_s_at | PDLIM4 | PDZ and LIM domain 4 | 9.16E-09 | -2.12 |
| 1238 | Cfa.3438.1.A1_at | --- | --- | 7.99E-08 | -2.12 |
| 1239 | CfaAffx.4263.1.S1_at | ICA1 | islet cell autoantigen 1, 69kDa | 6.15E-07 | -2.12 |
| 1240 | Cfa.643.1.A1_at | --- | --- | 0.000276447 | -2.13 |
| 1241 | Cfa.2923.2.A1_a_at | --- | --- | 2.59E-07 | -2.13 |
| 1242 | CfaAffx.19953.1.S1_at | ICOS | inducible T-cell co-stimulator | 1.08E-06 | -2.13 |
| 1243 | CfaAffx.29217.1.S1_x_at | S100A10 | S100 calcium binding protein A10 | 5.34E-05 | -2.13 |
| 1244 | Cfa.10438.1.A1_at | --- | --- | 0.000152318 | -2.13 |
| 1245 | Cfa.12231.1.A1_at | --- | --- | 9.90E-06 | -2.13 |
| 1246 | Cfa.18913.1.S1_s_at | ZEB2 | zinc finger E-box binding homeobox 2 | 0.000253805 | -2.13 |
| 1247 | Cfa.18308.1.S1_at | TGFBR3 | transforming growth factor, beta receptor III | 0.000160355 | -2.13 |
| 1248 | Cfa.20291.2.A1_at | --- | --- | 6.52E-05 | -2.13 |
| 1249 | CfaAffx.24358.1.S1_at | NSUN7 | NOP2/Sun domain family, member 7 | 2.21E-08 | -2.13 |
| 1250 | Cfa.19337.1.S1_at | SMARCD3 | SWI/SNF related, matrix associated, actin dependent regulator of chromatin, subf | 8.34E-11 | -2.13 |
| 1251 | Cfa.19638.1.S1_s_at | TMLHE | trimethyllysine hydroxylase, epsilon | 5.07E-07 | -2.13 |
| 1252 | Cfa.21098.1.S1_s_at | KCNJ8 | potassium inwardly-rectifying channel, subfamily J, member 8 | 1.14E-07 | -2.13 |
| 1253 | Cfa.10130.1.A1_at | GABARAPL1 | GABA(A) receptor-associated protein like 1 | 5.53E-08 | -2.13 |
| 1254 | Cfa.17994.1.S1_s_at | FAR2 | fatty acyl CoA reductase 2 | 1.89E-08 | -2.13 |
| 1255 | CfaAffx.110.1.S1_at | APBB2 | amyloid beta (A4) precursor protein-binding, family B, member 2 | 0.000140739 | -2.13 |
| 1256 | CfaAffx.4497.1.S1_at | MEOX2 | mesenchyme homeobox 2 | 1.28E-05 | -2.13 |
| 1257 | Cfa.4333.1.A1_at | CTSF | cathepsin F | 7.10E-07 | -2.13 |
| 1258 | CfaAffx.26205.1.S1_s_at | NUPR1 | nuclear protein, transcriptional regulator, 1 | 0.00709281 | -2.13 |
| 1259 | Cfa.8502.1.A1_at | --- | --- | 2.03E-08 | -2.13 |
| 1260 | Cfa.14230.1.A1_at | PMEPA1 | prostate transmembrane protein, androgen induced 1 | 2.51E-05 | -2.13 |
| 1261 | Cfa.3503.1.S1_at | GP1BA | glycoprotein Ib | 3.78E-07 | -2.13 |
| 1262 | Cfa.450.1.S1_at | ARMCX1 | armadillo repeat containing, X-linked 1 | 0.00153914 | -2.13 |
| 1263 | Cfa.13488.1.A1_at | --- | --- | 0.00283842 | -2.13 |
| 1264 | Cfa.3460.1.S1_s_at | --- | --- | 3.92E-09 | -2.14 |
| 1265 | Cfa.15698.1.A1_at | LOC608135 | similar to Non-muscle caldesmon (CDM) (L-caldesmon) | 3.45E-06 | -2.14 |
| 1266 | CfaAffx.9236.1.S1_at | MBNL2 | muscleblind-like 2 (Drosophila) | 2.16E-05 | -2.14 |
| 1267 | Cfa.676.1.S1_at | --- | --- | 0.000382091 | -2.14 |
| 1268 | Cfa.11975.1.A1_a_at | EPHB6 | EPH receptor B6 | 2.11E-09 | -2.14 |
| 1269 | CfaAffx.12840.1.S1_s_at | SH3D19 | SH3 domain containing 19 | 1.37E-05 | -2.14 |
| 1270 | CfaAffx.4016.1.S1_s_at | GPR116 | G protein-coupled receptor 116 | 0.000367621 | -2.14 |
| 1271 | Cfa.17088.2.S1_at | LOC475615 | similar to mitochondrial tumor suppressor 1 isoform 4 | 2.89E-05 | -2.14 |
| 1272 | CfaAffx.29950.1.S1_s_at | BAIAP3 | BAI1-associated protein 3 | 2.34E-10 | -2.14 |
| 1273 | Cfa.4697.1.A1_at | --- | --- | 5.54E-05 | -2.14 |
| 1274 | CfaAffx.2549.1.S1_at | TMEM71 | transmembrane protein 71 | 0.00130749 | -2.14 |
| 1275 | Cfa.4737.1.A1_at | --- | --- | 0.000104679 | -2.14 |
| 1276 | CfaAffx.581.1.S1_at | --- | --- | 0.000335752 | -2.14 |
| 1277 | CfaAffx.4324.1.S1_at | ECM2 | extracellular matrix protein 2, female organ and adipocyte specific | 1.45E-05 | -2.14 |
| 1278 | Cfa.11473.1.A1_at | B3GNT4 | UDP-GlcNAc:betaGal beta-1,3-N-acetylglucosaminyltransferase 4 | 3.38E-06 | -2.14 |
| 1279 | Cfa.10576.1.A1_at | --- | --- | 4.67E-08 | -2.14 |
| 1280 | Cfa.1428.1.A1_at | SLC2A8 | solute carrier family 2 (facilitated glucose transporter), member 8 | 8.19E-08 | -2.14 |
| 1281 | CfaAffx.1248.1.S1_at | LOC611318 | similar to F55A4.8a | 0.000189333 | -2.15 |
| 1282 | CfaAffx.13836.1.S1_at | FSIP1 | fibrous sheath interacting protein 1 | 0.000117194 | -2.15 |
| 1283 | Cfa.548.1.A1_at | --- | --- | 3.47E-05 | -2.15 |
| 1284 | CfaAffx.2245.1.S1_at | LOC484089 | similar to ribosomal protein L31 | 6.74E-05 | -2.15 |
| 1285 | CfaAffx.1704.1.S1_s_at | DLA-64 | MHC class I DLA-64 | 1.29E-05 | -2.15 |
| 1286 | CfaAffx.4852.1.S1_at | LAIR1 | leukocyte-associated immunoglobulin-like receptor 1 | 0.000687811 | -2.15 |
| 1287 | Cfa.11916.1.S1_at | SGCA | sarcoglycan, alpha (50kDa dystrophin-associated glycoprotein) | 2.98E-07 | -2.15 |
| 1288 | Cfa.10786.1.A1_s_at | PDIA5 | protein disulfide isomerase family A, member 5 | 0.00012196 | -2.15 |
| 1289 | Cfa.515.1.A1_at | --- | --- | 0.0175993 | -2.15 |
| 1290 | Cfa.2973.1.A1_at | SHE | Src homology 2 domain containing E | 8.36E-06 | -2.15 |
| 1291 | Cfa.15433.1.A1_at | --- | --- | 2.12E-05 | -2.15 |
| 1292 | Cfa.9711.1.A1_at | STXBP5 | syntaxin binding protein 5 (tomosyn) | 0.0195797 | -2.15 |
| 1293 | CfaAffx.15436.1.S1_at | PLTP | phospholipid transfer protein | 8.51E-05 | -2.15 |
| 1294 | Cfa.14896.1.A1_at | --- | --- | 0.00186596 | -2.15 |
| 1295 | Cfa.11828.1.A1_at | STOM | stomatin | 0.00433198 | -2.15 |
| 1296 | CfaAffx.27899.1.S1_at | CSF1R | colony stimulating factor 1 receptor | 8.75E-05 | -2.15 |
| 1297 | Cfa.458.1.A1_s_at | CDH11 | cadherin 11, type 2, OB-cadherin (osteoblast) | 7.72E-07 | -2.15 |
| 1298 | Cfa.6690.1.A1_at | --- | --- | 2.22E-06 | -2.15 |
| 1299 | Cfa.9203.1.A1_at | --- | --- | 0.00131515 | -2.15 |
| 1300 | CfaAffx.11087.1.S1_s_at | --- | --- | 3.88E-08 | -2.16 |
| 1301 | Cfa.837.1.S1_at | --- | --- | 1.11E-05 | -2.16 |
| 1302 | CfaAffx.4513.1.S1_s_at | LOC484264 | similar to zinc finger protein 91 (HPF7, HTF10) | 0.000545005 | -2.16 |
| 1303 | Cfa.16571.1.S1_s_at | MAP1B | microtubule-associated protein 1B | 0.0026353 | -2.16 |
| 1304 | Cfa.11238.1.A1_at | --- | --- | 1.38E-06 | -2.16 |
| 1305 | CfaAffx.2782.1.S1_s_at | EGR1 | early growth response 1 | 0.00919512 | -2.16 |
| 1306 | Cfa.16308.1.S1_a_at | PMP22 | peripheral myelin protein 22 | 0.00123181 | -2.16 |
| 1307 | Cfa.18164.1.S1_at | SHB | Src homology 2 domain containing adaptor protein B | 4.89E-06 | -2.16 |
| 1308 | Cfa.15829.1.S1_at | CAV2 | caveolin 2 | 0.00183254 | -2.16 |
| 1309 | Cfa.661.1.A1_at | --- | --- | 3.58E-05 | -2.16 |
| 1310 | CfaAffx.3656.1.S1_s_at | LOC482284 | similar to Multidrug resistance protein 3 (P-glycoprotein 3) | 1.31E-06 | -2.16 |
| 1311 | CfaAffx.3252.1.S1_at | CNTLN | centlein, centrosomal protein | 0.00211788 | -2.16 |
| 1312 | Cfa.15828.1.S1_at | RRBP1 | ribosome binding protein 1 homolog 180kDa (dog) | 1.57E-05 | -2.16 |
| 1313 | Cfa.7184.1.A1_at | RILP | Rab interacting lysosomal protein | 1.59E-08 | -2.16 |
| 1314 | CfaAffx.12229.1.S1_at | LYVE1 | lymphatic vessel endothelial hyaluronan receptor 1 | 0.00268587 | -2.16 |
| 1315 | Cfa.4802.1.S1_at | TNFSF12-TNFSF13 | TNFSF12-TNFSF13 readthrough | 1.43E-06 | -2.16 |
| 1316 | Cfa.15776.1.A1_at | --- | --- | 2.11E-07 | -2.16 |
| 1317 | Cfa.3619.1.S1_at | CD38 | CD38 molecule | 0.00160824 | -2.16 |
| 1318 | CfaAffx.14248.1.S1_s_at | TMEM55A | transmembrane protein 55A | 2.83E-08 | -2.16 |
| 1319 | CfaAffx.20968.1.S1_x_at | LOC486392 /// LOC491364 /// LOC491494 /// LOC607125 /// LOC607213 /// LOC612135 | similar to Ig lambda chain V region 4A precursor /// similar to Ig lambda chain | 7.79E-05 | -2.16 |
| 1320 | Cfa.13855.2.S1_s_at | MECOM | MDS1 and EVI1 complex locus | 4.19E-06 | -2.16 |
| 1321 | CfaAffx.18986.1.S1_at | GAS2L1 | growth arrest-specific 2 like 1 | 1.10E-06 | -2.16 |
| 1322 | Cfa.15378.1.A1_at | LOC609573 | hypothetical protein LOC609573 | 2.90E-07 | -2.16 |
| 1323 | Cfa.8772.2.A1_at | LOC478722 | similar to CG3625-PB, isoform B | 0.000220057 | -2.16 |
| 1324 | Cfa.16270.1.S1_at | Sep-05 | septin 5 | 2.33E-05 | -2.16 |
| 1325 | Cfa.13196.1.A1_at | ASS1 | argininosuccinate synthase 1 | 5.38E-05 | -2.16 |
| 1326 | Cfa.1441.1.S1_at | --- | --- | 0.00192293 | -2.16 |
| 1327 | CfaAffx.17216.1.S1_s_at | SCN3A | sodium channel, voltage-gated, type III, alpha subunit | 0.001808 | -2.16 |
| 1328 | CfaAffx.10423.1.S1_s_at | CHL1 | cell adhesion molecule with homology to L1CAM (close homolog of L1) | 0.000688795 | -2.16 |
| 1329 | Cfa.3102.1.A1_at | --- | --- | 0.00224028 | -2.17 |
| 1330 | CfaAffx.835.1.S1_s_at | LOC491855 /// MYL9 | similar to Myosin regulatory light chain 2, smooth muscle isoform (Myosin RLC) ( | 0.000182496 | -2.17 |
| 1331 | CfaAffx.247.1.S1_x_at | LOC607255 | similar to Ig lambda chain V-I region BL2 precursor | 7.47E-06 | -2.17 |
| 1332 | Cfa.16194.1.S1_at | CAMK1 | calcium/calmodulin-dependent protein kinase I | 6.88E-09 | -2.17 |
| 1333 | Cfa.411.1.A1_at | DHRS4 | dehydrogenase/reductase (SDR family) member 4 | 3.02E-05 | -2.17 |
| 1334 | Cfa.16248.1.S1_at | CCND1 | cyclin D1 | 5.55E-05 | -2.17 |
| 1335 | Cfa.14914.1.A1_at | --- | --- | 2.59E-06 | -2.17 |
| 1336 | Cfa.17079.2.S1_a_at | DENND2D | DENN/MADD domain containing 2D | 2.64E-07 | -2.17 |
| 1337 | Cfa.13106.1.A1_x_at | --- | --- | 0.0019315 | -2.17 |
| 1338 | Cfa.19323.1.S1_s_at | --- | --- | 0.000165066 | -2.17 |
| 1339 | Cfa.12336.1.A1_at | --- | --- | 1.64E-05 | -2.17 |
| 1340 | CfaAffx.28520.1.S1_at | EGFLAM | EGF-like, fibronectin type III and laminin G domains | 0.000760941 | -2.17 |
| 1341 | Cfa.1297.1.A1_at | TMEFF2 | transmembrane protein with EGF-like and two follistatin-like domains 2 | 0.0128206 | -2.17 |
| 1342 | Cfa.2682.1.S1_s_at | MITF | microphthalmia-associated transcription factor | 3.08E-08 | -2.17 |
| 1343 | Cfa.10910.1.A1_s_at | LAMB1 | laminin, beta 1 | 0.000422158 | -2.17 |
| 1344 | Cfa.8151.1.A1_at | --- | --- | 0.000133355 | -2.17 |
| 1345 | Cfa.3636.1.S1_s_at | CD8A | CD8a molecule | 1.66E-05 | -2.17 |
| 1346 | Cfa.15152.1.A1_at | LOC479114 | similar to CG9590-PA | 3.21E-09 | -2.17 |
| 1347 | Cfa.3621.1.A1_at | --- | --- | 5.38E-05 | -2.17 |
| 1348 | Cfa.1612.1.A1_at | --- | --- | 3.83E-08 | -2.17 |
| 1349 | Cfa.12315.1.A1_at | --- | --- | 5.92E-06 | -2.17 |
| 1350 | CfaAffx.7307.1.S1_s_at | PRTFDC1 | phosphoribosyl transferase domain containing 1 | 1.56E-08 | -2.17 |
| 1351 | Cfa.4487.1.S1_at | --- | --- | 1.32E-05 | -2.17 |
| 1352 | Cfa.3425.1.A1_at | --- | --- | 0.0123522 | -2.17 |
| 1353 | CfaAffx.24207.1.S1_s_at | PLP2 | proteolipid protein 2 (colonic epithelium-enriched) | 0.00012519 | -2.18 |
| 1354 | Cfa.2486.1.S1_at | --- | --- | 1.44E-05 | -2.18 |
| 1355 | Cfa.14607.1.A1_at | --- | --- | 5.46E-06 | -2.18 |
| 1356 | Cfa.11603.1.A1_at | --- | --- | 9.24E-08 | -2.18 |
| 1357 | Cfa.19459.1.S1_at | PIAS1 | protein inhibitor of activated STAT, 1 | 0.00729541 | -2.18 |
| 1358 | Cfa.4658.1.A1_at | PKIG | protein kinase (cAMP-dependent, catalytic) inhibitor gamma | 8.14E-10 | -2.18 |
| 1359 | Cfa.14719.1.A1_at | --- | --- | 6.42E-06 | -2.18 |
| 1360 | Cfa.10753.1.A1_at | --- | --- | 2.28E-06 | -2.18 |
| 1361 | Cfa.1752.1.S1_at | PDE5A | phosphodiesterase 5A, cGMP-specific | 1.70E-05 | -2.18 |
| 1362 | CfaAffx.27200.1.S1_s_at | --- | --- | 0.000724921 | -2.18 |
| 1363 | Cfa.2852.1.A1_at | --- | --- | 4.12E-05 | -2.18 |
| 1364 | Cfa.797.1.S1_at | LGALS3 | lectin, galactoside-binding, soluble, 3 | 0.00313525 | -2.18 |
| 1365 | Cfa.12651.1.A1_at | TGFBR2 | transforming growth factor, beta receptor II (70/80kDa) | 3.97E-08 | -2.18 |
| 1366 | Cfa.10888.1.A1_at | --- | --- | 0.000316828 | -2.18 |
| 1367 | Cfa.12445.1.A1_at | --- | --- | 9.95E-08 | -2.18 |
| 1368 | CfaAffx.7845.1.S1_s_at | TSC22D1 | TSC22 domain family, member 1 | 7.35E-05 | -2.18 |
| 1369 | Cfa.20690.1.A1_at | --- | --- | 0.000117416 | -2.18 |
| 1370 | Cfa.745.1.S1_at | --- | --- | 4.06E-08 | -2.19 |
| 1371 | Cfa.5596.1.A1_at | --- | --- | 2.07E-08 | -2.19 |
| 1372 | Cfa.2045.1.S1_at | --- | --- | 0.000256346 | -2.19 |
| 1373 | CfaAffx.6180.1.S1_s_at | --- | --- | 0.000290319 | -2.19 |
| 1374 | CfaAffx.5393.1.S1_s_at | ZNF642 | zinc finger protein 642 | 9.18E-06 | -2.19 |
| 1375 | CfaAffx.8584.1.S1_at | ITIH5 | inter-alpha (globulin) inhibitor H5 | 0.00020494 | -2.19 |
| 1376 | Cfa.4475.1.A1_at | --- | --- | 0.00323274 | -2.19 |
| 1377 | Cfa.6273.1.S1_at | SERPINB6 | Serpin peptidase inhibitor, clade B (ovalbumin), member 6 | 1.85E-09 | -2.19 |
| 1378 | Cfa.5012.1.A1_at | --- | --- | 3.62E-08 | -2.19 |
| 1379 | Cfa.15236.1.S1_at | --- | --- | 8.24E-11 | -2.19 |
| 1380 | Cfa.5134.1.A1_s_at | GLIPR1 | GLI pathogenesis-related 1 | 2.18E-05 | -2.19 |
| 1381 | Cfa.4004.1.S1_at | TTLL3 | tubulin tyrosine ligase-like family, member 3 | 1.11E-07 | -2.19 |
| 1382 | CfaAffx.6099.1.S1_s_at | IL4I1 | interleukin 4 induced 1 | 5.46E-10 | -2.19 |
| 1383 | Cfa.3972.1.A1_s_at | SLC22A17 | solute carrier family 22, member 17 | 1.35E-07 | -2.19 |
| 1384 | Cfa.6273.2.A1_at | SERPINB6 | serpin peptidase inhibitor, clade B (ovalbumin), member 6 | 1.26E-09 | -2.19 |
| 1385 | CfaAffx.15613.1.S1_s_at | LRRK2 | leucine-rich repeat kinase 2 | 0.000628987 | -2.20 |
| 1386 | Cfa.9620.1.A1_at | --- | --- | 0.000302685 | -2.20 |
| 1387 | CfaAffx.26690.1.S1_at | KLHL4 | kelch-like 4 (Drosophila) | 9.48E-05 | -2.20 |
| 1388 | CfaAffx.27747.1.S1_at | CDH2 | cadherin 2, type 1, N-cadherin (neuronal) | 9.73E-05 | -2.20 |
| 1389 | Cfa.927.2.A1_a_at | LOC607729 | similar to a disintegrin and metalloprotease domain 3 (cyritestin) | 1.68E-05 | -2.20 |
| 1390 | CfaAffx.28685.1.S1_at | RAB34 | RAB34, member RAS oncogene family | 8.45E-07 | -2.20 |
| 1391 | Cfa.19381.1.S1_s_at | LOC475236 | similar to dynein, cytoplasmic, intermediate polypeptide 1 | 0.000325707 | -2.20 |
| 1392 | CfaAffx.6300.1.S1_s_at | CPT1C | carnitine palmitoyltransferase 1C | 0.000186204 | -2.20 |
| 1393 | CfaAffx.10948.1.S1_at | TMC2 | transmembrane channel-like 2 | 0.00013928 | -2.20 |
| 1394 | CfaAffx.13368.1.S1_s_at | PAG1 | phosphoprotein associated with glycosphingolipid microdomains 1 | 0.000191079 | -2.20 |
| 1395 | CfaAffx.2004.1.S1_s_at | SLC27A6 | solute carrier family 27 (fatty acid transporter), member 6 | 0.00564534 | -2.20 |
| 1396 | Cfa.508.1.S1_at | --- | --- | 0.000431259 | -2.20 |
| 1397 | CfaAffx.11262.1.S1_at | STK33 | serine/threonine kinase 33 | 3.96E-07 | -2.20 |
| 1398 | Cfa.3482.1.S1_at | VNN1 | vanin 1 | 5.96E-07 | -2.20 |
| 1399 | Cfa.227.1.S1_at | AKAP12 | A kinase (PRKA) anchor protein 12 | 0.000481994 | -2.20 |
| 1400 | CfaAffx.20520.1.S1_at | GPR155 | G protein-coupled receptor 155 | 9.35E-08 | -2.20 |
| 1401 | Cfa.89.1.S1_at | TMEM47 | transmembrane protein 47 | 7.15E-07 | -2.20 |
| 1402 | Cfa.20235.1.S1_s_at | FYN | FYN oncogene related to SRC, FGR, YES | 0.000209865 | -2.20 |
| 1403 | CfaAffx.8934.1.S1_s_at | CAMK1 | calcium/calmodulin-dependent protein kinase I | 6.39E-08 | -2.20 |
| 1404 | CfaAffx.30657.1.S1_at | CCBL1 | cysteine conjugate-beta lyase, cytoplasmic | 2.16E-05 | -2.20 |
| 1405 | Cfa.14126.1.A1_at | --- | --- | 0.000183787 | -2.20 |
| 1406 | Cfa.9097.1.A1_at | --- | --- | 0.00119376 | -2.20 |
| 1407 | Cfa.15451.1.A1_at | --- | --- | 0.000154163 | -2.21 |
| 1408 | Cfa.2168.1.A1_at | --- | --- | 7.09E-08 | -2.21 |
| 1409 | CfaAffx.7315.1.S1_s_at | THNSL1 | threonine synthase-like 1 (S. cerevisiae) | 0.00306288 | -2.21 |
| 1410 | CfaAffx.30585.1.S1_s_at | PTGES | prostaglandin E synthase | 0.000116136 | -2.21 |
| 1411 | CfaAffx.19989.1.S1_at | THEM5 | thioesterase superfamily member 5 | 0.00061424 | -2.21 |
| 1412 | Cfa.1373.1.S1_at | --- | --- | 0.000423382 | -2.21 |
| 1413 | Cfa.5261.1.A1_at | --- | --- | 0.00738844 | -2.21 |
| 1414 | Cfa.9384.1.S1_s_at | ARG2 | arginase, type II | 4.88E-06 | -2.21 |
| 1415 | Cfa.15554.1.A1_at | --- | --- | 0.0163482 | -2.21 |
| 1416 | CfaAffx.12364.1.S1_at | SMAD1 | SMAD family member 1 | 4.38E-07 | -2.21 |
| 1417 | Cfa.2778.1.S1_at | CPEB3 | cytoplasmic polyadenylation element binding protein 3 | 6.93E-08 | -2.21 |
| 1418 | Cfa.14333.1.A1_at | --- | --- | 0.0086829 | -2.21 |
| 1419 | CfaAffx.27807.1.S1_at | PAK3 | p21 protein (Cdc42/Rac)-activated kinase 3 | 0.000314429 | -2.21 |
| 1420 | CfaAffx.16822.1.S1_s_at | NAV1 | neuron navigator 1 | 4.22E-05 | -2.21 |
| 1421 | Cfa.18202.2.S1_a_at | FEZ1 | fasciculation and elongation protein zeta 1 (zygin I) | 0.00106136 | -2.21 |
| 1422 | CfaAffx.10717.1.S1_s_at | --- | --- | 7.73E-06 | -2.21 |
| 1423 | Cfa.4647.1.A1_at | --- | --- | 0.000177004 | -2.21 |
| 1424 | Cfa.5303.1.A1_at | --- | --- | 1.08E-07 | -2.21 |
| 1425 | Cfa.6272.1.S1_at | --- | --- | 2.87E-08 | -2.21 |
| 1426 | Cfa.19675.1.S1_at | --- | --- | 2.71E-05 | -2.22 |
| 1427 | Cfa.5885.1.S1_s_at | PAFAH1B3 | platelet-activating factor acetylhydrolase 1b, catalytic subunit 3 (29kDa) | 1.32E-05 | -2.22 |
| 1428 | Cfa.2787.1.A1_at | --- | --- | 2.07E-09 | -2.22 |
| 1429 | CfaAffx.21536.1.S1_at | TM7SF2 | transmembrane 7 superfamily member 2 | 3.38E-06 | -2.22 |
| 1430 | Cfa.17555.1.S1_at | LOC609372 | hypothetical protein LOC609372 | 1.78E-07 | -2.22 |
| 1431 | Cfa.15178.1.A1_at | --- | --- | 7.81E-07 | -2.22 |
| 1432 | CfaAffx.9623.1.S1_at | FOLR1 | folate receptor 1 (adult) | 1.11E-07 | -2.22 |
| 1433 | CfaAffx.18955.1.S1_s_at | ACTA2 /// LOC488984 | actin, alpha 2, smooth muscle, aorta /// similar to Actin, alpha skeletal muscle | 0.00806441 | -2.22 |
| 1434 | Cfa.19264.1.S1_s_at | ABLIM3 | actin binding LIM protein family, member 3 | 2.75E-06 | -2.22 |
| 1435 | Cfa.8939.1.A1_at | --- | --- | 0.00495376 | -2.22 |
| 1436 | Cfa.10786.1.A1_at | PDIA5 | protein disulfide isomerase family A, member 5 | 6.42E-05 | -2.22 |
| 1437 | Cfa.19702.1.S1_at | --- | --- | 0.00751679 | -2.22 |
| 1438 | Cfa.8150.1.A1_at | --- | --- | 0.00147771 | -2.22 |
| 1439 | Cfa.8309.1.A1_at | --- | --- | 1.78E-05 | -2.22 |
| 1440 | Cfa.19947.1.A1_at | --- | --- | 7.81E-06 | -2.22 |
| 1441 | CfaAffx.10594.1.S1_at | APBB1 | amyloid beta (A4) precursor protein-binding, family B, member 1 (Fe65) | 6.01E-11 | -2.22 |
| 1442 | CfaAffx.5981.1.S1_at | TNFSF8 | tumor necrosis factor (ligand) superfamily, member 8 | 0.000861612 | -2.22 |
| 1443 | Cfa.14621.1.S1_a_at | FLNA | filamin A, alpha | 2.16E-09 | -2.22 |
| 1444 | Cfa.14515.1.A1_at | --- | --- | 1.67E-06 | -2.23 |
| 1445 | Cfa.18819.1.S1_at | GBP1 | guanylate binding protein 1, interferon-inducible, 67kDa | 0.00313001 | -2.23 |
| 1446 | Cfa.669.1.A1_at | --- | --- | 1.34E-10 | -2.23 |
| 1447 | CfaAffx.13632.1.S1_at | PRPH | peripherin | 5.08E-09 | -2.23 |
| 1448 | Cfa.8590.1.A1_s_at | ALOX12 | arachidonate 12-lipoxygenase | 0.000146918 | -2.23 |
| 1449 | Cfa.4120.1.S1_at | --- | --- | 1.14E-05 | -2.23 |
| 1450 | Cfa.16922.1.S1_s_at | LOC475615 | similar to mitochondrial tumor suppressor 1 isoform 4 | 2.01E-06 | -2.23 |
| 1451 | Cfa.12436.1.A1_at | PGCP | plasma glutamate carboxypeptidase | 1.96E-05 | -2.23 |
| 1452 | Cfa.14624.1.A1_s_at | --- | --- | 0.000402087 | -2.23 |
| 1453 | CfaAffx.19652.1.S1_s_at | SELENBP1 | selenium binding protein 1 | 4.57E-06 | -2.23 |
| 1454 | Cfa.7505.1.A1_at | TEKT3 | tektin 3 | 0.00082875 | -2.23 |
| 1455 | Cfa.17442.1.S1_at | EMCN | endomucin | 0.000486098 | -2.23 |
| 1456 | CfaAffx.24778.1.S1_at | --- | --- | 3.53E-07 | -2.23 |
| 1457 | CfaAffx.12247.1.S1_s_at | PAM | peptidylglycine alpha-amidating monooxygenase | 2.19E-05 | -2.24 |
| 1458 | Cfa.381.1.A1_at | --- | --- | 4.40E-06 | -2.24 |
| 1459 | Cfa.3077.1.S1_at | --- | --- | 2.70E-07 | -2.24 |
| 1460 | Cfa.9030.1.A1_at | --- | --- | 0.000435051 | -2.24 |
| 1461 | CfaAffx.17316.1.S1_at | LOC607793 | similar to T-cell receptor alpha chain V region PY14 precursor | 7.97E-10 | -2.24 |
| 1462 | CfaAffx.4044.1.S1_s_at | TPM2 | tropomyosin 2 (beta) | 0.000964338 | -2.24 |
| 1463 | Cfa.11291.1.A1_at | LOC611562 | hypothetical protein LOC611562 | 0.000133317 | -2.24 |
| 1464 | Cfa.4505.1.A1_at | CTTN | cortactin | 1.11E-05 | -2.24 |
| 1465 | Cfa.6206.1.A1_a_at | LOC476006 | similar to glutathione S-transferase, pi 1 | 0.00669839 | -2.24 |
| 1466 | Cfa.19492.2.A1_at | --- | --- | 2.35E-11 | -2.24 |
| 1467 | CfaAffx.19768.1.S1_at | MAN1C1 | mannosidase, alpha, class 1C, member 1 | 0.000230459 | -2.24 |
| 1468 | CfaAffx.30944.1.S1_at | GBP5 | guanylate binding protein 5 | 0.00163505 | -2.24 |
| 1469 | CfaAffx.22355.1.S1_at | BCHE | butyrylcholinesterase | 0.00120521 | -2.24 |
| 1470 | Cfa.13446.1.A1_at | TMTC4 | transmembrane and tetratricopeptide repeat containing 4 | 3.11E-05 | -2.24 |
| 1471 | CfaAffx.23712.1.S1_at | LOC610244 | similar to Protein C10orf11 homolog | 0.0018889 | -2.24 |
| 1472 | Cfa.4588.1.A1_at | WIPI1 | WD repeat domain, phosphoinositide interacting 1 | 5.29E-05 | -2.24 |
| 1473 | Cfa.8950.1.A1_at | --- | --- | 0.000103412 | -2.24 |
| 1474 | Cfa.1320.1.A1_at | FXYD6 | FXYD domain containing ion transport regulator 6 | 7.14E-07 | -2.24 |
| 1475 | CfaAffx.6555.1.S1_at | SCML4 | sex comb on midleg-like 4 (Drosophila) | 2.07E-05 | -2.25 |
| 1476 | Cfa.21634.1.S1_at | TSPAN12 | tetraspanin 12 | 0.000147052 | -2.25 |
| 1477 | CfaAffx.16101.1.S1_s_at | TSPAN5 | tetraspanin 5 | 2.32E-07 | -2.25 |
| 1478 | Cfa.11225.2.A1_at | --- | --- | 5.78E-07 | -2.25 |
| 1479 | CfaAffx.3650.1.S1_s_at | TMEM2 | transmembrane protein 2 | 3.03E-05 | -2.25 |
| 1480 | CfaAffx.1500.1.S1_s_at | MSRB3 | methionine sulfoxide reductase B3 | 1.25E-05 | -2.25 |
| 1481 | CfaAffx.7902.1.S1_at | CBLC | Cas-Br-M (murine) ecotropic retroviral transforming sequence c | 4.24E-05 | -2.25 |
| 1482 | Cfa.649.1.S1_s_at | EMP3 | epithelial membrane protein 3 | 9.60E-06 | -2.25 |
| 1483 | Cfa.12798.1.A1_at | --- | --- | 1.04E-06 | -2.25 |
| 1484 | CfaAffx.15606.1.S1_at | LRRK2 | leucine-rich repeat kinase 2 | 0.000454042 | -2.25 |
| 1485 | CfaAffx.4111.1.S1_at | CRISP3 | cysteine-rich secretory protein 3 | 0.0020695 | -2.25 |
| 1486 | Cfa.10255.1.A1_at | MYO1B | myosin IB | 5.51E-11 | -2.25 |
| 1487 | Cfa.1402.1.S1_at | LOC610662 | similar to catenin, alpha-like 1 | 1.20E-08 | -2.25 |
| 1488 | Cfa.9844.1.A1_at | --- | --- | 5.96E-08 | -2.25 |
| 1489 | CfaAffx.27799.1.S1_s_at | ZNF521 | zinc finger protein 521 | 1.70E-06 | -2.25 |
| 1490 | Cfa.11111.1.A1_at | BHMT | betaine--homocysteine S-methyltransferase | 1.18E-05 | -2.25 |
| 1491 | Cfa.9164.1.A1_at | --- | --- | 0.00074829 | -2.25 |
| 1492 | Cfa.14362.1.S1_s_at | LOC477558 | similar to Glutathione S-transferase theta 2 (GST class-theta 2) | 0.000119463 | -2.25 |
| 1493 | CfaAffx.1190.1.S1_s_at | CCDC68 | coiled-coil domain containing 68 | 0.000210868 | -2.25 |
| 1494 | CfaAffx.13739.1.S1_s_at | CXCL13 | chemokine (C-X-C motif) ligand 13 | 0.00991254 | -2.25 |
| 1495 | Cfa.3093.1.A1_at | COL3A1 | collagen, type III, alpha 1 | 0.00387777 | -2.26 |
| 1496 | Cfa.15527.1.A1_at | --- | --- | 0.000113166 | -2.26 |
| 1497 | Cfa.15791.1.A1_at | --- | --- | 0.00190168 | -2.26 |
| 1498 | Cfa.3974.1.A1_at | --- | --- | 5.60E-09 | -2.26 |
| 1499 | CfaAffx.6393.1.S1_s_at | TRIB2 | tribbles homolog 2 (Drosophila) | 0.00137528 | -2.26 |
| 1500 | Cfa.17027.1.S1_at | LRP2 | low density lipoprotein receptor-related protein 2 | 2.69E-06 | -2.26 |
| 1501 | Cfa.5258.1.A1_s_at | SDC2 | syndecan 2 | 8.26E-07 | -2.26 |
| 1502 | CfaAffx.12806.1.S1_at | LOC610426 | similar to R17.3 | 0.000431833 | -2.26 |
| 1503 | CfaAffx.22992.1.S1_at | LOC610489 | similar to Cytochrome P450 27, mitochondrial precursor (Cytochrome P-450C27/25) | 6.87E-05 | -2.26 |
| 1504 | Cfa.3699.1.S1_s_at | AGTR1 | angiotensin II receptor, type 1 | 0.0138988 | -2.26 |
| 1505 | Cfa.13175.1.S1_at | MYCT1 | myc target 1 | 0.000114024 | -2.26 |
| 1506 | Cfa.12548.1.A1_at | --- | --- | 0.00174857 | -2.26 |
| 1507 | Cfa.6331.1.A1_at | PROC | protein C (inactivator of coagulation factors Va and VIIIa) | 0.00119585 | -2.26 |
| 1508 | Cfa.10739.1.A1_s_at | CADM1 | cell adhesion molecule 1 | 0.000107809 | -2.26 |
| 1509 | Cfa.14987.1.A1_at | GIPC1 | GIPC PDZ domain containing family, member 1 | 2.18E-07 | -2.26 |
| 1510 | Cfa.11884.1.A1_at | --- | --- | 0.00175572 | -2.26 |
| 1511 | Cfa.2854.1.S1_at | AGXT2L2 | alanine-glyoxylate aminotransferase 2-like 2 | 1.09E-08 | -2.26 |
| 1512 | Cfa.8942.1.A1_at | --- | --- | 2.62E-06 | -2.26 |
| 1513 | Cfa.3378.1.S1_at | NUPR1 | nuclear protein, transcriptional regulator, 1 | 0.0012897 | -2.26 |
| 1514 | Cfa.15623.1.A1_at | FZD7 | frizzled homolog 7 (Drosophila) | 0.000322089 | -2.26 |
| 1515 | Cfa.12028.1.A1_at | PCSK5 | Proprotein convertase subtilisin/kexin type 5 | 0.00014813 | -2.26 |
| 1516 | CfaAffx.7820.1.S1_at | GIMAP5 | GTPase, IMAP family member 5 | 1.21E-07 | -2.26 |
| 1517 | Cfa.3842.1.S1_at | ICAM1 | intercellular adhesion molecule 1 | 2.89E-06 | -2.26 |
| 1518 | Cfa.354.1.A1_at | --- | --- | 5.36E-06 | -2.26 |
| 1519 | Cfa.2572.2.S1_at | --- | --- | 0.000387412 | -2.27 |
| 1520 | Cfa.2450.1.A1_at | --- | --- | 2.23E-06 | -2.27 |
| 1521 | CfaAffx.22619.1.S1_at | LOC486720 | similar to lymphocyte-activation protein 3 precursor | 0.00253058 | -2.27 |
| 1522 | Cfa.4179.1.A1_at | HTRA1 | HtrA serine peptidase 1 | 2.37E-06 | -2.27 |
| 1523 | Cfa.2961.1.A1_at | --- | --- | 1.29E-07 | -2.27 |
| 1524 | CfaAffx.26728.1.S1_at | LOC478351 | similar to calmodulin-like 4 | 0.000310798 | -2.27 |
| 1525 | Cfa.13192.1.S1_at | MYLK | myosin light chain kinase | 3.56E-06 | -2.27 |
| 1526 | Cfa.5295.1.A1_at | PLP2 | proteolipid protein 2 (colonic epithelium-enriched) | 2.19E-05 | -2.27 |
| 1527 | Cfa.8897.1.A1_at | --- | --- | 2.56E-06 | -2.27 |
| 1528 | Cfa.5952.1.A1_at | --- | --- | 6.22E-07 | -2.27 |
| 1529 | CfaAffx.30080.1.S1_s_at | ELANE | elastase, neutrophil expressed | 0.00173652 | -2.27 |
| 1530 | Cfa.2671.1.S1_at | EPB41L3 | erythrocyte membrane protein band 4.1-like 3 | 3.78E-05 | -2.27 |
| 1531 | Cfa.4589.1.A1_s_at | C1R | complement component 1, r subcomponent | 3.57E-05 | -2.27 |
| 1532 | Cfa.13940.1.A1_at | --- | --- | 5.90E-09 | -2.27 |
| 1533 | CfaAffx.24809.1.S1_s_at | LOC479114 | similar to CG9590-PA | 4.16E-08 | -2.27 |
| 1534 | Cfa.12470.1.A1_at | COPA | coatomer protein complex, subunit alpha | 1.58E-08 | -2.27 |
| 1535 | Cfa.12370.1.A1_at | --- | --- | 1.30E-05 | -2.27 |
| 1536 | Cfa.8262.1.A1_at | GALNT14 | UDP-N-acetyl-alpha-D-galactosamine:polypeptide N-acetylgalactosaminyltransferase | 0.0205717 | -2.27 |
| 1537 | Cfa.12429.1.A1_at | --- | --- | 5.49E-08 | -2.27 |
| 1538 | Cfa.1475.1.S1_at | TGM2 | transglutaminase 2 (C polypeptide, protein-glutamine-gamma-glutamyltransferase) | 0.000238519 | -2.27 |
| 1539 | Cfa.3578.1.S1_at | --- | --- | 3.92E-07 | -2.27 |
| 1540 | CfaAffx.2218.1.S1_s_at | ENPP2 | ectonucleotide pyrophosphatase/phosphodiesterase 2 | 2.15E-07 | -2.27 |
| 1541 | Cfa.15819.1.S1_s_at | --- | --- | 1.18E-07 | -2.27 |
| 1542 | Cfa.5248.1.A1_at | --- | --- | 8.37E-07 | -2.27 |
| 1543 | Cfa.16412.1.A1_at | --- | --- | 0.000343686 | -2.28 |
| 1544 | Cfa.5943.1.A1_at | --- | --- | 0.00132119 | -2.28 |
| 1545 | Cfa.2016.1.A1_at | --- | --- | 1.25E-07 | -2.28 |
| 1546 | Cfa.4679.1.A1_at | DICER1 | dicer 1, ribonuclease type III | 0.000329771 | -2.28 |
| 1547 | CfaAffx.5206.1.S1_at | SIGLEC12 | sialic acid binding Ig-like lectin 12 | 0.000158355 | -2.28 |
| 1548 | Cfa.7025.1.A1_at | PROCA1 | protein interacting with cyclin A1 | 1.04E-08 | -2.28 |
| 1549 | Cfa.13305.1.A1_at | --- | --- | 3.50E-09 | -2.28 |
| 1550 | Cfa.155.1.S1_s_at | RTN1 | reticulon 1 | 0.00871107 | -2.28 |
| 1551 | Cfa.15379.1.A1_at | --- | --- | 0.000212269 | -2.28 |
| 1552 | CfaAffx.28818.1.S1_at | NPR3 | natriuretic peptide receptor C/guanylate cyclase C (atrionatriuretic peptide rec | 0.000120106 | -2.28 |
| 1553 | CfaAffx.30943.1.S1_s_at | GBP5 | guanylate binding protein 5 | 0.000226881 | -2.28 |
| 1554 | Cfa.5423.1.A1_s_at | MARK1 | MAP/microtubule affinity-regulating kinase 1 | 0.00101283 | -2.28 |
| 1555 | Cfa.14378.1.S1_at | DGAT2 | diacylglycerol O-acyltransferase homolog 2 (mouse) | 0.0109349 | -2.28 |
| 1556 | Cfa.12785.1.A1_s_at | --- | --- | 2.72E-05 | -2.29 |
| 1557 | CfaAffx.3671.1.S1_s_at | SPINK4 | serine peptidase inhibitor, Kazal type 4 | 5.82E-06 | -2.29 |
| 1558 | CfaAffx.11821.1.S1_at | DEPDC7 | DEP domain containing 7 | 0.000874524 | -2.29 |
| 1559 | CfaAffx.27815.1.S1_s_at | TTC39C | tetratricopeptide repeat domain 39C | 0.00228788 | -2.29 |
| 1560 | CfaAffx.13353.1.S1_s_at | EPB41L1 | erythrocyte membrane protein band 4.1-like 1 | 1.83E-08 | -2.29 |
| 1561 | CfaAffx.20784.1.S1_at | HOXD10 | homeobox D10 | 1.07E-05 | -2.29 |
| 1562 | Cfa.15214.1.A1_at | --- | --- | 2.85E-06 | -2.29 |
| 1563 | CfaAffx.27193.1.S1_at | ATG4D | ATG4 autophagy related 4 homolog D (S. cerevisiae) | 1.57E-06 | -2.29 |
| 1564 | Cfa.2346.3.A1_at | SKIV2L2 | superkiller viralicidic activity 2-like 2 (S. cerevisiae) | 3.59E-10 | -2.29 |
| 1565 | Cfa.15726.1.A1_at | --- | --- | 4.57E-06 | -2.29 |
| 1566 | Cfa.540.1.A1_at | LHFP | lipoma HMGIC fusion partner | 7.71E-05 | -2.29 |
| 1567 | Cfa.12346.1.A1_at | LIN7A | lin-7 homolog A (C. elegans) | 6.29E-05 | -2.29 |
| 1568 | Cfa.11001.1.A1_at | --- | --- | 1.50E-05 | -2.29 |
| 1569 | Cfa.8341.1.A1_at | --- | --- | 2.11E-07 | -2.29 |
| 1570 | CfaAffx.11223.1.S1_s_at | SLC1A2 | solute carrier family 1 (glial high affinity glutamate transporter), member 2 | 0.000394408 | -2.29 |
| 1571 | CfaAffx.29932.1.S1_s_at | EGFL7 | EGF-like-domain, multiple 7 | 4.38E-08 | -2.29 |
| 1572 | Cfa.12997.1.A1_at | --- | --- | 4.71E-07 | -2.29 |
| 1573 | Cfa.232.1.A1_at | SH3BGRL2 | SH3 domain binding glutamic acid-rich protein like 2 | 0.00646242 | -2.29 |
| 1574 | CfaAffx.7268.1.S1_s_at | DTNB | dystrobrevin, beta | 9.80E-09 | -2.29 |
| 1575 | Cfa.16389.1.A1_s_at | --- | --- | 2.10E-06 | -2.29 |
| 1576 | CfaAffx.5783.1.S1_at | MEIS1 | Meis homeobox 1 | 0.00344276 | -2.29 |
| 1577 | Cfa.63.1.A1_at | --- | --- | 1.71E-08 | -2.29 |
| 1578 | Cfa.19622.1.S1_at | DDAH2 | dimethylarginine dimethylaminohydrolase 2 | 1.87E-05 | -2.29 |
| 1579 | CfaAffx.12364.1.S1_s_at | SMAD1 | SMAD family member 1 | 1.02E-07 | -2.30 |
| 1580 | Cfa.16683.1.A1_at | --- | --- | 0.00145233 | -2.30 |
| 1581 | Cfa.13909.1.A1_at | LAMB1 | laminin, beta 1 | 0.000652411 | -2.30 |
| 1582 | Cfa.10722.1.A1_at | --- | --- | 5.68E-06 | -2.30 |
| 1583 | CfaAffx.20845.1.S1_s_at | CADM1 | cell adhesion molecule 1 | 9.08E-05 | -2.30 |
| 1584 | Cfa.1972.1.A1_at | COL14A1 | collagen, type XIV, alpha 1 | 0.000294634 | -2.30 |
| 1585 | Cfa.19606.1.A1_at | --- | --- | 0.00186335 | -2.30 |
| 1586 | CfaAffx.27826.1.S1_s_at | LAMA3 | laminin, alpha 3 | 3.48E-10 | -2.30 |
| 1587 | Cfa.15462.1.A1_at | --- | --- | 2.20E-06 | -2.30 |
| 1588 | Cfa.21252.1.S1_s_at | ENPP2 | ectonucleotide pyrophosphatase/phosphodiesterase 2 | 3.46E-07 | -2.30 |
| 1589 | Cfa.4664.1.A1_at | --- | --- | 0.00416627 | -2.30 |
| 1590 | Cfa.6371.1.A1_at | NFIL3 | nuclear factor, interleukin 3 regulated | 0.00252016 | -2.30 |
| 1591 | CfaAffx.16558.1.S1_s_at | RGS2 | regulator of G-protein signaling 2, 24kDa | 0.0209708 | -2.30 |
| 1592 | CfaAffx.21120.1.S1_s_at | HMCN1 | hemicentin 1 | 0.000631182 | -2.30 |
| 1593 | Cfa.1516.1.A1_at | --- | --- | 2.42E-09 | -2.30 |
| 1594 | Cfa.15428.1.A1_at | --- | --- | 2.84E-05 | -2.30 |
| 1595 | CfaAffx.17807.1.S1_s_at | CD1E | CD1e molecule | 0.00490787 | -2.30 |
| 1596 | Cfa.17625.1.S1_at | EPAS1 | endothelial PAS domain protein 1 | 6.21E-07 | -2.30 |
| 1597 | Cfa.3699.1.A1_at | AGTR1 | angiotensin II receptor, type 1 | 0.00948988 | -2.30 |
| 1598 | Cfa.16683.1.A1_s_at | --- | --- | 0.0134047 | -2.30 |
| 1599 | CfaAffx.12647.1.S1_at | HPGD | hydroxyprostaglandin dehydrogenase 15-(NAD) | 0.00193341 | -2.30 |
| 1600 | CfaAffx.7442.1.S1_at | PGLYRP1 | peptidoglycan recognition protein 1 | 0.0162157 | -2.30 |
| 1601 | Cfa.7012.1.A1_at | PITPNM2 | phosphatidylinositol transfer protein, membrane-associated 2 | 0.000107981 | -2.30 |
| 1602 | Cfa.12639.1.A1_at | LOC611359 | hypothetical protein LOC611359 | 0.00262764 | -2.31 |
| 1603 | Cfa.13905.1.A1_at | --- | --- | 3.35E-11 | -2.31 |
| 1604 | CfaAffx.20885.1.S1_s_at | DGKG | diacylglycerol kinase, gamma 90kDa | 8.15E-10 | -2.31 |
| 1605 | CfaAffx.17176.1.S1_s_at | CD80 | CD80 molecule | 1.57E-07 | -2.31 |
| 1606 | Cfa.1836.1.A1_at | --- | --- | 7.60E-07 | -2.31 |
| 1607 | Cfa.3997.1.A1_at | HTR7 | 5-hydroxytryptamine (serotonin) receptor 7 (adenylate cyclase-coupled) | 0.00777987 | -2.31 |
| 1608 | Cfa.11036.1.A1_at | --- | --- | 3.79E-08 | -2.31 |
| 1609 | Cfa.11382.1.A1_s_at | ACSF2 | acyl-CoA synthetase family member 2 | 0.000282289 | -2.31 |
| 1610 | Cfa.1991.1.A1_at | --- | --- | 8.71E-11 | -2.31 |
| 1611 | Cfa.3616.1.S1_at | TJP1 | tight junction protein 1 (zona occludens 1) | 2.03E-07 | -2.31 |
| 1612 | Cfa.19597.1.S1_at | --- | --- | 4.24E-07 | -2.31 |
| 1613 | CfaAffx.11483.1.S1_at | RASGEF1A | RasGEF domain family, member 1A | 1.53E-09 | -2.31 |
| 1614 | Cfa.1434.1.A1_at | --- | --- | 5.07E-06 | -2.31 |
| 1615 | Cfa.17236.1.S1_at | FLNB | filamin B, beta | 1.43E-06 | -2.32 |
| 1616 | Cfa.3628.1.S1_s_at | MMP13 | matrix metallopeptidase 13 (collagenase 3) | 0.00531039 | -2.32 |
| 1617 | CfaAffx.14622.1.S1_at | F2R | coagulation factor II (thrombin) receptor | 5.01E-05 | -2.32 |
| 1618 | CfaAffx.13512.1.S1_s_at | GUCY1A3 | guanylate cyclase 1, soluble, alpha 3 | 3.46E-06 | -2.32 |
| 1619 | Cfa.12703.1.A1_s_at | CTSF | cathepsin F | 5.90E-07 | -2.32 |
| 1620 | Cfa.16438.1.S1_at | --- | --- | 0.00151152 | -2.32 |
| 1621 | Cfa.13723.1.A1_at | EPB41L4B | erythrocyte membrane protein band 4.1 like 4B | 2.18E-09 | -2.32 |
| 1622 | Cfa.9644.1.A1_at | --- | --- | 1.36E-07 | -2.32 |
| 1623 | Cfa.14175.1.A1_at | --- | --- | 0.00634426 | -2.32 |
| 1624 | Cfa.11452.1.A1_at | SMTN | smoothelin | 2.48E-06 | -2.32 |
| 1625 | Cfa.2576.1.S1_at | --- | --- | 4.31E-10 | -2.32 |
| 1626 | Cfa.15140.1.A1_s_at | GPR116 | G protein-coupled receptor 116 | 0.00016107 | -2.32 |
| 1627 | Cfa.11630.1.S1_s_at | CNN3 | calponin 3, acidic | 4.00E-07 | -2.32 |
| 1628 | CfaAffx.22170.1.S1_s_at | --- | --- | 5.56E-05 | -2.32 |
| 1629 | Cfa.13115.1.A1_s_at | --- | --- | 0.00118568 | -2.32 |
| 1630 | Cfa.5232.1.A1_at | ACVR1 | activin A receptor, type I | 1.16E-10 | -2.32 |
| 1631 | Cfa.3984.1.A1_at | --- | --- | 9.68E-09 | -2.32 |
| 1632 | CfaAffx.10229.1.S1_at | CYP1B1 | cytochrome P450, family 1, subfamily B, polypeptide 1 | 7.69E-05 | -2.32 |
| 1633 | Cfa.15138.1.A1_at | --- | --- | 0.00301469 | -2.32 |
| 1634 | Cfa.15633.1.A1_at | --- | --- | 1.05E-05 | -2.32 |
| 1635 | Cfa.5284.1.A1_at | PAG1 | phosphoprotein associated with glycosphingolipid microdomains 1 | 3.36E-05 | -2.33 |
| 1636 | CfaAffx.14233.1.S1_s_at | PROS1 | protein S (alpha) | 0.000824936 | -2.33 |
| 1637 | CfaAffx.21433.1.S1_at | RIMKLB | ribosomal modification protein rimK-like family member B | 4.59E-07 | -2.33 |
| 1638 | Cfa.15004.1.A1_s_at | LOC491436 /// LOC611153 | hypothetical LOC491436 /// similar to CG14692-PA | 0.000388086 | -2.33 |
| 1639 | CfaAffx.28431.1.S1_s_at | MYH11 | myosin, heavy chain 11, smooth muscle | 5.11E-07 | -2.33 |
| 1640 | Cfa.8062.1.A1_at | --- | --- | 5.78E-11 | -2.33 |
| 1641 | Cfa.2821.1.A1_at | --- | --- | 0.00169683 | -2.33 |
| 1642 | Cfa.10459.1.A1_at | TTLL12 | Tubulin tyrosine ligase-like family, member 12 | 4.38E-05 | -2.33 |
| 1643 | CfaAffx.23270.1.S1_s_at | LOC611894 | hypothetical protein LOC611894 | 1.49E-06 | -2.33 |
| 1644 | CfaAffx.24065.1.S1_x_at | POMP | proteasome maturation protein | 4.06E-06 | -2.33 |
| 1645 | Cfa.13491.1.A1_s_at | FOLH1 | folate hydrolase (prostate-specific membrane antigen) 1 | 0.0151886 | -2.33 |
| 1646 | CfaAffx.6886.1.S1_at | SLC37A3 | solute carrier family 37 (glycerol-3-phosphate transporter), member 3 | 1.02E-07 | -2.33 |
| 1647 | Cfa.7641.1.S1_at | LOC612202 | hypothetical protein LOC612202 | 4.77E-09 | -2.33 |
| 1648 | Cfa.3649.1.S1_s_at | S100A4 | S100 calcium binding protein A4 | 4.55E-07 | -2.33 |
| 1649 | Cfa.3972.1.A1_at | SLC22A17 | solute carrier family 22, member 17 | 5.32E-08 | -2.33 |
| 1650 | CfaAffx.22116.1.S1_at | --- | --- | 8.78E-07 | -2.33 |
| 1651 | Cfa.12900.1.A1_x_at | RBP7 | retinol binding protein 7, cellular | 0.000164376 | -2.33 |
| 1652 | Cfa.13472.1.A1_at | WDFY3 | WD repeat and FYVE domain containing 3 | 5.21E-07 | -2.33 |
| 1653 | Cfa.19408.1.S1_at | --- | --- | 2.61E-05 | -2.33 |
| 1654 | Cfa.2855.1.A1_at | --- | --- | 2.82E-13 | -2.33 |
| 1655 | CfaAffx.27403.1.S1_at | HS3ST3B1 | heparan sulfate (glucosamine) 3-O-sulfotransferase 3B1 | 0.00649414 | -2.33 |
| 1656 | Cfa.13012.1.A1_at | --- | --- | 3.65E-05 | -2.33 |
| 1657 | CfaAffx.7700.1.S1_s_at | LOC487114 | similar to mannose receptor, C type 1-like 1 | 0.013079 | -2.33 |
| 1658 | Cfa.6458.1.A1_s_at | CFB | complement factor B | 8.05E-08 | -2.33 |
| 1659 | Cfa.17191.1.S1_a_at | LOC479917 | similar to Y73F8A.5 | 4.39E-09 | -2.33 |
| 1660 | Cfa.12684.1.A1_at | --- | --- | 9.03E-09 | -2.33 |
| 1661 | Cfa.317.1.A1_at | CMBL | carboxymethylenebutenolidase homolog (Pseudomonas) | 4.95E-05 | -2.33 |
| 1662 | Cfa.8902.1.A1_at | --- | --- | 0.000429549 | -2.34 |
| 1663 | Cfa.2730.1.S1_at | --- | --- | 0.00110744 | -2.34 |
| 1664 | CfaAffx.3383.1.S1_at | GCNT1 | glucosaminyl (N-acetyl) transferase 1, core 2 | 0.00456853 | -2.34 |
| 1665 | Cfa.9639.1.A1_at | --- | --- | 0.00285278 | -2.34 |
| 1666 | Cfa.942.1.S1_at | CXADR | coxsackie virus and adenovirus receptor | 0.000486363 | -2.34 |
| 1667 | Cfa.15824.1.S1_at | CCL13 | chemokine (C-C motif) ligand 13 | 0.000476502 | -2.34 |
| 1668 | CfaAffx.13535.1.S1_s_at | MEIS2 | Meis homeobox 2 | 0.00359551 | -2.34 |
| 1669 | CfaAffx.13109.1.S1_at | GPR98 | G protein-coupled receptor 98 | 2.19E-08 | -2.34 |
| 1670 | CfaAffx.9344.1.S1_s_at | FARP1 | FERM, RhoGEF (ARHGEF) and pleckstrin domain protein 1 (chondrocyte-derived) | 1.25E-08 | -2.34 |
| 1671 | CfaAffx.51.1.S1_at | --- | --- | 0.013872 | -2.34 |
| 1672 | CfaAffx.13041.1.S1_s_at | FMN1 | formin 1 | 1.56E-06 | -2.34 |
| 1673 | Cfa.9253.1.A1_at | --- | --- | 2.13E-05 | -2.34 |
| 1674 | Cfa.16990.1.S1_at | DYSF | dysferlin, limb girdle muscular dystrophy 2B (autosomal recessive) | 1.18E-07 | -2.34 |
| 1675 | Cfa.10569.1.A1_s_at | FNIP2 | folliculin interacting protein 2 | 3.41E-08 | -2.34 |
| 1676 | Cfa.9351.1.A1_s_at | CAMTA1 | calmodulin binding transcription activator 1 | 0.00388485 | -2.34 |
| 1677 | Cfa.4885.1.A1_at | --- | --- | 3.28E-05 | -2.34 |
| 1678 | CfaAffx.3629.1.S1_s_at | ATP8A1 | ATPase, aminophospholipid transporter (APLT), class I, type 8A, member 1 | 2.03E-07 | -2.34 |
| 1679 | CfaAffx.11012.1.S1_at | --- | --- | 3.60E-09 | -2.34 |
| 1680 | CfaAffx.18023.1.S1_at | ARHGAP6 | Rho GTPase activating protein 6 | 2.44E-09 | -2.34 |
| 1681 | Cfa.17416.1.S1_s_at | HPCAL1 | hippocalcin-like 1 | 8.74E-09 | -2.35 |
| 1682 | Cfa.7527.1.A1_at | --- | --- | 0.000688103 | -2.35 |
| 1683 | Cfa.3473.1.S1_at | CAV2 | caveolin 2 | 0.000485494 | -2.35 |
| 1684 | Cfa.12558.1.A1_s_at | PRKD1 | protein kinase D1 | 1.24E-06 | -2.35 |
| 1685 | Cfa.10141.1.A1_at | --- | --- | 1.05E-09 | -2.35 |
| 1686 | Cfa.5091.1.A1_s_at | DDX25 | DEAD (Asp-Glu-Ala-Asp) box polypeptide 25 | 8.48E-05 | -2.35 |
| 1687 | CfaAffx.15735.1.S1_at | ABCD2 | ATP-binding cassette, sub-family D (ALD), member 2 | 0.00360164 | -2.35 |
| 1688 | Cfa.15678.1.A1_at | --- | --- | 7.92E-05 | -2.35 |
| 1689 | Cfa.15665.1.A1_at | --- | --- | 0.00292083 | -2.35 |
| 1690 | Cfa.14491.1.A1_at | --- | --- | 5.80E-06 | -2.35 |
| 1691 | Cfa.19952.1.S1_at | --- | --- | 0.000997965 | -2.35 |
| 1692 | CfaAffx.20484.1.S1_s_at | PTPRE | protein tyrosine phosphatase, receptor type, E | 7.57E-05 | -2.35 |
| 1693 | CfaAffx.28491.1.S1_at | NOS2 | nitric oxide synthase 2, inducible | 3.16E-05 | -2.35 |
| 1694 | CfaAffx.20841.1.S1_at | LOC480041 | similar to niban protein isoform 2 | 0.00420141 | -2.35 |
| 1695 | Cfa.4612.1.A1_at | --- | --- | 1.97E-11 | -2.35 |
| 1696 | CfaAffx.4026.1.S1_at | GPR110 | G protein-coupled receptor 110 | 0.000999029 | -2.35 |
| 1697 | CfaAffx.5919.1.S1_at | GPR85 | G protein-coupled receptor 85 | 0.000161726 | -2.35 |
| 1698 | Cfa.294.1.A1_at | --- | --- | 5.52E-08 | -2.35 |
| 1699 | Cfa.134.1.S1_s_at | HGFAC | HGF activator | 2.02E-05 | -2.35 |
| 1700 | CfaAffx.4938.1.S1_at | COL12A1 | collagen, type XII, alpha 1 | 4.06E-06 | -2.36 |
| 1701 | CfaAffx.16186.1.S1_at | --- | --- | 1.52E-07 | -2.36 |
| 1702 | CfaAffx.21460.1.S1_at | GGT1 | gamma-glutamyltransferase 1 | 0.000366683 | -2.36 |
| 1703 | Cfa.19619.1.A1_at | FBN2 | fibrillin 2 | 0.0177854 | -2.36 |
| 1704 | Cfa.3738.1.S1_s_at | DMD | dystrophin (muscular dystrophy, Duchenne and Becker types) | 4.67E-07 | -2.36 |
| 1705 | Cfa.6002.1.S1_at | LOC448801 | mastin | 0.00895046 | -2.36 |
| 1706 | Cfa.3657.1.S1_at | PTGER2 | prostaglandin E receptor 2 (subtype EP2), 53kDa | 0.000441376 | -2.36 |
| 1707 | Cfa.12455.1.A1_at | --- | --- | 0.0208976 | -2.36 |
| 1708 | Cfa.9092.1.A1_at | --- | --- | 9.91E-05 | -2.36 |
| 1709 | CfaAffx.22058.1.S1_at | OSBPL1A | oxysterol binding protein-like 1A | 0.00109799 | -2.36 |
| 1710 | Cfa.4498.2.A1_at | --- | --- | 0.000499761 | -2.36 |
| 1711 | Cfa.7678.1.A1_at | IGFBP6 | insulin-like growth factor binding protein 6 | 0.000144068 | -2.36 |
| 1712 | CfaAffx.410.1.S1_s_at | --- | --- | 5.30E-05 | -2.36 |
| 1713 | CfaAffx.22756.1.S1_at | --- | --- | 2.37E-08 | -2.36 |
| 1714 | Cfa.19444.1.S1_at | --- | --- | 1.10E-10 | -2.36 |
| 1715 | Cfa.12246.1.A1_at | MAN1C1 | mannosidase, alpha, class 1C, member 1 | 1.93E-05 | -2.37 |
| 1716 | CfaAffx.6613.1.S1_at | --- | --- | 1.14E-08 | -2.37 |
| 1717 | CfaAffx.20805.1.S1_at | KLRD1 | killer cell lectin-like receptor subfamily D, member 1 | 0.00104915 | -2.37 |
| 1718 | CfaAffx.30029.1.S1_at | CLIC2 | chloride intracellular channel 2 | 2.21E-07 | -2.37 |
| 1719 | Cfa.6877.1.A1_at | SERINC5 | serine incorporator 5 | 0.00130725 | -2.37 |
| 1720 | CfaAffx.11414.1.S1_s_at | CXCL12 | chemokine (C-X-C motif) ligand 12 | 0.000182535 | -2.37 |
| 1721 | CfaAffx.11025.1.S1_at | LOC477200 /// LOC486026 /// LOC608447 | similar to dynein, cytoplasmic, light chain 2A /// similar to dynein, cytoplasmi | 0.00875373 | -2.37 |
| 1722 | CfaAffx.30934.1.S1_s_at | LRRC8C | leucine rich repeat containing 8 family, member C | 2.63E-06 | -2.37 |
| 1723 | Cfa.11563.1.A1_at | CDKN1A | Cyclin-dependent kinase inhibitor 1A (p21, Cip1) | 2.28E-05 | -2.37 |
| 1724 | CfaAffx.17330.1.S1_s_at | LOC607913 | hypothetical protein LOC607913 | 0.00404966 | -2.38 |
| 1725 | Cfa.13399.1.A1_at | --- | --- | 2.72E-06 | -2.38 |
| 1726 | Cfa.11182.1.A1_s_at | RAMP2 | receptor (G protein-coupled) activity modifying protein 2 | 8.84E-07 | -2.38 |
| 1727 | Cfa.3177.1.S1_at | GNAI1 | guanine nucleotide binding protein (G protein), alpha inhibiting activity polype | 4.33E-07 | -2.38 |
| 1728 | Cfa.6582.1.A1_at | --- | --- | 1.27E-07 | -2.38 |
| 1729 | CfaAffx.20085.1.S1_at | --- | --- | 6.72E-05 | -2.38 |
| 1730 | Cfa.621.1.A1_at | --- | --- | 5.82E-09 | -2.38 |
| 1731 | Cfa.15788.3.S1_at | TPM2 | tropomyosin 2 (beta) | 0.000117469 | -2.38 |
| 1732 | CfaAffx.17765.1.S1_s_at | TXNIP | thioredoxin interacting protein | 0.000107687 | -2.38 |
| 1733 | Cfa.5486.1.A1_at | --- | --- | 1.63E-10 | -2.38 |
| 1734 | Cfa.8924.1.A1_at | --- | --- | 0.00783526 | -2.38 |
| 1735 | Cfa.3060.1.A1_at | --- | --- | 2.52E-05 | -2.38 |
| 1736 | Cfa.8075.1.A1_at | SLC9A3R1 | solute carrier family 9 (sodium/hydrogen exchanger), member 3 regulator 1 | 2.93E-06 | -2.38 |
| 1737 | Cfa.1145.1.A2_at | DSTN | destrin (actin depolymerizing factor) | 2.54E-06 | -2.38 |
| 1738 | Cfa.10453.1.A1_at | --- | --- | 2.04E-06 | -2.38 |
| 1739 | Cfa.14015.1.A1_at | RTKN | rhotekin | 3.95E-11 | -2.38 |
| 1740 | Cfa.11357.1.S1_at | CDC42EP3 | CDC42 effector protein (Rho GTPase binding) 3 | 1.13E-07 | -2.38 |
| 1741 | CfaAffx.14726.1.S1_s_at | OLFML3 | olfactomedin-like 3 | 1.91E-05 | -2.38 |
| 1742 | Cfa.19615.1.S1_s_at | NOSTRIN | nitric oxide synthase trafficker | 1.40E-05 | -2.38 |
| 1743 | Cfa.11697.1.A1_at | EPB41L1 | erythrocyte membrane protein band 4.1-like 1 | 1.22E-07 | -2.39 |
| 1744 | Cfa.21609.1.S1_at | LTBR | lymphotoxin beta receptor (TNFR superfamily, member 3) | 4.31E-05 | -2.39 |
| 1745 | CfaAffx.2022.1.S1_at | PKHD1L1 | polycystic kidney and hepatic disease 1 (autosomal recessive)-like 1 | 0.000810147 | -2.39 |
| 1746 | Cfa.20974.1.S1_at | COPZ2 | coatomer protein complex, subunit zeta 2 | 8.42E-05 | -2.39 |
| 1747 | Cfa.14217.1.A1_at | --- | --- | 0.00457921 | -2.39 |
| 1748 | CfaAffx.15408.1.S1_at | PDZRN4 | PDZ domain containing RING finger 4 | 1.51E-09 | -2.39 |
| 1749 | CfaAffx.28016.1.S1_s_at | AFAP1L1 | actin filament associated protein 1-like 1 | 2.00E-07 | -2.39 |
| 1750 | Cfa.3200.1.S1_s_at | --- | --- | 7.49E-06 | -2.39 |
| 1751 | Cfa.10873.1.A1_at | PTPN3 | protein tyrosine phosphatase, non-receptor type 3 | 3.05E-09 | -2.39 |
| 1752 | CfaAffx.21523.1.S1_s_at | SEMA6D | sema domain, transmembrane domain (TM), and cytoplasmic domain, (semaphorin) 6D | 3.08E-05 | -2.39 |
| 1753 | Cfa.11125.1.A1_at | --- | --- | 0.00013626 | -2.39 |
| 1754 | CfaAffx.30061.1.S1_at | CAMTA1 | calmodulin binding transcription activator 1 | 0.00604078 | -2.39 |
| 1755 | Cfa.3221.1.S1_at | --- | --- | 2.43E-06 | -2.39 |
| 1756 | CfaAffx.5057.1.S1_s_at | --- | --- | 0.00142944 | -2.39 |
| 1757 | Cfa.4004.1.S1_s_at | TTLL3 | tubulin tyrosine ligase-like family, member 3 | 1.07E-08 | -2.39 |
| 1758 | CfaAffx.16363.1.S1_at | CD200 | CD200 molecule | 9.46E-07 | -2.40 |
| 1759 | CfaAffx.17716.1.S1_s_at | VWA5A | von Willebrand factor A domain containing 5A | 5.05E-07 | -2.40 |
| 1760 | CfaAffx.1201.1.S1_s_at | RDH16 | retinol dehydrogenase 16 (all-trans) | 3.51E-05 | -2.40 |
| 1761 | CfaAffx.5199.1.S1_s_at | OSBPL3 | oxysterol binding protein-like 3 | 0.00015342 | -2.40 |
| 1762 | CfaAffx.26023.1.S1_at | SGCA | sarcoglycan, alpha (50kDa dystrophin-associated glycoprotein) | 3.30E-06 | -2.40 |
| 1763 | Cfa.20624.1.S1_at | FES | feline sarcoma oncogene | 3.18E-05 | -2.40 |
| 1764 | CfaAffx.15723.1.S1_s_at | --- | --- | 0.00117276 | -2.40 |
| 1765 | Cfa.63.1.S1_at | --- | --- | 1.62E-10 | -2.40 |
| 1766 | CfaAffx.28277.1.S1_x_at | LOC607343 | similar to Ig heavy chain V-III region VH26 precursor | 0.0003595 | -2.40 |
| 1767 | Cfa.5686.1.A1_at | --- | --- | 6.54E-06 | -2.40 |
| 1768 | Cfa.14023.1.A1_at | --- | --- | 2.00E-06 | -2.40 |
| 1769 | Cfa.10329.1.A1_at | --- | --- | 3.80E-07 | -2.40 |
| 1770 | Cfa.9038.1.A1_at | --- | --- | 2.14E-06 | -2.40 |
| 1771 | Cfa.8892.1.A1_at | CHL1 | cell adhesion molecule with homology to L1CAM (close homolog of L1) | 8.42E-05 | -2.40 |
| 1772 | Cfa.692.1.A1_at | --- | --- | 8.65E-07 | -2.41 |
| 1773 | Cfa.15136.1.S1_at | --- | --- | 1.11E-06 | -2.41 |
| 1774 | CfaAffx.17379.1.S1_at | ARSD | arylsulfatase D | 2.00E-09 | -2.41 |
| 1775 | Cfa.11754.1.A1_at | ZNF532 | zinc finger protein 532 | 5.93E-06 | -2.41 |
| 1776 | CfaAffx.23032.1.S1_s_at | LOC610492 /// RAMP2 | similar to Receptor activity-modifying protein 2 precursor (CRLR activity-modify | 2.69E-07 | -2.41 |
| 1777 | Cfa.15053.1.A1_at | --- | --- | 1.34E-06 | -2.41 |
| 1778 | Cfa.14055.1.A1_at | IDO2 | indoleamine 2,3-dioxygenase 2 | 3.09E-09 | -2.41 |
| 1779 | Cfa.12345.1.A1_at | LMCD1 | LIM and cysteine-rich domains 1 | 4.43E-05 | -2.41 |
| 1780 | Cfa.15041.1.A1_at | --- | --- | 8.00E-06 | -2.41 |
| 1781 | Cfa.9684.1.A1_at | LOC487291 | similar to zinc finger protein 423 | 2.64E-07 | -2.41 |
| 1782 | Cfa.10995.1.A1_s_at | WASF1 | WAS protein family, member 1 | 0.00733439 | -2.41 |
| 1783 | Cfa.17029.1.S1_at | SORBS3 | sorbin and SH3 domain containing 3 | 9.15E-11 | -2.41 |
| 1784 | Cfa.9746.1.A1_s_at | HRASLS | HRAS-like suppressor | 0.000492628 | -2.41 |
| 1785 | CfaAffx.5152.1.S1_at | DFNA5 | deafness, autosomal dominant 5 | 2.52E-05 | -2.41 |
| 1786 | CfaAffx.2951.1.S1_at | MAPK13 | mitogen-activated protein kinase 13 | 7.46E-06 | -2.41 |
| 1787 | Cfa.13064.1.A1_at | --- | --- | 1.51E-07 | -2.41 |
| 1788 | CfaAffx.23377.1.S1_s_at | TCEA2 | transcription elongation factor A (SII), 2 | 1.23E-11 | -2.41 |
| 1789 | Cfa.10442.1.A1_at | --- | --- | 6.38E-11 | -2.41 |
| 1790 | Cfa.3046.1.A1_at | --- | --- | 6.91E-09 | -2.41 |
| 1791 | CfaAffx.25046.1.S1_at | PCDH7 | protocadherin 7 | 2.53E-05 | -2.41 |
| 1792 | Cfa.17425.1.S1_at | --- | --- | 1.29E-05 | -2.41 |
| 1793 | CfaAffx.26728.1.S1_s_at | LOC478351 | similar to calmodulin-like 4 | 7.57E-05 | -2.42 |
| 1794 | Cfa.9445.1.A1_at | LOC478351 | similar to calmodulin-like 4 | 5.28E-05 | -2.42 |
| 1795 | Cfa.8872.1.A1_at | --- | --- | 1.37E-08 | -2.42 |
| 1796 | Cfa.8838.1.A1_a_at | --- | --- | 2.51E-05 | -2.42 |
| 1797 | CfaAffx.17307.1.S1_s_at | CD99 | CD99 molecule | 2.85E-06 | -2.42 |
| 1798 | CfaAffx.19128.1.S1_s_at | SYCP2 | synaptonemal complex protein 2 | 0.00200222 | -2.42 |
| 1799 | Cfa.13202.1.S1_x_at | ASL | argininosuccinate lyase | 4.34E-07 | -2.42 |
| 1800 | Cfa.3607.1.S1_s_at | CYP21 | cytochrome P450c21 | 0.00202113 | -2.42 |
| 1801 | Cfa.2283.1.A1_at | --- | --- | 0.00199478 | -2.42 |
| 1802 | Cfa.16261.1.S1_at | LOC609455 | hypothetical protein LOC609455 | 2.27E-09 | -2.42 |
| 1803 | CfaAffx.22014.1.S1_at | SPAG16 | sperm associated antigen 16 | 0.00279565 | -2.42 |
| 1804 | Cfa.9602.1.A1_s_at | --- | --- | 1.97E-06 | -2.42 |
| 1805 | Cfa.10017.1.A1_at | --- | --- | 5.51E-07 | -2.42 |
| 1806 | Cfa.20636.1.S1_at | PPP1R1B | protein phosphatase 1, regulatory (inhibitor) subunit 1B | 0.00180838 | -2.42 |
| 1807 | CfaAffx.25177.1.S1_at | LOC490399 | similar to macrophage scavenger receptor 2 | 0.00108684 | -2.42 |
| 1808 | CfaAffx.314.1.S1_s_at | LOC482753 | similar to T-cell receptor beta chain V region 86T1 precursor | 3.85E-13 | -2.42 |
| 1809 | Cfa.5377.1.A1_s_at | PTK2 | PTK2 protein tyrosine kinase 2 | 3.21E-08 | -2.43 |
| 1810 | CfaAffx.17020.1.S1_s_at | BAI2 | brain-specific angiogenesis inhibitor 2 | 2.22E-06 | -2.43 |
| 1811 | CfaAffx.1247.1.S1_s_at | VNN1 | vanin 1 | 7.36E-08 | -2.43 |
| 1812 | CfaAffx.4293.1.S1_at | --- | --- | 6.15E-07 | -2.43 |
| 1813 | CfaAffx.12741.1.S1_at | PROCR | protein C receptor, endothelial | 8.77E-05 | -2.43 |
| 1814 | Cfa.15593.1.A1_at | --- | --- | 2.56E-06 | -2.43 |
| 1815 | Cfa.12605.1.A1_at | --- | --- | 4.06E-06 | -2.43 |
| 1816 | Cfa.6540.1.A1_at | THEG | Theg homolog (mouse) | 3.88E-06 | -2.43 |
| 1817 | Cfa.13081.1.A1_s_at | SQRDL | sulfide quinone reductase-like (yeast) | 4.90E-06 | -2.43 |
| 1818 | Cfa.3339.1.A1_at | LOC606804 | similar to Sh3 domain YSC-like 1 | 0.00053207 | -2.43 |
| 1819 | CfaAffx.30862.1.S1_at | MTSS1L | metastasis suppressor 1-like | 5.24E-08 | -2.43 |
| 1820 | Cfa.11112.1.A1_at | --- | --- | 0.000702744 | -2.43 |
| 1821 | Cfa.4751.1.A1_at | --- | --- | 4.37E-08 | -2.43 |
| 1822 | CfaAffx.827.1.S1_at | CDC42EP3 | CDC42 effector protein (Rho GTPase binding) 3 | 3.54E-09 | -2.43 |
| 1823 | Cfa.7878.1.A1_at | --- | --- | 1.73E-08 | -2.43 |
| 1824 | Cfa.19092.1.S1_s_at | CNRIP1 | cannabinoid receptor interacting protein 1 | 1.00E-08 | -2.43 |
| 1825 | CfaAffx.9169.1.S1_at | RBMS3 | RNA binding motif, single stranded interacting protein 3 | 3.74E-06 | -2.43 |
| 1826 | Cfa.13544.1.A1_at | TSPAN9 | tetraspanin 9 | 5.49E-06 | -2.43 |
| 1827 | CfaAffx.16295.1.S1_at | TMPRSS7 | transmembrane protease, serine 7 | 4.04E-05 | -2.43 |
| 1828 | Cfa.18965.1.S1_s_at | LCK | lymphocyte-specific protein tyrosine kinase | 8.28E-12 | -2.43 |
| 1829 | CfaAffx.12766.1.S1_at | ROBO2 | roundabout, axon guidance receptor, homolog 2 (Drosophila) | 0.000407973 | -2.43 |
| 1830 | CfaAffx.9556.1.S1_at | LOC482844 | similar to secreted frizzled-related protein 1 | 0.000263153 | -2.43 |
| 1831 | CfaAffx.3189.1.S1_s_at | LOC474709 | similar to Nuclear factor 1 B-type (Nuclear factor 1/B) (NF1-B) (NFI-B) (NF-I/B) | 1.01E-05 | -2.43 |
| 1832 | Cfa.16353.1.S1_at | --- | --- | 2.78E-05 | -2.44 |
| 1833 | CfaAffx.24425.1.S1_at | KRT17 | keratin 17 | 0.000805986 | -2.44 |
| 1834 | Cfa.3470.1.S1_s_at | MMP9 | matrix metallopeptidase 9 (gelatinase B, 92kDa gelatinase, 92kDa type IV collage | 0.00721595 | -2.44 |
| 1835 | CfaAffx.5349.1.S1_x_at | HOXA4 | homeobox A4 | 4.19E-08 | -2.44 |
| 1836 | CfaAffx.8275.1.S1_at | TMEM37 | transmembrane protein 37 | 3.70E-06 | -2.44 |
| 1837 | CfaAffx.4100.1.S1_at | IL18RAP | interleukin 18 receptor accessory protein | 0.00175108 | -2.44 |
| 1838 | CfaAffx.18111.1.S1_s_at | MTMR11 | myotubularin related protein 11 | 4.77E-06 | -2.44 |
| 1839 | Cfa.10333.1.A1_s_at | ACSS3 | acyl-CoA synthetase short-chain family member 3 | 4.87E-07 | -2.44 |
| 1840 | Cfa.19690.1.S1_at | --- | --- | 6.75E-11 | -2.44 |
| 1841 | Cfa.1946.1.A1_at | HMCN1 | hemicentin 1 | 0.00216569 | -2.44 |
| 1842 | CfaAffx.28804.1.S1_s_at | GPC3 | glypican 3 | 0.001216 | -2.44 |
| 1843 | CfaAffx.8426.1.S1_s_at | NCKAP5 | NCK-associated protein 5 | 1.74E-08 | -2.44 |
| 1844 | Cfa.19391.1.S1_at | EGFL7 | EGF-like-domain, multiple 7 | 1.56E-10 | -2.44 |
| 1845 | CfaAffx.30147.1.S1_s_at | CA6 | carbonic anhydrase VI | 0.000220299 | -2.45 |
| 1846 | Cfa.2338.1.A1_at | --- | --- | 9.39E-07 | -2.45 |
| 1847 | CfaAffx.3629.1.S1_at | ATP8A1 | ATPase, aminophospholipid transporter (APLT), class I, type 8A, member 1 | 1.42E-07 | -2.45 |
| 1848 | Cfa.14779.1.S1_at | LOC474709 | similar to Nuclear factor 1 B-type (Nuclear factor 1/B) (NF1-B) (NFI-B) (NF-I/B) | 5.00E-07 | -2.45 |
| 1849 | Cfa.7294.1.A1_at | --- | --- | 4.89E-07 | -2.45 |
| 1850 | Cfa.9040.1.A1_at | FLRT2 | fibronectin leucine rich transmembrane protein 2 | 8.56E-08 | -2.45 |
| 1851 | Cfa.1893.1.A1_at | LOC474698 | similar to catenin (cadherin-associated protein), alpha 1, 102kDa | 1.74E-07 | -2.45 |
| 1852 | Cfa.13929.1.A1_at | --- | --- | 3.07E-11 | -2.45 |
| 1853 | Cfa.8885.1.A1_at | --- | --- | 0.00147188 | -2.45 |
| 1854 | CfaAffx.22232.1.S1_s_at | NCKAP1 | NCK-associated protein 1 | 3.83E-06 | -2.45 |
| 1855 | CfaAffx.8413.1.S1_at | SYN2 | synapsin II | 8.25E-11 | -2.45 |
| 1856 | CfaAffx.17850.1.S1_at | PITPNC1 | phosphatidylinositol transfer protein, cytoplasmic 1 | 0.000819759 | -2.45 |
| 1857 | Cfa.14908.1.A1_at | FAH | fumarylacetoacetate hydrolase (fumarylacetoacetase) | 3.11E-06 | -2.45 |
| 1858 | Cfa.2838.1.A1_at | --- | --- | 7.63E-05 | -2.45 |
| 1859 | Cfa.6227.1.A1_at | SOAT2 | sterol O-acyltransferase 2 | 2.90E-10 | -2.45 |
| 1860 | Cfa.10785.1.A1_at | --- | --- | 3.33E-08 | -2.46 |
| 1861 | Cfa.10817.1.A1_at | --- | --- | 5.02E-05 | -2.46 |
| 1862 | Cfa.11592.1.A1_at | --- | --- | 1.51E-09 | -2.46 |
| 1863 | Cfa.330.1.S1_at | --- | --- | 4.85E-07 | -2.46 |
| 1864 | Cfa.13754.1.A1_at | --- | --- | 6.88E-07 | -2.46 |
| 1865 | CfaAffx.4323.1.S1_s_at | ECM2 | extracellular matrix protein 2, female organ and adipocyte specific | 2.31E-05 | -2.46 |
| 1866 | CfaAffx.6847.1.S1_at | SPATA6 | spermatogenesis associated 6 | 3.14E-07 | -2.46 |
| 1867 | Cfa.5110.1.A1_at | --- | --- | 4.12E-06 | -2.46 |
| 1868 | Cfa.15744.1.A1_at | ACVRL1 | activin A receptor type II-like 1 | 1.38E-11 | -2.46 |
| 1869 | Cfa.6156.1.A1_at | --- | --- | 3.75E-07 | -2.46 |
| 1870 | Cfa.3849.1.S1_s_at | HSPB1 | heat shock 27kDa protein 1 | 9.72E-07 | -2.47 |
| 1871 | Cfa.524.1.S1_at | --- | --- | 1.74E-07 | -2.47 |
| 1872 | Cfa.12876.1.S1_at | --- | --- | 0.00021312 | -2.47 |
| 1873 | Cfa.11162.1.A1_s_at | PPP2R2B | protein phosphatase 2, regulatory subunit B, beta | 1.63E-05 | -2.47 |
| 1874 | Cfa.357.1.A1_at | --- | --- | 0.000196747 | -2.47 |
| 1875 | Cfa.7314.1.A1_at | --- | --- | 6.54E-05 | -2.47 |
| 1876 | Cfa.15785.1.A1_at | --- | --- | 0.000709258 | -2.47 |
| 1877 | Cfa.697.1.A1_at | --- | --- | 0.000962581 | -2.47 |
| 1878 | Cfa.5689.1.A1_at | --- | --- | 1.38E-06 | -2.47 |
| 1879 | Cfa.9712.1.A1_at | --- | --- | 8.06E-07 | -2.47 |
| 1880 | Cfa.10048.1.A1_at | --- | --- | 6.40E-07 | -2.47 |
| 1881 | Cfa.10320.1.A1_at | --- | --- | 4.19E-10 | -2.47 |
| 1882 | Cfa.1443.1.S1_s_at | SCHIP1 | schwannomin interacting protein 1 | 4.25E-07 | -2.47 |
| 1883 | Cfa.7652.1.A1_s_at | PPFIA2 | protein tyrosine phosphatase, receptor type, f polypeptide (PTPRF), interacting | 1.00E-07 | -2.47 |
| 1884 | Cfa.13769.1.A1_at | --- | --- | 3.44E-05 | -2.48 |
| 1885 | Cfa.15764.1.A1_at | --- | --- | 4.70E-08 | -2.48 |
| 1886 | Cfa.14019.1.S1_at | --- | --- | 3.40E-05 | -2.48 |
| 1887 | Cfa.10426.1.A1_at | --- | --- | 1.57E-06 | -2.48 |
| 1888 | Cfa.3911.1.S1_s_at | IGF1R | insulin-like growth factor 1 receptor | 4.73E-06 | -2.48 |
| 1889 | CfaAffx.6485.1.S1_s_at | PRDM1 | PR domain containing 1, with ZNF domain | 0.000305035 | -2.48 |
| 1890 | Cfa.9547.3.A1_at | PLK1S1 | polo-like kinase 1 substrate 1 | 3.46E-05 | -2.48 |
| 1891 | Cfa.12195.3.A1_at | FHL1 | Four and a half LIM domains 1 | 0.00183829 | -2.48 |
| 1892 | Cfa.12034.1.A1_at | --- | --- | 4.55E-06 | -2.48 |
| 1893 | CfaAffx.7837.1.S1_at | PTER | phosphotriesterase related | 2.58E-06 | -2.48 |
| 1894 | Cfa.8528.1.A1_at | LOC611704 | hypothetical protein LOC611704 | 0.00307472 | -2.48 |
| 1895 | CfaAffx.14998.1.S1_at | GCNT4 | glucosaminyl (N-acetyl) transferase 4, core 2 | 1.55E-10 | -2.48 |
| 1896 | Cfa.858.1.A1_s_at | PDGFRL | platelet-derived growth factor receptor-like | 6.82E-05 | -2.48 |
| 1897 | Cfa.12433.1.A1_at | CD99 | CD99 molecule | 6.59E-07 | -2.48 |
| 1898 | CfaAffx.2790.1.S1_s_at | EGR1 | early growth response 1 | 0.00224036 | -2.49 |
| 1899 | Cfa.10203.1.A1_at | --- | --- | 0.00294864 | -2.49 |
| 1900 | Cfa.19635.1.S1_at | --- | --- | 1.99E-05 | -2.49 |
| 1901 | Cfa.3169.1.A1_at | --- | --- | 0.00546369 | -2.49 |
| 1902 | Cfa.12906.1.A1_s_at | SLC39A8 | solute carrier family 39 (zinc transporter), member 8 | 0.0118622 | -2.49 |
| 1903 | Cfa.5258.1.A1_at | SDC2 | syndecan 2 | 1.56E-06 | -2.49 |
| 1904 | Cfa.12391.1.A1_s_at | RBPMS | RNA binding protein with multiple splicing | 3.91E-06 | -2.49 |
| 1905 | Cfa.8493.1.A1_at | --- | --- | 4.01E-14 | -2.49 |
| 1906 | Cfa.16361.1.S1_at | --- | --- | 6.27E-05 | -2.49 |
| 1907 | Cfa.12528.1.A1_at | COL1A2 | collagen, type I, alpha 2 | 2.91E-05 | -2.49 |
| 1908 | Cfa.11267.1.A1_at | --- | --- | 5.76E-05 | -2.49 |
| 1909 | Cfa.5322.1.S1_at | LOC608636 | hypothetical protein LOC608636 | 9.79E-09 | -2.49 |
| 1910 | Cfa.14628.1.A1_at | --- | --- | 5.46E-06 | -2.49 |
| 1911 | CfaAffx.1775.1.S1_s_at | MIOX | myo-inositol oxygenase | 1.42E-05 | -2.49 |
| 1912 | CfaAffx.2526.1.S1_s_at | PTPRK | protein tyrosine phosphatase, receptor type, K | 1.73E-07 | -2.49 |
| 1913 | Cfa.6381.1.A1_at | LPHN2 | latrophilin 2 | 2.24E-05 | -2.50 |
| 1914 | Cfa.1791.1.A1_s_at | Sep-04 | septin 4 | 6.86E-07 | -2.50 |
| 1915 | Cfa.11134.1.A1_at | SERPINB1 | serpin peptidase inhibitor, clade B (ovalbumin), member 1 | 2.43E-06 | -2.50 |
| 1916 | Cfa.3849.1.S1_at | HSPB1 | heat shock 27kDa protein 1 | 4.33E-06 | -2.50 |
| 1917 | Cfa.11054.1.A1_at | --- | --- | 4.33E-07 | -2.50 |
| 1918 | CfaAffx.21823.1.S1_at | VPREB1 | pre-B lymphocyte 1 | 9.98E-05 | -2.50 |
| 1919 | Cfa.13977.1.A1_at | --- | --- | 3.16E-06 | -2.50 |
| 1920 | CfaAffx.15213.1.S1_s_at | DBNDD2 | dysbindin (dystrobrevin binding protein 1) domain containing 2 | 1.02E-09 | -2.50 |
| 1921 | Cfa.14552.1.A1_at | --- | --- | 4.47E-12 | -2.50 |
| 1922 | CfaAffx.13048.1.S1_at | --- | --- | 0.00528013 | -2.51 |
| 1923 | Cfa.16521.1.S1_s_at | VWA3B | von Willebrand factor A domain containing 3B | 8.19E-08 | -2.51 |
| 1924 | Cfa.17217.1.S1_s_at | ME3 | malic enzyme 3, NADP(+)-dependent, mitochondrial | 1.23E-06 | -2.51 |
| 1925 | Cfa.7499.1.S1_at | GCOM1 | GRINL1A complex locus | 4.62E-06 | -2.51 |
| 1926 | CfaAffx.539.1.S1_x_at | LOC490893 /// LOC490894 /// LOC606869 /// LOC606881 /// LOC606916 /// LOC606928 /// LOC606943 /// LOC607010 /// LOC607079 /// LOC607113 /// LOC607124 /// LOC607179 /// LOC607467 /// LOC607479 /// LOC607491 /// LOC607553 | similar to Ig heavy chain V-III region VH26 precursor /// similar to Ig heavy ch | 0.0057667 | -2.51 |
| 1927 | CfaAffx.14964.1.S1_s_at | F13A1 | coagulation factor XIII, A1 polypeptide | 0.00632769 | -2.51 |
| 1928 | CfaAffx.5853.1.S1_at | --- | --- | 4.98E-08 | -2.51 |
| 1929 | CfaAffx.2190.1.S1_at | SLC22A4 | solute carrier family 22 (organic cation/ergothioneine transporter), member 4 | 0.000921245 | -2.51 |
| 1930 | CfaAffx.8900.1.S1_at | LBH | limb bud and heart development homolog (mouse) | 3.81E-11 | -2.51 |
| 1931 | CfaAffx.28832.1.S1_at | PIPOX | pipecolic acid oxidase | 0.000170808 | -2.51 |
| 1932 | Cfa.12306.1.A1_at | --- | --- | 9.49E-09 | -2.51 |
| 1933 | Cfa.8751.1.A1_at | MAP1LC3A | microtubule-associated protein 1 light chain 3 alpha | 2.18E-09 | -2.51 |
| 1934 | CfaAffx.23488.1.S1_s_at | PROM1 | prominin 1 | 1.29E-05 | -2.51 |
| 1935 | Cfa.7985.1.A1_at | --- | --- | 2.24E-10 | -2.51 |
| 1936 | CfaAffx.24337.1.S1_s_at | UCHL1 | ubiquitin carboxyl-terminal esterase L1 (ubiquitin thiolesterase) | 5.82E-05 | -2.51 |
| 1937 | Cfa.7236.1.A1_s_at | NRXN1 | neurexin 1 | 0.000284064 | -2.51 |
| 1938 | Cfa.12326.1.A1_at | --- | --- | 1.09E-05 | -2.51 |
| 1939 | Cfa.13421.1.A1_at | --- | --- | 9.66E-08 | -2.51 |
| 1940 | Cfa.9469.1.A1_at | --- | --- | 2.94E-09 | -2.52 |
| 1941 | CfaAffx.505.1.S1_at | --- | --- | 2.45E-08 | -2.52 |
| 1942 | Cfa.12195.3.S1_at | FHL1 | four and a half LIM domains 1 | 0.00197656 | -2.52 |
| 1943 | Cfa.14639.1.S1_at | SLC11A1 | solute carrier family 11 (proton-coupled divalent metal ion transporters), membe | 0.0179849 | -2.52 |
| 1944 | CfaAffx.18147.1.S1_at | OTUD7B | OTU domain containing 7B | 1.60E-06 | -2.52 |
| 1945 | CfaAffx.23296.1.S1_at | VAMP1 | vesicle-associated membrane protein 1 (synaptobrevin 1) | 6.59E-09 | -2.52 |
| 1946 | CfaAffx.6375.1.S1_at | --- | --- | 4.81E-10 | -2.52 |
| 1947 | Cfa.13059.1.A1_s_at | COL14A1 | Collagen, type XIV, alpha 1 | 0.000226746 | -2.52 |
| 1948 | Cfa.5515.1.S1_at | --- | --- | 2.55E-06 | -2.52 |
| 1949 | Cfa.6219.1.A1_at | TMEM54 | transmembrane protein 54 | 2.79E-06 | -2.52 |
| 1950 | Cfa.7234.1.A1_at | --- | --- | 0.00772196 | -2.52 |
| 1951 | Cfa.10469.1.A1_at | LOC609688 | hypothetical protein LOC609688 | 1.35E-05 | -2.53 |
| 1952 | Cfa.78.1.S1_s_at | SLC15A1 | solute carrier family 15 (oligopeptide transporter), member 1 | 0.00777177 | -2.53 |
| 1953 | Cfa.2906.1.A1_at | --- | --- | 0.000463923 | -2.53 |
| 1954 | Cfa.19323.1.S1_at | --- | --- | 0.000208138 | -2.53 |
| 1955 | Cfa.14068.1.A1_at | NR1H4 | nuclear receptor subfamily 1, group H, member 4 | 2.15E-06 | -2.53 |
| 1956 | Cfa.14925.1.A1_s_at | AMDHD1 | amidohydrolase domain containing 1 | 1.32E-05 | -2.53 |
| 1957 | Cfa.17306.1.S1_s_at | TMEM2 | transmembrane protein 2 | 5.30E-05 | -2.53 |
| 1958 | Cfa.10303.1.S1_at | FGL2 | fibrinogen-like 2 | 0.000626208 | -2.53 |
| 1959 | Cfa.4145.1.A1_at | --- | --- | 5.45E-06 | -2.53 |
| 1960 | Cfa.16994.1.S1_at | --- | --- | 7.14E-08 | -2.53 |
| 1961 | CfaAffx.10875.1.S1_at | CPXM1 | carboxypeptidase X (M14 family), member 1 | 0.000180006 | -2.53 |
| 1962 | Cfa.19432.1.S1_at | --- | --- | 4.72E-08 | -2.53 |
| 1963 | Cfa.10819.1.A1_at | TBC1D8 | TBC1 domain family, member 8 (with GRAM domain) | 0.00230986 | -2.53 |
| 1964 | CfaAffx.14701.1.S1_s_at | STAB1 | stabilin 1 | 0.000288792 | -2.53 |
| 1965 | Cfa.2942.1.S1_at | --- | --- | 1.30E-06 | -2.53 |
| 1966 | Cfa.2219.1.A1_at | ITGA6 | integrin, alpha 6 | 6.78E-06 | -2.53 |
| 1967 | Cfa.2863.1.A1_at | CLCN4 | chloride channel 4 | 8.89E-10 | -2.53 |
| 1968 | Cfa.13969.1.A1_at | CADPS2 | Ca++-dependent secretion activator 2 | 9.38E-06 | -2.53 |
| 1969 | CfaAffx.12207.1.S1_s_at | MAP1LC3A | microtubule-associated protein 1 light chain 3 alpha | 1.21E-07 | -2.53 |
| 1970 | Cfa.19699.1.S1_at | JAG1 | jagged 1 (Alagille syndrome) | 1.03E-08 | -2.54 |
| 1971 | Cfa.14833.1.A1_at | --- | --- | 0.000398875 | -2.54 |
| 1972 | Cfa.14528.1.A1_at | DLA-79 | MHC class Ib | 0.000155273 | -2.54 |
| 1973 | Cfa.13419.1.A1_at | --- | --- | 2.28E-09 | -2.54 |
| 1974 | Cfa.12205.1.A1_at | --- | --- | 0.000111154 | -2.54 |
| 1975 | Cfa.13966.1.A1_at | --- | --- | 3.73E-07 | -2.54 |
| 1976 | CfaAffx.16663.1.S1_at | SLC16A14 | solute carrier family 16, member 14 (monocarboxylic acid transporter 14) | 9.84E-07 | -2.54 |
| 1977 | Cfa.15522.1.A1_at | --- | --- | 1.01E-07 | -2.54 |
| 1978 | CfaAffx.8901.1.S1_s_at | TIE1 | tyrosine kinase with immunoglobulin-like and EGF-like domains 1 | 6.21E-07 | -2.54 |
| 1979 | Cfa.9156.1.A1_at | CAMTA1 | calmodulin binding transcription activator 1 | 0.000871424 | -2.55 |
| 1980 | CfaAffx.19461.1.S1_at | PRKD1 | protein kinase D1 | 3.58E-08 | -2.55 |
| 1981 | Cfa.5636.1.A1_at | --- | --- | 5.71E-12 | -2.55 |
| 1982 | CfaAffx.18129.1.S1_s_at | AOH3 | aldehyde oxidase 3 | 0.000611412 | -2.55 |
| 1983 | Cfa.11197.1.A1_s_at | SCG3 | secretogranin III | 4.56E-06 | -2.55 |
| 1984 | Cfa.698.5.A1_at | --- | --- | 6.26E-09 | -2.55 |
| 1985 | Cfa.11051.1.A1_at | CACNA2D3 | calcium channel, voltage-dependent, alpha 2/delta subunit 3 | 6.15E-10 | -2.55 |
| 1986 | Cfa.6273.1.S1_s_at | SERPINB6 | serpin peptidase inhibitor, clade B (ovalbumin), member 6 | 9.97E-11 | -2.55 |
| 1987 | Cfa.12144.1.A1_at | ERRFI1 | ERBB receptor feedback inhibitor 1 | 3.73E-05 | -2.55 |
| 1988 | Cfa.11182.1.A1_at | RAMP2 | receptor (G protein-coupled) activity modifying protein 2 | 2.24E-08 | -2.55 |
| 1989 | CfaAffx.18574.1.S1_s_at | ARHGEF12 | Rho guanine nucleotide exchange factor (GEF) 12 | 1.70E-08 | -2.55 |
| 1990 | Cfa.7466.2.A1_a_at | NMS | neuromedin S | 2.15E-05 | -2.56 |
| 1991 | Cfa.5104.1.A1_at | --- | --- | 0.000301232 | -2.56 |
| 1992 | CfaAffx.8579.1.S1_at | CXCR4 | chemokine (C-X-C motif) receptor 4 | 0.00124536 | -2.56 |
| 1993 | Cfa.2124.1.A1_at | --- | --- | 3.94E-08 | -2.56 |
| 1994 | CfaAffx.13191.1.S1_s_at | --- | --- | 0.000802656 | -2.56 |
| 1995 | Cfa.2913.1.A1_at | --- | --- | 0.000175471 | -2.56 |
| 1996 | CfaAffx.7431.1.S1_at | HIF3A | hypoxia inducible factor 3, alpha subunit | 0.00215033 | -2.56 |
| 1997 | CfaAffx.13075.1.S1_at | DLA-79 | MHC class Ib | 0.000313571 | -2.56 |
| 1998 | Cfa.6782.1.A1_at | --- | --- | 3.41E-05 | -2.56 |
| 1999 | CfaAffx.7365.1.S1_at | CST9L | cystatin 9-like | 0.00459808 | -2.56 |
| 2000 | Cfa.9543.1.A1_at | --- | --- | 1.60E-06 | -2.56 |
| 2001 | Cfa.20867.1.S1_s_at | PIPOX | pipecolic acid oxidase | 5.77E-05 | -2.56 |
| 2002 | Cfa.10785.1.A1_s_at | --- | --- | 3.84E-07 | -2.56 |
| 2003 | CfaAffx.9377.1.S1_s_at | SLC4A7 | solute carrier family 4, sodium bicarbonate cotransporter, member 7 | 2.62E-08 | -2.56 |
| 2004 | Cfa.1238.3.A1_at | GSN | gelsolin | 0.000210201 | -2.56 |
| 2005 | Cfa.5456.1.A1_at | --- | --- | 0.000154404 | -2.56 |
| 2006 | Cfa.13202.1.S1_s_at | ASL | argininosuccinate lyase | 1.69E-07 | -2.56 |
| 2007 | Cfa.3966.1.S1_at | --- | --- | 1.59E-07 | -2.56 |
| 2008 | Cfa.90.1.A1_at | PPL | periplakin | 0.00440518 | -2.56 |
| 2009 | CfaAffx.4515.1.S1_at | --- | --- | 2.02E-06 | -2.57 |
| 2010 | CfaAffx.22286.1.S1_at | MAOA | monoamine oxidase A | 0.00224375 | -2.57 |
| 2011 | Cfa.14395.1.A1_s_at | PTER | phosphotriesterase related | 1.51E-05 | -2.57 |
| 2012 | Cfa.20654.1.A1_at | --- | --- | 0.000275176 | -2.57 |
| 2013 | Cfa.5543.1.A1_at | --- | --- | 3.50E-05 | -2.57 |
| 2014 | Cfa.2601.1.S1_at | ACPL2 | acid phosphatase-like 2 | 5.19E-05 | -2.57 |
| 2015 | Cfa.4926.1.A1_s_at | PLA2G16 | phospholipase A2, group XVI | 6.27E-05 | -2.57 |
| 2016 | Cfa.2320.1.A1_at | --- | --- | 9.70E-05 | -2.57 |
| 2017 | CfaAffx.19933.1.S1_s_at | PDE6H | phosphodiesterase 6H, cGMP-specific, cone, gamma | 5.14E-06 | -2.57 |
| 2018 | Cfa.6155.1.A1_at | --- | --- | 2.42E-09 | -2.57 |
| 2019 | CfaAffx.23588.1.S1_s_at | CC2D2A | coiled-coil and C2 domain containing 2A | 1.63E-05 | -2.57 |
| 2020 | Cfa.3806.1.S1_s_at | CALCA | calcitonin/calcitonin-related polypeptide, alpha | 1.96E-06 | -2.57 |
| 2021 | Cfa.9991.1.A1_at | --- | --- | 4.79E-07 | -2.57 |
| 2022 | Cfa.14251.1.A1_at | TTC12 | tetratricopeptide repeat domain 12 | 6.13E-09 | -2.57 |
| 2023 | Cfa.12385.1.A1_at | LOC607395 | hypothetical protein LOC607395 | 4.28E-05 | -2.57 |
| 2024 | CfaAffx.15143.1.S1_at | CBR3 | carbonyl reductase 3 | 3.68E-08 | -2.57 |
| 2025 | Cfa.14436.1.A1_at | --- | --- | 2.65E-05 | -2.58 |
| 2026 | CfaAffx.15444.1.S1_s_at | NKD1 | naked cuticle homolog 1 (Drosophila) | 2.57E-07 | -2.58 |
| 2027 | CfaAffx.14682.1.S1_s_at | SERPINB6 | serpin peptidase inhibitor, clade B (ovalbumin), member 6 | 4.31E-13 | -2.58 |
| 2028 | Cfa.16922.2.S1_a_at | LOC475615 | similar to mitochondrial tumor suppressor 1 isoform 4 | 1.95E-08 | -2.58 |
| 2029 | Cfa.9992.1.A1_at | --- | --- | 1.17E-07 | -2.58 |
| 2030 | CfaAffx.28534.1.S1_s_at | SMARCA1 | SWI/SNF related, matrix associated, actin dependent regulator of chromatin, subf | 5.31E-09 | -2.58 |
| 2031 | Cfa.213.2.S1_a_at | TNNT2 | troponin T type 2 (cardiac) | 2.00E-09 | -2.58 |
| 2032 | Cfa.5201.1.A1_at | --- | --- | 3.84E-07 | -2.58 |
| 2033 | Cfa.12527.1.A1_at | --- | --- | 0.000952449 | -2.58 |
| 2034 | Cfa.7298.1.A1_a_at | PPT2 | palmitoyl-protein thioesterase 2 | 5.27E-09 | -2.58 |
| 2035 | Cfa.12834.1.A1_at | --- | --- | 3.16E-07 | -2.58 |
| 2036 | Cfa.1904.1.A1_at | --- | --- | 0.000365483 | -2.58 |
| 2037 | CfaAffx.28801.1.S1_s_at | GPC4 | glypican 4 | 6.56E-08 | -2.58 |
| 2038 | Cfa.14297.1.A1_s_at | MAOB | monoamine oxidase B | 0.00945283 | -2.58 |
| 2039 | Cfa.5181.1.A1_at | --- | --- | 4.94E-06 | -2.59 |
| 2040 | Cfa.12543.1.A1_s_at | NPR2 | natriuretic peptide receptor B/guanylate cyclase B (atrionatriuretic peptide rec | 1.69E-09 | -2.59 |
| 2041 | Cfa.3705.1.S1_at | COL4A3 | collagen, type IV, alpha 3 (Goodpasture antigen) | 2.41E-05 | -2.59 |
| 2042 | CfaAffx.4494.1.S1_s_at | ZNF550 | zinc finger protein 550 | 1.25E-06 | -2.59 |
| 2043 | CfaAffx.4062.1.S1_at | LOC611190 | similar to esophageal cancer related gene 4 protein | 0.00543763 | -2.59 |
| 2044 | Cfa.14101.1.A1_at | --- | --- | 0.0161546 | -2.59 |
| 2045 | Cfa.6011.1.A1_at | C1QTNF7 | C1q and tumor necrosis factor related protein 7 | 3.26E-06 | -2.59 |
| 2046 | Cfa.2509.1.S1_at | FABP5 | fatty acid binding protein 5 (psoriasis-associated) | 6.55E-06 | -2.60 |
| 2047 | Cfa.5733.1.A1_at | --- | --- | 1.33E-08 | -2.60 |
| 2048 | Cfa.11275.2.A1_s_at | PID1 | phosphotyrosine interaction domain containing 1 | 0.00663481 | -2.60 |
| 2049 | Cfa.14863.1.A1_at | --- | --- | 2.26E-06 | -2.60 |
| 2050 | Cfa.4859.1.A1_at | --- | --- | 4.18E-08 | -2.60 |
| 2051 | CfaAffx.3699.1.S1_s_at | STEAP4 | STEAP family member 4 | 1.60E-07 | -2.60 |
| 2052 | Cfa.1721.1.A1_s_at | DLC1 | deleted in liver cancer 1 | 1.07E-06 | -2.60 |
| 2053 | CfaAffx.51.1.S1_s_at | --- | --- | 0.00891494 | -2.60 |
| 2054 | CfaAffx.28024.1.S1_at | CLEC4G | C-type lectin domain family 4, member G | 0.000732515 | -2.61 |
| 2055 | Cfa.3104.1.S1_at | --- | --- | 8.58E-10 | -2.61 |
| 2056 | CfaAffx.22528.1.S1_at | RND2 | Rho family GTPase 2 | 1.11E-06 | -2.61 |
| 2057 | Cfa.303.1.A1_at | --- | --- | 2.03E-06 | -2.61 |
| 2058 | CfaAffx.27143.1.S1_s_at | --- | --- | 8.03E-09 | -2.61 |
| 2059 | Cfa.14792.1.A1_s_at | --- | --- | 5.05E-10 | -2.61 |
| 2060 | CfaAffx.347.1.S1_at | LOC486381 | similar to Ig lambda chain V-I region BL2 precursor | 1.23E-05 | -2.61 |
| 2061 | Cfa.8946.1.A1_at | S100A10 | S100 calcium binding protein A10 | 3.41E-06 | -2.61 |
| 2062 | Cfa.16067.1.S1_at | DNAJA4 | DnaJ (Hsp40) homolog, subfamily A, member 4 | 1.38E-08 | -2.61 |
| 2063 | Cfa.15210.1.A1_at | --- | --- | 2.46E-06 | -2.61 |
| 2064 | Cfa.3085.1.A1_at | --- | --- | 4.70E-08 | -2.61 |
| 2065 | Cfa.18759.2.S1_at | LOC490458 | similar to S100 calcium binding protein A16 | 1.22E-09 | -2.62 |
| 2066 | Cfa.7534.1.A1_a_at | --- | --- | 0.000495029 | -2.62 |
| 2067 | Cfa.1479.1.A1_at | --- | --- | 5.37E-07 | -2.62 |
| 2068 | Cfa.1454.1.A1_at | LOC477754 | similar to fasting induced gene | 5.06E-05 | -2.62 |
| 2069 | Cfa.1473.1.A1_at | --- | --- | 3.65E-07 | -2.62 |
| 2070 | CfaAffx.23009.1.S1_s_at | LOC609192 | similar to NKG2-F type II integral membrane protein (NKG2-F activating NK recept | 5.33E-05 | -2.62 |
| 2071 | Cfa.11426.1.A1_at | --- | --- | 4.90E-08 | -2.62 |
| 2072 | CfaAffx.16558.1.S1_at | RGS2 | regulator of G-protein signaling 2, 24kDa | 0.00645366 | -2.62 |
| 2073 | Cfa.5203.1.A1_at | --- | --- | 2.71E-07 | -2.62 |
| 2074 | Cfa.12198.1.A1_at | MYL9 | myosin, light chain 9, regulatory | 6.39E-06 | -2.62 |
| 2075 | CfaAffx.1568.1.S1_at | IL26 | interleukin 26 | 1.11E-05 | -2.63 |
| 2076 | CfaAffx.17423.1.S1_at | CD160 | CD160 molecule | 1.24E-07 | -2.63 |
| 2077 | CfaAffx.19502.1.S1_at | CD84 | CD84 molecule | 1.89E-13 | -2.63 |
| 2078 | Cfa.12476.1.A1_at | --- | --- | 7.60E-08 | -2.63 |
| 2079 | Cfa.18476.1.S1_at | CD83 | CD83 molecule | 1.05E-05 | -2.63 |
| 2080 | CfaAffx.17517.1.S1_at | SLCO3A1 | solute carrier organic anion transporter family, member 3A1 | 3.30E-06 | -2.63 |
| 2081 | CfaAffx.2510.1.S1_at | TGFBI | transforming growth factor, beta-induced, 68kDa | 1.62E-06 | -2.63 |
| 2082 | Cfa.1484.1.S1_s_at | PAM | peptidylglycine alpha-amidating monooxygenase | 3.87E-10 | -2.63 |
| 2083 | CfaAffx.30901.1.S1_s_at | TGFBR3 | transforming growth factor, beta receptor III | 0.000155766 | -2.63 |
| 2084 | Cfa.5332.1.A1_at | CLIC2 | chloride intracellular channel 2 | 9.95E-08 | -2.64 |
| 2085 | Cfa.19158.1.S1_s_at | GIMAP7 | GTPase, IMAP family member 7 | 1.61E-07 | -2.64 |
| 2086 | Cfa.13258.1.A1_at | --- | --- | 5.08E-08 | -2.64 |
| 2087 | Cfa.14105.1.A1_at | --- | --- | 1.53E-08 | -2.64 |
| 2088 | Cfa.4123.1.A1_at | --- | --- | 4.32E-05 | -2.64 |
| 2089 | Cfa.12925.1.A1_at | --- | --- | 1.06E-08 | -2.64 |
| 2090 | Cfa.6224.1.A1_at | --- | --- | 0.00151478 | -2.64 |
| 2091 | CfaAffx.20741.1.S1_s_at | RHOC | ras homolog gene family, member C | 5.09E-08 | -2.64 |
| 2092 | CfaAffx.24176.1.S1_at | RHOJ | ras homolog gene family, member J | 2.10E-07 | -2.64 |
| 2093 | CfaAffx.28345.1.S1_at | IL12RB2 | interleukin 12 receptor, beta 2 | 1.54E-08 | -2.64 |
| 2094 | Cfa.4948.1.A1_s_at | ECM2 | extracellular matrix protein 2, female organ and adipocyte specific | 2.40E-05 | -2.65 |
| 2095 | Cfa.11268.1.A1_at | --- | --- | 8.90E-08 | -2.65 |
| 2096 | Cfa.15630.1.A1_at | HOXB7 | homeobox B7 | 9.28E-09 | -2.65 |
| 2097 | Cfa.560.1.S1_at | NCKAP1 | NCK-associated protein 1 | 1.72E-07 | -2.65 |
| 2098 | Cfa.18980.2.S1_at | LOC608135 | similar to Non-muscle caldesmon (CDM) (L-caldesmon) | 2.20E-07 | -2.65 |
| 2099 | Cfa.3200.1.S1_at | --- | --- | 7.45E-07 | -2.65 |
| 2100 | Cfa.1484.2.S1_at | PAM | peptidylglycine alpha-amidating monooxygenase | 2.67E-07 | -2.65 |
| 2101 | Cfa.1827.1.S1_at | --- | --- | 6.85E-07 | -2.65 |
| 2102 | Cfa.1629.1.A1_at | DMXL2 | Dmx-like 2 | 7.84E-05 | -2.65 |
| 2103 | Cfa.2036.1.S1_at | --- | --- | 0.00286589 | -2.65 |
| 2104 | CfaAffx.7698.1.S1_at | LOC487114 | similar to mannose receptor, C type 1-like 1 | 0.0106815 | -2.65 |
| 2105 | Cfa.13082.1.A1_s_at | --- | --- | 6.95E-06 | -2.65 |
| 2106 | Cfa.12408.1.A1_at | --- | --- | 0.000269799 | -2.65 |
| 2107 | Cfa.19653.1.A1_at | --- | --- | 1.99E-05 | -2.65 |
| 2108 | Cfa.15525.1.A1_at | --- | --- | 2.18E-06 | -2.65 |
| 2109 | Cfa.2098.1.A1_at | --- | --- | 7.21E-08 | -2.65 |
| 2110 | Cfa.9863.1.A1_at | --- | --- | 5.23E-05 | -2.66 |
| 2111 | Cfa.15323.1.S1_at | --- | --- | 5.33E-06 | -2.66 |
| 2112 | Cfa.3643.1.S1_s_at | PTH1R | parathyroid hormone 1 receptor | 2.26E-08 | -2.66 |
| 2113 | Cfa.16357.1.A1_at | --- | --- | 0.00151664 | -2.66 |
| 2114 | Cfa.4824.1.A1_at | LARP6 | La ribonucleoprotein domain family, member 6 | 2.10E-06 | -2.66 |
| 2115 | CfaAffx.31283.1.S1_s_at | --- | --- | 2.31E-07 | -2.66 |
| 2116 | CfaAffx.18415.1.S1_at | ST8SIA1 | ST8 alpha-N-acetyl-neuraminide alpha-2,8-sialyltransferase 1 | 4.78E-05 | -2.66 |
| 2117 | Cfa.3500.1.S1_s_at | DIO1 | deiodinase, iodothyronine, type I | 0.00230504 | -2.66 |
| 2118 | Cfa.20274.1.S1_at | DAB2 | disabled homolog 2, mitogen-responsive phosphoprotein (Drosophila) | 1.37E-06 | -2.67 |
| 2119 | Cfa.1248.1.S1_at | MPDZ | multiple PDZ domain protein | 8.01E-08 | -2.67 |
| 2120 | Cfa.9867.1.A1_at | --- | --- | 0.000195079 | -2.67 |
| 2121 | Cfa.7163.1.A1_at | TMEM51 | Transmembrane protein 51 | 6.93E-07 | -2.68 |
| 2122 | CfaAffx.21661.1.S1_s_at | LOC477555 | similar to glutathione S-transferase, theta 3 | 6.22E-05 | -2.68 |
| 2123 | CfaAffx.27331.1.S1_s_at | DNAH3 | dynein, axonemal, heavy chain 3 | 7.99E-06 | -2.68 |
| 2124 | CfaAffx.7388.1.S1_at | CST7 | cystatin F (leukocystatin) | 0.000175068 | -2.68 |
| 2125 | Cfa.18423.1.S1_at | SNCG | synuclein, gamma (breast cancer-specific protein 1) | 1.25E-06 | -2.68 |
| 2126 | CfaAffx.8715.1.S1_s_at | HNMT | histamine N-methyltransferase | 4.49E-08 | -2.68 |
| 2127 | Cfa.11105.1.A1_at | --- | --- | 1.54E-07 | -2.68 |
| 2128 | Cfa.14829.1.A1_at | --- | --- | 7.80E-08 | -2.69 |
| 2129 | Cfa.4489.1.S1_at | UCHL1 | ubiquitin carboxyl-terminal esterase L1 (ubiquitin thiolesterase) | 7.51E-06 | -2.69 |
| 2130 | CfaAffx.6380.1.S1_s_at | IL15 | interleukin 15 | 5.55E-09 | -2.69 |
| 2131 | CfaAffx.537.1.S1_x_at | --- | --- | 3.11E-05 | -2.70 |
| 2132 | Cfa.6433.1.A1_at | ZCCHC12 | zinc finger, CCHC domain containing 12 | 4.33E-07 | -2.70 |
| 2133 | CfaAffx.26481.1.S1_s_at | ZNF564 | zinc finger protein 564 | 7.92E-07 | -2.70 |
| 2134 | CfaAffx.27795.1.S1_s_at | UBE2Q2 | ubiquitin-conjugating enzyme E2Q family member 2 | 4.43E-15 | -2.70 |
| 2135 | Cfa.4466.1.A1_at | --- | --- | 1.22E-06 | -2.70 |
| 2136 | CfaAffx.23267.1.S1_at | YAP1 | Yes-associated protein 1 | 1.63E-08 | -2.70 |
| 2137 | Cfa.12686.1.A1_at | --- | --- | 0.00143794 | -2.70 |
| 2138 | Cfa.5244.1.A1_at | CD99 | CD99 molecule | 2.15E-07 | -2.70 |
| 2139 | CfaAffx.310.1.S1_at | LOC606893 | similar to Ig lambda chain V-I region BL2 precursor | 9.64E-08 | -2.70 |
| 2140 | CfaAffx.4508.1.S1_s_at | LOC484264 | similar to zinc finger protein 91 (HPF7, HTF10) | 2.17E-07 | -2.71 |
| 2141 | Cfa.6403.1.A1_at | ITGB4 | integrin, beta 4 | 2.37E-09 | -2.71 |
| 2142 | Cfa.19952.2.A1_at | --- | --- | 0.000189372 | -2.71 |
| 2143 | Cfa.19623.1.S1_at | LOC482730 | hypothetical LOC482730 | 4.49E-07 | -2.71 |
| 2144 | Cfa.14420.1.A1_at | --- | --- | 0.00021326 | -2.71 |
| 2145 | CfaAffx.2527.1.S1_s_at | PTPRK | protein tyrosine phosphatase, receptor type, K | 6.34E-10 | -2.71 |
| 2146 | Cfa.11192.1.A1_at | --- | --- | 2.39E-09 | -2.71 |
| 2147 | Cfa.5691.1.A1_at | --- | --- | 4.97E-10 | -2.71 |
| 2148 | Cfa.9083.1.A1_at | --- | --- | 1.17E-12 | -2.71 |
| 2149 | Cfa.12037.1.A1_at | PPM1J | protein phosphatase, Mg2+/Mn2+ dependent, 1J | 0.000369143 | -2.71 |
| 2150 | CfaAffx.11012.1.S1_s_at | PXDNL | peroxidasin homolog (Drosophila)-like | 1.06E-07 | -2.71 |
| 2151 | Cfa.14981.1.S1_s_at | --- | --- | 7.71E-08 | -2.71 |
| 2152 | Cfa.500.1.S1_at | EDNRA | endothelin receptor type A | 0.000104842 | -2.72 |
| 2153 | Cfa.184.1.S1_at | KIT | v-kit Hardy-Zuckerman 4 feline sarcoma viral oncogene homolog | 0.00047902 | -2.72 |
| 2154 | Cfa.16253.1.S1_at | TENC1 | tensin like C1 domain containing phosphatase (tensin 2) | 1.14E-09 | -2.72 |
| 2155 | CfaAffx.3386.1.S1_s_at | MLLT3 | myeloid/lymphoid or mixed-lineage leukemia (trithorax homolog, Drosophila); tran | 4.30E-07 | -2.72 |
| 2156 | Cfa.11653.1.A1_at | --- | --- | 8.89E-10 | -2.72 |
| 2157 | Cfa.15098.1.A1_at | LOC612917 | similar to Spindlin-like protein 2 (SPIN-2) | 0.000186527 | -2.72 |
| 2158 | Cfa.11476.1.A1_at | KANK3 | KN motif and ankyrin repeat domains 3 | 6.39E-07 | -2.72 |
| 2159 | CfaAffx.15417.1.S1_at | PDZRN4 | PDZ domain containing RING finger 4 | 1.71E-08 | -2.72 |
| 2160 | CfaAffx.29530.1.S1_s_at | ASPA | aspartoacylase (Canavan disease) | 3.49E-06 | -2.72 |
| 2161 | CfaAffx.22735.1.S1_at | GUCY1A2 | guanylate cyclase 1, soluble, alpha 2 | 2.98E-05 | -2.72 |
| 2162 | Cfa.19139.1.S1_at | --- | --- | 4.94E-06 | -2.72 |
| 2163 | CfaAffx.14654.1.S1_s_at | ACVR1 | activin A receptor, type I | 3.54E-08 | -2.73 |
| 2164 | Cfa.10832.1.A1_at | --- | --- | 0.000574749 | -2.73 |
| 2165 | Cfa.6261.1.S1_at | KRTCAP3 | keratinocyte associated protein 3 | 3.35E-11 | -2.73 |
| 2166 | Cfa.10877.1.A1_at | --- | --- | 1.45E-08 | -2.73 |
| 2167 | Cfa.13673.1.A1_at | --- | --- | 3.56E-11 | -2.73 |
| 2168 | Cfa.11867.1.A1_at | LOC480041 | similar to niban protein isoform 2 | 0.000125777 | -2.73 |
| 2169 | Cfa.9487.1.A1_at | MATN2 | matrilin 2 | 1.13E-05 | -2.73 |
| 2170 | Cfa.4744.1.A1_at | --- | --- | 1.46E-08 | -2.73 |
| 2171 | CfaAffx.6450.1.S1_s_at | GSN | gelsolin | 0.000505133 | -2.73 |
| 2172 | Cfa.12523.1.A1_at | --- | --- | 1.62E-06 | -2.73 |
| 2173 | Cfa.9204.1.A1_at | --- | --- | 2.78E-08 | -2.74 |
| 2174 | Cfa.9548.1.A1_at | PSEN2 | presenilin 2 (Alzheimer disease 4) | 3.16E-09 | -2.74 |
| 2175 | Cfa.1798.1.S1_at | --- | --- | 1.31E-07 | -2.74 |
| 2176 | Cfa.21578.1.S1_at | TMEM2 | transmembrane protein 2 | 5.46E-07 | -2.74 |
| 2177 | Cfa.5301.1.S1_at | TIE1 | tyrosine kinase with immunoglobulin-like and EGF-like domains 1 | 3.66E-08 | -2.74 |
| 2178 | Cfa.9100.1.A1_at | --- | --- | 0.00184564 | -2.74 |
| 2179 | CfaAffx.15570.1.S1_s_at | OCA2 | oculocutaneous albinism II | 0.00219068 | -2.74 |
| 2180 | Cfa.1374.1.S1_at | CPE | carboxypeptidase E | 4.53E-09 | -2.74 |
| 2181 | CfaAffx.24563.1.S1_at | KRT27 | keratin 27 | 0.00069669 | -2.74 |
| 2182 | CfaAffx.7813.1.S1_at | GIMAP7 | GTPase, IMAP family member 7 | 8.50E-09 | -2.74 |
| 2183 | Cfa.6170.1.A1_at | PDE6H | phosphodiesterase 6H, cGMP-specific, cone, gamma | 1.47E-06 | -2.74 |
| 2184 | CfaAffx.1414.1.S1_s_at | LRIG3 | leucine-rich repeats and immunoglobulin-like domains 3 | 3.55E-09 | -2.74 |
| 2185 | Cfa.6914.1.A1_at | --- | --- | 6.97E-06 | -2.74 |
| 2186 | CfaAffx.5830.1.S1_at | GKN2 | gastrokine 2 | 2.90E-08 | -2.74 |
| 2187 | Cfa.14611.1.A1_at | --- | --- | 0.000247063 | -2.74 |
| 2188 | Cfa.1995.1.A1_at | --- | --- | 6.85E-09 | -2.74 |
| 2189 | Cfa.17958.1.S1_s_at | MARCKSL1 | MARCKS-like 1 | 0.000415729 | -2.74 |
| 2190 | CfaAffx.21092.1.S1_s_at | TRIM69 | tripartite motif-containing 69 | 3.03E-06 | -2.75 |
| 2191 | CfaAffx.4501.1.S1_at | LOC484264 | similar to zinc finger protein 91 (HPF7, HTF10) | 2.06E-07 | -2.75 |
| 2192 | Cfa.15263.1.A1_s_at | PHLDB2 | pleckstrin homology-like domain, family B, member 2 | 3.81E-06 | -2.75 |
| 2193 | CfaAffx.24021.1.S1_s_at | --- | --- | 0.000141677 | -2.75 |
| 2194 | CfaAffx.4067.1.S1_s_at | SGCE | sarcoglycan, epsilon | 2.67E-08 | -2.75 |
| 2195 | Cfa.7915.1.A1_at | --- | --- | 9.65E-05 | -2.75 |
| 2196 | CfaAffx.8303.1.S1_s_at | VILL | villin-like | 8.57E-11 | -2.75 |
| 2197 | Cfa.14733.1.A1_s_at | MCAM | melanoma cell adhesion molecule | 2.29E-07 | -2.75 |
| 2198 | CfaAffx.12530.1.S1_s_at | STOX2 | storkhead box 2 | 4.21E-08 | -2.75 |
| 2199 | Cfa.8595.1.A1_a_at | PGCP | plasma glutamate carboxypeptidase | 2.12E-07 | -2.75 |
| 2200 | CfaAffx.2345.1.S1_s_at | SNTB1 | syntrophin, beta 1 (dystrophin-associated protein A1, 59kDa, basic component 1) | 3.53E-09 | -2.75 |
| 2201 | Cfa.9196.1.A1_at | --- | --- | 1.05E-07 | -2.76 |
| 2202 | Cfa.6369.1.A1_at | FBP1 | fructose-1,6-bisphosphatase 1 | 0.0030451 | -2.76 |
| 2203 | Cfa.9207.1.A1_at | --- | --- | 3.58E-09 | -2.76 |
| 2204 | Cfa.14933.1.A1_at | --- | --- | 4.99E-07 | -2.76 |
| 2205 | Cfa.2493.1.S1_at | GLRB | glycine receptor, beta | 0.00483932 | -2.76 |
| 2206 | CfaAffx.3209.1.S1_s_at | DNAH8 | dynein, axonemal, heavy chain 8 | 7.10E-13 | -2.76 |
| 2207 | CfaAffx.10118.1.S1_at | POSTN | periostin, osteoblast specific factor | 0.000402793 | -2.76 |
| 2208 | Cfa.125.1.S1_s_at | EDN1 | endothelin 1 | 0.000919264 | -2.76 |
| 2209 | Cfa.12217.1.A1_at | --- | --- | 0.000218488 | -2.76 |
| 2210 | Cfa.13931.1.A1_at | PLEKHG3 | pleckstrin homology domain containing, family G (with RhoGef domain) member 3 | 1.12E-08 | -2.76 |
| 2211 | CfaAffx.11152.1.S1_at | RAD21L1 | RAD21-like 1 (S. pombe) | 9.51E-15 | -2.76 |
| 2212 | CfaAffx.11634.1.S1_s_at | ZNF792 | zinc finger protein 792 | 3.57E-06 | -2.76 |
| 2213 | Cfa.15944.1.A1_at | ATP10A | ATPase, class V, type 10A | 0.00125705 | -2.77 |
| 2214 | CfaAffx.956.1.S1_at | PLAC8 | placenta-specific 8 | 0.000186807 | -2.77 |
| 2215 | Cfa.15700.1.A1_s_at | PHLDB2 | pleckstrin homology-like domain, family B, member 2 | 2.24E-08 | -2.77 |
| 2216 | Cfa.485.1.A1_at | --- | --- | 6.34E-06 | -2.77 |
| 2217 | Cfa.1610.1.S1_at | --- | --- | 2.10E-09 | -2.78 |
| 2218 | Cfa.2308.1.A1_at | --- | --- | 1.33E-06 | -2.78 |
| 2219 | CfaAffx.17445.1.S1_s_at | LOC486632 /// LOC491723 /// LOC608476 | similar to Fatty acid-binding protein, adipocyte (AFABP) (Adipocyte lipid-bindin | 0.0088679 | -2.78 |
| 2220 | CfaAffx.16257.1.S1_s_at | PHLDB2 | pleckstrin homology-like domain, family B, member 2 | 3.27E-06 | -2.78 |
| 2221 | Cfa.14473.1.A1_x_at | --- | --- | 0.0027626 | -2.78 |
| 2222 | Cfa.16986.1.S1_at | LOC490075 | similar to abnormal CHEmotaxis family member (che-11) | 5.12E-17 | -2.78 |
| 2223 | Cfa.522.1.A1_at | LIN7B | lin-7 homolog B (C. elegans) | 1.97E-08 | -2.78 |
| 2224 | Cfa.9479.1.A1_at | --- | --- | 7.04E-06 | -2.78 |
| 2225 | CfaAffx.15646.1.S1_s_at | STAT4 | signal transducer and activator of transcription 4 | 2.66E-06 | -2.78 |
| 2226 | CfaAffx.11273.1.S1_s_at | EHF | ets homologous factor | 6.54E-08 | -2.78 |
| 2227 | Cfa.15478.1.A1_at | C4BPB | complement component 4 binding protein, beta | 0.00632318 | -2.79 |
| 2228 | Cfa.1886.1.S1_at | --- | --- | 7.22E-09 | -2.79 |
| 2229 | CfaAffx.3955.1.S1_s_at | RCAN2 | regulator of calcineurin 2 | 3.86E-11 | -2.79 |
| 2230 | Cfa.5417.1.A1_at | --- | --- | 8.35E-09 | -2.79 |
| 2231 | Cfa.14137.1.A1_at | --- | --- | 1.19E-06 | -2.79 |
| 2232 | CfaAffx.17443.1.S1_at | NR5A2 | nuclear receptor subfamily 5, group A, member 2 | 3.15E-07 | -2.79 |
| 2233 | CfaAffx.28663.1.S1_at | LOC611582 | similar to FLJ44048 protein | 7.54E-06 | -2.79 |
| 2234 | Cfa.20505.1.S1_at | --- | --- | 9.08E-14 | -2.79 |
| 2235 | CfaAffx.16021.1.S1_at | ARHGAP32 | Rho GTPase activating protein 32 | 1.47E-09 | -2.79 |
| 2236 | Cfa.7446.1.A1_at | ADCY2 | adenylate cyclase 2 (brain) | 3.99E-05 | -2.79 |
| 2237 | CfaAffx.345.1.S1_at | LOC491494 /// LOC607125 | similar to Ig lambda chain V region 4A precursor /// similar to Ig lambda chain | 2.86E-08 | -2.80 |
| 2238 | Cfa.18102.1.S1_at | DAB2 | disabled homolog 2, mitogen-responsive phosphoprotein (Drosophila) | 1.13E-06 | -2.80 |
| 2239 | CfaAffx.21638.1.S1_at | LOC611565 | similar to scavenger receptor cysteine-rich type 1 protein M160 precursor | 2.39E-11 | -2.80 |
| 2240 | CfaAffx.20721.1.S1_s_at | CCL24 | chemokine (C-C motif) ligand 24 | 0.000833656 | -2.80 |
| 2241 | Cfa.2627.1.A1_at | --- | --- | 0.000599443 | -2.80 |
| 2242 | Cfa.6053.1.S1_at | MCAM | melanoma cell adhesion molecule | 7.90E-07 | -2.80 |
| 2243 | CfaAffx.13342.1.S1_at | VEGFC | vascular endothelial growth factor C | 1.32E-07 | -2.80 |
| 2244 | Cfa.19007.1.S1_s_at | C7 | complement component 7 | 1.59E-06 | -2.80 |
| 2245 | Cfa.4830.1.A1_at | RBPMS | RNA binding protein with multiple splicing | 3.87E-05 | -2.80 |
| 2246 | Cfa.10234.1.A1_at | --- | --- | 5.06E-09 | -2.80 |
| 2247 | Cfa.15809.1.S1_at | CCL19 | chemokine (C-C motif) ligand 19 | 0.000156591 | -2.80 |
| 2248 | Cfa.17874.1.S1_s_at | IGF2 | insulin-like growth factor 2 (somatomedin A) | 0.000729719 | -2.80 |
| 2249 | Cfa.15089.1.A1_at | THRA | Thyroid hormone receptor, alpha (erythroblastic leukemia viral (v-erb-a) oncogen | 1.08E-12 | -2.80 |
| 2250 | Cfa.13357.1.A1_s_at | THSD1 | thrombospondin, type I, domain containing 1 | 2.78E-06 | -2.81 |
| 2251 | Cfa.11315.1.A1_at | --- | --- | 4.80E-08 | -2.81 |
| 2252 | Cfa.5358.1.A1_at | --- | --- | 3.67E-07 | -2.81 |
| 2253 | CfaAffx.12260.1.S1_at | SLCO6A1 | solute carrier organic anion transporter family, member 6A1 | 4.63E-05 | -2.81 |
| 2254 | Cfa.13031.1.A1_at | --- | --- | 4.11E-08 | -2.81 |
| 2255 | CfaAffx.3870.1.S1_s_at | DNAJB5 | DnaJ (Hsp40) homolog, subfamily B, member 5 | 1.94E-08 | -2.81 |
| 2256 | Cfa.8880.1.A1_at | --- | --- | 2.56E-09 | -2.81 |
| 2257 | Cfa.6867.1.A1_at | DARC | Duffy blood group, chemokine receptor | 7.57E-05 | -2.81 |
| 2258 | Cfa.2037.1.A1_at | FARP1 | FERM, RhoGEF (ARHGEF) and pleckstrin domain protein 1 (chondrocyte-derived) | 1.05E-09 | -2.81 |
| 2259 | Cfa.11549.1.A1_s_at | FBXW10 | F-box and WD repeat domain containing 10 | 2.12E-10 | -2.82 |
| 2260 | Cfa.12746.1.S1_at | EPCAM | epithelial cell adhesion molecule | 0.00103045 | -2.82 |
| 2261 | Cfa.4978.1.A1_at | --- | --- | 1.84E-09 | -2.82 |
| 2262 | CfaAffx.10900.1.S1_at | SNAI2 | snail homolog 2 (Drosophila) | 7.26E-08 | -2.82 |
| 2263 | CfaAffx.23518.1.S1_at | KCNA1 | potassium voltage-gated channel, shaker-related subfamily, member 1 (episodic at | 5.95E-07 | -2.82 |
| 2264 | Cfa.3.1.S1_s_at | SLC46A2 | solute carrier family 46, member 2 | 1.56E-06 | -2.82 |
| 2265 | CfaAffx.10355.1.S1_at | FGF1 | fibroblast growth factor 1 (acidic) | 0.000433394 | -2.82 |
| 2266 | CfaAffx.6292.1.S1_at | --- | --- | 9.39E-06 | -2.82 |
| 2267 | Cfa.878.1.A1_at | GSTM3 | glutathione S-transferase mu 3 (brain) | 0.000169765 | -2.82 |
| 2268 | Cfa.15471.1.A1_at | SEPP1 | selenoprotein P, plasma, 1 | 7.80E-05 | -2.83 |
| 2269 | Cfa.8483.1.A1_a_at | --- | --- | 1.84E-06 | -2.83 |
| 2270 | CfaAffx.15058.1.S1_at | LOC478715 | similar to bone morphogenetic protein 6 | 5.17E-06 | -2.83 |
| 2271 | Cfa.13715.1.A1_at | LOC479458 | caspase 12 | 0.000915546 | -2.83 |
| 2272 | CfaAffx.17010.1.S1_s_at | ABCA8 | ATP-binding cassette, sub-family A (ABC1), member 8 | 0.00417643 | -2.83 |
| 2273 | Cfa.2597.1.S1_at | --- | --- | 1.41E-05 | -2.83 |
| 2274 | Cfa.1627.1.S1_at | --- | --- | 8.26E-07 | -2.83 |
| 2275 | Cfa.6243.1.A1_at | LOC611035 | hypothetical protein LOC611035 | 2.02E-10 | -2.83 |
| 2276 | Cfa.1558.1.A1_at | --- | --- | 0.000120563 | -2.83 |
| 2277 | Cfa.19420.1.S1_at | --- | --- | 2.68E-07 | -2.83 |
| 2278 | Cfa.3036.1.S1_at | B3GALT2 | UDP-Gal:betaGlcNAc beta 1,3-galactosyltransferase, polypeptide 2 | 0.00334036 | -2.83 |
| 2279 | Cfa.12195.5.S1_at | ACTC1 | actin, alpha, cardiac muscle 1 | 0.00263137 | -2.84 |
| 2280 | Cfa.9823.1.A1_at | --- | --- | 3.24E-10 | -2.84 |
| 2281 | CfaAffx.5189.1.S1_s_at | NKG7 | natural killer cell group 7 sequence | 0.0015936 | -2.84 |
| 2282 | Cfa.8955.1.A1_at | --- | --- | 2.19E-10 | -2.84 |
| 2283 | Cfa.531.1.S1_at | --- | --- | 0.00168888 | -2.85 |
| 2284 | Cfa.5931.1.A1_s_at | --- | --- | 8.27E-06 | -2.85 |
| 2285 | Cfa.14550.1.A1_at | PARD3 | par-3 partitioning defective 3 homolog (C. elegans) | 1.61E-05 | -2.85 |
| 2286 | Cfa.12289.1.A1_at | KLK4 | kallikrein-related peptidase 4 | 0.000171781 | -2.85 |
| 2287 | Cfa.2579.1.A1_at | LOC475470 | similar to tripartite motif protein TRIM2 | 3.17E-05 | -2.85 |
| 2288 | Cfa.3154.1.A1_at | --- | --- | 4.71E-07 | -2.85 |
| 2289 | Cfa.12309.1.A1_s_at | NSUN7 | NOP2/Sun domain family, member 7 | 7.65E-09 | -2.85 |
| 2290 | CfaAffx.23850.1.S1_x_at | LOC491686 | similar to Ig kappa chain V-II region RPMI 6410 precursor | 1.91E-05 | -2.85 |
| 2291 | Cfa.1667.1.A1_at | --- | --- | 3.57E-06 | -2.86 |
| 2292 | Cfa.6920.1.A1_at | --- | --- | 0.00120447 | -2.86 |
| 2293 | Cfa.3887.2.A1_s_at | FXYD1 | FXYD domain containing ion transport regulator 1 | 6.53E-08 | -2.86 |
| 2294 | Cfa.17597.1.S1_at | LOC609015 | similar to chromosome 9 open reading frame 61 | 8.67E-09 | -2.86 |
| 2295 | CfaAffx.25266.1.S1_at | LOC612917 | similar to Spindlin-like protein 2 (SPIN-2) | 5.37E-06 | -2.86 |
| 2296 | Cfa.20410.1.S1_at | --- | --- | 3.04E-06 | -2.86 |
| 2297 | CfaAffx.2958.1.S1_s_at | NTRK2 | neurotrophic tyrosine kinase, receptor, type 2 | 1.80E-05 | -2.86 |
| 2298 | CfaAffx.24414.1.S1_at | FOXP3 | forkhead box P3 | 2.33E-10 | -2.86 |
| 2299 | CfaAffx.24064.1.S1_at | ACTA2 | actin, alpha 2, smooth muscle, aorta | 6.40E-06 | -2.86 |
| 2300 | CfaAffx.4423.1.S1_s_at | LPHN3 | latrophilin 3 | 3.30E-07 | -2.86 |
| 2301 | Cfa.13436.1.A1_at | SLC16A14 | solute carrier family 16, member 14 (monocarboxylic acid transporter 14) | 1.85E-08 | -2.87 |
| 2302 | CfaAffx.5084.1.S1_at | ZNF462 | zinc finger protein 462 | 1.30E-07 | -2.87 |
| 2303 | Cfa.8993.1.A1_at | --- | --- | 5.01E-14 | -2.87 |
| 2304 | Cfa.2314.1.A1_at | --- | --- | 5.68E-05 | -2.87 |
| 2305 | Cfa.1512.1.A1_at | --- | --- | 2.15E-07 | -2.87 |
| 2306 | Cfa.13661.1.A1_at | --- | --- | 8.22E-06 | -2.87 |
| 2307 | Cfa.14342.1.A1_a_at | GPX3 | glutathione peroxidase 3 | 6.95E-06 | -2.87 |
| 2308 | CfaAffx.27686.1.S1_at | LOC611757 | similar to proline-serine-threonine phosphatase interacting protein 1 | 2.29E-08 | -2.87 |
| 2309 | Cfa.15083.1.S1_at | EFEMP1 | EGF-containing fibulin-like extracellular matrix protein 1 | 0.00408431 | -2.87 |
| 2310 | Cfa.8759.1.A1_at | HSPA1L | heat shock 70kDa protein 1-like | 9.32E-07 | -2.87 |
| 2311 | Cfa.15179.2.A1_at | --- | --- | 1.30E-07 | -2.87 |
| 2312 | Cfa.2656.1.S1_at | --- | --- | 6.55E-07 | -2.87 |
| 2313 | Cfa.11049.1.A1_at | APOL5 | apolipoprotein L, 5 | 6.51E-09 | -2.88 |
| 2314 | Cfa.14631.1.A1_at | --- | --- | 8.56E-07 | -2.88 |
| 2315 | Cfa.6341.1.A1_at | PDZK1IP1 | PDZK1 interacting protein 1 | 0.00101876 | -2.88 |
| 2316 | Cfa.10852.1.A1_at | --- | --- | 1.25E-06 | -2.88 |
| 2317 | Cfa.1931.1.A1_at | SMARCA1 | SWI/SNF related, matrix associated, actin dependent regulator of chromatin, subf | 1.50E-08 | -2.88 |
| 2318 | Cfa.15610.1.A1_s_at | GPR116 | G protein-coupled receptor 116 | 2.83E-06 | -2.88 |
| 2319 | Cfa.878.1.A1_s_at | GSTM3 | glutathione S-transferase mu 3 (brain) | 0.000163497 | -2.88 |
| 2320 | Cfa.3887.1.S1_s_at | FXYD1 | FXYD domain containing ion transport regulator 1 | 2.41E-08 | -2.88 |
| 2321 | Cfa.9852.1.A1_at | --- | --- | 4.78E-09 | -2.88 |
| 2322 | Cfa.16339.1.S1_at | CXCR3 | chemokine (C-X-C motif) receptor 3 | 0.000860491 | -2.89 |
| 2323 | Cfa.572.2.S1_a_at | CDO1 | cysteine dioxygenase, type I | 0.00701668 | -2.89 |
| 2324 | Cfa.13606.1.A1_at | --- | --- | 3.48E-08 | -2.89 |
| 2325 | Cfa.2500.1.S1_at | --- | --- | 7.01E-08 | -2.89 |
| 2326 | Cfa.2349.1.S1_at | --- | --- | 1.49E-08 | -2.89 |
| 2327 | Cfa.12307.1.A1_at | --- | --- | 4.77E-06 | -2.89 |
| 2328 | Cfa.12797.1.A1_at | --- | --- | 0.00506471 | -2.89 |
| 2329 | Cfa.9117.1.A1_at | --- | --- | 9.38E-06 | -2.89 |
| 2330 | CfaAffx.1630.1.S1_s_at | PTPRB /// PTPRR | protein tyrosine phosphatase, receptor type, B /// protein tyrosine phosphatase, | 4.81E-07 | -2.89 |
| 2331 | Cfa.11992.1.A1_at | TCEA2 | transcription elongation factor A (SII), 2 | 4.47E-15 | -2.89 |
| 2332 | CfaAffx.25257.1.S1_at | LOC612077 | hypothetical protein LOC612077 | 4.23E-05 | -2.90 |
| 2333 | CfaAffx.23153.1.S1_at | MMP13 | matrix metallopeptidase 13 (collagenase 3) | 0.0105255 | -2.90 |
| 2334 | Cfa.11335.1.A1_at | AMDHD1 | amidohydrolase domain containing 1 | 7.50E-06 | -2.90 |
| 2335 | Cfa.3619.1.S1_s_at | CD38 | CD38 molecule | 2.10E-05 | -2.90 |
| 2336 | Cfa.12190.1.A1_at | PTGES | prostaglandin E synthase | 3.20E-05 | -2.90 |
| 2337 | CfaAffx.26464.1.S1_s_at | ZNF791 | zinc finger protein 791 | 1.70E-05 | -2.90 |
| 2338 | Cfa.14473.1.A1_at | --- | --- | 0.00237872 | -2.90 |
| 2339 | Cfa.11382.1.A1_at | ACSF2 | acyl-CoA synthetase family member 2 | 3.02E-05 | -2.90 |
| 2340 | CfaAffx.11636.1.S1_at | ZNF792 | zinc finger protein 792 | 7.31E-07 | -2.90 |
| 2341 | Cfa.2316.1.S1_at | --- | --- | 0.000170164 | -2.91 |
| 2342 | Cfa.14387.1.A1_s_at | ACP6 | acid phosphatase 6, lysophosphatidic | 3.39E-10 | -2.91 |
| 2343 | Cfa.20785.1.S1_s_at | SRGN | serglycin | 8.21E-08 | -2.91 |
| 2344 | Cfa.12614.1.A1_at | --- | --- | 1.91E-08 | -2.91 |
| 2345 | CfaAffx.7949.1.S1_at | --- | --- | 1.97E-05 | -2.91 |
| 2346 | CfaAffx.27758.1.S1_at | CHRDL1 | chordin-like 1 | 5.81E-09 | -2.91 |
| 2347 | Cfa.2512.1.S1_at | --- | --- | 0.000825903 | -2.91 |
| 2348 | Cfa.10685.1.A1_at | --- | --- | 1.16E-07 | -2.91 |
| 2349 | CfaAffx.28758.1.S1_s_at | INADL | InaD-like (Drosophila) | 6.37E-05 | -2.92 |
| 2350 | Cfa.14972.2.S1_a_at | SVIL | supervillin | 1.81E-07 | -2.92 |
| 2351 | Cfa.4103.1.A1_at | LDOC1 | leucine zipper, down-regulated in cancer 1 | 4.56E-07 | -2.92 |
| 2352 | CfaAffx.12902.1.S1_s_at | NR2F1 | nuclear receptor subfamily 2, group F, member 1 | 1.14E-08 | -2.92 |
| 2353 | Cfa.14676.1.A1_at | --- | --- | 4.59E-06 | -2.92 |
| 2354 | CfaAffx.19909.1.S1_s_at | ANK3 | ankyrin 3, node of Ranvier (ankyrin G) | 2.65E-10 | -2.92 |
| 2355 | Cfa.12255.1.A1_at | --- | --- | 0.000365679 | -2.92 |
| 2356 | Cfa.139.1.A1_at | --- | --- | 3.25E-08 | -2.92 |
| 2357 | Cfa.4577.1.A1_at | --- | --- | 3.17E-07 | -2.92 |
| 2358 | Cfa.12678.1.A1_at | --- | --- | 0.000549313 | -2.92 |
| 2359 | Cfa.10549.1.A1_at | --- | --- | 0.000179613 | -2.92 |
| 2360 | Cfa.2084.1.A1_at | --- | --- | 1.57E-07 | -2.93 |
| 2361 | Cfa.1245.1.S1_s_at | --- | --- | 1.03E-06 | -2.93 |
| 2362 | Cfa.1645.1.S1_at | MYH11 | myosin, heavy chain 11, smooth muscle | 5.04E-07 | -2.93 |
| 2363 | CfaAffx.17039.1.S1_at | NR2F2 | nuclear receptor subfamily 2, group F, member 2 | 4.14E-07 | -2.93 |
| 2364 | Cfa.18478.1.S1_at | BCAM | basal cell adhesion molecule (Lutheran blood group) | 1.65E-08 | -2.93 |
| 2365 | CfaAffx.4990.1.S1_s_at | CDCA7L | cell division cycle associated 7-like | 0.000100892 | -2.93 |
| 2366 | CfaAffx.16677.1.S1_s_at | ACRV1 | acrosomal vesicle protein 1 | 3.89E-09 | -2.94 |
| 2367 | Cfa.1752.1.S2_at | PDE5A | phosphodiesterase 5A, cGMP-specific | 3.96E-10 | -2.94 |
| 2368 | Cfa.15835.1.S1_at | --- | --- | 0.0127019 | -2.94 |
| 2369 | Cfa.11304.1.A1_at | TPBG | trophoblast glycoprotein | 5.43E-09 | -2.94 |
| 2370 | CfaAffx.4737.1.S1_at | LOC481633 | similar to plasticity related gene 3 | 8.83E-05 | -2.94 |
| 2371 | Cfa.6440.1.A1_s_at | --- | --- | 5.98E-12 | -2.94 |
| 2372 | CfaAffx.20168.1.S1_s_at | FN3K | fructosamine 3 kinase | 7.60E-07 | -2.94 |
| 2373 | Cfa.1249.1.S1_at | --- | --- | 8.73E-06 | -2.94 |
| 2374 | Cfa.4973.1.A1_at | --- | --- | 4.29E-08 | -2.94 |
| 2375 | CfaAffx.28081.1.S1_at | HTR4 | 5-hydroxytryptamine (serotonin) receptor 4 | 1.76E-05 | -2.94 |
| 2376 | Cfa.14413.1.S1_at | PROS1 | protein S (alpha) | 0.000135541 | -2.95 |
| 2377 | Cfa.14849.1.A1_at | --- | --- | 3.54E-07 | -2.95 |
| 2378 | Cfa.15276.1.A1_at | GPRC5B | G protein-coupled receptor, family C, group 5, member B | 3.43E-06 | -2.95 |
| 2379 | Cfa.10050.1.A1_at | --- | --- | 2.66E-05 | -2.95 |
| 2380 | Cfa.19449.1.S1_at | --- | --- | 0.000210674 | -2.95 |
| 2381 | Cfa.1851.1.S1_at | --- | --- | 6.01E-08 | -2.95 |
| 2382 | Cfa.20779.1.S1_at | CXCL12 | chemokine (C-X-C motif) ligand 12 | 7.18E-07 | -2.95 |
| 2383 | CfaAffx.6781.1.S1_s_at | HSD17B14 | hydroxysteroid (17-beta) dehydrogenase 14 | 6.67E-05 | -2.95 |
| 2384 | CfaAffx.23.1.S1_at | DMD | dystrophin (muscular dystrophy, Duchenne and Becker types) | 1.59E-08 | -2.95 |
| 2385 | Cfa.3798.1.A1_at | INPP5A | inositol polyphosphate-5-phosphatase, 40kDa | 5.08E-06 | -2.95 |
| 2386 | Cfa.15263.2.A1_at | PHLDB2 | pleckstrin homology-like domain, family B, member 2 | 2.94E-07 | -2.96 |
| 2387 | Cfa.20785.1.S1_at | SRGN | serglycin | 3.55E-07 | -2.96 |
| 2388 | CfaAffx.1767.1.S1_at | CNKSR3 | CNKSR family member 3 | 1.20E-09 | -2.96 |
| 2389 | Cfa.13894.1.S1_s_at | MME | membrane metallo-endopeptidase | 6.58E-05 | -2.96 |
| 2390 | Cfa.3856.1.S1_s_at | PTHLH | parathyroid hormone-like hormone | 2.30E-08 | -2.96 |
| 2391 | Cfa.5536.1.A1_at | MYCN | v-myc myelocytomatosis viral related oncogene, neuroblastoma derived (avian) | 5.41E-05 | -2.96 |
| 2392 | CfaAffx.4916.1.S1_at | STON1-GTF2A1L | STON1-GTF2A1L readthrough | 2.62E-08 | -2.96 |
| 2393 | Cfa.4569.1.S1_s_at | IL21 | interleukin 21 | 0.00138937 | -2.96 |
| 2394 | Cfa.4090.1.A1_s_at | MGLL | monoglyceride lipase | 1.18E-05 | -2.96 |
| 2395 | CfaAffx.14934.1.S1_at | NRN1 | neuritin 1 | 0.0186897 | -2.96 |
| 2396 | Cfa.12424.1.A1_at | RGS10 | regulator of G-protein signaling 10 | 6.13E-09 | -2.96 |
| 2397 | CfaAffx.14248.1.S1_at | TMEM55A | transmembrane protein 55A | 1.55E-08 | -2.97 |
| 2398 | Cfa.4940.1.A1_at | --- | --- | 1.08E-05 | -2.97 |
| 2399 | Cfa.10634.1.A1_at | ITGA7 | integrin, alpha 7 | 1.18E-06 | -2.97 |
| 2400 | CfaAffx.28328.1.S1_at | PRAM1 | PML-RARA regulated adaptor molecule 1 | 9.77E-09 | -2.97 |
| 2401 | Cfa.19426.1.S1_at | --- | --- | 1.73E-06 | -2.97 |
| 2402 | Cfa.19584.1.A1_at | --- | --- | 3.74E-08 | -2.97 |
| 2403 | Cfa.13363.1.A1_at | LOC479637 | hypothetical LOC479637 | 7.57E-07 | -2.97 |
| 2404 | CfaAffx.3904.1.S1_at | --- | --- | 0.000173352 | -2.98 |
| 2405 | Cfa.7812.1.A1_at | --- | --- | 8.19E-12 | -2.98 |
| 2406 | CfaAffx.12367.1.S1_at | MFAP3L | microfibrillar-associated protein 3-like | 0.000394222 | -2.98 |
| 2407 | Cfa.2273.1.S1_at | SLC39A12 | solute carrier family 39 (zinc transporter), member 12 | 0.00090925 | -2.98 |
| 2408 | Cfa.6133.1.S1_at | DPT | dermatopontin | 0.00398856 | -2.98 |
| 2409 | Cfa.14075.1.A1_at | INADL | InaD-like (Drosophila) | 8.56E-06 | -2.98 |
| 2410 | CfaAffx.24953.1.S1_s_at | GSDMA | gasdermin A | 1.22E-07 | -2.99 |
| 2411 | CfaAffx.14120.1.S1_at | LOC609038 | similar to lysozyme | 0.0123591 | -2.99 |
| 2412 | CfaAffx.8999.1.S1_at | ago61 | glycosyltransferase | 7.83E-10 | -2.99 |
| 2413 | CfaAffx.3963.1.S1_at | VLDLR | very low density lipoprotein receptor | 6.26E-05 | -2.99 |
| 2414 | CfaAffx.30818.1.S1_s_at | BCAR3 | breast cancer anti-estrogen resistance 3 | 9.06E-07 | -2.99 |
| 2415 | Cfa.17561.1.S1_at | ITGB5 | integrin, beta 5 | 1.92E-06 | -3.00 |
| 2416 | Cfa.12222.1.A1_s_at | NKG7 | natural killer cell group 7 sequence | 0.00116957 | -3.00 |
| 2417 | CfaAffx.19514.1.S1_s_at | SLAMF1 | signaling lymphocytic activation molecule family member 1 | 2.62E-09 | -3.00 |
| 2418 | Cfa.10767.1.S1_s_at | CYP3A12 /// LOC479740 | cytochrome P-450 3A12 /// hypothetical LOC479740 | 0.0155848 | -3.00 |
| 2419 | Cfa.12367.1.A1_at | --- | --- | 1.12E-07 | -3.01 |
| 2420 | Cfa.12543.1.A1_at | NPR2 | natriuretic peptide receptor B/guanylate cyclase B (atrionatriuretic peptide rec | 3.72E-09 | -3.01 |
| 2421 | Cfa.19446.1.S1_at | --- | --- | 2.53E-08 | -3.01 |
| 2422 | Cfa.1575.1.A1_s_at | MID2 | midline 2 | 2.61E-05 | -3.01 |
| 2423 | CfaAffx.12064.1.S1_s_at | GLT8D2 | glycosyltransferase 8 domain containing 2 | 6.92E-08 | -3.01 |
| 2424 | Cfa.20638.1.S1_at | CPT1C | carnitine palmitoyltransferase 1C | 7.04E-06 | -3.02 |
| 2425 | CfaAffx.8422.1.S1_at | NCKAP5 | NCK-associated protein 5 | 1.42E-10 | -3.02 |
| 2426 | Cfa.2541.1.A1_at | LOC611318 | similar to F55A4.8a | 2.61E-06 | -3.02 |
| 2427 | Cfa.14091.1.A1_at | --- | --- | 1.06E-09 | -3.03 |
| 2428 | Cfa.21286.1.S1_at | EFNA1 | ephrin-A1 | 6.16E-09 | -3.03 |
| 2429 | Cfa.11188.1.A1_at | --- | --- | 0.0014663 | -3.03 |
| 2430 | Cfa.13599.1.A1_at | RH30 | Rh antigen-like protein | 3.27E-06 | -3.03 |
| 2431 | Cfa.572.1.S1_at | CDO1 | cysteine dioxygenase, type I | 0.00365304 | -3.03 |
| 2432 | Cfa.11540.1.A1_at | --- | --- | 6.42E-05 | -3.03 |
| 2433 | Cfa.3098.1.S1_at | --- | --- | 5.38E-11 | -3.03 |
| 2434 | Cfa.11676.1.A1_at | --- | --- | 2.91E-09 | -3.04 |
| 2435 | CfaAffx.2836.1.S1_s_at | GRAP2 | GRB2-related adaptor protein 2 | 1.49E-10 | -3.04 |
| 2436 | Cfa.6909.1.A1_at | ARHGEF15 | Rho guanine nucleotide exchange factor (GEF) 15 | 2.48E-08 | -3.04 |
| 2437 | CfaAffx.29397.1.S1_s_at | CELF5 | CUGBP, Elav-like family member 5 | 1.80E-05 | -3.04 |
| 2438 | Cfa.8978.1.A1_at | --- | --- | 2.90E-06 | -3.04 |
| 2439 | Cfa.10415.1.A1_at | --- | --- | 0.0013825 | -3.04 |
| 2440 | Cfa.3110.1.A1_at | AHNAK | AHNAK nucleoprotein | 1.91E-07 | -3.05 |
| 2441 | Cfa.6138.1.A1_at | EMR4P | egf-like module containing, mucin-like, hormone receptor-like 4 pseudogene | 0.0132115 | -3.05 |
| 2442 | Cfa.5098.1.A1_at | --- | --- | 0.00184973 | -3.05 |
| 2443 | Cfa.14550.1.A1_s_at | PARD3 | par-3 partitioning defective 3 homolog (C. elegans) | 7.01E-07 | -3.05 |
| 2444 | Cfa.9605.1.A1_at | --- | --- | 5.30E-07 | -3.05 |
| 2445 | Cfa.16871.1.S1_at | SLC27A6 | solute carrier family 27 (fatty acid transporter), member 6 | 0.00144585 | -3.05 |
| 2446 | CfaAffx.571.1.S1_at | LOC611632 | similar to Ceruloplasmin precursor (Ferroxidase) | 7.76E-08 | -3.05 |
| 2447 | Cfa.11630.2.A1_s_at | CNN3 | calponin 3, acidic | 3.26E-09 | -3.05 |
| 2448 | CfaAffx.22720.1.S1_s_at | GUCY1A2 | guanylate cyclase 1, soluble, alpha 2 | 9.26E-06 | -3.05 |
| 2449 | Cfa.14053.1.A1_at | --- | --- | 2.12E-07 | -3.06 |
| 2450 | Cfa.5696.1.A1_at | --- | --- | 7.33E-06 | -3.06 |
| 2451 | CfaAffx.16221.1.S1_s_at | CD96 | CD96 molecule | 1.94E-05 | -3.06 |
| 2452 | Cfa.10212.1.A1_at | MAPRE3 | microtubule-associated protein, RP/EB family, member 3 | 1.03E-10 | -3.06 |
| 2453 | Cfa.9782.1.A1_at | --- | --- | 1.17E-05 | -3.07 |
| 2454 | Cfa.11059.1.A1_at | AMT | aminomethyltransferase | 9.86E-10 | -3.07 |
| 2455 | Cfa.5940.1.A1_at | LOC475003 | similar to Y57A10A.26 | 1.86E-08 | -3.07 |
| 2456 | Cfa.15422.1.A1_at | --- | --- | 8.66E-11 | -3.07 |
| 2457 | Cfa.2874.1.A1_at | --- | --- | 1.20E-06 | -3.07 |
| 2458 | Cfa.17854.1.S1_at | ZNF704 | zinc finger protein 704 | 5.40E-10 | -3.07 |
| 2459 | Cfa.5694.1.A1_at | --- | --- | 3.52E-09 | -3.08 |
| 2460 | CfaAffx.21462.1.S1_s_at | GGT1 | gamma-glutamyltransferase 1 | 0.000264756 | -3.08 |
| 2461 | Cfa.3588.1.S1_at | CLCN1 | chloride channel 1, skeletal muscle | 3.53E-06 | -3.08 |
| 2462 | Cfa.20111.1.S1_s_at | NSUN7 | NOP2/Sun domain family, member 7 | 1.65E-09 | -3.08 |
| 2463 | Cfa.12465.1.A1_at | --- | --- | 3.85E-07 | -3.08 |
| 2464 | CfaAffx.12246.1.S1_at | GGT7 | gamma-glutamyltransferase 7 | 9.97E-11 | -3.08 |
| 2465 | Cfa.2465.1.A1_at | AGPHD1 | aminoglycoside phosphotransferase domain containing 1 | 6.46E-06 | -3.08 |
| 2466 | Cfa.17999.1.S1_at | HNRPLL | heterogeneous nuclear ribonucleoprotein L-like | 2.30E-08 | -3.08 |
| 2467 | Cfa.3247.1.S1_at | SPARCL1 | SPARC-like 1 (hevin) | 2.72E-07 | -3.09 |
| 2468 | Cfa.11257.1.A1_at | --- | --- | 3.08E-08 | -3.09 |
| 2469 | CfaAffx.10301.1.S1_at | SIGLEC1 | sialic acid binding Ig-like lectin 1, sialoadhesin | 0.0027591 | -3.09 |
| 2470 | CfaAffx.26739.1.S1_at | MPO | myeloperoxidase | 0.00453352 | -3.09 |
| 2471 | Cfa.10366.1.A1_at | NTRK2 | neurotrophic tyrosine kinase, receptor, type 2 | 8.64E-08 | -3.09 |
| 2472 | Cfa.6076.1.A1_at | --- | --- | 8.15E-07 | -3.09 |
| 2473 | Cfa.12271.1.A1_at | LOC480441 | similar to RAP2C, member of RAS oncogene family | 0.000389261 | -3.09 |
| 2474 | CfaAffx.27614.1.S1_s_at | GPRC5B | G protein-coupled receptor, family C, group 5, member B | 1.21E-06 | -3.10 |
| 2475 | Cfa.10312.1.A1_at | --- | --- | 5.48E-08 | -3.10 |
| 2476 | Cfa.14484.1.S1_at | RHOC | ras homolog gene family, member C | 5.90E-10 | -3.10 |
| 2477 | Cfa.16496.1.S1_at | --- | --- | 0.00071951 | -3.10 |
| 2478 | CfaAffx.5291.1.S1_s_at | LOC475166 | similar to immunoglobulin J chain | 0.000603085 | -3.10 |
| 2479 | CfaAffx.9754.1.S1_s_at | --- | --- | 1.24E-05 | -3.10 |
| 2480 | CfaAffx.28273.1.S1_at | LOC607188 | similar to Ig heavy chain V-III region VH26 precursor | 0.000416295 | -3.10 |
| 2481 | CfaAffx.24289.1.S1_s_at | AHNAK | AHNAK nucleoprotein | 9.93E-07 | -3.10 |
| 2482 | CfaAffx.7712.1.S1_s_at | SYTL2 | synaptotagmin-like 2 | 3.67E-15 | -3.10 |
| 2483 | Cfa.19787.1.S1_at | --- | --- | 1.58E-09 | -3.10 |
| 2484 | Cfa.11179.1.A1_at | --- | --- | 2.43E-11 | -3.10 |
| 2485 | Cfa.1838.1.A1_at | --- | --- | 6.95E-06 | -3.11 |
| 2486 | Cfa.11785.1.A1_at | --- | --- | 8.56E-08 | -3.11 |
| 2487 | Cfa.15576.1.A1_at | ICA1 | islet cell autoantigen 1, 69kDa | 8.38E-10 | -3.11 |
| 2488 | Cfa.10172.1.A1_at | --- | --- | 1.23E-07 | -3.11 |
| 2489 | Cfa.1966.1.A1_at | --- | --- | 6.18E-05 | -3.11 |
| 2490 | Cfa.9698.1.A1_at | --- | --- | 1.93E-06 | -3.11 |
| 2491 | Cfa.5773.1.A1_at | --- | --- | 0.014381 | -3.12 |
| 2492 | Cfa.12222.1.A1_at | NKG7 | natural killer cell group 7 sequence | 0.000656466 | -3.12 |
| 2493 | Cfa.1484.1.S1_at | PAM | peptidylglycine alpha-amidating monooxygenase | 9.48E-11 | -3.12 |
| 2494 | Cfa.9713.1.A1_at | --- | --- | 2.98E-09 | -3.12 |
| 2495 | Cfa.19608.1.S1_at | --- | --- | 7.14E-08 | -3.12 |
| 2496 | CfaAffx.1115.1.S1_at | --- | --- | 1.56E-07 | -3.12 |
| 2497 | Cfa.15783.1.A1_at | FBP2 | fructose-1,6-bisphosphatase 2 | 6.54E-05 | -3.12 |
| 2498 | CfaAffx.28402.1.S1_s_at | GHR | growth hormone receptor | 0.000137797 | -3.12 |
| 2499 | Cfa.11027.1.A1_at | LETM2 | Leucine zipper-EF-hand containing transmembrane protein 2 | 5.85E-05 | -3.12 |
| 2500 | Cfa.3011.1.A1_a_at | --- | --- | 0.000165498 | -3.13 |
| 2501 | Cfa.16947.1.A1_at | CH25H | cholesterol 25-hydroxylase | 1.46E-05 | -3.13 |
| 2502 | Cfa.15209.1.A1_at | SMOC1 | SPARC related modular calcium binding 1 | 0.00135702 | -3.13 |
| 2503 | Cfa.17153.1.S1_at | SYCE1 | synaptonemal complex central element protein 1 | 9.22E-06 | -3.13 |
| 2504 | CfaAffx.16400.1.S1_s_at | TARSL2 | threonyl-tRNA synthetase-like 2 | 4.33E-05 | -3.14 |
| 2505 | Cfa.16445.1.S1_at | --- | --- | 2.34E-09 | -3.14 |
| 2506 | Cfa.10974.1.S1_at | DBNDD2 | dysbindin (dystrobrevin binding protein 1) domain containing 2 | 8.97E-11 | -3.14 |
| 2507 | Cfa.3030.1.A1_at | --- | --- | 8.55E-07 | -3.14 |
| 2508 | Cfa.9054.1.A1_at | --- | --- | 2.33E-06 | -3.14 |
| 2509 | Cfa.20920.1.S1_s_at | SERPINB1 | serpin peptidase inhibitor, clade B (ovalbumin), member 1 | 1.14E-05 | -3.15 |
| 2510 | CfaAffx.6513.1.S1_s_at | AIM1 | absent in melanoma 1 | 0.000146291 | -3.15 |
| 2511 | CfaAffx.12485.1.S1_at | --- | --- | 0.000244711 | -3.15 |
| 2512 | Cfa.8104.2.S1_at | CARHSP1 | calcium regulated heat stable protein 1, 24kDa | 1.53E-08 | -3.15 |
| 2513 | CfaAffx.13545.1.S1_at | --- | --- | 2.71E-08 | -3.15 |
| 2514 | CfaAffx.11797.1.S1_s_at | ACOX2 | acyl-CoA oxidase 2, branched chain | 4.67E-08 | -3.15 |
| 2515 | Cfa.21287.1.S1_s_at | TMOD4 | tropomodulin 4 (muscle) | 2.56E-07 | -3.15 |
| 2516 | Cfa.14612.1.A1_at | --- | --- | 1.92E-08 | -3.15 |
| 2517 | CfaAffx.485.1.S1_x_at | --- | --- | 5.27E-09 | -3.16 |
| 2518 | CfaAffx.7002.1.S1_at | CABP5 | calcium binding protein 5 | 3.66E-08 | -3.16 |
| 2519 | CfaAffx.21028.1.S1_x_at | ZNF85 | zinc finger protein 85 | 1.16E-06 | -3.16 |
| 2520 | CfaAffx.4438.1.S1_at | FRMPD1 | FERM and PDZ domain containing 1 | 3.08E-08 | -3.16 |
| 2521 | CfaAffx.10633.1.S1_s_at | MAP4K3 | mitogen-activated protein kinase kinase kinase kinase 3 | 8.85E-08 | -3.16 |
| 2522 | Cfa.15373.1.A1_at | --- | --- | 2.25E-13 | -3.16 |
| 2523 | CfaAffx.3852.1.S1_s_at | CCL19 | chemokine (C-C motif) ligand 19 | 0.000101864 | -3.17 |
| 2524 | Cfa.9613.1.A1_at | --- | --- | 2.01E-07 | -3.17 |
| 2525 | Cfa.6686.1.A1_s_at | PHF1 | PHD finger protein 1 | 1.01E-09 | -3.17 |
| 2526 | Cfa.2706.1.S1_s_at | LIFR | leukemia inhibitory factor receptor alpha | 1.09E-06 | -3.17 |
| 2527 | CfaAffx.19910.1.S1_s_at | --- | --- | 9.56E-10 | -3.18 |
| 2528 | Cfa.6839.1.A1_at | --- | --- | 2.35E-11 | -3.18 |
| 2529 | Cfa.528.1.S1_at | --- | --- | 2.50E-06 | -3.18 |
| 2530 | Cfa.2704.1.A1_at | ST8SIA1 | ST8 alpha-N-acetyl-neuraminide alpha-2,8-sialyltransferase 1 | 7.62E-05 | -3.18 |
| 2531 | Cfa.5847.1.A1_at | --- | --- | 5.68E-06 | -3.18 |
| 2532 | CfaAffx.28268.1.S1_at | LOC607079 | similar to Ig heavy chain V-III region VH26 precursor | 4.53E-05 | -3.18 |
| 2533 | Cfa.11085.1.A1_at | RGS2 | regulator of G-protein signaling 2, 24kDa | 0.00145223 | -3.19 |
| 2534 | Cfa.1366.1.A1_at | --- | --- | 0.00774788 | -3.19 |
| 2535 | Cfa.7688.1.A1_s_at | RGS12 | regulator of G-protein signaling 12 | 7.72E-08 | -3.19 |
| 2536 | CfaAffx.3911.1.S1_at | --- | --- | 0.000591039 | -3.19 |
| 2537 | CfaAffx.8402.1.S1_s_at | PPARG | peroxisome proliferator-activated receptor gamma | 0.0002805 | -3.19 |
| 2538 | CfaAffx.11156.1.S1_at | RAD21L1 | RAD21-like 1 (S. pombe) | 6.91E-12 | -3.19 |
| 2539 | Cfa.5507.1.S1_s_at | IKZF2 | IKAROS family zinc finger 2 (Helios) | 1.38E-05 | -3.19 |
| 2540 | Cfa.13102.1.A1_at | MYOC | myocilin, trabecular meshwork inducible glucocorticoid response | 0.00996088 | -3.20 |
| 2541 | Cfa.19127.1.A1_at | --- | --- | 7.75E-09 | -3.20 |
| 2542 | Cfa.20271.1.S1_s_at | FGL1 | fibrinogen-like 1 | 6.09E-05 | -3.20 |
| 2543 | Cfa.7840.1.A1_at | CABP5 | calcium binding protein 5 | 7.54E-06 | -3.20 |
| 2544 | CfaAffx.10352.1.S1_at | --- | --- | 0.0067393 | -3.20 |
| 2545 | Cfa.12293.1.A1_s_at | NR2F2 | nuclear receptor subfamily 2, group F, member 2 | 2.95E-07 | -3.20 |
| 2546 | Cfa.13861.1.A1_s_at | FN3K | fructosamine 3 kinase | 1.24E-08 | -3.20 |
| 2547 | Cfa.12209.1.A1_a_at | --- | --- | 3.98E-07 | -3.20 |
| 2548 | Cfa.8381.1.A1_at | TRIP10 | thyroid hormone receptor interactor 10 | 5.76E-09 | -3.21 |
| 2549 | CfaAffx.18689.1.S1_s_at | SPO11 | SPO11 meiotic protein covalently bound to DSB homolog (S. cerevisiae) | 3.73E-09 | -3.21 |
| 2550 | CfaAffx.23018.1.S1_at | LOC490595 | similar to T-cell receptor alpha chain V region CTL-F3 precursor | 9.68E-13 | -3.21 |
| 2551 | Cfa.11151.1.S1_at | --- | --- | 3.15E-08 | -3.21 |
| 2552 | CfaAffx.21226.1.S1_s_at | AK3L1 /// LOC475238 /// LOC609761 /// LOC611161 /// LOC611170 | adenylate kinase 3-like 1 /// similar to ZK84.1 /// similar to Nascent polypepti | 0.00036561 | -3.21 |
| 2553 | Cfa.14245.1.A1_s_at | GPR110 | G protein-coupled receptor 110 | 7.50E-05 | -3.21 |
| 2554 | Cfa.9029.1.A1_at | --- | --- | 2.09E-05 | -3.21 |
| 2555 | CfaAffx.11118.1.S1_at | RASL11A | RAS-like, family 11, member A | 3.11E-05 | -3.22 |
| 2556 | Cfa.15823.1.S1_at | CCL24 | chemokine (C-C motif) ligand 24 | 0.000699913 | -3.22 |
| 2557 | CfaAffx.15043.1.S1_at | CLIC6 | chloride intracellular channel 6 | 9.31E-07 | -3.22 |
| 2558 | Cfa.14028.1.A1_at | HOXA4 | homeobox A4 | 5.86E-08 | -3.22 |
| 2559 | CfaAffx.13553.1.S1_at | SUCNR1 | succinate receptor 1 | 0.0051355 | -3.22 |
| 2560 | Cfa.1213.1.S1_s_at | NR4A1 | nuclear receptor subfamily 4, group A, member 1 | 0.000207514 | -3.22 |
| 2561 | Cfa.13651.1.A1_at | --- | --- | 6.50E-12 | -3.22 |
| 2562 | Cfa.17644.1.S1_s_at | RRAGD | Ras-related GTP binding D | 5.20E-07 | -3.22 |
| 2563 | Cfa.10773.1.A1_at | LOC477662 | hypothetical LOC477662 | 1.96E-05 | -3.23 |
| 2564 | Cfa.1849.1.A1_at | LOC487114 | similar to mannose receptor, C type 1-like 1 | 0.000236681 | -3.23 |
| 2565 | CfaAffx.579.1.S1_at | LOC607270 | similar to Ig heavy chain V-III region VH26 precursor | 7.76E-06 | -3.23 |
| 2566 | Cfa.8026.1.A1_at | PPP1R13B | protein phosphatase 1, regulatory (inhibitor) subunit 13B | 7.17E-11 | -3.23 |
| 2567 | Cfa.16368.1.S1_at | --- | --- | 1.57E-07 | -3.24 |
| 2568 | Cfa.9687.1.A1_at | --- | --- | 0.00173075 | -3.24 |
| 2569 | Cfa.9318.1.A1_at | CHMP4C | chromatin modifying protein 4C | 2.39E-06 | -3.24 |
| 2570 | Cfa.6936.1.A1_at | COMP | cartilage oligomeric matrix protein | 2.21E-05 | -3.24 |
| 2571 | Cfa.12485.1.A1_at | LOC609535 | hypothetical protein LOC609535 | 1.79E-08 | -3.24 |
| 2572 | CfaAffx.16441.1.S1_s_at | LOC488248 | similar to cytidine monophospho-N-acetylneuraminic acid hydroxylase | 6.78E-06 | -3.24 |
| 2573 | CfaAffx.28227.1.S1_at | --- | --- | 0.0119698 | -3.24 |
| 2574 | Cfa.506.1.S1_at | --- | --- | 9.08E-07 | -3.25 |
| 2575 | CfaAffx.13864.1.S1_at | --- | --- | 8.07E-16 | -3.25 |
| 2576 | Cfa.19085.1.S1_at | PRTFDC1 | phosphoribosyl transferase domain containing 1 | 6.59E-10 | -3.25 |
| 2577 | CfaAffx.23295.1.S1_s_at | LDB2 | LIM domain binding 2 | 3.56E-06 | -3.25 |
| 2578 | CfaAffx.16221.1.S1_at | CD96 | CD96 molecule | 6.58E-06 | -3.25 |
| 2579 | Cfa.20479.1.S1_at | KIF5C | kinesin family member 5C | 1.14E-07 | -3.26 |
| 2580 | Cfa.10935.1.A1_at | --- | --- | 0.000169803 | -3.26 |
| 2581 | Cfa.334.1.A1_s_at | KHDRBS3 | KH domain containing, RNA binding, signal transduction associated 3 | 1.70E-07 | -3.26 |
| 2582 | CfaAffx.15629.1.S1_s_at | STAT4 | signal transducer and activator of transcription 4 | 1.24E-07 | -3.26 |
| 2583 | Cfa.3990.1.A1_at | LOC608500 | similar to Protein C20orf103 precursor | 6.95E-11 | -3.26 |
| 2584 | Cfa.15172.1.A1_at | DOCK1 | dedicator of cytokinesis 1 | 5.50E-08 | -3.27 |
| 2585 | Cfa.5230.1.A1_s_at | NOSTRIN | nitric oxide synthase trafficker | 6.56E-09 | -3.27 |
| 2586 | Cfa.20618.1.S1_at | --- | --- | 7.83E-12 | -3.27 |
| 2587 | Cfa.11961.1.A1_s_at | ITGB7 | integrin, beta 7 | 3.47E-07 | -3.27 |
| 2588 | Cfa.9400.1.A1_at | ARAP3 | ArfGAP with RhoGAP domain, ankyrin repeat and PH domain 3 | 2.38E-08 | -3.27 |
| 2589 | Cfa.11274.1.A1_at | --- | --- | 5.56E-08 | -3.27 |
| 2590 | CfaAffx.15001.1.S1_s_at | SPARCL1 | SPARC-like 1 (hevin) | 2.02E-07 | -3.27 |
| 2591 | Cfa.14002.1.A1_at | --- | --- | 1.02E-08 | -3.27 |
| 2592 | CfaAffx.506.1.S1_x_at | --- | --- | 1.15E-08 | -3.27 |
| 2593 | Cfa.15610.1.A1_at | GPR116 | G protein-coupled receptor 116 | 1.64E-07 | -3.28 |
| 2594 | CfaAffx.28752.1.S1_at | CIDEA | cell death-inducing DFFA-like effector a | 0.015394 | -3.28 |
| 2595 | CfaAffx.13164.1.S1_s_at | --- | --- | 0.000431365 | -3.28 |
| 2596 | Cfa.7920.1.A1_s_at | LIN7A | lin-7 homolog A (C. elegans) | 3.86E-05 | -3.28 |
| 2597 | Cfa.3487.1.S1_s_at | ADIPOQ | adiponectin, C1Q and collagen domain containing | 0.0144611 | -3.28 |
| 2598 | Cfa.21340.1.S1_at | SVIP | small VCP/p97-interacting protein | 1.74E-05 | -3.28 |
| 2599 | CfaAffx.20584.1.S1_s_at | --- | --- | 9.96E-07 | -3.28 |
| 2600 | Cfa.18930.1.S1_s_at | CFP | complement factor properdin | 2.60E-08 | -3.28 |
| 2601 | Cfa.2532.1.A1_at | --- | --- | 5.87E-05 | -3.28 |
| 2602 | Cfa.2365.1.A1_at | --- | --- | 1.95E-05 | -3.29 |
| 2603 | Cfa.10658.1.A1_at | --- | --- | 0.0159182 | -3.29 |
| 2604 | Cfa.2921.1.S1_at | MTMR11 | myotubularin related protein 11 | 6.13E-08 | -3.29 |
| 2605 | Cfa.2585.1.A1_at | LOC611894 | hypothetical protein LOC611894 | 3.48E-09 | -3.29 |
| 2606 | Cfa.17587.1.S1_at | PALMD | palmdelphin | 2.73E-06 | -3.29 |
| 2607 | Cfa.10696.1.A1_at | --- | --- | 9.74E-05 | -3.29 |
| 2608 | Cfa.14425.1.S1_at | HPN | hepsin | 6.80E-05 | -3.30 |
| 2609 | Cfa.20795.1.S1_at | LOC608476 | similar to Fatty acid-binding protein, adipocyte (AFABP) (Adipocyte lipid-bindin | 0.00259149 | -3.30 |
| 2610 | Cfa.20323.1.S1_at | --- | --- | 1.15E-09 | -3.31 |
| 2611 | Cfa.6177.1.S1_s_at | SULT2B1 | sulfotransferase family, cytosolic, 2B, member 1 | 2.48E-06 | -3.31 |
| 2612 | Cfa.5585.1.A1_a_at | LOC609535 | hypothetical protein LOC609535 | 1.02E-08 | -3.32 |
| 2613 | CfaAffx.6377.1.S1_at | INPP4B | inositol polyphosphate-4-phosphatase, type II, 105kDa | 8.84E-11 | -3.32 |
| 2614 | CfaAffx.25181.1.S1_s_at | CD5L | CD5 molecule-like | 0.000422316 | -3.33 |
| 2615 | Cfa.12195.8.A1_at | --- | --- | 0.0016459 | -3.33 |
| 2616 | CfaAffx.22549.1.S1_s_at | COMP | cartilage oligomeric matrix protein | 2.69E-05 | -3.34 |
| 2617 | Cfa.5230.1.A1_at | NOSTRIN | nitric oxide synthase trafficker | 8.99E-09 | -3.34 |
| 2618 | CfaAffx.7934.1.S1_at | TNFSF11 | tumor necrosis factor (ligand) superfamily, member 11 | 6.61E-07 | -3.34 |
| 2619 | Cfa.4598.1.A1_at | --- | --- | 4.29E-11 | -3.35 |
| 2620 | CfaAffx.26485.1.S1_s_at | ZNF564 | zinc finger protein 564 | 1.01E-09 | -3.35 |
| 2621 | Cfa.12254.1.A1_s_at | CPO | carboxypeptidase O | 1.07E-12 | -3.35 |
| 2622 | CfaAffx.19962.1.S1_s_at | NMB | neuromedin B | 1.35E-10 | -3.35 |
| 2623 | Cfa.1817.1.A1_at | --- | --- | 1.89E-06 | -3.36 |
| 2624 | Cfa.15060.1.A1_a_at | MICAL2 | microtubule associated monoxygenase, calponin and LIM domain containing 2 | 3.85E-07 | -3.36 |
| 2625 | CfaAffx.19295.1.S1_s_at | CMA1 | chymase 1, mast cell | 0.00040356 | -3.36 |
| 2626 | Cfa.8496.1.A1_at | ITIH3 | inter-alpha (globulin) inhibitor H3 | 3.03E-06 | -3.36 |
| 2627 | CfaAffx.21020.1.S1_at | --- | --- | 1.32E-07 | -3.36 |
| 2628 | CfaAffx.3697.1.S1_at | STEAP4 | STEAP family member 4 | 1.31E-08 | -3.37 |
| 2629 | CfaAffx.14757.1.S1_s_at | PYROXD2 | pyridine nucleotide-disulphide oxidoreductase domain 2 | 2.29E-10 | -3.38 |
| 2630 | Cfa.5013.1.A1_at | --- | --- | 2.77E-05 | -3.38 |
| 2631 | CfaAffx.24497.1.S1_s_at | CLEC10A | C-type lectin domain family 10, member A | 1.64E-05 | -3.39 |
| 2632 | CfaAffx.17630.1.S1_s_at | LOC611813 | similar to double C2, gamma (predicted) | 6.62E-13 | -3.39 |
| 2633 | CfaAffx.6579.1.S1_at | --- | --- | 5.95E-07 | -3.39 |
| 2634 | CfaAffx.13435.1.S1_s_at | JAM2 | junctional adhesion molecule 2 | 5.92E-07 | -3.40 |
| 2635 | Cfa.3748.1.S2_at | TRIB2 | tribbles homolog 2 (Drosophila) | 0.000121088 | -3.40 |
| 2636 | Cfa.5525.1.A1_at | --- | --- | 2.88E-10 | -3.40 |
| 2637 | Cfa.9396.1.A1_s_at | VLDLR | very low density lipoprotein receptor | 7.32E-05 | -3.41 |
| 2638 | Cfa.13203.1.S1_at | HNMT | histamine N-methyltransferase | 2.72E-09 | -3.41 |
| 2639 | Cfa.19233.1.S1_at | LOC611878 | similar to Y51H7BR.7 | 2.57E-13 | -3.41 |
| 2640 | Cfa.1291.1.S1_at | --- | --- | 2.97E-20 | -3.41 |
| 2641 | CfaAffx.26854.1.S1_at | S100A9 | S100 calcium binding protein A9 | 0.00765596 | -3.41 |
| 2642 | Cfa.7153.1.A1_s_at | BMP2 | bone morphogenetic protein 2 | 2.59E-06 | -3.42 |
| 2643 | CfaAffx.17699.1.S1_at | VWA5A | von Willebrand factor A domain containing 5A | 6.68E-09 | -3.42 |
| 2644 | CfaAffx.26834.1.S1_s_at | --- | --- | 0.00997038 | -3.42 |
| 2645 | CfaAffx.3150.1.S1_at | MPDZ | multiple PDZ domain protein | 4.14E-09 | -3.42 |
| 2646 | CfaAffx.1722.1.S1_s_at | SYNE1 | spectrin repeat containing, nuclear envelope 1 | 9.63E-08 | -3.43 |
| 2647 | CfaAffx.28389.1.S1_s_at | SGIP1 | SH3-domain GRB2-like (endophilin) interacting protein 1 | 8.18E-10 | -3.43 |
| 2648 | CfaAffx.225.1.S1_x_at | LOC486374 /// LOC486377 /// LOC486386 /// LOC486387 /// LOC486389 /// LOC491362 /// LOC491364 /// LOC491454 /// LOC606893 /// LOC606941 /// LOC606953 /// LOC612050 /// LOC612054 /// LOC612081 /// LOC612128 /// LOC612135 | similar to Ig lambda chain V-I region BL2 precursor /// similar to Ig lambda cha | 0.0171259 | -3.43 |
| 2649 | Cfa.9524.1.A1_at | --- | --- | 0.00422832 | -3.43 |
| 2650 | Cfa.16431.1.A1_at | --- | --- | 3.52E-06 | -3.43 |
| 2651 | Cfa.10197.1.A1_at | PNMA1 | paraneoplastic antigen MA1 | 2.11E-09 | -3.43 |
| 2652 | CfaAffx.18903.1.S1_s_at | RGS10 | regulator of G-protein signaling 10 | 3.68E-09 | -3.43 |
| 2653 | CfaAffx.484.1.S1_x_at | --- | --- | 1.86E-10 | -3.44 |
| 2654 | Cfa.12164.1.A1_at | PLA2G16 | phospholipase A2, group XVI | 1.68E-06 | -3.44 |
| 2655 | Cfa.3906.1.S1_at | ABCA4 | ATP-binding cassette, sub-family A (ABC1), member 4 | 2.80E-06 | -3.44 |
| 2656 | Cfa.10150.1.A1_at | --- | --- | 5.86E-07 | -3.44 |
| 2657 | CfaAffx.6095.1.S1_at | --- | --- | 4.13E-07 | -3.44 |
| 2658 | Cfa.1903.1.A1_a_at | --- | --- | 9.20E-12 | -3.45 |
| 2659 | CfaAffx.16997.1.S1_at | LOC608130 | hypothetical protein LOC608130 | 3.76E-09 | -3.45 |
| 2660 | Cfa.5549.1.A1_at | --- | --- | 6.91E-07 | -3.45 |
| 2661 | Cfa.9680.1.A1_at | --- | --- | 1.54E-12 | -3.46 |
| 2662 | Cfa.20055.1.A1_at | NSUN7 | NOL1/NOP2/Sun domain family, member 7 | 5.80E-08 | -3.46 |
| 2663 | CfaAffx.26832.1.S1_s_at | --- | --- | 0.0100421 | -3.46 |
| 2664 | Cfa.4404.1.S1_at | --- | --- | 7.63E-09 | -3.46 |
| 2665 | Cfa.7409.1.S1_at | GLT8D2 | glycosyltransferase 8 domain containing 2 | 4.81E-07 | -3.46 |
| 2666 | Cfa.14923.1.A1_s_at | SNTB2 | syntrophin, beta 2 (dystrophin-associated protein A1, 59kDa, basic component 2) | 1.92E-07 | -3.46 |
| 2667 | Cfa.11437.1.A1_at | SAT2 | spermidine/spermine N1-acetyltransferase family member 2 | 1.11E-07 | -3.47 |
| 2668 | Cfa.12383.1.A1_at | --- | --- | 8.83E-06 | -3.47 |
| 2669 | Cfa.70.1.A1_s_at | SULT1A1 | sulfotransferase family, cytosolic, 1A, phenol-preferring, member 1 | 3.82E-10 | -3.47 |
| 2670 | Cfa.13710.1.S1_at | --- | --- | 4.54E-06 | -3.47 |
| 2671 | CfaAffx.26522.1.S1_at | LAT | linker for activation of T cells | 2.37E-11 | -3.47 |
| 2672 | Cfa.2828.1.A1_at | --- | --- | 7.11E-09 | -3.48 |
| 2673 | Cfa.2147.1.A1_at | --- | --- | 4.27E-08 | -3.48 |
| 2674 | CfaAffx.22356.1.S1_s_at | EFHC2 | EF-hand domain (C-terminal) containing 2 | 6.40E-11 | -3.48 |
| 2675 | CfaAffx.11744.1.S1_at | LRRC70 | leucine rich repeat containing 70 | 3.22E-09 | -3.48 |
| 2676 | CfaAffx.13394.1.S1_s_at | EPHX2 | epoxide hydrolase 2, cytoplasmic | 6.56E-05 | -3.48 |
| 2677 | Cfa.19.2.A1_at | LOC482284 | similar to Multidrug resistance protein 3 (P-glycoprotein 3) | 1.16E-05 | -3.49 |
| 2678 | CfaAffx.16330.1.S1_s_at | CPNE8 | copine VIII | 2.15E-05 | -3.49 |
| 2679 | Cfa.9027.1.A1_at | --- | --- | 4.89E-05 | -3.49 |
| 2680 | Cfa.2436.1.S1_at | GHR | growth hormone receptor | 4.06E-05 | -3.49 |
| 2681 | Cfa.5419.1.A1_s_at | FAIM2 | Fas apoptotic inhibitory molecule 2 | 1.39E-12 | -3.50 |
| 2682 | Cfa.18258.3.S1_at | LOC607380 | similar to downregulated in renal cell carcinoma | 1.86E-07 | -3.50 |
| 2683 | Cfa.8590.1.A1_at | ALOX12 | arachidonate 12-lipoxygenase | 6.92E-07 | -3.50 |
| 2684 | Cfa.15588.1.A1_at | --- | --- | 4.36E-10 | -3.50 |
| 2685 | Cfa.12428.1.A1_at | --- | --- | 6.19E-07 | -3.51 |
| 2686 | CfaAffx.2070.1.S1_at | PRRT1 | proline-rich transmembrane protein 1 | 2.26E-07 | -3.51 |
| 2687 | Cfa.6163.1.A1_at | --- | --- | 2.87E-09 | -3.51 |
| 2688 | CfaAffx.28273.1.S1_x_at | LOC607188 | similar to Ig heavy chain V-III region VH26 precursor | 4.79E-05 | -3.51 |
| 2689 | Cfa.14907.1.A1_at | --- | --- | 1.83E-09 | -3.51 |
| 2690 | CfaAffx.10319.1.S1_s_at | HNRPLL | heterogeneous nuclear ribonucleoprotein L-like | 2.48E-07 | -3.52 |
| 2691 | Cfa.15602.1.A1_at | --- | --- | 1.67E-09 | -3.52 |
| 2692 | Cfa.10053.1.A1_at | --- | --- | 2.17E-07 | -3.52 |
| 2693 | CfaAffx.21286.1.S1_s_at | LTF | lactotransferrin | 0.00305719 | -3.53 |
| 2694 | Cfa.12293.1.A1_at | NR2F2 | nuclear receptor subfamily 2, group F, member 2 | 2.02E-08 | -3.53 |
| 2695 | CfaAffx.6423.1.S1_at | KEL | Kell blood group, metallo-endopeptidase | 8.18E-11 | -3.54 |
| 2696 | CfaAffx.26487.1.S1_s_at | ZNF564 | zinc finger protein 564 | 2.11E-09 | -3.54 |
| 2697 | CfaAffx.29964.1.S1_at | TNFRSF25 | tumor necrosis factor receptor superfamily, member 25 | 4.39E-14 | -3.54 |
| 2698 | Cfa.12063.1.A1_at | SLC4A11 | solute carrier family 4, sodium borate transporter, member 11 | 0.00031972 | -3.54 |
| 2699 | Cfa.5650.1.A1_at | KCNJ8 | potassium inwardly-rectifying channel, subfamily J, member 8 | 2.79E-13 | -3.54 |
| 2700 | Cfa.9973.1.A1_at | AQP5 | aquaporin 5 | 1.20E-12 | -3.54 |
| 2701 | Cfa.11570.1.A1_at | LIFR | leukemia inhibitory factor receptor alpha | 1.72E-07 | -3.54 |
| 2702 | CfaAffx.10318.1.S1_at | HNRPLL | heterogeneous nuclear ribonucleoprotein L-like | 3.99E-08 | -3.55 |
| 2703 | Cfa.6079.1.A1_at | --- | --- | 2.05E-11 | -3.55 |
| 2704 | CfaAffx.20727.1.S1_s_at | LOC611161 | hypothetical protein LOC611161 | 5.56E-05 | -3.56 |
| 2705 | CfaAffx.2043.1.S1_at | SLC22A3 | solute carrier family 22 (extraneuronal monoamine transporter), member 3 | 1.04E-08 | -3.56 |
| 2706 | Cfa.7626.1.S1_s_at | SPATA6 | spermatogenesis associated 6 | 2.82E-09 | -3.56 |
| 2707 | CfaAffx.14695.1.S1_at | STAB1 | stabilin 1 | 1.25E-07 | -3.56 |
| 2708 | Cfa.11961.1.A1_at | ITGB7 | integrin, beta 7 | 2.12E-07 | -3.56 |
| 2709 | CfaAffx.2463.1.S1_s_at | MTSS1 | metastasis suppressor 1 | 1.09E-05 | -3.56 |
| 2710 | Cfa.11022.1.A1_at | --- | --- | 0.000537551 | -3.56 |
| 2711 | CfaAffx.4040.1.S1_s_at | KDR | kinase insert domain receptor (a type III receptor tyrosine kinase) | 3.57E-08 | -3.56 |
| 2712 | CfaAffx.15259.1.S1_at | ALCAM | activated leukocyte cell adhesion molecule | 3.42E-08 | -3.57 |
| 2713 | Cfa.13284.1.A1_at | LIPC | lipase, hepatic | 1.83E-05 | -3.57 |
| 2714 | Cfa.8991.1.A1_at | --- | --- | 9.64E-07 | -3.57 |
| 2715 | CfaAffx.10713.1.S1_at | RBPMS | RNA binding protein with multiple splicing | 5.88E-09 | -3.57 |
| 2716 | Cfa.2935.1.A1_at | --- | --- | 1.99E-06 | -3.57 |
| 2717 | Cfa.1808.1.S1_at | --- | --- | 1.14E-08 | -3.57 |
| 2718 | Cfa.20631.1.S1_at | GPRC5C | G protein-coupled receptor, family C, group 5, member C | 4.26E-12 | -3.57 |
| 2719 | Cfa.2354.1.A1_at | FGF13 | fibroblast growth factor 13 | 1.81E-10 | -3.58 |
| 2720 | Cfa.688.1.S1_at | --- | --- | 1.42E-05 | -3.58 |
| 2721 | Cfa.6352.1.A1_at | SSPO | SCO-spondin homolog (Bos taurus) | 3.73E-07 | -3.58 |
| 2722 | Cfa.6358.1.A1_at | --- | --- | 1.39E-07 | -3.58 |
| 2723 | Cfa.15489.1.S1_at | RBP4 | retinol binding protein 4, plasma | 0.00408149 | -3.59 |
| 2724 | CfaAffx.5550.1.S1_s_at | LOC481674 | similar to alpha-2u globulin PGCL4 | 3.86E-07 | -3.59 |
| 2725 | Cfa.10205.1.A1_at | --- | --- | 3.86E-11 | -3.59 |
| 2726 | Cfa.2366.1.A1_at | ANK3 | ankyrin 3, node of Ranvier (ankyrin G) | 1.03E-08 | -3.59 |
| 2727 | Cfa.16786.1.S1_at | RRAGD | Ras-related GTP binding D | 2.90E-08 | -3.60 |
| 2728 | Cfa.10235.1.A1_at | --- | --- | 4.78E-05 | -3.62 |
| 2729 | Cfa.5010.1.A1_at | --- | --- | 2.93E-09 | -3.62 |
| 2730 | Cfa.15010.1.A1_at | --- | --- | 2.59E-06 | -3.62 |
| 2731 | CfaAffx.19834.1.S1_s_at | ANK3 | ankyrin 3, node of Ranvier (ankyrin G) | 2.16E-09 | -3.62 |
| 2732 | Cfa.838.1.A1_at | CD59 | CD59 molecule, complement regulatory protein | 5.58E-06 | -3.62 |
| 2733 | CfaAffx.24915.1.S1_at | EMR2 | egf-like module containing, mucin-like, hormone receptor-like 2 | 0.000222343 | -3.62 |
| 2734 | CfaAffx.2644.1.S1_at | KHDRBS3 | KH domain containing, RNA binding, signal transduction associated 3 | 2.64E-06 | -3.62 |
| 2735 | Cfa.9956.1.A1_at | --- | --- | 2.78E-07 | -3.62 |
| 2736 | CfaAffx.10578.1.S1_at | CD36 | CD36 molecule (thrombospondin receptor) | 0.00858191 | -3.63 |
| 2737 | CfaAffx.9808.1.S1_at | BMP2 | bone morphogenetic protein 2 | 7.59E-07 | -3.63 |
| 2738 | Cfa.9077.1.A1_at | --- | --- | 2.43E-07 | -3.63 |
| 2739 | CfaAffx.21279.1.S1_s_at | DMD | dystrophin (muscular dystrophy, Duchenne and Becker types) | 2.09E-09 | -3.64 |
| 2740 | Cfa.3557.1.S1_s_at | CTLA4 | cytotoxic T-lymphocyte-associated protein 4 | 5.00E-12 | -3.64 |
| 2741 | Cfa.5820.1.A1_at | --- | --- | 6.88E-08 | -3.64 |
| 2742 | Cfa.9467.1.A1_at | RELN | reelin | 3.92E-07 | -3.65 |
| 2743 | CfaAffx.15063.1.S1_s_at | --- | --- | 3.00E-05 | -3.65 |
| 2744 | CfaAffx.5988.1.S1_at | ZNF30 | zinc finger protein 30 | 2.71E-15 | -3.65 |
| 2745 | Cfa.10265.1.S1_at | --- | --- | 3.02E-07 | -3.65 |
| 2746 | Cfa.10601.2.S1_a_at | TNFAIP8L3 | tumor necrosis factor, alpha-induced protein 8-like 3 | 8.05E-06 | -3.65 |
| 2747 | CfaAffx.8731.1.S1_s_at | HYI | hydroxypyruvate isomerase homolog (E. coli) | 4.92E-10 | -3.65 |
| 2748 | CfaAffx.3829.1.S1_s_at | TXK | TXK tyrosine kinase | 2.57E-12 | -3.66 |
| 2749 | Cfa.12050.1.A1_a_at | TSPAN5 | tetraspanin 5 | 1.15E-12 | -3.66 |
| 2750 | Cfa.2520.1.A1_at | RIC8B | resistance to inhibitors of cholinesterase 8 homolog B (C. elegans) | 1.74E-06 | -3.66 |
| 2751 | Cfa.8598.1.A1_at | ITIH1 | inter-alpha (globulin) inhibitor H1 | 6.55E-09 | -3.67 |
| 2752 | Cfa.4285.1.A1_at | --- | --- | 3.11E-06 | -3.67 |
| 2753 | Cfa.15533.1.A1_at | ANXA9 | annexin A9 | 5.38E-07 | -3.67 |
| 2754 | Cfa.19665.1.A1_at | PLCL1 | phospholipase C-like 1 | 1.44E-07 | -3.67 |
| 2755 | Cfa.1266.1.A1_at | JAM2 | junctional adhesion molecule 2 | 7.09E-09 | -3.67 |
| 2756 | Cfa.21035.1.S1_at | LOC607913 | hypothetical protein LOC607913 | 0.000273628 | -3.68 |
| 2757 | Cfa.14728.1.A1_s_at | --- | --- | 6.78E-07 | -3.68 |
| 2758 | CfaAffx.24961.1.S1_at | LOC488839 | hypothetical LOC488839 | 6.08E-06 | -3.68 |
| 2759 | CfaAffx.26710.1.S1_s_at | LOC608881 | similar to protocadherin 11 X-linked isoform a precursor | 0.00528749 | -3.68 |
| 2760 | Cfa.5127.1.A1_s_at | RUNX1T1 | runt-related transcription factor 1; translocated to, 1 (cyclin D-related) | 2.14E-06 | -3.69 |
| 2761 | CfaAffx.21613.1.S1_at | ANGPTL1 | angiopoietin-like 1 | 4.70E-07 | -3.69 |
| 2762 | CfaAffx.16279.1.S1_s_at | UBASH3A | ubiquitin associated and SH3 domain containing A | 4.91E-08 | -3.69 |
| 2763 | CfaAffx.1754.1.S1_s_at | RGS17 | regulator of G-protein signaling 17 | 3.70E-09 | -3.69 |
| 2764 | Cfa.1856.1.S1_at | --- | --- | 9.75E-14 | -3.69 |
| 2765 | Cfa.7827.1.A1_at | --- | --- | 4.23E-06 | -3.69 |
| 2766 | Cfa.3685.4.S1_at | LOC480788 /// LOC608959 | similar to T-cell receptor beta chain C region /// similar to T-cell receptor be | 1.94E-10 | -3.70 |
| 2767 | CfaAffx.26852.1.S1_at | S100A8 | S100 calcium binding protein A8 | 0.00601873 | -3.70 |
| 2768 | CfaAffx.26459.1.S1_at | --- | --- | 7.06E-11 | -3.70 |
| 2769 | Cfa.2966.1.S1_at | LTBP1 | latent transforming growth factor beta binding protein 1 | 7.53E-05 | -3.70 |
| 2770 | Cfa.4307.1.S1_at | NR2F1 | nuclear receptor subfamily 2, group F, member 1 | 1.45E-08 | -3.70 |
| 2771 | Cfa.6471.1.A1_at | AKR1E2 | aldo-keto reductase family 1, member E2 | 1.06E-09 | -3.70 |
| 2772 | Cfa.12477.1.A1_at | --- | --- | 1.57E-06 | -3.71 |
| 2773 | Cfa.12518.1.A1_at | --- | --- | 1.19E-05 | -3.71 |
| 2774 | CfaAffx.11071.1.S1_at | NPHS1 | nephrosis 1, congenital, Finnish type (nephrin) | 1.16E-05 | -3.71 |
| 2775 | CfaAffx.13996.1.S1_s_at | CPE | carboxypeptidase E | 6.75E-08 | -3.72 |
| 2776 | Cfa.16336.1.S1_at | CMA1 | chymase 1, mast cell | 8.99E-05 | -3.72 |
| 2777 | CfaAffx.28209.1.S1_at | ITGA1 | integrin, alpha 1 | 7.99E-12 | -3.72 |
| 2778 | Cfa.770.1.S1_s_at | FMO3 | flavin containing monooxygenase 3 | 1.22E-08 | -3.72 |
| 2779 | Cfa.11568.1.A1_at | --- | --- | 3.33E-08 | -3.72 |
| 2780 | CfaAffx.16623.1.S1_at | --- | --- | 2.19E-08 | -3.72 |
| 2781 | CfaAffx.18936.1.S1_at | KCNJ8 | potassium inwardly-rectifying channel, subfamily J, member 8 | 4.57E-09 | -3.72 |
| 2782 | Cfa.15235.1.S1_at | ITGA5 | integrin, alpha 5 (fibronectin receptor, alpha polypeptide) | 1.39E-07 | -3.73 |
| 2783 | Cfa.11857.1.A1_at | --- | --- | 2.98E-09 | -3.73 |
| 2784 | Cfa.10175.1.A1_at | --- | --- | 3.36E-09 | -3.73 |
| 2785 | Cfa.19188.1.A1_at | MAGEH1 | melanoma antigen family H, 1 | 3.40E-07 | -3.73 |
| 2786 | Cfa.3596.2.S1_s_at | CD80 | CD80 molecule | 1.21E-10 | -3.74 |
| 2787 | Cfa.3299.1.A1_at | --- | --- | 6.42E-06 | -3.75 |
| 2788 | CfaAffx.14648.1.S1_at | CRTAC1 | cartilage acidic protein 1 | 8.41E-12 | -3.75 |
| 2789 | Cfa.2443.1.A1_at | GPRC5B | G protein-coupled receptor, family C, group 5, member B | 1.23E-08 | -3.75 |
| 2790 | CfaAffx.4502.1.S1_at | LOC484264 | similar to zinc finger protein 91 (HPF7, HTF10) | 1.41E-07 | -3.75 |
| 2791 | Cfa.2507.1.S1_at | --- | --- | 5.55E-12 | -3.76 |
| 2792 | Cfa.14.1.A1_at | LAMA3 | laminin, alpha 3 | 1.22E-08 | -3.77 |
| 2793 | CfaAffx.4729.1.S1_at | TMEFF1 | transmembrane protein with EGF-like and two follistatin-like domains 1 | 5.41E-05 | -3.77 |
| 2794 | Cfa.12081.1.A1_at | --- | --- | 3.79E-07 | -3.77 |
| 2795 | Cfa.2353.1.A1_at | --- | --- | 3.36E-05 | -3.77 |
| 2796 | Cfa.2364.1.A1_at | --- | --- | 5.64E-15 | -3.78 |
| 2797 | CfaAffx.18903.1.S1_at | RGS10 | regulator of G-protein signaling 10 | 9.09E-10 | -3.78 |
| 2798 | CfaAffx.19298.1.S1_at | CTSG | cathepsin G | 0.000371362 | -3.78 |
| 2799 | Cfa.15132.1.A1_at | --- | --- | 3.06E-11 | -3.79 |
| 2800 | Cfa.15702.1.S1_at | --- | --- | 8.24E-06 | -3.79 |
| 2801 | CfaAffx.21459.1.S1_at | AICDA | activation-induced cytidine deaminase | 0.00022059 | -3.79 |
| 2802 | Cfa.12975.1.A1_at | --- | --- | 5.11E-08 | -3.80 |
| 2803 | Cfa.15076.2.A1_s_at | VTN | vitronectin | 3.30E-08 | -3.81 |
| 2804 | Cfa.14286.1.A1_at | --- | --- | 7.78E-08 | -3.81 |
| 2805 | Cfa.9441.1.A1_at | --- | --- | 4.13E-09 | -3.82 |
| 2806 | Cfa.272.1.A1_at | --- | --- | 2.18E-13 | -3.82 |
| 2807 | Cfa.6145.1.A1_at | SLC26A11 | solute carrier family 26, member 11 | 5.43E-10 | -3.83 |
| 2808 | Cfa.16244.1.A1_at | RUNX2 | runt-related transcription factor 2 | 3.17E-07 | -3.83 |
| 2809 | Cfa.18338.1.S1_at | LOC611571 | similar to T-cell receptor zeta chain isoform 2 precursor | 2.82E-07 | -3.83 |
| 2810 | CfaAffx.19116.1.S1_at | ANXA9 | annexin A9 | 3.46E-06 | -3.84 |
| 2811 | Cfa.4791.1.A1_at | GSTT1 | glutathione S-transferase theta 1 | 3.16E-06 | -3.85 |
| 2812 | Cfa.7326.1.A1_s_at | SOX30 | SRY (sex determining region Y)-box 30 | 0.000455395 | -3.85 |
| 2813 | Cfa.17088.1.A1_s_at | LOC475615 | similar to mitochondrial tumor suppressor 1 isoform 4 | 2.80E-08 | -3.86 |
| 2814 | Cfa.11205.1.A1_at | --- | --- | 4.63E-07 | -3.86 |
| 2815 | Cfa.6136.1.A1_at | ROBO3 | roundabout, axon guidance receptor, homolog 3 (Drosophila) | 1.43E-10 | -3.86 |
| 2816 | CfaAffx.30566.1.S1_s_at | LOC607460 | similar to Pancreatic alpha-amylase precursor (PA) (1,4-alpha-D-glucan glucanohy | 4.18E-06 | -3.87 |
| 2817 | Cfa.12777.1.A1_at | --- | --- | 2.03E-07 | -3.87 |
| 2818 | CfaAffx.26833.1.S1_s_at | S100A2 | S100 calcium binding protein A2 | 1.27E-06 | -3.87 |
| 2819 | CfaAffx.4577.1.S1_at | KHDRBS2 | KH domain containing, RNA binding, signal transduction associated 2 | 8.30E-09 | -3.87 |
| 2820 | Cfa.9472.1.A1_at | --- | --- | 2.25E-07 | -3.88 |
| 2821 | Cfa.4178.1.S1_at | AIM1 | absent in melanoma 1 | 0.000166705 | -3.88 |
| 2822 | CfaAffx.29513.1.S1_at | TNFRSF4 | tumor necrosis factor receptor superfamily, member 4 | 3.34E-11 | -3.89 |
| 2823 | Cfa.15200.1.S1_at | TMEM55A | transmembrane protein 55A | 2.47E-10 | -3.89 |
| 2824 | Cfa.15594.1.A1_at | --- | --- | 9.79E-06 | -3.90 |
| 2825 | Cfa.9459.1.A1_s_at | BCL6 | B-cell CLL/lymphoma 6 | 2.19E-08 | -3.90 |
| 2826 | CfaAffx.14166.1.S1_at | LOC608621 | hypothetical protein LOC608621 | 2.16E-14 | -3.91 |
| 2827 | CfaAffx.23816.1.S1_s_at | NLGN1 | neuroligin 1 | 8.60E-11 | -3.92 |
| 2828 | Cfa.20346.1.S1_at | --- | --- | 8.95E-09 | -3.94 |
| 2829 | Cfa.4845.1.A1_at | THSD1 | thrombospondin, type I, domain containing 1 | 2.33E-07 | -3.94 |
| 2830 | CfaAffx.1769.1.S1_at | LOC474456 | similar to SH3 and multiple ankyrin repeat domains 3 (Shank3) (Proline-rich syna | 7.72E-13 | -3.94 |
| 2831 | Cfa.2657.1.A1_at | --- | --- | 1.29E-05 | -3.95 |
| 2832 | CfaAffx.23305.1.S1_at | CD27 | CD27 molecule | 4.16E-12 | -3.95 |
| 2833 | Cfa.21214.1.S1_s_at | IL7R | interleukin 7 receptor | 2.60E-05 | -3.95 |
| 2834 | Cfa.15563.1.A1_at | --- | --- | 3.94E-11 | -3.96 |
| 2835 | Cfa.11290.1.A1_s_at | TLE1 | transducin-like enhancer of split 1 (E(sp1) homolog, Drosophila) | 1.11E-07 | -3.96 |
| 2836 | CfaAffx.15167.1.S1_s_at | SLPI | secretory leukocyte peptidase inhibitor | 0.0202331 | -3.96 |
| 2837 | Cfa.14035.1.A1_at | --- | --- | 1.31E-08 | -3.98 |
| 2838 | Cfa.15037.1.A1_at | --- | --- | 3.34E-05 | -3.99 |
| 2839 | Cfa.10006.1.A1_at | --- | --- | 8.01E-09 | -4.00 |
| 2840 | Cfa.10287.1.S1_at | --- | --- | 1.45E-07 | -4.00 |
| 2841 | CfaAffx.28287.1.S1_s_at | MYOM1 | myomesin 1, 185kDa | 3.28E-07 | -4.00 |
| 2842 | Cfa.9116.1.A1_at | --- | --- | 1.83E-10 | -4.00 |
| 2843 | Cfa.651.1.S1_at | BCAR3 | breast cancer anti-estrogen resistance 3 | 1.07E-07 | -4.01 |
| 2844 | CfaAffx.21926.1.S1_s_at | LOC491462 /// LOC610966 | similar to zinc finger protein 154 (pHZ-92) /// similar to zinc finger protein 1 | 5.04E-06 | -4.02 |
| 2845 | Cfa.11187.1.A1_at | --- | --- | 8.14E-11 | -4.03 |
| 2846 | Cfa.14944.1.A1_at | AMT | aminomethyltransferase | 5.25E-10 | -4.04 |
| 2847 | Cfa.1637.1.S1_s_at | SSBP2 | single-stranded DNA binding protein 2 | 3.76E-07 | -4.04 |
| 2848 | Cfa.9206.1.A1_at | --- | --- | 0.000118956 | -4.05 |
| 2849 | Cfa.12642.1.A1_at | --- | --- | 0.00871086 | -4.05 |
| 2850 | Cfa.18826.1.S1_at | RGS1 | regulator of G-protein signaling 1 | 5.13E-05 | -4.05 |
| 2851 | Cfa.4713.1.A1_at | --- | --- | 3.37E-10 | -4.05 |
| 2852 | Cfa.9052.1.A1_s_at | --- | --- | 6.26E-10 | -4.06 |
| 2853 | CfaAffx.28126.1.S1_at | GZMK | granzyme K (granzyme 3; tryptase II) | 0.000590345 | -4.06 |
| 2854 | CfaAffx.15515.1.S1_s_at | MMRN1 | multimerin 1 | 0.00583957 | -4.06 |
| 2855 | Cfa.1493.1.A1_at | FHL2 | four and a half LIM domains 2 | 9.26E-05 | -4.08 |
| 2856 | Cfa.11031.1.A1_at | --- | --- | 6.16E-13 | -4.09 |
| 2857 | Cfa.15979.1.S1_at | --- | --- | 0.000848829 | -4.09 |
| 2858 | CfaAffx.21934.1.S1_s_at | DNAJA4 | DnaJ (Hsp40) homolog, subfamily A, member 4 | 1.50E-08 | -4.10 |
| 2859 | CfaAffx.722.1.S1_x_at | --- | --- | 1.16E-08 | -4.10 |
| 2860 | Cfa.7231.1.A1_at | --- | --- | 3.06E-10 | -4.10 |
| 2861 | Cfa.2268.1.S1_at | LRIG3 | leucine-rich repeats and immunoglobulin-like domains 3 | 1.25E-08 | -4.10 |
| 2862 | Cfa.3746.1.S1_s_at | HTR2A | 5-hydroxytryptamine (serotonin) receptor 2A | 8.18E-10 | -4.11 |
| 2863 | Cfa.12981.1.A1_at | --- | --- | 0.00108583 | -4.12 |
| 2864 | Cfa.1636.1.A1_s_at | SSBP2 | single-stranded DNA binding protein 2 | 2.39E-07 | -4.12 |
| 2865 | Cfa.4482.1.S1_at | --- | --- | 1.74E-12 | -4.13 |
| 2866 | Cfa.6346.1.A1_at | --- | --- | 8.09E-11 | -4.13 |
| 2867 | Cfa.9970.1.A1_s_at | RBP7 | retinol binding protein 7, cellular | 0.00017857 | -4.14 |
| 2868 | CfaAffx.11406.1.S1_s_at | FGL1 | fibrinogen-like 1 | 4.12E-06 | -4.14 |
| 2869 | Cfa.11549.1.A1_at | FBXW10 | F-box and WD repeat domain containing 10 | 1.58E-10 | -4.15 |
| 2870 | Cfa.10616.1.A1_at | --- | --- | 1.45E-10 | -4.15 |
| 2871 | CfaAffx.10552.1.S1_at | CCRL1 | chemokine (C-C motif) receptor-like 1 | 2.95E-06 | -4.16 |
| 2872 | CfaAffx.1909.1.S1_s_at | SYTL3 | synaptotagmin-like 3 | 8.88E-09 | -4.17 |
| 2873 | CfaAffx.8031.1.S1_at | BEND7 | BEN domain containing 7 | 1.32E-06 | -4.18 |
| 2874 | Cfa.15224.1.S1_at | ME1 | Malic enzyme 1, NADP(+)-dependent, cytosolic | 4.25E-05 | -4.18 |
| 2875 | CfaAffx.23471.1.S1_at | XCL2 | chemokine (C motif) ligand 2 | 6.20E-07 | -4.19 |
| 2876 | Cfa.3596.2.S1_at | CD80 | CD80 molecule | 8.54E-11 | -4.19 |
| 2877 | Cfa.12195.14.S1_s_at | LOC475754 | similar to Ig kappa chain C region, B allele | 0.000162287 | -4.20 |
| 2878 | Cfa.2400.1.A1_at | LOC478827 | similar to Secreted frizzled-related protein 3 precursor (sFRP-3) (Frizzled-rela | 0.000410379 | -4.20 |
| 2879 | Cfa.3000.1.A1_at | --- | --- | 6.51E-09 | -4.20 |
| 2880 | CfaAffx.13491.1.S1_at | GPR171 | G protein-coupled receptor 171 | 0.000151234 | -4.20 |
| 2881 | CfaAffx.20902.1.S1_x_at | LOC486389 /// LOC491454 /// LOC606941 /// LOC606953 /// LOC612050 /// LOC612054 /// LOC612081 /// LOC612128 | similar to Ig lambda chain V-I region BL2 precursor /// similar to Ig lambda cha | 4.79E-05 | -4.21 |
| 2882 | CfaAffx.16575.1.S1_s_at | RGS1 | regulator of G-protein signaling 1 | 7.59E-05 | -4.22 |
| 2883 | Cfa.13870.1.A1_at | --- | --- | 1.24E-12 | -4.23 |
| 2884 | CfaAffx.1729.1.S1_at | NCR3 | natural cytotoxicity triggering receptor 3 | 1.01E-08 | -4.23 |
| 2885 | CfaAffx.11459.1.S1_at | CHCHD7 | coiled-coil-helix-coiled-coil-helix domain containing 7 | 5.50E-07 | -4.23 |
| 2886 | Cfa.12431.1.A1_at | --- | --- | 3.04E-09 | -4.23 |
| 2887 | Cfa.2700.1.A1_at | --- | --- | 1.92E-05 | -4.23 |
| 2888 | CfaAffx.21823.1.S1_s_at | VPREB1 | pre-B lymphocyte 1 | 4.70E-06 | -4.24 |
| 2889 | CfaAffx.22327.1.S1_s_at | LOC480788 | similar to T-cell receptor beta chain C region | 3.30E-13 | -4.25 |
| 2890 | CfaAffx.23890.1.S1_x_at | LOC609871 | similar to Ig lambda chain V region 4A precursor | 6.23E-09 | -4.25 |
| 2891 | CfaAffx.20730.1.S1_s_at | LOC491436 /// LOC610478 /// LOC611153 /// LRRC37A2 | hypothetical LOC491436 /// similar to CG14692-PA /// similar to CG14692-PA /// l | 0.000172525 | -4.26 |
| 2892 | Cfa.2717.2.A1_at | NTRK3 | neurotrophic tyrosine kinase, receptor, type 3 | 7.26E-10 | -4.26 |
| 2893 | Cfa.7364.1.A1_at | LOC480007 | hypothetical LOC480007 | 1.12E-08 | -4.27 |
| 2894 | Cfa.5377.1.A1_at | PTK2 | PTK2 protein tyrosine kinase 2 | 2.90E-09 | -4.27 |
| 2895 | Cfa.6383.1.A1_at | --- | --- | 7.09E-07 | -4.28 |
| 2896 | CfaAffx.25541.1.S1_s_at | RORA | RAR-related orphan receptor A | 6.53E-07 | -4.28 |
| 2897 | Cfa.14527.1.A1_at | ANKRD55 | ankyrin repeat domain 55 | 5.20E-10 | -4.29 |
| 2898 | CfaAffx.6520.1.S1_s_at | AIM1 | absent in melanoma 1 | 0.000248378 | -4.29 |
| 2899 | Cfa.9120.1.A1_at | --- | --- | 0.000166362 | -4.30 |
| 2900 | Cfa.14276.1.A1_at | --- | --- | 0.00164651 | -4.30 |
| 2901 | Cfa.14465.1.S1_at | --- | --- | 0.000100133 | -4.31 |
| 2902 | Cfa.15618.1.A1_at | --- | --- | 3.88E-10 | -4.31 |
| 2903 | CfaAffx.6891.1.S1_at | SULT2B1 | sulfotransferase family, cytosolic, 2B, member 1 | 0.000177341 | -4.32 |
| 2904 | Cfa.16997.1.S1_at | DUSP23 | dual specificity phosphatase 23 | 2.66E-10 | -4.32 |
| 2905 | CfaAffx.17811.1.S1_at | CD1E | CD1e molecule | 0.000121777 | -4.32 |
| 2906 | Cfa.11584.1.A1_at | BFSP1 | beaded filament structural protein 1, filensin | 1.89E-12 | -4.33 |
| 2907 | Cfa.17550.1.S1_s_at | CIDEC | cell death-inducing DFFA-like effector c | 0.0122431 | -4.34 |
| 2908 | Cfa.269.1.A1_at | RYR2 | Ryanodine receptor 2 (cardiac) | 0.000326165 | -4.34 |
| 2909 | Cfa.6443.1.A1_at | --- | --- | 2.25E-06 | -4.34 |
| 2910 | Cfa.13439.1.S1_at | FGL1 | fibrinogen-like 1 | 5.58E-06 | -4.35 |
| 2911 | Cfa.14070.1.A1_at | --- | --- | 4.06E-05 | -4.35 |
| 2912 | Cfa.15116.1.S1_s_at | DPP4 | dipeptidyl-peptidase 4 | 7.05E-13 | -4.36 |
| 2913 | Cfa.13214.1.A1_at | --- | --- | 9.18E-05 | -4.36 |
| 2914 | Cfa.10899.1.A1_at | --- | --- | 5.15E-08 | -4.37 |
| 2915 | Cfa.1528.1.A1_at | --- | --- | 2.23E-08 | -4.37 |
| 2916 | CfaAffx.23018.1.S1_x_at | LOC490595 | similar to T-cell receptor alpha chain V region CTL-F3 precursor | 1.58E-13 | -4.39 |
| 2917 | Cfa.9321.1.A1_at | --- | --- | 4.55E-09 | -4.40 |
| 2918 | CfaAffx.7872.1.S1_at | RAB30 | RAB30, member RAS oncogene family | 5.42E-09 | -4.40 |
| 2919 | Cfa.16244.1.S1_s_at | RUNX2 | runt-related transcription factor 2 | 6.19E-07 | -4.40 |
| 2920 | Cfa.3510.1.S2_at | IL8 | interleukin 8 | 0.0034537 | -4.40 |
| 2921 | Cfa.16280.1.S1_at | --- | --- | 1.52E-12 | -4.42 |
| 2922 | CfaAffx.19983.1.S1_at | CAMP | cathelicidin antimicrobial peptide | 0.000465777 | -4.42 |
| 2923 | Cfa.2650.1.A1_s_at | --- | --- | 1.74E-08 | -4.43 |
| 2924 | CfaAffx.4018.1.S1_at | SIT1 | signaling threshold regulating transmembrane adaptor 1 | 1.36E-10 | -4.44 |
| 2925 | Cfa.21214.1.S1_at | IL7R | interleukin 7 receptor | 4.65E-05 | -4.44 |
| 2926 | Cfa.4865.2.A1_a_at | --- | --- | 9.40E-06 | -4.44 |
| 2927 | CfaAffx.345.1.S1_x_at | LOC491494 /// LOC607125 | similar to Ig lambda chain V region 4A precursor /// similar to Ig lambda chain | 3.15E-08 | -4.44 |
| 2928 | Cfa.3742.1.S1_s_at | ALCAM | activated leukocyte cell adhesion molecule | 5.96E-09 | -4.45 |
| 2929 | Cfa.1329.1.A1_at | PRPH | peripherin | 2.71E-07 | -4.45 |
| 2930 | Cfa.12195.1.S1_at | --- | --- | 0.00224757 | -4.45 |
| 2931 | CfaAffx.17325.1.S1_at | --- | --- | 8.33E-13 | -4.46 |
| 2932 | Cfa.15675.1.A1_at | --- | --- | 6.94E-11 | -4.46 |
| 2933 | CfaAffx.11036.1.S1_s_at | LOC475605 | similar to Low-density lipoprotein receptor-related protein 4 precursor (LDLR da | 5.07E-08 | -4.49 |
| 2934 | Cfa.1376.1.A1_at | --- | --- | 4.74E-10 | -4.49 |
| 2935 | Cfa.10773.1.A1_s_at | LOC477662 | hypothetical LOC477662 | 8.96E-05 | -4.49 |
| 2936 | CfaAffx.28434.1.S1_s_at | SH2D1A | SH2 domain containing 1A | 1.34E-07 | -4.50 |
| 2937 | CfaAffx.438.1.S1_s_at | KRT18 /// LOC486139 | keratin 18 /// similar to Keratin, type I cytoskeletal 18 (Cytokeratin 18) (K18) | 0.00285164 | -4.50 |
| 2938 | CfaAffx.6567.1.S1_at | KCNS3 | potassium voltage-gated channel, delayed-rectifier, subfamily S, member 3 | 2.29E-09 | -4.50 |
| 2939 | Cfa.15273.1.A1_at | LOC477555 | similar to glutathione S-transferase, theta 3 | 1.52E-07 | -4.50 |
| 2940 | Cfa.1280.1.A1_at | NDRG2 | NDRG family member 2 | 3.91E-07 | -4.50 |
| 2941 | Cfa.14377.1.S1_at | --- | --- | 0.000894875 | -4.50 |
| 2942 | CfaAffx.11018.1.S1_at | PXDNL | peroxidasin homolog (Drosophila)-like | 5.41E-08 | -4.51 |
| 2943 | Cfa.4920.1.A1_at | --- | --- | 9.29E-06 | -4.51 |
| 2944 | Cfa.2335.1.A1_at | F13A1 | coagulation factor XIII, A1 polypeptide | 0.00302377 | -4.51 |
| 2945 | Cfa.10966.1.A1_at | DNM1 | dynamin 1 | 4.76E-07 | -4.52 |
| 2946 | CfaAffx.1908.1.S1_at | SYTL3 | synaptotagmin-like 3 | 1.20E-09 | -4.52 |
| 2947 | Cfa.4221.1.S1_at | --- | --- | 4.61E-10 | -4.53 |
| 2948 | CfaAffx.6110.1.S1_at | IL4I1 | interleukin 4 induced 1 | 8.20E-11 | -4.53 |
| 2949 | Cfa.11352.1.A1_at | --- | --- | 2.13E-11 | -4.53 |
| 2950 | Cfa.1282.1.A1_s_at | PTGDS | prostaglandin D2 synthase 21kDa (brain) | 2.02E-07 | -4.53 |
| 2951 | Cfa.10907.1.S1_at | --- | --- | 0.000191144 | -4.54 |
| 2952 | Cfa.14395.1.A1_at | PTER | phosphotriesterase related | 8.59E-06 | -4.55 |
| 2953 | Cfa.2391.1.S1_at | AASS | aminoadipate-semialdehyde synthase | 2.20E-11 | -4.56 |
| 2954 | Cfa.6022.1.A1_at | S100A2 | S100 calcium binding protein A2 | 8.45E-07 | -4.58 |
| 2955 | Cfa.10818.1.A1_at | --- | --- | 5.93E-11 | -4.58 |
| 2956 | Cfa.9084.1.A1_at | CCDC3 | coiled-coil domain containing 3 | 3.05E-07 | -4.58 |
| 2957 | Cfa.13718.1.S1_s_at | SNCAIP | synuclein, alpha interacting protein | 1.70E-09 | -4.58 |
| 2958 | Cfa.10427.1.A1_at | HSD17B14 | hydroxysteroid (17-beta) dehydrogenase 14 | 1.04E-05 | -4.59 |
| 2959 | CfaAffx.9221.1.S1_at | EOMES | eomesodermin homolog (Xenopus laevis) | 1.37E-06 | -4.60 |
| 2960 | Cfa.12022.1.A1_at | DNAH8 | dynein, axonemal, heavy chain 8 | 1.64E-14 | -4.60 |
| 2961 | Cfa.1912.1.S1_at | GM2A | GM2 ganglioside activator | 4.29E-08 | -4.61 |
| 2962 | Cfa.13580.1.S1_at | RIMBP2 | RIMS binding protein 2 | 1.64E-08 | -4.61 |
| 2963 | Cfa.9534.1.A1_at | --- | --- | 3.50E-09 | -4.62 |
| 2964 | Cfa.14108.1.A1_at | --- | --- | 3.22E-10 | -4.63 |
| 2965 | Cfa.8764.2.A1_at | ACSBG2 | acyl-CoA synthetase bubblegum family member 2 | 2.50E-10 | -4.63 |
| 2966 | Cfa.6344.1.A1_at | --- | --- | 0.00133719 | -4.64 |
| 2967 | Cfa.19564.1.S1_at | --- | --- | 1.46E-07 | -4.64 |
| 2968 | Cfa.12541.1.A1_at | --- | --- | 4.65E-06 | -4.65 |
| 2969 | Cfa.244.1.S1_at | UBE2Q2 | ubiquitin-conjugating enzyme E2Q family member 2 | 9.68E-14 | -4.68 |
| 2970 | CfaAffx.10994.1.S1_s_at | FLT3 | fms-related tyrosine kinase 3 | 1.86E-05 | -4.69 |
| 2971 | CfaAffx.5058.1.S1_s_at | ZNF616 | zinc finger protein 616 | 1.10E-07 | -4.69 |
| 2972 | Cfa.6034.1.A1_at | --- | --- | 2.73E-07 | -4.69 |
| 2973 | CfaAffx.13984.1.S1_s_at | TIAM1 | T-cell lymphoma invasion and metastasis 1 | 1.49E-12 | -4.69 |
| 2974 | Cfa.7125.1.A1_at | CPXM2 | carboxypeptidase X (M14 family), member 2 | 3.15E-08 | -4.70 |
| 2975 | Cfa.8846.1.A1_s_at | CFB | complement factor B | 8.29E-07 | -4.71 |
| 2976 | Cfa.7134.1.A1_at | --- | --- | 2.48E-09 | -4.72 |
| 2977 | Cfa.14034.1.A1_at | LRP2 | low density lipoprotein receptor-related protein 2 | 1.43E-09 | -4.72 |
| 2978 | CfaAffx.24050.1.S1_s_at | PRKCH | protein kinase C, eta | 6.87E-11 | -4.72 |
| 2979 | Cfa.9535.1.A1_s_at | --- | --- | 2.66E-08 | -4.74 |
| 2980 | CfaAffx.11528.1.S1_s_at | SCUBE2 | signal peptide, CUB domain, EGF-like 2 | 7.41E-11 | -4.74 |
| 2981 | Cfa.14793.1.A1_at | --- | --- | 1.82E-10 | -4.76 |
| 2982 | Cfa.12186.1.A1_at | --- | --- | 0.00397105 | -4.77 |
| 2983 | Cfa.3332.1.S1_at | --- | --- | 2.86E-09 | -4.77 |
| 2984 | Cfa.13326.1.A1_at | --- | --- | 7.48E-12 | -4.78 |
| 2985 | Cfa.12929.1.A1_at | --- | --- | 1.13E-09 | -4.78 |
| 2986 | Cfa.5955.1.S1_at | CD5L | CD5 molecule-like | 4.64E-05 | -4.78 |
| 2987 | Cfa.10154.1.A1_at | --- | --- | 3.03E-08 | -4.80 |
| 2988 | CfaAffx.20685.1.S1_s_at | APOD | apolipoprotein D | 9.27E-07 | -4.81 |
| 2989 | Cfa.1669.1.A1_at | --- | --- | 7.53E-09 | -4.81 |
| 2990 | CfaAffx.13148.1.S1_s_at | CXADR | coxsackie virus and adenovirus receptor | 0.00101203 | -4.82 |
| 2991 | Cfa.14803.1.A1_at | --- | --- | 9.60E-08 | -4.82 |
| 2992 | CfaAffx.17502.1.S1_at | AXIN2 | axin 2 | 6.06E-10 | -4.83 |
| 2993 | CfaAffx.2491.1.S1_at | THEMIS | thymocyte selection associated | 1.53E-12 | -4.84 |
| 2994 | Cfa.9602.1.A1_at | --- | --- | 9.94E-08 | -4.85 |
| 2995 | Cfa.61.2.S1_s_at | PTGS1 | prostaglandin-endoperoxide synthase 1 (prostaglandin G/H synthase and cyclooxyge | 7.14E-09 | -4.85 |
| 2996 | CfaAffx.20522.1.S1_s_at | SH3GL3 | SH3-domain GRB2-like 3 | 2.89E-06 | -4.85 |
| 2997 | Cfa.5630.1.A1_at | --- | --- | 5.15E-07 | -4.86 |
| 2998 | Cfa.3130.1.A1_at | --- | --- | 4.46E-08 | -4.87 |
| 2999 | CfaAffx.18273.1.S1_at | FCER1A | Fc fragment of IgE, high affinity I, receptor for; alpha polypeptide | 1.50E-05 | -4.88 |
| 3000 | Cfa.1413.1.A1_s_at | LGI1 | leucine-rich, glioma inactivated 1 | 8.19E-07 | -4.88 |
| 3001 | Cfa.6387.1.A1_at | --- | --- | 2.52E-11 | -4.90 |
| 3002 | Cfa.1252.1.A1_at | LOC474447 | similar to calcium-activated potassium channel beta 4 subunit | 7.15E-07 | -4.90 |
| 3003 | Cfa.3626.1.A1_s_at | FGFR2 | fibroblast growth factor receptor 2 | 1.49E-09 | -4.90 |
| 3004 | Cfa.13589.1.A1_at | --- | --- | 3.50E-05 | -4.91 |
| 3005 | Cfa.12088.1.A1_at | --- | --- | 1.61E-10 | -4.92 |
| 3006 | Cfa.11921.1.A1_at | AMICA1 | adhesion molecule, interacts with CXADR antigen 1 | 6.55E-08 | -4.92 |
| 3007 | CfaAffx.19837.1.S1_s_at | AMICA1 | adhesion molecule, interacts with CXADR antigen 1 | 6.32E-07 | -4.92 |
| 3008 | Cfa.574.1.A1_at | EPHX2 | epoxide hydrolase 2, cytoplasmic | 1.67E-06 | -4.94 |
| 3009 | Cfa.6128.1.A1_at | SLPI | secretory leukocyte peptidase inhibitor | 0.00821907 | -4.95 |
| 3010 | CfaAffx.16101.1.S1_at | TSPAN5 | tetraspanin 5 | 2.49E-08 | -4.95 |
| 3011 | Cfa.11233.1.A1_at | SSTR2 | somatostatin receptor 2 | 2.34E-14 | -4.98 |
| 3012 | Cfa.14148.1.A1_at | --- | --- | 1.27E-07 | -4.98 |
| 3013 | CfaAffx.11273.1.S1_at | EHF | ets homologous factor | 1.19E-09 | -5.01 |
| 3014 | Cfa.11885.1.A1_at | --- | --- | 5.89E-12 | -5.01 |
| 3015 | CfaAffx.7333.1.S1_at | --- | --- | 1.24E-06 | -5.02 |
| 3016 | Cfa.6289.1.A1_at | --- | --- | 2.10E-05 | -5.02 |
| 3017 | Cfa.16335.1.S1_at | GFI1 | growth factor independent 1 transcription repressor | 7.66E-07 | -5.03 |
| 3018 | Cfa.4552.1.S1_s_at | CAMP | cathelicidin antimicrobial peptide | 0.000275336 | -5.03 |
| 3019 | Cfa.12290.1.A1_at | --- | --- | 0.000438728 | -5.04 |
| 3020 | Cfa.10644.1.A1_at | SH3GL3 | SH3-domain GRB2-like 3 | 5.60E-06 | -5.04 |
| 3021 | Cfa.10428.1.A1_at | --- | --- | 5.04E-07 | -5.06 |
| 3022 | CfaAffx.22982.1.S1_s_at | LOC490595 /// LOC491376 /// LOC491543 /// LOC607750 | similar to T-cell receptor alpha chain V region CTL-F3 precursor /// similar to | 5.92E-14 | -5.09 |
| 3023 | Cfa.12998.1.A1_at | --- | --- | 0.000307583 | -5.09 |
| 3024 | Cfa.19850.1.S1_at | --- | --- | 5.34E-09 | -5.09 |
| 3025 | CfaAffx.25096.1.S1_s_at | CD6 | CD6 molecule | 1.44E-15 | -5.10 |
| 3026 | CfaAffx.1646.1.S1_s_at | TSPAN8 | tetraspanin 8 | 1.42E-08 | -5.11 |
| 3027 | CfaAffx.19837.1.S1_at | AMICA1 | adhesion molecule, interacts with CXADR antigen 1 | 3.00E-07 | -5.11 |
| 3028 | Cfa.1451.1.A1_at | MYOM1 | myomesin 1, 185kDa | 3.80E-07 | -5.11 |
| 3029 | Cfa.7842.1.A1_s_at | ENPP3 | ectonucleotide pyrophosphatase/phosphodiesterase 3 | 6.59E-06 | -5.11 |
| 3030 | Cfa.12539.1.A1_at | --- | --- | 7.31E-09 | -5.12 |
| 3031 | Cfa.10957.1.A1_at | --- | --- | 8.89E-12 | -5.12 |
| 3032 | Cfa.3409.1.A1_at | --- | --- | 8.99E-10 | -5.13 |
| 3033 | Cfa.9369.1.S1_at | --- | --- | 6.52E-06 | -5.13 |
| 3034 | Cfa.14738.1.A1_at | --- | --- | 2.95E-10 | -5.14 |
| 3035 | CfaAffx.13209.1.S1_s_at | CP | ceruloplasmin (ferroxidase) | 1.20E-07 | -5.16 |
| 3036 | Cfa.2878.1.A1_s_at | CP | ceruloplasmin (ferroxidase) | 1.89E-07 | -5.16 |
| 3037 | CfaAffx.2384.1.S1_s_at | TCF7 | transcription factor 7 (T-cell specific, HMG-box) | 9.64E-12 | -5.16 |
| 3038 | CfaAffx.28416.1.S1_at | --- | --- | 3.36E-10 | -5.18 |
| 3039 | Cfa.12195.9.A1_at | --- | --- | 1.53E-10 | -5.18 |
| 3040 | CfaAffx.24293.1.S1_at | LOC480351 | similar to testis expressed gene 21 | 6.77E-12 | -5.19 |
| 3041 | CfaAffx.7643.1.S1_at | HTR2A | 5-hydroxytryptamine (serotonin) receptor 2A | 2.71E-09 | -5.20 |
| 3042 | Cfa.3942.1.A1_at | --- | --- | 3.87E-10 | -5.20 |
| 3043 | Cfa.3890.1.S1_at | CD3E | CD3e molecule, epsilon (CD3-TCR complex) | 1.19E-07 | -5.20 |
| 3044 | CfaAffx.741.1.S1_at | FYB | FYN binding protein | 1.04E-08 | -5.23 |
| 3045 | Cfa.13671.1.A1_at | --- | --- | 2.15E-12 | -5.25 |
| 3046 | Cfa.15230.1.A1_at | --- | --- | 8.26E-08 | -5.25 |
| 3047 | CfaAffx.26444.1.S1_s_at | IL12B | interleukin 12B (natural killer cell stimulatory factor 2, cytotoxic lymphocyte | 4.92E-16 | -5.30 |
| 3048 | Cfa.16636.1.S1_at | --- | --- | 4.18E-08 | -5.30 |
| 3049 | Cfa.12409.1.A1_at | --- | --- | 1.90E-07 | -5.31 |
| 3050 | CfaAffx.360.1.S1_s_at | ADAMDEC1 | ADAM-like, decysin 1 | 0.0018108 | -5.32 |
| 3051 | Cfa.2717.1.A1_a_at | NTRK3 | neurotrophic tyrosine kinase, receptor, type 3 | 6.50E-10 | -5.32 |
| 3052 | Cfa.11152.1.A1_at | --- | --- | 1.80E-05 | -5.32 |
| 3053 | CfaAffx.6649.1.S1_s_at | --- | --- | 2.28E-08 | -5.32 |
| 3054 | CfaAffx.13307.1.S1_s_at | STMN2 | stathmin-like 2 | 3.30E-08 | -5.32 |
| 3055 | Cfa.9686.1.A1_at | --- | --- | 0.00012526 | -5.34 |
| 3056 | Cfa.19829.1.S1_at | --- | --- | 7.96E-07 | -5.34 |
| 3057 | Cfa.15116.1.S1_at | DPP4 | dipeptidyl-peptidase 4 | 9.16E-13 | -5.35 |
| 3058 | Cfa.2684.1.A1_at | --- | --- | 4.16E-12 | -5.35 |
| 3059 | Cfa.15713.1.A1_s_at | MARCO | macrophage receptor with collagenous structure | 2.61E-05 | -5.36 |
| 3060 | CfaAffx.26524.1.S1_s_at | LAT | linker for activation of T cells | 4.24E-10 | -5.42 |
| 3061 | Cfa.9301.1.A1_at | --- | --- | 1.62E-10 | -5.42 |
| 3062 | Cfa.3988.1.S1_at | --- | --- | 2.16E-12 | -5.45 |
| 3063 | CfaAffx.19953.1.S1_s_at | ICOS | inducible T-cell co-stimulator | 1.63E-10 | -5.45 |
| 3064 | Cfa.6338.1.A1_s_at | --- | --- | 1.51E-10 | -5.45 |
| 3065 | Cfa.6795.1.S1_at | LOC608537 | hypothetical protein LOC608537 | 9.24E-11 | -5.47 |
| 3066 | Cfa.4784.1.A1_at | --- | --- | 5.47E-10 | -5.48 |
| 3067 | Cfa.8905.1.A1_at | LOC492040 | similar to zinc finger, CCHC domain containing 12 | 5.52E-13 | -5.48 |
| 3068 | CfaAffx.28285.1.S1_at | LOC490895 | similar to Ig heavy chain V-III region VH26 precursor | 4.25E-05 | -5.49 |
| 3069 | Cfa.2445.1.S1_at | --- | --- | 5.57E-08 | -5.49 |
| 3070 | CfaAffx.17301.1.S1_at | LOC607937 | similar to T-cell receptor alpha chain C region | 2.20E-09 | -5.49 |
| 3071 | Cfa.21327.1.S1_at | LOC481674 | similar to alpha-2u globulin PGCL4 | 1.37E-12 | -5.50 |
| 3072 | CfaAffx.6651.1.S1_at | --- | --- | 4.67E-14 | -5.51 |
| 3073 | Cfa.3050.1.A1_at | --- | --- | 2.32E-08 | -5.51 |
| 3074 | CfaAffx.11632.1.S1_s_at | KRT18 | keratin 18 | 0.000941318 | -5.51 |
| 3075 | Cfa.4754.1.A1_s_at | ABCA6 | ATP-binding cassette, sub-family A (ABC1), member 6 | 1.83E-05 | -5.54 |
| 3076 | Cfa.15762.1.S1_at | --- | --- | 0.0015601 | -5.54 |
| 3077 | Cfa.11170.1.A1_at | DOCK8 | dedicator of cytokinesis 8 | 1.57E-06 | -5.54 |
| 3078 | Cfa.16733.1.S1_at | --- | --- | 5.30E-10 | -5.54 |
| 3079 | CfaAffx.29026.1.S1_at | LOC610563 | similar to CG12132-PA | 1.25E-09 | -5.55 |
| 3080 | Cfa.12256.1.A1_at | --- | --- | 0.000157968 | -5.56 |
| 3081 | Cfa.15663.1.A1_at | --- | --- | 2.16E-05 | -5.59 |
| 3082 | Cfa.1240.1.A1_at | --- | --- | 3.67E-05 | -5.59 |
| 3083 | Cfa.5419.1.A1_at | FAIM2 | Fas apoptotic inhibitory molecule 2 | 5.66E-14 | -5.60 |
| 3084 | Cfa.3663.1.A1_s_at | FCER1A | Fc fragment of IgE, high affinity I, receptor for; alpha polypeptide | 7.23E-06 | -5.62 |
| 3085 | Cfa.15713.1.A1_at | MARCO | macrophage receptor with collagenous structure | 1.08E-05 | -5.63 |
| 3086 | Cfa.8977.1.A1_at | --- | --- | 1.19E-08 | -5.66 |
| 3087 | Cfa.16577.1.S1_at | ASPA | aspartoacylase (Canavan disease) | 2.17E-07 | -5.66 |
| 3088 | Cfa.5139.1.A1_s_at | FST | follistatin | 1.86E-12 | -5.68 |
| 3089 | Cfa.4288.1.A1_at | --- | --- | 2.25E-13 | -5.69 |
| 3090 | Cfa.11943.1.A1_at | RDH16 | retinol dehydrogenase 16 (all-trans) | 9.00E-09 | -5.73 |
| 3091 | Cfa.5452.1.A1_at | --- | --- | 1.03E-06 | -5.74 |
| 3092 | Cfa.13359.1.A1_at | --- | --- | 5.31E-07 | -5.74 |
| 3093 | CfaAffx.10997.1.S1_at | FLT3 | fms-related tyrosine kinase 3 | 4.24E-06 | -5.75 |
| 3094 | CfaAffx.11903.1.S1_at | LOC611906 | similar to Ig kappa chain V-III region VG precursor | 4.21E-06 | -5.76 |
| 3095 | Cfa.3794.1.A1_s_at | MAL | mal, T-cell differentiation protein | 1.77E-06 | -5.76 |
| 3096 | Cfa.16398.1.S1_at | SULF2 | sulfatase 2 | 2.33E-05 | -5.77 |
| 3097 | CfaAffx.28217.1.S1_at | --- | --- | 0.000839239 | -5.82 |
| 3098 | Cfa.1417.1.S1_at | APOD | apolipoprotein D | 7.24E-08 | -5.84 |
| 3099 | Cfa.17456.1.S1_at | LOC484897 | similar to egf-like module containing, mucin-like, hormone receptor-like sequenc | 6.87E-05 | -5.84 |
| 3100 | CfaAffx.18180.1.S1_at | CRTAM | cytotoxic and regulatory T cell molecule | 7.74E-11 | -5.87 |
| 3101 | CfaAffx.13148.1.S1_at | CXADR | coxsackie virus and adenovirus receptor | 0.000485838 | -5.89 |
| 3102 | Cfa.11048.1.A1_at | TLE1 | transducin-like enhancer of split 1 (E(sp1) homolog, Drosophila) | 1.15E-07 | -5.90 |
| 3103 | CfaAffx.16562.1.S1_at | RGS13 | regulator of G-protein signaling 13 | 3.50E-05 | -5.90 |
| 3104 | CfaAffx.28271.1.S1_x_at | LOC607179 | similar to Ig heavy chain V-III region VH26 precursor | 0.000609412 | -5.92 |
| 3105 | CfaAffx.5350.1.S1_at | HOXA5 | homeobox A5 | 5.29E-10 | -5.95 |
| 3106 | Cfa.12394.1.A1_at | --- | --- | 1.06E-10 | -5.96 |
| 3107 | CfaAffx.3167.1.S1_s_at | LOC482261 | similar to family with sequence similarity 40, member B | 1.25E-11 | -5.97 |
| 3108 | Cfa.11186.1.A1_at | --- | --- | 4.72E-05 | -5.98 |
| 3109 | Cfa.11355.1.A1_at | --- | --- | 2.37E-08 | -5.98 |
| 3110 | CfaAffx.19812.1.S1_s_at | ANK3 | ankyrin 3, node of Ranvier (ankyrin G) | 2.14E-08 | -5.99 |
| 3111 | CfaAffx.21985.1.S1_s_at | IKZF2 | IKAROS family zinc finger 2 (Helios) | 8.84E-08 | -6.00 |
| 3112 | Cfa.6072.1.A1_at | --- | --- | 2.15E-06 | -6.00 |
| 3113 | Cfa.6455.1.A1_at | --- | --- | 5.25E-08 | -6.01 |
| 3114 | CfaAffx.30778.1.S1_s_at | SLC44A3 | solute carrier family 44, member 3 | 4.21E-14 | -6.01 |
| 3115 | Cfa.4827.1.A1_at | --- | --- | 4.15E-07 | -6.10 |
| 3116 | Cfa.12664.2.A1_s_at | ANK3 | ankyrin 3, node of Ranvier (ankyrin G) | 2.45E-07 | -6.13 |
| 3117 | CfaAffx.11938.1.S1_at | --- | --- | 1.91E-15 | -6.13 |
| 3118 | CfaAffx.23639.1.S1_at | LOC486384 | similar to Ig lambda chain V-I region BL2 precursor | 1.93E-07 | -6.14 |
| 3119 | CfaAffx.3380.1.S1_s_at | MLLT3 | myeloid/lymphoid or mixed-lineage leukemia (trithorax homolog, Drosophila); tran | 5.02E-11 | -6.16 |
| 3120 | Cfa.7473.1.A1_at | DDR1 | discoidin domain receptor tyrosine kinase 1 | 9.45E-10 | -6.17 |
| 3121 | Cfa.1647.1.A1_at | --- | --- | 2.46E-10 | -6.19 |
| 3122 | Cfa.3806.1.S1_at | CALCA | calcitonin/calcitonin-related polypeptide, alpha | 5.20E-07 | -6.22 |
| 3123 | CfaAffx.4263.1.S1_s_at | ICA1 | islet cell autoantigen 1, 69kDa | 7.47E-11 | -6.23 |
| 3124 | CfaAffx.1028.1.S1_s_at | CD226 | CD226 molecule | 5.87E-14 | -6.24 |
| 3125 | Cfa.11204.1.A1_at | LOC609534 | similar to response gene to complement 32 | 1.71E-09 | -6.24 |
| 3126 | Cfa.5402.1.A1_at | --- | --- | 1.17E-09 | -6.24 |
| 3127 | Cfa.9688.1.A1_at | NXPH2 | neurexophilin 2 | 3.07E-10 | -6.27 |
| 3128 | Cfa.2662.1.A1_at | LRRN1 | leucine rich repeat neuronal 1 | 3.47E-11 | -6.30 |
| 3129 | Cfa.15746.1.A1_at | --- | --- | 1.85E-08 | -6.31 |
| 3130 | CfaAffx.16646.1.S1_at | LCK | lymphocyte-specific protein tyrosine kinase | 4.63E-11 | -6.32 |
| 3131 | Cfa.15584.1.A1_at | LOC479637 | hypothetical LOC479637 | 9.36E-10 | -6.32 |
| 3132 | CfaAffx.21009.1.S1_at | LOC611455 | similar to Alpha-2-macroglobulin precursor (Alpha-2-M) | 1.22E-10 | -6.33 |
| 3133 | Cfa.16456.1.S1_at | --- | --- | 0.00310915 | -6.35 |
| 3134 | Cfa.4886.1.A1_at | OCA2 | oculocutaneous albinism II | 0.000166314 | -6.35 |
| 3135 | Cfa.3487.1.S1_at | ADIPOQ | adiponectin, C1Q and collagen domain containing | 0.000893349 | -6.37 |
| 3136 | Cfa.17196.1.S1_at | SH2D1A | SH2 domain containing 1A | 3.55E-10 | -6.42 |
| 3137 | CfaAffx.8754.1.S1_s_at | NXPH2 | neurexophilin 2 | 7.63E-11 | -6.42 |
| 3138 | Cfa.5063.1.S1_at | NMB | neuromedin B | 2.17E-12 | -6.43 |
| 3139 | CfaAffx.22348.1.S1_at | EFHC2 | EF-hand domain (C-terminal) containing 2 | 4.63E-09 | -6.46 |
| 3140 | CfaAffx.30778.1.S1_at | SLC44A3 | solute carrier family 44, member 3 | 2.95E-13 | -6.49 |
| 3141 | CfaAffx.12060.1.S1_s_at | CD8B | CD8b molecule | 3.90E-10 | -6.53 |
| 3142 | Cfa.15076.2.A1_a_at | VTN | vitronectin | 3.88E-09 | -6.54 |
| 3143 | CfaAffx.17544.1.S1_at | LEF1 | lymphoid enhancer-binding factor 1 | 1.42E-09 | -6.58 |
| 3144 | Cfa.11007.1.A1_at | --- | --- | 4.65E-10 | -6.62 |
| 3145 | Cfa.3478.1.S1_at | --- | --- | 0.000606479 | -6.64 |
| 3146 | CfaAffx.22878.1.S1_at | LOC486394 /// LOC607364 /// LOC607394 /// LOC607415 /// LOC607424 /// LOC607441 /// LOC607465 /// LOC607497 | similar to Ig lambda chain V-III region LOI /// similar to Immunoglobulin lambda | 0.00115727 | -6.64 |
| 3147 | Cfa.18289.1.S1_at | CD7 | CD7 molecule | 2.60E-16 | -6.70 |
| 3148 | Cfa.8363.1.A1_at | --- | --- | 1.67E-12 | -6.74 |
| 3149 | CfaAffx.4122.1.S1_at | IL1R2 | interleukin 1 receptor, type II | 3.38E-05 | -6.75 |
| 3150 | Cfa.5947.1.A1_at | --- | --- | 3.15E-12 | -6.78 |
| 3151 | Cfa.8884.1.A1_s_at | LOC483848 | similar to CG30418-PA | 8.31E-11 | -6.82 |
| 3152 | CfaAffx.28437.1.S1_s_at | C7 | complement component 7 | 6.10E-09 | -6.83 |
| 3153 | CfaAffx.28790.1.S1_at | --- | --- | 1.18E-05 | -6.85 |
| 3154 | Cfa.3684.1.S1_s_at | LOC609043 | similar to T-cell receptor beta chain V region CTL-L17 precursor | 1.42E-15 | -6.86 |
| 3155 | CfaAffx.8446.1.S1_at | GATA3 | GATA binding protein 3 | 1.24E-09 | -6.87 |
| 3156 | CfaAffx.8561.1.S1_at | CCR8 | chemokine (C-C motif) receptor 8 | 1.17E-15 | -6.87 |
| 3157 | CfaAffx.28246.1.S1_s_at | LOC606810 | similar to Ig heavy chain V-III region VH26 precursor | 1.84E-06 | -6.91 |
| 3158 | Cfa.17592.1.S1_at | LOC611813 | similar to double C2, gamma (predicted) | 4.53E-10 | -6.92 |
| 3159 | CfaAffx.22344.1.S1_s_at | LOC480788 | similar to T-cell receptor beta chain C region | 1.00E-12 | -6.96 |
| 3160 | CfaAffx.23303.1.S1_at | CD27 | CD27 molecule | 4.74E-13 | -6.99 |
| 3161 | CfaAffx.6655.1.S1_at | LOC482753 /// LOC609043 | similar to T-cell receptor beta chain V region 86T1 precursor /// similar to T-c | 1.09E-14 | -6.99 |
| 3162 | Cfa.4754.1.A1_at | ABCA6 | ATP-binding cassette, sub-family A (ABC1), member 6 | 5.58E-06 | -7.05 |
| 3163 | CfaAffx.4969.1.S1_s_at | DNAH11 | dynein, axonemal, heavy chain 11 | 2.72E-06 | -7.12 |
| 3164 | CfaAffx.812.1.S1_at | LOC486382 | similar to Ig lambda chain V-I region BL2 precursor | 2.73E-07 | -7.13 |
| 3165 | Cfa.16545.1.S1_at | --- | --- | 8.07E-06 | -7.19 |
| 3166 | Cfa.5221.1.A1_s_at | IL1R2 | interleukin 1 receptor, type II | 1.62E-05 | -7.19 |
| 3167 | CfaAffx.20393.1.S1_at | CTSW | cathepsin W | 2.09E-11 | -7.22 |
| 3168 | CfaAffx.28247.1.S1_s_at | --- | --- | 0.000320683 | -7.29 |
| 3169 | CfaAffx.705.1.S1_at | LOC608983 | similar to T-cell receptor beta chain V region YT35 precursor | 4.73E-12 | -7.29 |
| 3170 | Cfa.14472.1.A1_at | CCL14 | chemokine (C-C motif) ligand 14 | 8.78E-05 | -7.36 |
| 3171 | Cfa.5392.1.A1_s_at | --- | --- | 2.97E-07 | -7.37 |
| 3172 | Cfa.18970.1.S1_at | CDCA7L | cell division cycle associated 7-like | 1.05E-06 | -7.42 |
| 3173 | CfaAffx.8282.1.S1_at | VILL | villin-like | 6.53E-15 | -7.43 |
| 3174 | CfaAffx.6644.1.S1_s_at | LOC480788 | similar to T-cell receptor beta chain C region | 1.52E-08 | -7.48 |
| 3175 | Cfa.8414.1.A1_s_at | CIDEA | cell death-inducing DFFA-like effector a | 0.00166647 | -7.50 |
| 3176 | Cfa.9584.1.A1_s_at | EFHC2 | EF-hand domain (C-terminal) containing 2 | 3.69E-11 | -7.51 |
| 3177 | Cfa.13608.1.A1_at | --- | --- | 2.82E-15 | -7.51 |
| 3178 | Cfa.13410.1.A1_at | --- | --- | 4.19E-08 | -7.51 |
| 3179 | Cfa.12931.1.A1_at | DUSP27 | dual specificity phosphatase 27 (putative) | 7.04E-14 | -7.53 |
| 3180 | CfaAffx.6377.1.S1_s_at | INPP4B | inositol polyphosphate-4-phosphatase, type II, 105kDa | 8.22E-13 | -7.61 |
| 3181 | CfaAffx.1760.1.S1_at | OPRM1 | opioid receptor, mu 1 | 5.32E-10 | -7.62 |
| 3182 | CfaAffx.23322.1.S1_at | CFP | complement factor properdin | 1.38E-07 | -7.64 |
| 3183 | Cfa.19.1.S1_s_at | ABCB1 | ATP-binding cassette, sub-family B (MDR/TAP), member 1 | 1.10E-12 | -7.67 |
| 3184 | Cfa.2467.1.S1_at | GPC3 | glypican 3 | 7.31E-05 | -7.67 |
| 3185 | Cfa.13659.3.A1_at | FLOT1 | Flotillin 1 | 3.33E-06 | -7.68 |
| 3186 | Cfa.19648.1.S1_at | IDO1 | indoleamine 2,3-dioxygenase 1 | 3.73E-06 | -7.70 |
| 3187 | CfaAffx.28346.1.S1_at | IL23R | interleukin 23 receptor | 2.02E-13 | -7.70 |
| 3188 | Cfa.5725.1.A1_at | --- | --- | 3.18E-08 | -7.77 |
| 3189 | Cfa.179.1.S1_s_at | CD40LG | CD40 ligand | 4.89E-12 | -7.82 |
| 3190 | CfaAffx.849.1.S1_s_at | --- | --- | 2.00E-10 | -7.83 |
| 3191 | Cfa.5981.1.A1_at | --- | --- | 4.40E-08 | -7.87 |
| 3192 | CfaAffx.9573.1.S1_s_at | IDO1 | indoleamine 2,3-dioxygenase 1 | 1.98E-06 | -7.90 |
| 3193 | Cfa.9209.1.A1_at | LOC475605 | similar to Low-density lipoprotein receptor-related protein 4 precursor (LDLR da | 2.02E-10 | -7.90 |
| 3194 | Cfa.1339.1.A1_at | --- | --- | 1.71E-06 | -7.95 |
| 3195 | Cfa.21011.1.S1_at | CD8B | CD8b molecule | 3.97E-10 | -7.96 |
| 3196 | Cfa.4115.1.A1_at | DSCAML1 | Down syndrome cell adhesion molecule like 1 | 2.60E-06 | -7.97 |
| 3197 | CfaAffx.28248.1.S1_at | --- | --- | 0.000704587 | -8.11 |
| 3198 | CfaAffx.27914.1.S1_s_at | IL13RA2 | interleukin 13 receptor, alpha 2 | 7.33E-05 | -8.15 |
| 3199 | Cfa.59.1.S1_s_at | CYP3A26 | cytochrome P450 3A26 | 0.00418969 | -8.16 |
| 3200 | CfaAffx.11892.1.S1_at | LOC475754 /// LOC611906 | similar to Ig kappa chain C region, B allele /// similar to Ig kappa chain V-III | 9.61E-07 | -8.22 |
| 3201 | Cfa.4960.1.A1_at | --- | --- | 1.12E-08 | -8.27 |
| 3202 | CfaAffx.22642.1.S1_at | PTGDR | prostaglandin D2 receptor (DP) | 5.55E-13 | -8.28 |
| 3203 | CfaAffx.11585.1.S1_at | TOX | thymocyte selection-associated high mobility group box | 3.15E-07 | -8.35 |
| 3204 | CfaAffx.9573.1.S1_at | IDO1 | indoleamine 2,3-dioxygenase 1 | 2.34E-06 | -8.38 |
| 3205 | CfaAffx.10660.1.S1_s_at | TMEM178 | transmembrane protein 178 | 2.22E-09 | -8.42 |
| 3206 | Cfa.10787.1.A1_at | PRKCQ | protein kinase C, theta | 6.45E-12 | -8.43 |
| 3207 | CfaAffx.23352.1.S1_at | TNIK | TRAF2 and NCK interacting kinase | 8.18E-08 | -8.48 |
| 3208 | Cfa.11076.1.A1_at | --- | --- | 1.38E-14 | -8.56 |
| 3209 | Cfa.13393.1.A1_at | --- | --- | 8.19E-06 | -8.57 |
| 3210 | Cfa.7.1.S1_s_at | IL2 | interleukin 2 | 7.44E-10 | -8.66 |
| 3211 | CfaAffx.16090.1.S1_s_at | LOC478556 | similar to T-cell receptor interacting molecule | 1.37E-08 | -8.74 |
| 3212 | Cfa.10270.1.S1_at | STMN2 | stathmin-like 2 | 2.12E-08 | -8.74 |
| 3213 | Cfa.11141.1.A1_at | --- | --- | 4.77E-10 | -8.78 |
| 3214 | Cfa.13956.1.A1_at | --- | --- | 7.07E-08 | -8.84 |
| 3215 | Cfa.10948.1.S1_at | LOC610447 | similar to Visinin-like protein 1 (VILIP) (Neural visinin-like protein 1) (NVL-1 | 4.22E-13 | -8.88 |
| 3216 | CfaAffx.27812.1.S1_s_at | CCL14 | chemokine (C-C motif) ligand 14 | 6.13E-05 | -9.00 |
| 3217 | Cfa.4484.1.S1_at | --- | --- | 1.45E-06 | -9.12 |
| 3218 | CfaAffx.23890.1.S1_at | LOC609871 | similar to Ig lambda chain V region 4A precursor | 1.05E-12 | -9.20 |
| 3219 | CfaAffx.22981.1.S1_x_at | --- | --- | 2.50E-10 | -9.20 |
| 3220 | Cfa.12254.1.A1_at | CPO | carboxypeptidase O | 4.07E-11 | -9.21 |
| 3221 | CfaAffx.23305.1.S1_s_at | CD27 | CD27 molecule | 1.30E-08 | -9.24 |
| 3222 | CfaAffx.23355.1.S1_s_at | TNIK | TRAF2 and NCK interacting kinase | 1.03E-10 | -9.25 |
| 3223 | Cfa.9789.1.A1_at | --- | --- | 4.62E-06 | -9.29 |
| 3224 | CfaAffx.23301.1.S1_x_at | LOC491454 /// LOC606941 /// LOC606953 /// LOC607152 /// LOC612050 /// LOC612054 /// LOC612081 /// LOC612104 /// LOC612128 /// LOC612135 /// LOC612167 | similar to Ig lambda chain V-I region BL2 precursor /// similar to Ig lambda cha | 2.41E-08 | -9.39 |
| 3225 | Cfa.5320.1.A1_at | LOC480885 | Similar to Probable ubiquitin carboxyl-terminal hydrolase FAF-X (Ubiquitin thiol | 4.42E-14 | -9.47 |
| 3226 | CfaAffx.4419.1.S1_at | ZAP70 | zeta-chain (TCR) associated protein kinase 70kDa | 1.93E-11 | -9.48 |
| 3227 | Cfa.6173.1.A1_at | KLK11 | kallikrein-related peptidase 11 | 1.97E-10 | -9.48 |
| 3228 | CfaAffx.23301.1.S1_at | LOC606941 /// LOC606953 /// LOC607152 /// LOC612050 /// LOC612054 /// LOC612081 /// LOC612128 /// LOC612135 /// LOC612167 | similar to Ig lambda chain V-I region BL2 precursor /// similar to Ig lambda cha | 1.69E-07 | -9.70 |
| 3229 | CfaAffx.5065.1.S1_s_at | ZNF678 | zinc finger protein 678 | 1.18E-11 | -9.76 |
| 3230 | CfaAffx.706.1.S1_at | --- | --- | 5.35E-11 | -9.79 |
| 3231 | CfaAffx.21059.1.S1_at | LOC607368 | similar to Ig lambda chain V-IV region Bau | 0.000219945 | -9.90 |
| 3232 | CfaAffx.17535.1.S1_s_at | LEF1 | lymphoid enhancer-binding factor 1 | 2.85E-10 | -9.93 |
| 3233 | CfaAffx.4419.1.S1_s_at | --- | --- | 2.77E-12 | -9.94 |
| 3234 | Cfa.5555.1.A1_at | --- | --- | 1.05E-09 | -9.96 |
| 3235 | Cfa.9931.1.A1_at | --- | --- | 5.02E-10 | -9.96 |
| 3236 | Cfa.3479.1.A1_at | --- | --- | 7.07E-07 | -10.04 |
| 3237 | CfaAffx.8704.1.S1_s_at | PRKCQ | protein kinase C, theta | 3.31E-13 | -10.06 |
| 3238 | CfaAffx.25037.1.S1_at | CD5 | CD5 molecule | 1.57E-13 | -10.23 |
| 3239 | Cfa.12371.1.A1_s_at | FMO2 | flavin containing monooxygenase 2 (non-functional) | 2.81E-06 | -10.29 |
| 3240 | CfaAffx.22639.1.S1_s_at | LOC490595 /// LOC491543 | similar to T-cell receptor alpha chain V region CTL-F3 precursor /// similar to | 1.24E-12 | -10.33 |
| 3241 | Cfa.3685.4.S1_s_at | LOC608959 | similar to T-cell receptor beta chain V region CTL-F3 precursor | 1.53E-12 | -10.36 |
| 3242 | Cfa.3865.1.S1_at | LOC607937 | similar to T-cell receptor alpha chain C region | 1.99E-09 | -10.40 |
| 3243 | Cfa.3479.1.A1_x_at | --- | --- | 2.02E-06 | -10.42 |
| 3244 | CfaAffx.19814.1.S1_s_at | CD3E | CD3e molecule, epsilon (CD3-TCR complex) | 2.60E-09 | -10.47 |
| 3245 | Cfa.2343.1.S1_at | TMEM178 | transmembrane protein 178 | 4.19E-09 | -10.48 |
| 3246 | Cfa.3508.1.S1_s_at | CD28 | CD28 molecule | 9.54E-10 | -10.53 |
| 3247 | Cfa.3284.1.S1_s_at | --- | --- | 0.00024097 | -10.68 |
| 3248 | Cfa.1596.1.S1_at | --- | --- | 6.04E-10 | -10.85 |
| 3249 | CfaAffx.23398.1.S1_x_at | --- | --- | 3.23E-09 | -10.88 |
| 3250 | Cfa.2029.1.S1_at | LOC478556 | similar to T-cell receptor interacting molecule | 9.91E-08 | -10.88 |
| 3251 | CfaAffx.17301.1.S1_s_at | LOC607937 | similar to T-cell receptor alpha chain C region | 1.13E-09 | -10.94 |
| 3252 | Cfa.6201.1.A1_s_at | VPREB1 | pre-B lymphocyte 1 | 1.28E-06 | -11.04 |
| 3253 | CfaAffx.18462.1.S1_at | CSTA | cystatin A (stefin A) | 2.08E-05 | -11.18 |
| 3254 | CfaAffx.23527.1.S1_x_at | LOC491454 | similar to Ig lambda chain V-I region BL2 precursor | 5.03E-05 | -11.37 |
| 3255 | CfaAffx.20971.1.S1_at | LOC486392 | similar to Ig lambda chain V region 4A precursor | 3.36E-06 | -11.61 |
| 3256 | Cfa.179.1.S2_s_at | CD40LG | CD40 ligand | 1.21E-11 | -11.66 |
| 3257 | CfaAffx.22981.1.S1_s_at | --- | --- | 1.59E-10 | -11.86 |
| 3258 | CfaAffx.22083.1.S1_at | LOC612135 | similar to Ig lambda chain V region 4A precursor | 1.54E-06 | -12.32 |
| 3259 | CfaAffx.23040.1.S1_x_at | LOC491391 /// LOC491492 /// LOC491686 /// LOC607996 /// LOC608202 /// LOC608379 /// LOC609845 /// LOC610282 | similar to Ig kappa chain V-II region 26-10 /// similar to Ig kappa chain V-II r | 9.35E-06 | -12.46 |
| 3260 | CfaAffx.5065.1.S1_at | ZNF678 | zinc finger protein 678 | 3.20E-12 | -12.59 |
| 3261 | CfaAffx.265.1.S1_s_at | LOC607020 /// LOC612115 | similar to Ig lambda chain V-I region BL2 precursor /// similar to Ig lambda cha | 1.26E-08 | -12.97 |
| 3262 | CfaAffx.346.1.S1_at | LOC486380 | similar to Ig lambda chain V-I region BL2 precursor | 1.95E-05 | -13.19 |
| 3263 | CfaAffx.20958.1.S1_at | LOC607200 | similar to Ig lambda chain V-I region BL2 precursor | 4.70E-05 | -13.47 |
| 3264 | Cfa.6237.1.S1_at | TSPAN8 | tetraspanin 8 | 2.10E-10 | -13.93 |
| 3265 | CfaAffx.6656.1.S1_s_at | LOC609053 | similar to T-cell receptor beta chain V region CTL-L17 precursor | 1.28E-09 | -14.20 |
| 3266 | Cfa.4560.1.S1_s_at | AICDA | activation-induced cytidine deaminase | 1.50E-05 | -14.21 |
| 3267 | CfaAffx.23613.1.S1_x_at | LOC491492 | similar to Ig kappa chain V-II region RPMI 6410 precursor | 9.29E-06 | -15.17 |
| 3268 | CfaAffx.6656.1.S1_at | LOC609053 | similar to T-cell receptor beta chain V region CTL-L17 precursor | 1.46E-09 | -15.38 |
| 3269 | Cfa.337.1.A1_at | --- | --- | 1.04E-10 | -15.54 |
| 3270 | CfaAffx.246.1.S1_x_at | LOC486389 | similar to Ig lambda chain V-I region BL2 precursor | 1.23E-09 | -15.60 |
| 3271 | Cfa.6201.1.A1_at | VPREB1 | pre-B lymphocyte 1 | 2.60E-07 | -15.78 |
| 3272 | CfaAffx.21035.1.S1_at | LOC486393 | similar to Ig lambda chain V-II region MGC | 9.39E-08 | -15.85 |
| 3273 | CfaAffx.20950.1.S1_at | LOC486386 /// LOC491454 /// LOC606953 /// LOC612050 /// LOC612054 /// LOC612081 /// LOC612104 /// LOC612128 /// LOC612135 /// LOC612173 | similar to Ig lambda chain V-I region BL2 precursor /// similar to Ig lambda cha | 9.56E-09 | -15.92 |
| 3274 | CfaAffx.19330.1.S1_at | FABP2 | fatty acid binding protein 2, intestinal | 0.000110063 | -16.00 |
| 3275 | CfaAffx.20815.1.S1_x_at | LOC612050 | similar to Ig lambda chain V-I region BL2 precursor | 5.29E-05 | -16.15 |
| 3276 | CfaAffx.19414.1.S1_s_at | CE5 | CE5 protein | 3.25E-12 | -16.73 |
| 3277 | Cfa.10078.1.A1_at | PRKCQ | protein kinase C, theta | 4.60E-11 | -16.86 |
| 3278 | Cfa.8871.1.A1_at | --- | --- | 1.38E-11 | -17.74 |
| 3279 | CfaAffx.345.1.S1_s_at | LOC607125 | similar to Ig lambda chain V region 4A precursor | 1.22E-07 | -18.54 |
| 3280 | Cfa.3557.1.S2_s_at | CTLA4 | cytotoxic T-lymphocyte-associated protein 4 | 4.56E-09 | -20.10 |
| 3281 | CfaAffx.23723.1.S1_x_at | LOC606953 /// LOC612054 | similar to Ig lambda chain V-I region BL2 precursor /// similar to Ig lambda cha | 5.07E-11 | -20.95 |
| 3282 | CfaAffx.23584.1.S1_at | LOC491391 /// LOC491492 /// LOC491686 /// LOC607996 /// LOC608202 /// LOC608379 /// LOC609845 /// LOC610282 /// LOC611931 | similar to Ig kappa chain V-II region 26-10 /// similar to Ig kappa chain V-II r | 1.25E-06 | -21.34 |
| 3283 | CfaAffx.20953.1.S1_x_at | LOC486386 /// LOC491454 /// LOC606941 /// LOC606953 /// LOC607152 /// LOC612050 /// LOC612054 /// LOC612081 /// LOC612128 /// LOC612135 | similar to Ig lambda chain V-I region BL2 precursor /// similar to Ig lambda cha | 3.82E-10 | -22.01 |
| 3284 | Cfa.12371.1.A1_at | FMO2 | flavin containing monooxygenase 2 (non-functional) | 5.20E-07 | -22.42 |
| 3285 | Cfa.3450.1.S1_s_at | CCL17 | chemokine (C-C motif) ligand 17 | 1.84E-14 | -25.68 |
| 3286 | Cfa.280.1.S1_at | dla88 | MHC class I DLA-88 | 0.00396989 | -30.39 |

Table showing the differentialy expressed probesets identified by one-way ANOVA in the canine dataset (GSE30881). Using a FDR adjusted *p-value* of less than 0.05 and an absolute fold change of greater than 2, 3286 probesets were found to be differentially expressed between the healthy and DLBCL samples. Of these, 926 probesets were up-regulated and 2360 probesets were down-regulated in canine DLBCL.
