# Supplementary material for: Comparative Gene Expression Profiling Identifies Common Molecular Signatures of NF-κB Activation in Canine and Human Diffuse Large B Cell Lymphoma (DLBCL)
Source: PLoS One. 2013 Sep 4;8(9):e72591. doi: 10.1371/journal.pone.0072591 (PMC3762807; doi:10.1371/journal.pone.0072591)
Supplement: File S2 — ST 5: Gene Ontology (BP) enrichment in the up-regulated probesets in canine DLBCL (DAVID functional annotation chart, ranked on FDR). ST 6: Gene Ontology (BP) enrichment in the down-regulated probesets in canine DLBCL (DAVID functional annotation chart, ranked on FDR). ST 7: Kegg pathway hits in the up-regulated probesets in canine DLBCL (DAVID functional annotation chart, ranked on FDR). ST 8: Kegg pathway hits in the down-regulated probesets in canine DLBCL (DAVID functional annotation chart, ranked on FDR). (DOC) [file pone.0072591.s002.doc]

**Supplemental Data 2**

**ST 5: Gene Ontology (BP) enrichment in the up-regulated probesets in canine DLBCL (DAVID functional annotation chart, ranked on FDR)**

| **Sr. No.** | **Enriched Gene Ontology** | **Number of genes present in the query set that are involved in the Enriched Gene Ontology** | **Modified Fisher Exact *P-Value*** | **FDR** |
| --- | --- | --- | --- | --- |
| 1 | cell cycle process | 6 | 1.97E-05 | 0.02796062 |
| 2 | M phase | 5 | 9.36E-05 | 0.13281709 |
| 3 | cell cycle | 6 | 1.04E-04 | 0.14790488 |
| 4 | cell cycle phase | 5 | 2.12E-04 | 0.30125142 |
| 5 | chromosome | 4 | 7.29E-04 | 0.69392901 |
| 6 | meiosis | 3 | 0.00230139 | 3.21889051 |
| 7 | M phase of meiotic cell cycle | 3 | 0.00230139 | 3.21889051 |
| 8 | meiotic cell cycle | 3 | 0.00230139 | 3.21889051 |
| 9 | intracellular non-membrane-bounded organelle | 6 | 0.00372035 | 3.49464306 |
| 10 | non-membrane-bounded organelle | 6 | 0.00372035 | 3.49464306 |
| 11 | ATP binding | 7 | 0.00437764 | 4.67035971 |
| 12 | adenyl ribonucleotide binding | 7 | 0.00437764 | 4.67035971 |
| 13 | DNA replication | 3 | 0.00452322 | 6.23489506 |
| 14 | cell division | 4 | 0.00481031 | 6.61816933 |
| 15 | spindle | 3 | 0.00815202 | 7.51449543 |
| 16 | DNA metabolic process | 4 | 0.00737334 | 9.97588367 |
| 17 | adenyl nucleotide binding | 7 | 0.0096663 | 10.0479908 |
| 18 | RNA binding | 4 | 0.0096865 | 10.0679821 |
| 19 | purine nucleoside binding | 7 | 0.01320823 | 13.4938337 |
| 20 | nucleoside binding | 7 | 0.01320823 | 13.4938337 |
| 21 | cellular response to stress | 4 | 0.01250629 | 16.3655488 |
| 22 | microtubule cytoskeleton | 3 | 0.02155973 | 18.779899 |
| 23 | DNA repair | 3 | 0.01502643 | 19.345963 |
| 24 | ribonucleotide binding | 7 | 0.03374963 | 31.2221086 |
| 25 | purine ribonucleotide binding | 7 | 0.03374963 | 31.2221086 |
| 26 | condensed chromosome | 2 | 0.0540047 | 41.1299209 |
| 27 | response to DNA damage stimulus | 3 | 0.0367215 | 41.2146058 |
| 28 | 2'-5'-oligoadenylate synthetase activity | 2 | 0.05169184 | 43.9332722 |
| 29 | adenylyltransferase activity | 2 | 0.05169184 | 43.9332722 |
| 30 | damaged DNA binding | 2 | 0.05169184 | 43.9332722 |
| 31 | purine nucleotide binding | 7 | 0.05356653 | 45.1298526 |
| 32 | nucleotide binding | 7 | 0.05783521 | 47.7684056 |
| 33 | DNA binding | 5 | 0.06380158 | 51.2633996 |
| 34 | cell proliferation | 3 | 0.05031213 | 51.9560323 |
| 35 | DNA-dependent DNA replication | 2 | 0.05644007 | 56.1756257 |
| 36 | structure-specific DNA binding | 2 | 0.07657838 | 58.0440011 |
| 37 | cell death | 4 | 0.06819092 | 63.3200182 |
| 38 | death | 4 | 0.06819092 | 63.3200182 |
| 39 | ribonucleoside metabolic process | 2 | 0.08350941 | 71.0131393 |
| 40 | nucleoside metabolic process | 2 | 0.08350941 | 71.0131393 |
| 41 | cellular macromolecule catabolic process | 3 | 0.09951464 | 77.429112 |

**ST 6:** **Gene Ontology (BP) enrichment in the down-regulated probesets in canine DLBCL (DAVID functional annotation chart, ranked on FDR)**

| **Sr. No.** | **Enriched Gene Ontology** | **Number of genes present in the query set that are involved in the Enriched Gene Ontology** | **Modified Fisher Exact *P-Value*** | **FDR** |
| --- | --- | --- | --- | --- |
| 1 | regulation of cell proliferation | 15 | 8.08E-04 | 1.23339799 |
| 2 | cell surface | 11 | 0.002598624 | 2.83607312 |
| 3 | lymphocyte activation | 8 | 0.002802738 | 4.21744368 |
| 4 | immune response | 19 | 0.003696948 | 5.52762934 |
| 5 | positive regulation of cell proliferation | 10 | 0.005063267 | 7.49722573 |
| 6 | leukocyte activation | 8 | 0.005848382 | 8.61155976 |
| 7 | regulation of production of molecular mediator of immune response | 5 | 0.006268587 | 9.20279906 |
| 8 | regulation of immune effector process | 5 | 0.006268587 | 9.20279906 |
| 9 | cell activation | 8 | 0.008076284 | 11.7057331 |
| 10 | immune system development | 8 | 0.008076284 | 11.7057331 |
| 11 | positive regulation of leukocyte activation | 6 | 0.011247191 | 15.941021 |
| 12 | positive regulation of cell activation | 6 | 0.011247191 | 15.941021 |
| 13 | positive regulation of lymphocyte activation | 6 | 0.011247191 | 15.941021 |
| 14 | regulation of leukocyte mediated immunity | 4 | 0.016296748 | 22.2955646 |
| 15 | regulation of lymphocyte mediated immunity | 4 | 0.016296748 | 22.2955646 |
| 16 | positive regulation of signal transduction | 6 | 0.016467973 | 22.5029542 |
| 17 | positive regulation of leukocyte proliferation | 5 | 0.018428939 | 24.841491 |
| 18 | positive regulation of lymphocyte proliferation | 5 | 0.018428939 | 24.841491 |
| 19 | positive regulation of mononuclear cell proliferation | 5 | 0.018428939 | 24.841491 |
| 20 | external side of plasma membrane | 7 | 0.030940827 | 29.3562465 |
| 21 | regulation of lymphocyte activation | 6 | 0.023088688 | 30.1364815 |
| 22 | positive regulation of cell communication | 6 | 0.023088688 | 30.1364815 |
| 23 | positive regulation of immune system process | 6 | 0.023088688 | 30.1364815 |
| 24 | hemopoiesis | 7 | 0.024158154 | 31.3015043 |
| 25 | hemopoietic or lymphoid organ development | 7 | 0.024158154 | 31.3015043 |
| 26 | extracellular space | 15 | 0.034140176 | 31.8927097 |
| 27 | regulation of lymphocyte proliferation | 5 | 0.027773755 | 35.1070647 |
| 28 | lymphocyte differentiation | 5 | 0.027773755 | 35.1070647 |
| 29 | regulation of leukocyte proliferation | 5 | 0.027773755 | 35.1070647 |
| 30 | regulation of mononuclear cell proliferation | 5 | 0.027773755 | 35.1070647 |
| 31 | positive regulation of phosphate metabolic process | 4 | 0.029618832 | 36.972242 |
| 32 | positive regulation of protein modification process | 4 | 0.029618832 | 36.972242 |
| 33 | positive regulation of phosphorus metabolic process | 4 | 0.029618832 | 36.972242 |
| 34 | positive regulation of peptidyl-tyrosine phosphorylation | 4 | 0.029618832 | 36.972242 |
| 35 | positive regulation of protein amino acid phosphorylation | 4 | 0.029618832 | 36.972242 |
| 36 | regulation of immunoglobulin production | 4 | 0.029618832 | 36.972242 |
| 37 | positive regulation of phosphorylation | 4 | 0.029618832 | 36.972242 |
| 38 | regulation of leukocyte activation | 6 | 0.031224811 | 38.5547994 |
| 39 | leukocyte differentiation | 6 | 0.031224811 | 38.5547994 |
| 40 | regulation of cell activation | 6 | 0.031224811 | 38.5547994 |
| 41 | cytokine activity | 12 | 0.036784047 | 38.6568518 |
| 42 | cytokine binding | 6 | 0.054133238 | 51.6009579 |
| 43 | positive regulation of protein metabolic process | 4 | 0.047131515 | 52.3456653 |
| 44 | positive regulation of T cell proliferation | 4 | 0.047131515 | 52.3456653 |
| 45 | positive regulation of cellular protein metabolic process | 4 | 0.047131515 | 52.3456653 |
| 46 | T cell differentiation | 4 | 0.047131515 | 52.3456653 |
| 47 | extracellular region part | 18 | 0.06734756 | 53.7417801 |
| 48 | regulation of T cell proliferation | 4 | 0.068614062 | 66.4214717 |
| 49 | regulation of peptidyl-tyrosine phosphorylation | 4 | 0.068614062 | 66.4214717 |
| 50 | regulation of isotype switching | 3 | 0.080330587 | 72.3525981 |
| 51 | positive regulation of isotype switching | 3 | 0.080330587 | 72.3525981 |
| 52 | positive regulation of B cell activation | 3 | 0.080330587 | 72.3525981 |
| 53 | positive regulation of DNA metabolic process | 3 | 0.080330587 | 72.3525981 |
| 54 | regulation of B cell mediated immunity | 3 | 0.080330587 | 72.3525981 |
| 55 | regulation of myeloid leukocyte differentiation | 3 | 0.080330587 | 72.3525981 |
| 56 | regulation of myeloid cell differentiation | 3 | 0.080330587 | 72.3525981 |
| 57 | positive regulation of DNA recombination | 3 | 0.080330587 | 72.3525981 |
| 58 | regulation of immunoglobulin mediated immune response | 3 | 0.080330587 | 72.3525981 |
| 59 | regulation of DNA recombination | 3 | 0.080330587 | 72.3525981 |
| 60 | regulation of B cell activation | 3 | 0.080330587 | 72.3525981 |
| 61 | positive regulation of isotype switching to IgG isotypes | 3 | 0.080330587 | 72.3525981 |
| 62 | regulation of isotype switching to IgG isotypes | 3 | 0.080330587 | 72.3525981 |
| 63 | T cell activation | 4 | 0.093706624 | 77.921631 |
| 64 | positive regulation of T cell activation | 4 | 0.093706624 | 77.921631 |

**ST 7:** **Kegg pathway hits in the up-regulated probesets in canine DLBCL (DAVID functional annotation chart, ranked on FDR)**

| **Sr. No.** | **Name of the KEGG Pathways** | **Number of genes present in the query set that are involved in the pathway** | **Modified Fisher Exact P-Value** | **FDR** |
| --- | --- | --- | --- | --- |
| 1 | Cell cycle | 33 | 4.72E-20 | 5.54E-17 |
| 2 | DNA replication | 13 | 9.23E-10 | 1.08E-06 |
| 3 | p53 signaling pathway | 12 | 1.19E-05 | 0.013932775 |
| 4 | Oocyte meiosis | 13 | 4.17E-04 | 0.487577135 |
| 5 | Mismatch repair | 6 | 7.35E-04 | 0.85809733 |
| 6 | Pyrimidine metabolism | 11 | 8.68E-04 | 1.01319084 |
| 7 | One carbon pool by folate | 5 | 0.001637102 | 1.902531852 |
| 8 | Purine metabolism | 14 | 0.001907753 | 2.213855862 |
| 9 | Homologous recombination | 5 | 0.011449659 | 12.62900552 |
| 10 | Nucleotide excision repair | 6 | 0.016677457 | 17.89492746 |
| 11 | Butanoate metabolism | 5 | 0.017043081 | 18.25212417 |
| 12 | Synthesis and degradation of ketone bodies | 3 | 0.02412844 | 24.89952812 |
| 13 | Base excision repair | 5 | 0.029644617 | 29.72825233 |
| 14 | Progesterone-mediated oocyte maturation | 8 | 0.042833163 | 40.14441297 |
| 15 | Cysteine and methionine metabolism | 5 | 0.05459551 | 48.22160456 |
| 16 | Ubiquitin mediated proteolysis | 9 | 0.090085429 | 66.93753232 |

**ST 8:** **Kegg pathway hits in the down-regulated probesets in canine DLBCL (DAVID functional annotation chart, ranked on FDR)**

| **Sr. No.** | **Name of the KEGG Pathways** | **Number of genes present in the query set that are involved in the pathway** | **Modified Fisher Exact P-Value** | **FDR** |
| --- | --- | --- | --- | --- |
| 1 | Cytokine-cytokine receptor interaction | 39 | 9.31E-09 | 1.12E-05 |
| 2 | Cell adhesion molecules (CAMs) | 27 | 8.68E-07 | 0.001041992 |
| 3 | Hematopoietic cell lineage | 21 | 1.01E-06 | 0.00121782 |
| 4 | Intestinal immune network for IgA production | 14 | 1.19E-05 | 0.014316277 |
| 5 | ECM-receptor interaction | 18 | 6.56E-05 | 0.078733131 |
| 6 | Allograft rejection | 10 | 1.61E-04 | 0.192867997 |
| 7 | Complement and coagulation cascades | 15 | 1.94E-04 | 0.232926 |
| 8 | Focal adhesion | 30 | 2.43E-04 | 0.291958635 |
| 9 | Viral myocarditis | 14 | 3.54E-04 | 0.424588676 |
| 10 | Drug metabolism | 11 | 8.40E-04 | 1.004171553 |
| 11 | Autoimmune thyroid disease | 10 | 0.001012316 | 1.208976424 |
| 12 | Type I diabetes mellitus | 10 | 0.001259794 | 1.502485916 |
| 13 | T cell receptor signaling pathway | 19 | 0.001330781 | 1.586528703 |
| 14 | Dilated cardiomyopathy | 16 | 0.001628733 | 1.938564252 |
| 15 | Hypertrophic cardiomyopathy (HCM) | 15 | 0.002486397 | 2.945474416 |
| 16 | PPAR signaling pathway | 13 | 0.005138708 | 5.999656773 |
| 17 | Arrhythmogenic right ventricular cardiomyopathy (ARVC) | 13 | 0.005800765 | 6.748158147 |
| 18 | Graft-versus-host disease | 7 | 0.008211985 | 9.428259367 |
| 19 | Primary immunodeficiency | 8 | 0.009505497 | 10.83673416 |
| 20 | Vascular smooth muscle contraction | 17 | 0.010331213 | 11.72530675 |
| 21 | Metabolism of xenobiotics by cytochrome P450 | 8 | 0.01807534 | 19.67258841 |
| 22 | Pathways in cancer | 36 | 0.019774119 | 21.32573316 |
| 23 | Adherens junction | 12 | 0.020745143 | 22.25660535 |
| 24 | Tryptophan metabolism | 8 | 0.023942612 | 25.25096034 |
| 25 | Chemokine signaling pathway | 21 | 0.032491867 | 32.74554313 |
| 26 | Endocytosis | 22 | 0.047500757 | 44.2586319 |
| 27 | Histidine metabolism | 5 | 0.062783954 | 54.09999736 |
| 28 | Axon guidance | 16 | 0.065883516 | 55.89020128 |
| 29 | Arachidonic acid metabolism | 8 | 0.066166031 | 56.05014694 |
