# Supplementary material for: Comparative Gene Expression Profiling Identifies Common Molecular Signatures of NF-κB Activation in Canine and Human Diffuse Large B Cell Lymphoma (DLBCL)
Source: PLoS One. 2013 Sep 4;8(9):e72591. doi: 10.1371/journal.pone.0072591 (PMC3762807; doi:10.1371/journal.pone.0072591)
Supplement: File S3 — SF 1: Positive co-expression networks of canine and human DLBCLs. SF 2: Clusters identified from the co-expression network of human DLBCL. SF 3: Ingenuity Pathway Analysis results showing the top canonical pathways enriched in the up-regulated probesets of canine DLBCL. SF 4: Ingenuity Pathway Analysis results showing the top canonical pathways enriched in the down-regulated probesets of canine DLBCL. SF 5: Ingenuity Pathway Analysis results showing the top bio functions enriched in the up-regulated probesets of canine DLBCL. SF 6: Ingenuity Pathway Analysis results showing the top bio functions enriched in the down-regulated probesets of canine DLBCL. SF 7: Comparison of enrichment of KEGG pathways in the differentially expressed probesets of canine DLBCL and human DLBCL. SF 8: Comparison of enrichment of signalling pathways in the differentially expressed probesets of canine DLBCL and human DLBCL in IPA. SF 9: Comparison of enrichment of NF-κB signalling pathway in differentially expressed genes in canine lymphoma and human DLBCL using IPA. SF 10: Clusters identified from the co-expression network of canine DLBCL. SF 11: Flow chart of the bioinformatics analysis. SF 12: Hierarchical clustering and principal components analysis of the canine dataset GSE30881 showing the clustering of normal, naïve and relapsed samples. (DOC) [file pone.0072591.s003.doc]

**
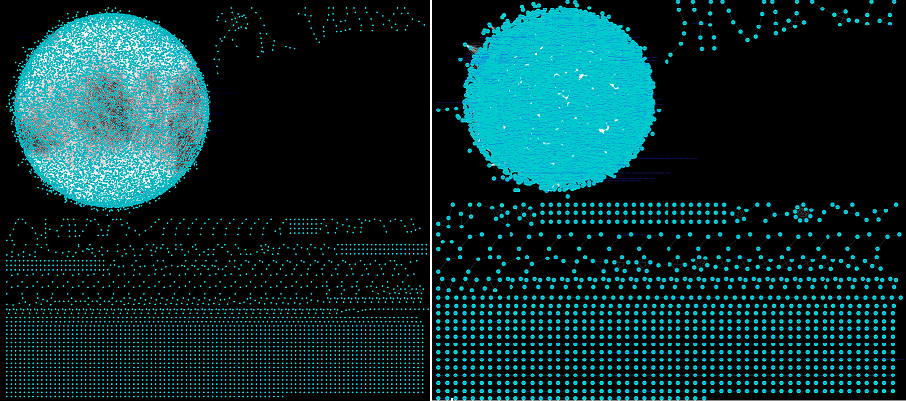
**

1. **(B)**

**SF 1: Positive co-expression networks of canine and human DLBCLs**

Co-expression networks of the positively correlated probesets in the canine (A) and human (B) datasets visualised in Cytoscape 2.6. Only the probesets that were significantly positively correlated at *p-value* less than 0.0001 with a statistical power of 0.8 were included in these networks. The probesets (nodes) are shown as cyan circles and the relationship among the probesets are shown in white interconnecting lines (edges). Since these figures are highly condensed, the individual nodes and edges are not recognisable.

1. The canine DLBCL co-expression network having 16,852 nodes and 120,270 edges was constructed using the array data from the 23 DLBCL samples.
2. The human DLBCL co-expression network having 21,435 nodes and 421,777 edges was constructed using the array data from the 45 DLBCL samples.

**
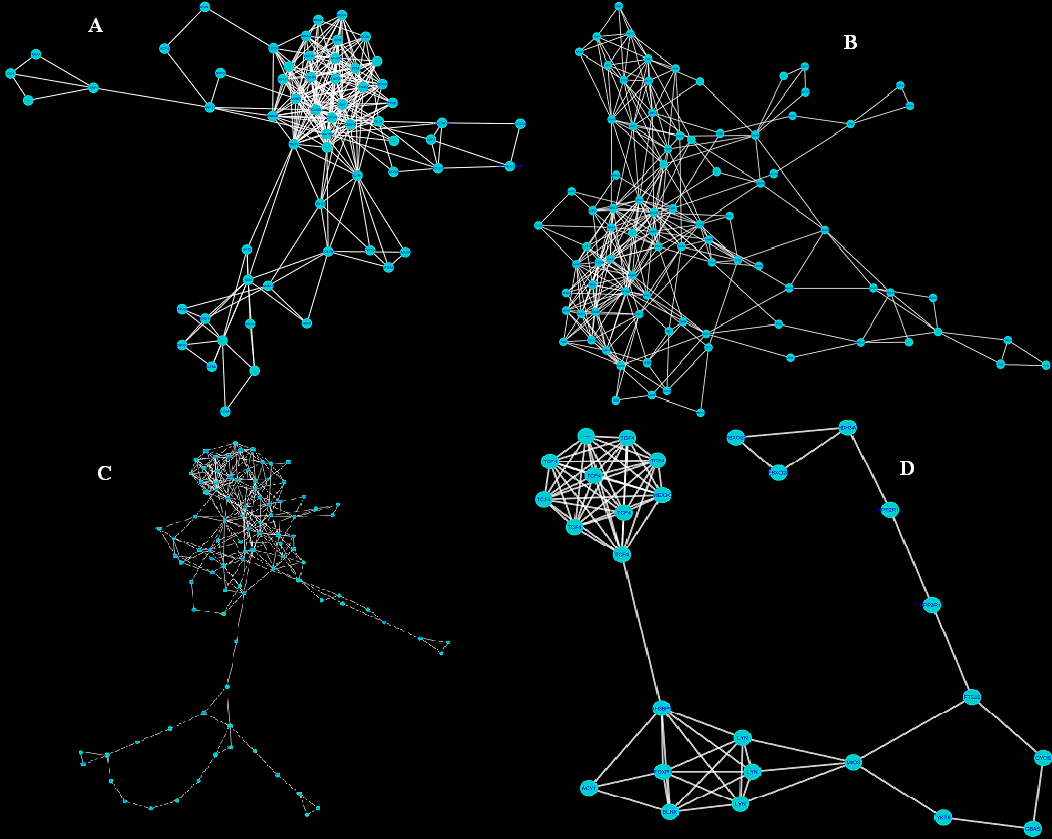
**

**SF 2: Clusters identified from the co-expression network of human DLBCL**

Cytoscapic view of some of the clusters identified using MCODE clustering algorithm in the co-expression network of human DLBCL. The clusters were numbered according to their rank in MCODE result. The probesets (nodes) are shown as cyan circles and labelled with their relevant gene symbol. Where gene annotation is not available, ‘---’ is used as label. The relationships among the probesets are shown in white interconnecting lines (edges).

1. Cluster no. 14: This cluster has 60 nodes and 271 edges. TREM1signalling, IL-10 signalling, IL-6 signalling and p-53 signalling pathways are significantly enriched while cell death is the top most IPA bio function enriched in this cluster.
2. Cluster no. 23: This cluster has 87 nodes and 320 edges. Toll-like receptor signalling, TREM1 signalling, inhibition of angiogenesis by TSP1 signalling and NF-κB signalling pathways are significantly enriched in this cluster while cellular movement is the top most IPA bio function enriched in this cluster.
3. Cluster no. 30: This cluster has 98 nodes and 315 edges. G-protein coupled receptor signalling, Wnt/β-catenin signalling, PTEN signalling, IL-15 signalling, IL-17 signalling, NF-κB signalling, PI3K/AKT signalling and JAK/Stat signalling are the significantly enriched pathways in this cluster while cellular movement is the top most IPA bio function enriched in this cluster.
4. Cluster no. 37: This cluster has 27 nodes and 78 edges. Fc-γ-RIIB signalling, Ceramide signalling, PI3K signalling in B lymphocytes, Wnt/β-catenin signalling, TWEAK signalling and cell cycle regulation by BTG family proteins signalling are the significantly enriched pathways in this cluster while cellular development is the top most IPA bio function enriched in this cluster.

**
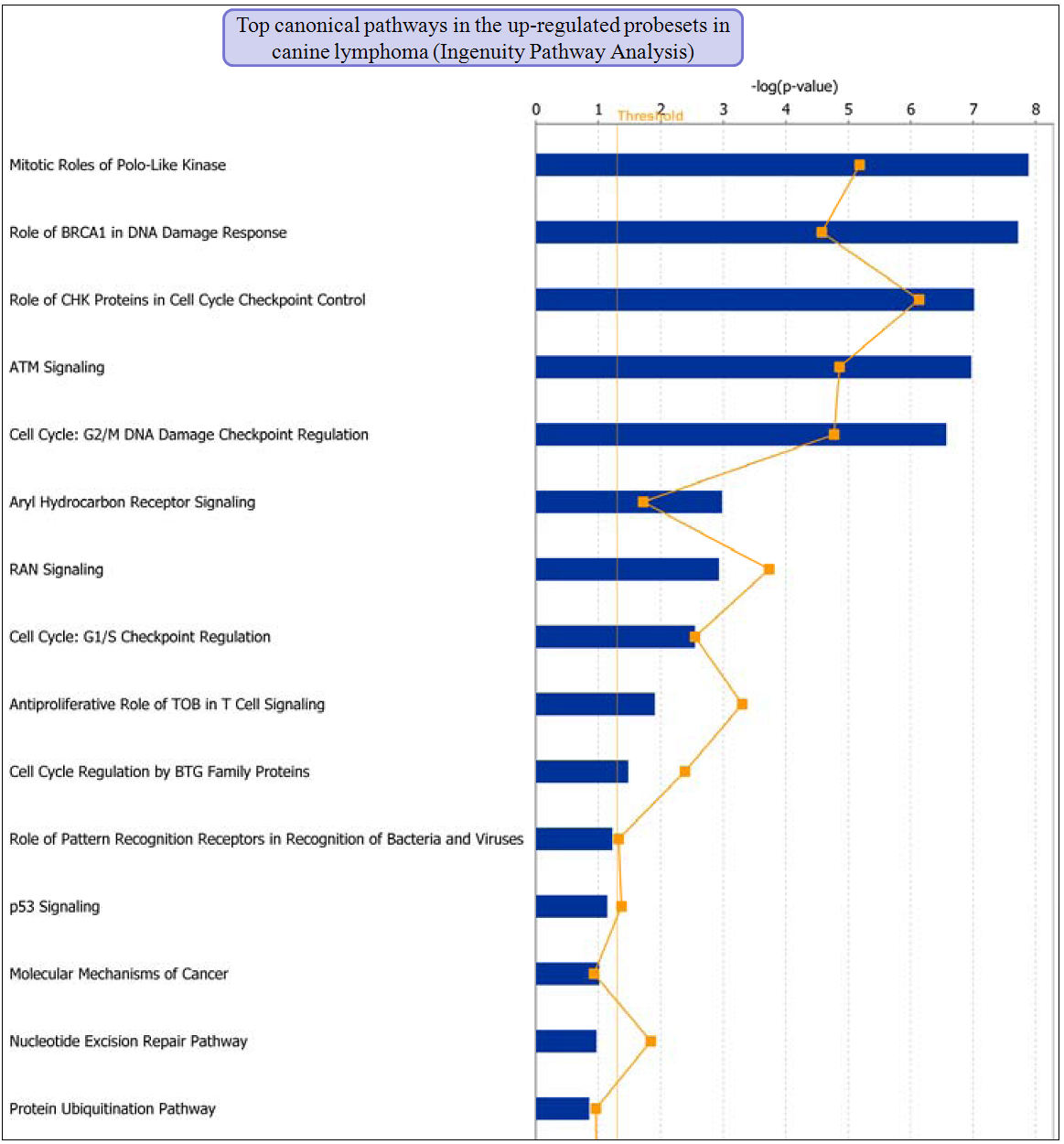
**

**SF 3: Ingenuity Pathway Analysis results showing the top canonical pathways enriched in the up-regulated probesets of canine DLBCL**

The canonical pathways that are enriched in the 926 up-regulated probesets in canine DLBCL are shown as bar charts in this figure. The length of the bars corresponds to the negative log of *p-value* of enrichment score for the canonical pathway enriched in the gene set.


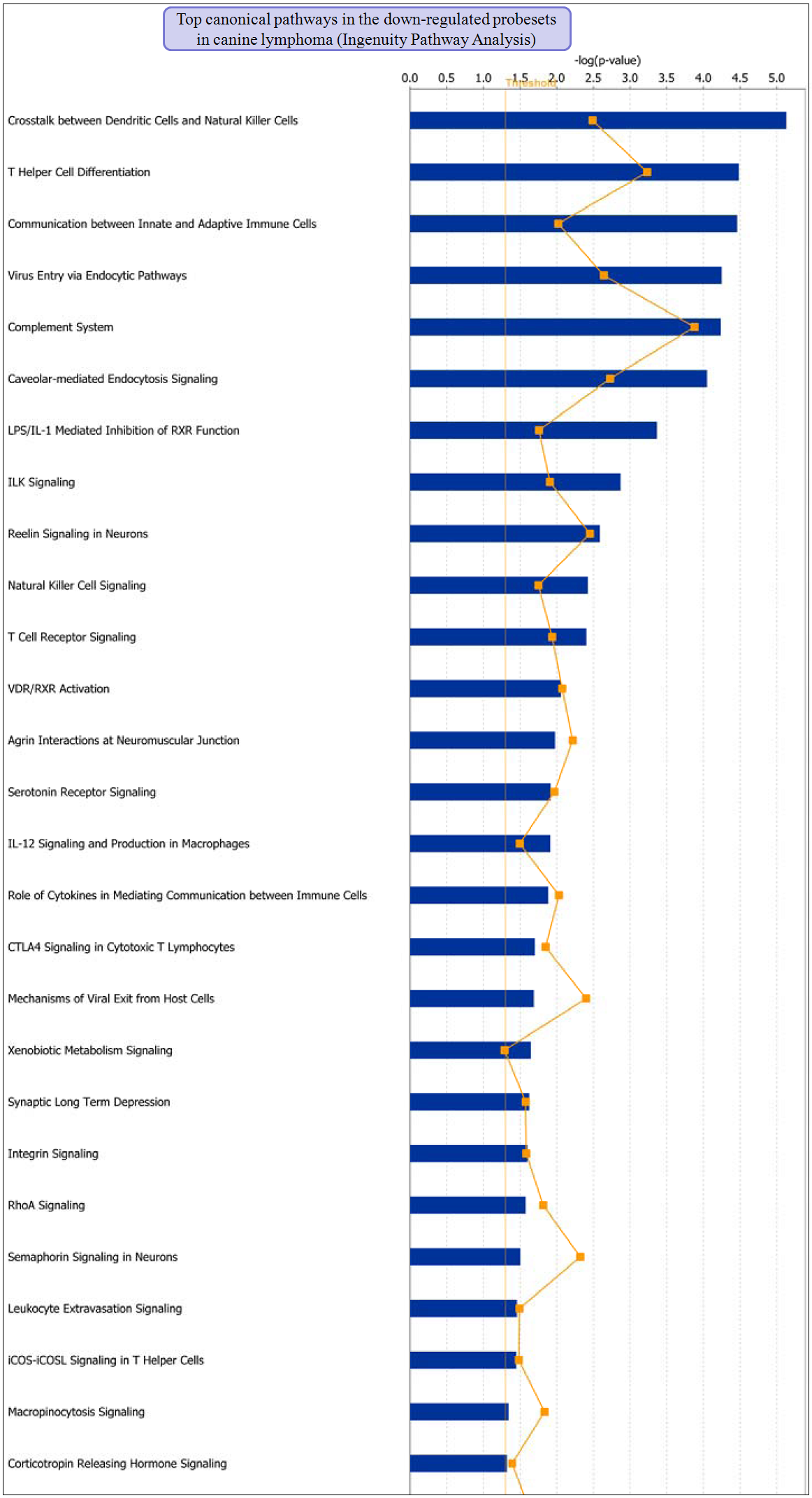


**SF 4: Ingenuity Pathway Analysis results showing the top canonical pathways enriched in the down-regulated probesets of canine DLBCL**

The canonical pathways that are enriched in the 2360 down-regulated probesets in canine DLBCL are shown as bar charts in this figure. The length of the bars corresponds to the negative log of *p-value* of enrichment score for the canonical pathway enriched in the gene set.

**
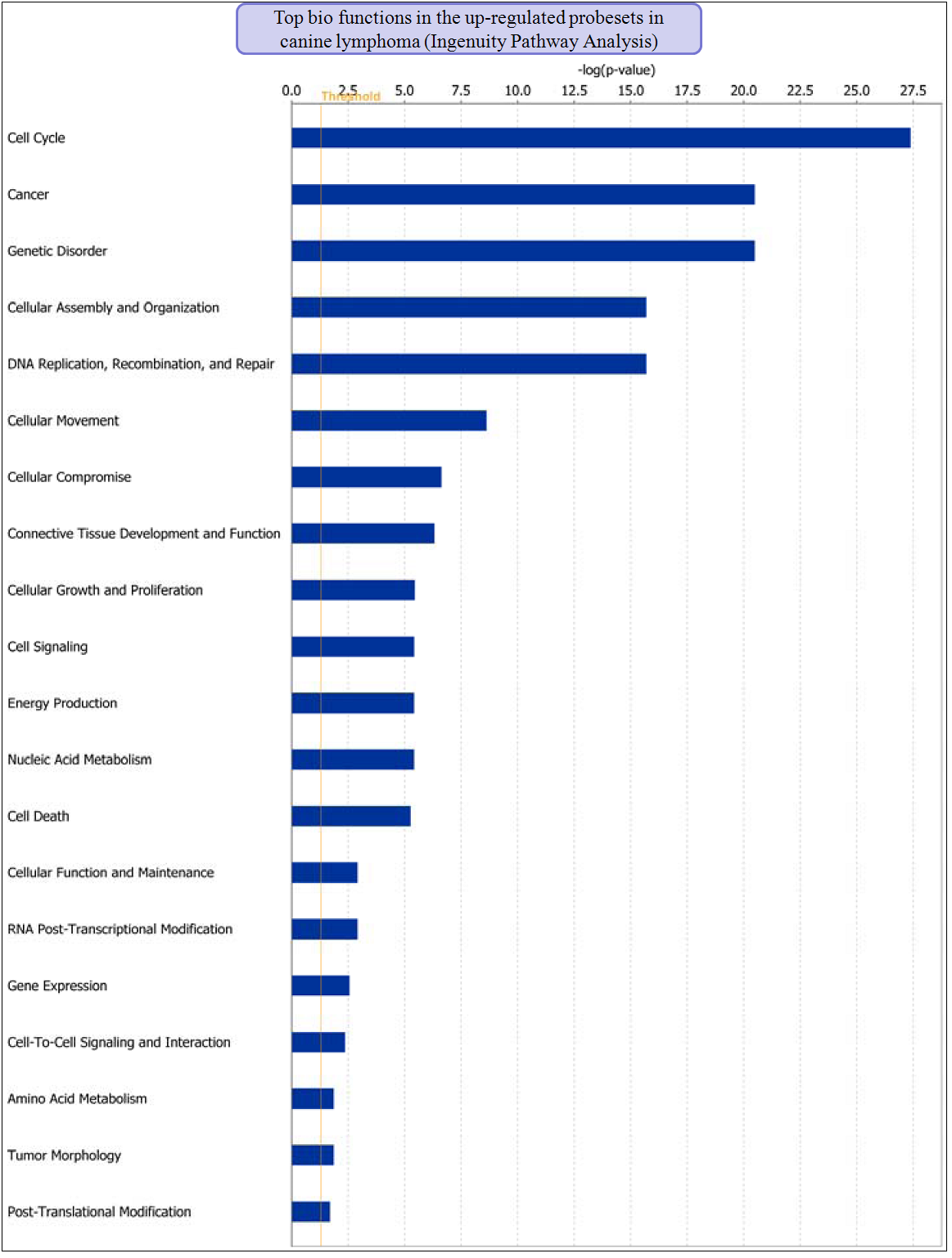
**

**SF 5: Ingenuity Pathway Analysis results showing the top bio functions enriched in the up-regulated probesets of canine DLBCL**

Enrichment of the IPA bio functions in the 926 up-regulated probesets in canine DLBCL are shown as bar charts in this figure. The length of the bars corresponds to the negative log of *p-value* of enrichment score for the canonical pathway enriched in the gene set.

**
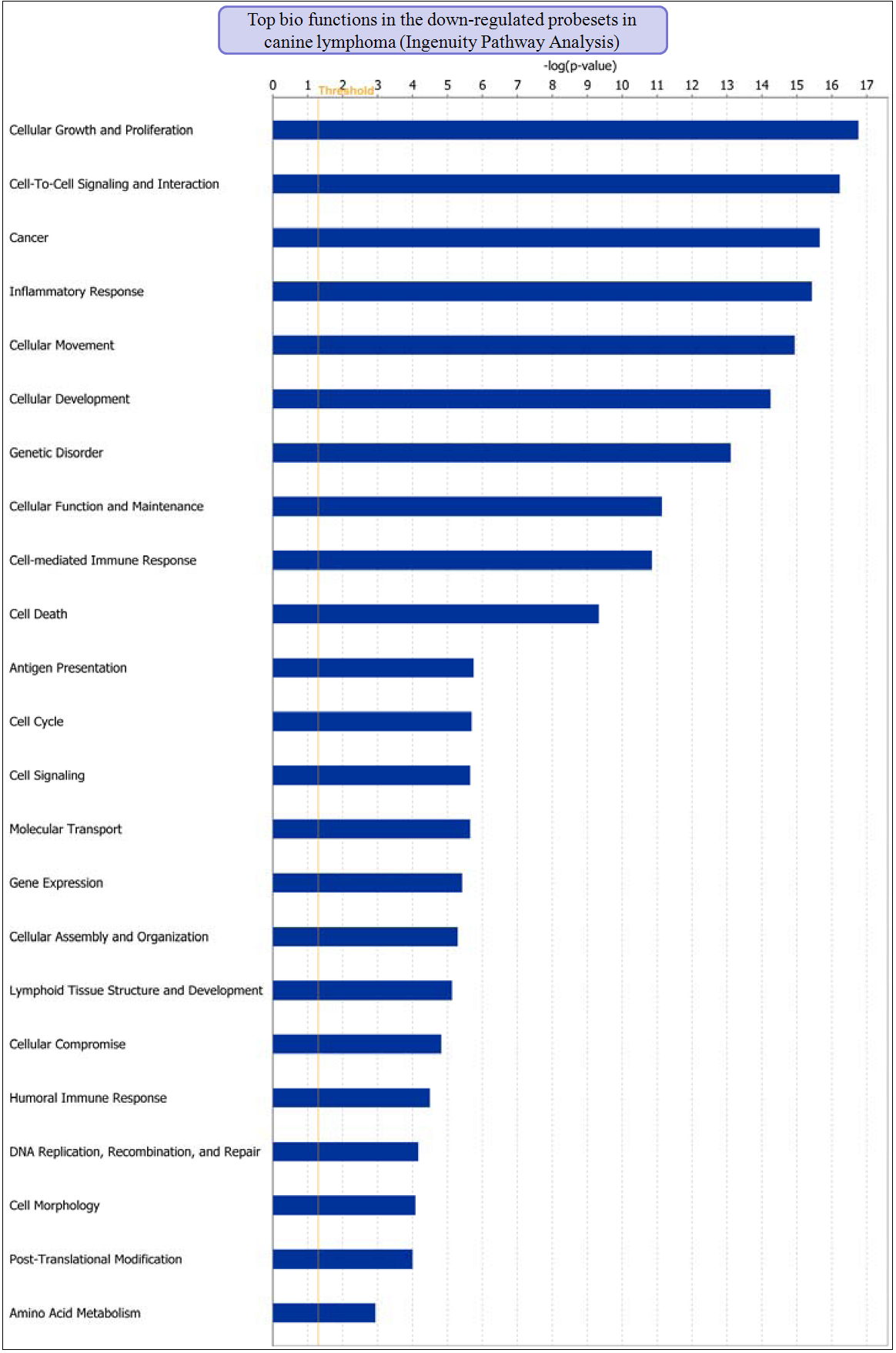
**

**SF 6: Ingenuity Pathway Analysis results showing the top bio functions enriched in the down-regulated probesets of canine DLBCL**

Enrichment of the IPA bio functions in the 2360 down -regulated probesets in canine DLBCL are shown as bar charts in this figure. The length of the bars corresponds to the negative log of *p-value* of enrichment score for the canonical pathway enriched in the gene set.

**
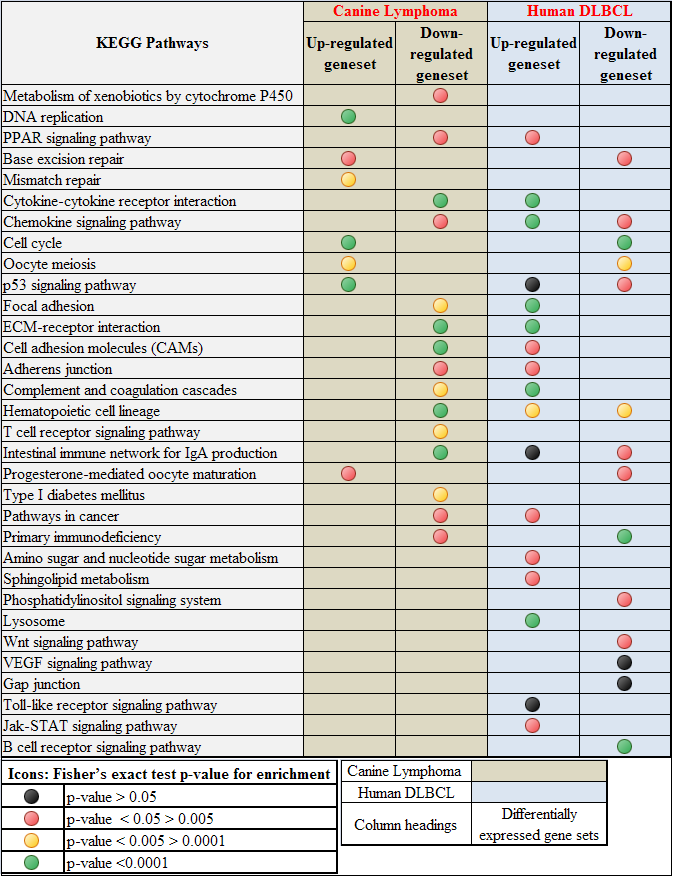
**

**SF 7: Comparison of enrichment of KEGG pathways in the differentially expressed probesets of canine DLBCL and human DLBCL**

The differentially expressed probesets of canine DLBCL and human DLBCL were analysed for enrichment of KEGG pathways using DAVID functional annotation tool. The results from the analysis are compiled and the *p-values* of the enrichment score computed by Fisher’s exact test are represented graphically as coloured icons.

**
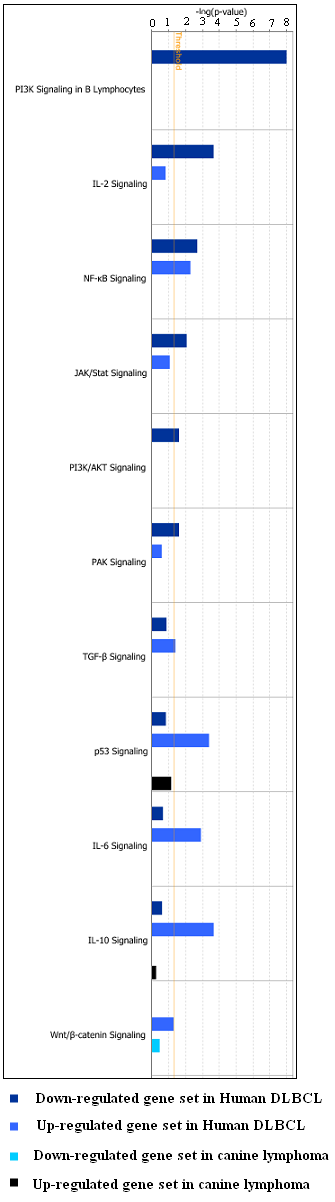
**

**SF 8: Comparison of enrichment of signalling pathways in the differentially expressed probesets of canine DLBCL and human DLBCL in IPA**

The differentially expressed probesets of canine DLBCL and human DLBCL were analysed for enrichment of signalling pathways in IPA. The result of the comparison analysis is represented graphically. The lengths of the bars show their significance, negative log of the *p-value* for the pathway computed by Fisher’s exact test, in the relevant pathway.

**
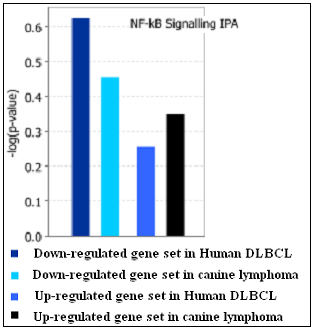
**

**SF 9: Comparison of enrichment of NF-κB signalling pathway in differentially expressed genes in canine lymphoma and human DLBCL using IPA**

The differentially expressed probesets of canine DLBCL and human DLBCL were analysed for enrichment of the NF-κB signalling pathway using IPA tool. The result of the comparison analysis is represented graphically. The heights of the bars show their significance (negative log of the *p-value* computed by Fisher’s exact test) of enrichment in NF-κB signalling pathway.

**
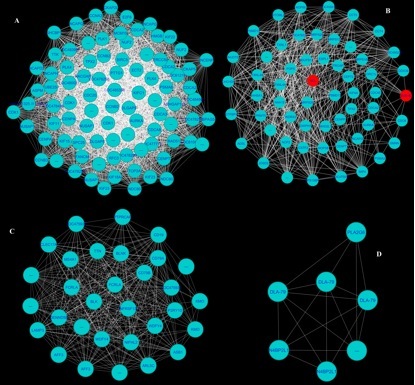
**

**SF 10: Clusters identified from the co-expression network of canine DLBCL**Cytoscapic view of some of the clusters identified using MCODE clustering algorithm in the co-expression network of canine DLBCL. The clusters were numbered according to their rank in MCODE result. The probesets (nodes) are shown as cyan circles and labelled with their relevant gene symbol. Where gene annotation is not available, ‘---’ is used as label. The relationships among the probesets are shown in white interconnecting lines (edges).

1. Cluster no. 2: This cluster has 88 nodes and 3132 edges. Cell Cycle Regulation signalling pathways (Mitotic roles of Polo-like kinase, Cell Cycle: G2/M DNA damage check point regulation and ATM signalling), role of BRCA1 in DNA damage response pathway and protein ubiquitination pathway are significantly enriched while cell cycle is the top most IPA bio function enriched in this cluster.
2. Cluster no. 6: This cluster has 57 nodes and 541edges. Cell cycle control of chromosomal replication pathway is significantly enriched in this cluster while cell cycle is the top most IPA bio function of this cluster. The two NF-κB target genes in this cluster are shown in red colour.
3. Cluster no. 7: This cluster has 31 nodes and 375 edges. PI3K signalling in B lymphocytes, B cell development, Fc-γ-RIIB signalling in B lymphocytes are significantly enriched in this cluster while cellular growth and proliferation is the top most IPA bio function of this cluster.
4. Cluster no. 26: This cluster has 7 nodes and 18 edges. Antigen presentation pathway and OX40 signalling pathwayare significantly enriched in this cluster while cellular development is the top most IPA bio function of this cluster.

**
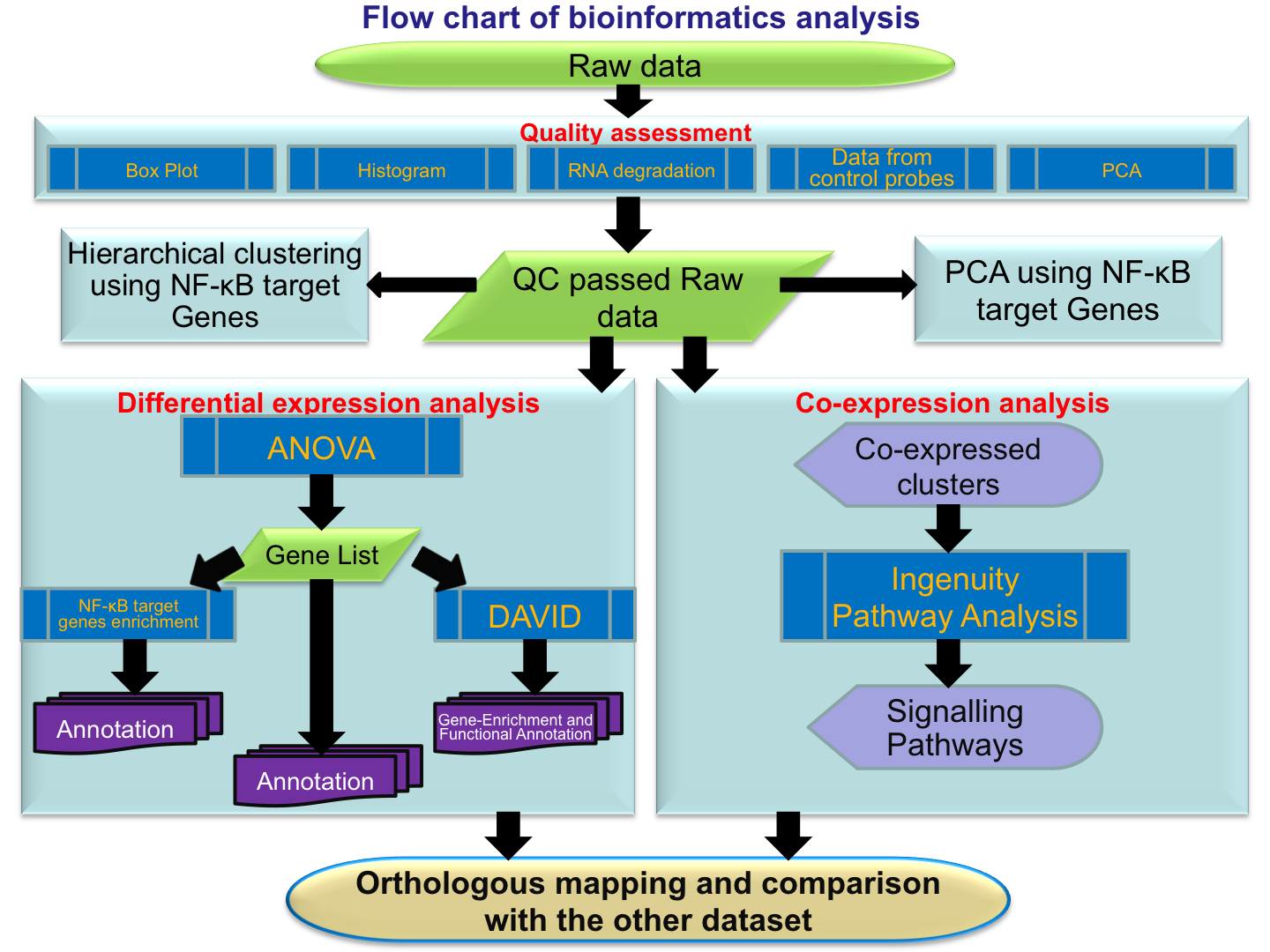
**

**SF 11: Flow chart of the bioinformatics analysis**

This figure shows the schematic representation of the workflow involved in the bioinformatics analyses. Both canine and human datasets were separately analysed using this flow chart and then their results were compared. The main steps in the workflow were:

1. Quality analysis
2. Hierarchical clustering of the dataset using NF-kappaB target gene set
3. Principal components analysis of the dataset using NF-kappaB target gene set
4. Differential expression analysis
5. Positive co-expression network analysis
6. Functional annotations of the gene lists and clusters

While differential expression analysis identified genes that were differentially expressed between cancer and normal samples, the co-expression network analysis clustered the genes in cancer samples that were expressed in similar fashion. Both the analyses are complementary for comparing the canine and human DLBCL.


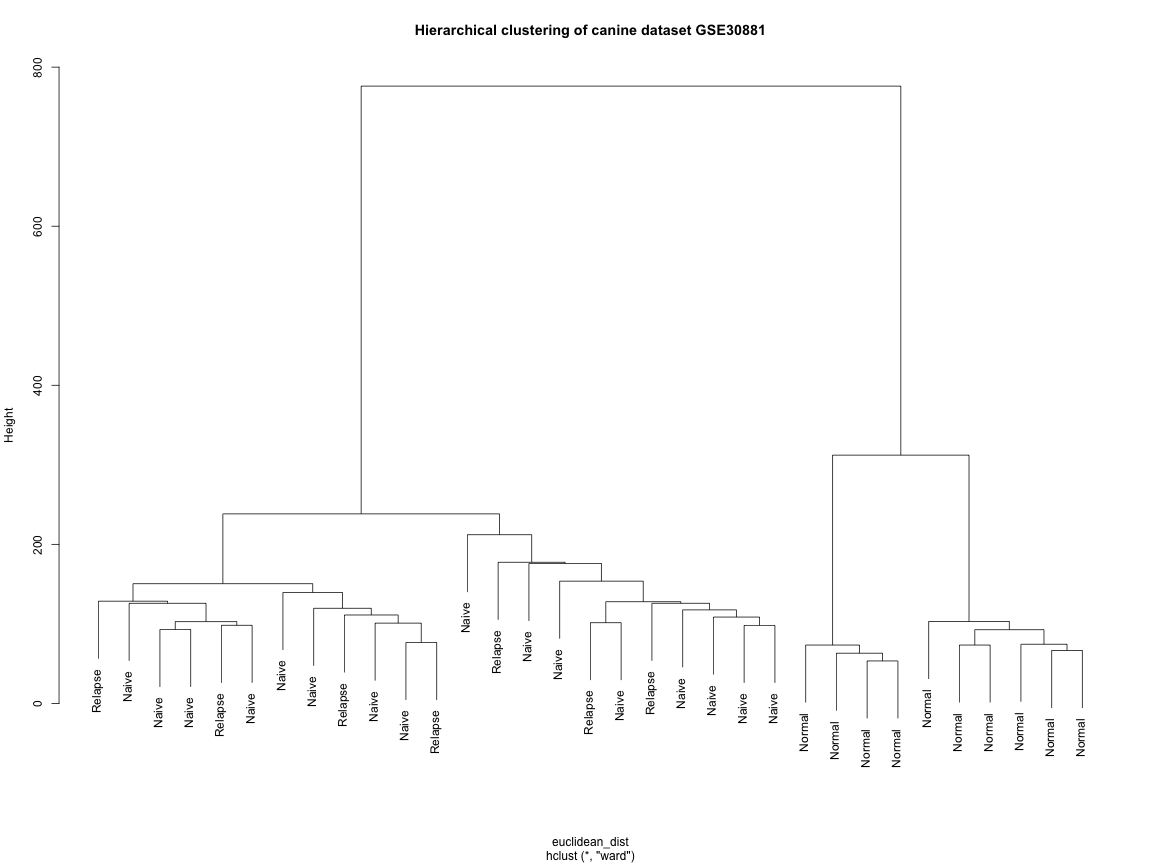


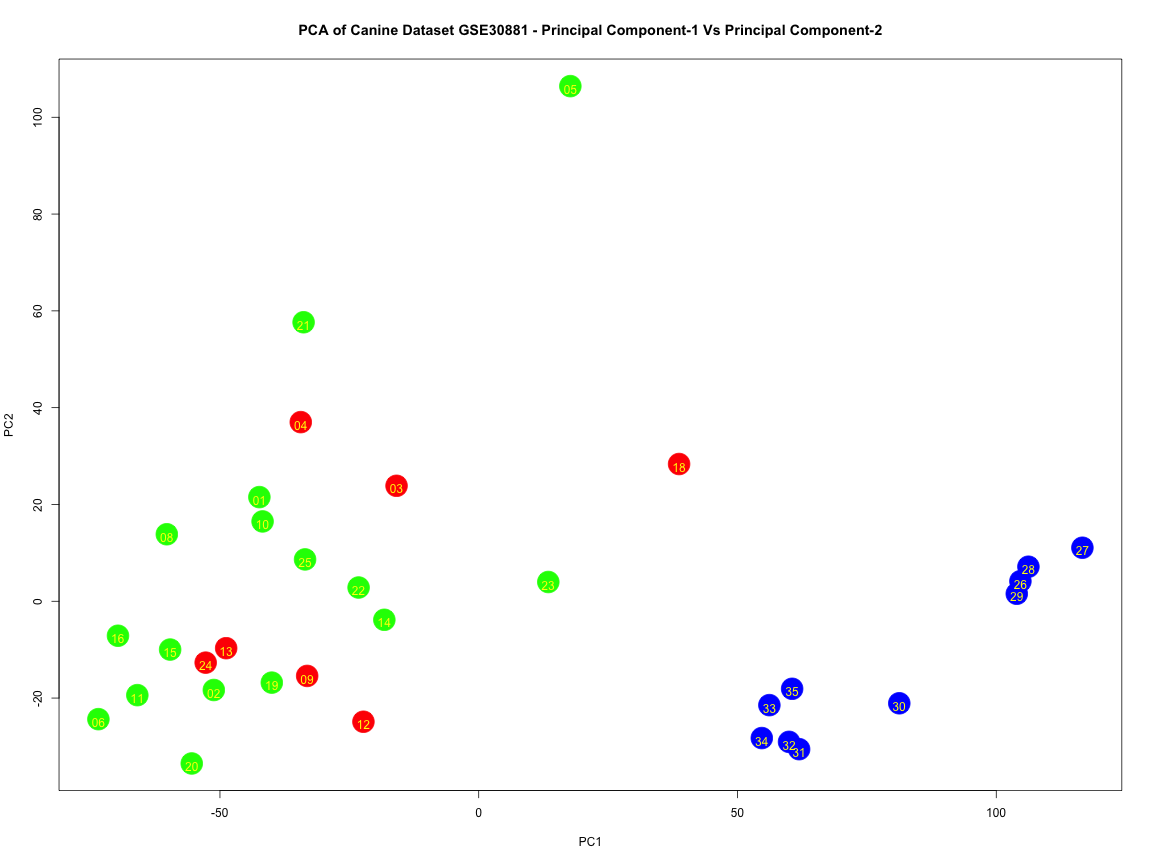


**SF 12: Hierarchical clustering and principal components analysis of the canine dataset GSE30881 showing the clustering of normal, naïve and relapsed samples**

Hierarchical clustering using Euclidean distance and Ward agglomeration method shows clustering of normal and cancer samples. However, there is no separation between naïve and relapsed samples. This is also reflected in the PCA using the first two principal components. The normal samples are coloured in blue, naïve cancer samples are coloured in green and the relapsed/refractory cancer samples are in red.
