# Supplementary material for: Comparative Gene Expression Profiling Identifies Common Molecular Signatures of NF-κB Activation in Canine and Human Diffuse Large B Cell Lymphoma (DLBCL)
Source: PLoS One. 2013 Sep 4;8(9):e72591. doi: 10.1371/journal.pone.0072591 (PMC3762807; doi:10.1371/journal.pone.0072591)
Supplement: File S4 — Supplemental data 4: GSEA Report for Dataset Canine_GSE30881_RMA_cg_2. (PDF) [file pone.0072591.s004.pdf]

GSEA Report for Dataset Canine_GSE30881_RMA_cg_2

Gene set: MSigDB c2.cp.v3.1

Enrichment in phenotype: Cancer (23 samples)

- 394 / 997 gene sets are upregulated in phenotype **Cancer**
- 74 gene sets are significant at FDR < 25%
- 15 gene sets are significantly enriched at nominal pvalue < 1%
- 62 gene sets are significantly enriched at nominal pvalue < 5%
- Snapshot of enrichment results
- Detailed enrichment results in html format
- Detailed enrichment results in excel format (tab delimited text)
- Guide to interpret results

Enrichment in phenotype: Healthy (10 samples)

- 603 / 997 gene sets are upregulated in phenotype **Healthy**
- 0 gene sets are significantly enriched at FDR < 25%
- 5 gene sets are significantly enriched at nominal pvalue < 1%
- 49 gene sets are significantly enriched at nominal pvalue < 5%
- Snapshot of enrichment results
- Detailed enrichment results in html format
- Detailed enrichment results in excel format (tab delimited text)
- Guide to interpret results

Dataset details

- The dataset has 43035 native features
- After collapsing features into gene symbols, there are: 16794 genes

Gene set details

- Gene set size filters (min=15, max=500) resulted in filtering out 455 / 1452 gene sets
- The remaining 997 gene sets were used in the analysis
- List of gene sets used and their sizes (restricted to features in the specified dataset)

Gene markers for the Cancer *versus* Healthy comparison

- The dataset has 16794 features (genes)
- # of markers for phenotype **Cancer**: 10383 (61.8%) with correlation area 50.0%
- # of markers for phenotype **Healthy**: 6411 (38.2%) with correlation area 50.0%
- Detailed rank ordered gene list for all features in the dataset
- Heat map and gene list correlation profile for all features in the dataset
- Buttefly plot of significant genes

Global statistics and plots

- Plot of p-values *vs.* NES
- Global ES histogram

Other

- Parameters used for this analysis

---

xtools.gsea.Gsea [Sun, May 12, '13 6 PM 30] Report: Canine_c2.cp.v3.1_p10000.Gsea.1368379813729.rpt by user: mani
Website: www.broadinstitute.org/GSEA Questions & Suggestions: Email

producer_class xtools.gsea.Gsea
producer_timestamp 1368379813729
param collapse true
param cls /GSEA/phenodata_canine.cls#Cancer_versus_Healthy param plot_top_x 20
param norm meandiv
param save_rnd_lists false
param median false
param num 100
param scoring_scheme weighted
param make_sets true
param mode Max_probe
param gmx gseaftp.broadinstitute.org://pub/gsea/gene_sets/c2.cp.v3.1.symbols.gmt param gui false
param chip /GSEA/Canine_2.na32.annot.chip
param metric Signal2Noise
param rpt_label Canine_c2.cp.v3.1_p10000
param help false
param order descending
param out /GSEA
param permute phenotype
param rnd_type no_balance
param set_min 15
param include_only_symbols true
param sort real
param rnd_seed timestamp
param nperm 10000
param zip_report false
param set_max 500
param res /GSEA/Canine_GSE30881_RMA_cg_2.txt
file /GSEA/Canine_c2.cp.v3.1_p10000.Gsea.1368379813729/index.html

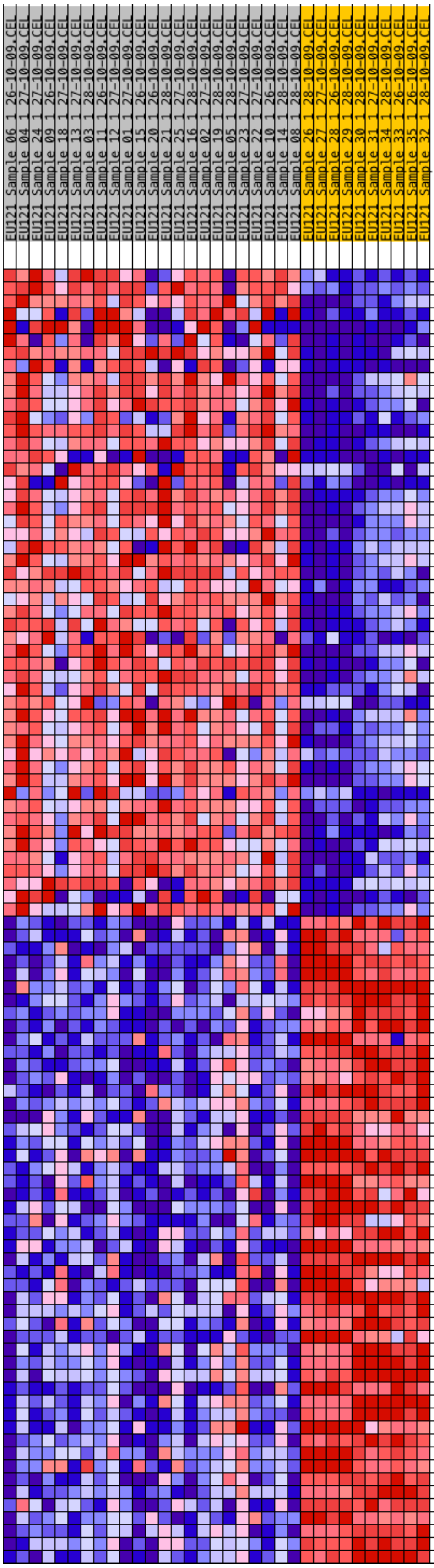

SampleName		
LOC478793		
ACCN5	ACCN5	amiloride-sensitive cation channel 5, intestinal
DNA2		
GSTA4 /// LOC606977		
LOC490493		
LONRF2	LONRF2	LON peptidase N-terminal domain and ring finger 2
SGOL1	SGOL1	shugoshin-like 1 (S. pombe)
SMPX	SMPX	small muscle protein, X-linked
LOC612464		
TTK	TTK	TTK protein kinase
CDCA5	CDCA5	cell division cycle associated 5
ELOVL4	ELOVL4	elongation of very long chain fatty acids (FEN1/Elo2, SUR4/Elo3, yeast)-like 4
ADORA2B	ADORA2B	adenosine A2b receptor
EXO1	EXO1	exonuclease 1
LRP1B	LRP1B	low density lipoprotein-related protein 1B (deleted in tumors)
SLC38A11		
LOC487888		
PLK4	PLK4	polo-like kinase 4 (Drosophila)
LOC477773		
NEK2	NEK2	NIMA (never in mitosis gene a)-related kinase 2
LOC607795 /// LOC609		
SLC6A12	SLC6A12	solute carrier family 6 (neurotransmitter transporter, betaine/GABA), member 12
LOC480027		
BCL2L14	BCL2L14	BCL2-like 14 (apoptosis facilitator)
FBXO43	FBXO43	F-box protein 43
LOC476098		
CDC45		
PRKAA2	PRKAA2	protein kinase, AMP-activated, alpha 2 catalytic subunit
SLC4A4	SLC4A4	solute carrier family 4, sodium bicarbonate cotransporter, member 4
BUB1B	BUB1B	BUB1 budding uninhibited by benzimidazoles 1 homolog beta (yeast)
CDCA7	CDCA7	cell division cycle associated 7
CDC6	CDC6	CDC6 cell division cycle 6 homolog (S. cerevisiae)
GTSE1	GTSE1	G-2 and S-phase expressed 1
SFRP2	SFRP2	secreted frizzled-related protein 2
DEPDC1	DEPDC1	DEP domain containing 1
PMCH	PMCH	pro-melanin-concentrating hormone
CENPF	CENPF	centromere protein F, 350/400ka (mitosin)
MYO5C	MYO5C	myosin VC
E2F8	E2F8	E2F transcription factor 8
ASPM	ASPM	asp (abnormal spindle) homolog, microcephaly associated (Drosophila)
ARMC2	ARMC2	armadillo repeat containing 2
DTL	DTL	denticleless homolog (Drosophila)
CENPK	CENPK	centromere protein K
SERPINB5	SERPINB5	serpin peptidase inhibitor, clade B (ovalbumin), member 5
LOC609907		
LOC609269		
CLSPN	CLSPN	claspin homolog (Xenopus laevis)
LOC607509 /// PHYH		
GTSF1		
ESCO2	ESCO2	establishment of cohesion 1 homolog 2 (S. cerevisiae)
CCR8	CCR8	chemokine (C-C motif) receptor 8
LOC491454 /// LOC606		
LOC475605		
LOC606953 /// LOC612		
LOC486389		
LOC610447		
CCL17	CCL17	chemokine (C-C motif) ligand 17
IL2	IL2	interleukin 2
LOC490595		
LOC606941 /// LOC606		
LOC482753 /// LOC609		
LEF1	LEF1	lymphoid enhancer-binding factor 1
LRRN1	LRRN1	leucine rich repeat neuronal 1
ZNF678	ZNF678	zinc finger protein 678
ZAP70	ZAP70	zeta-chain (TCR) associated protein kinase 70kDa
LOC607020 /// LOC612		
KHDRBS2	KHDRBS2	KH domain containing, RNA binding, signal transduction associated 2
LOC486386 /// LOC491		
LOC607125		
IL23R	IL23R	interleukin 23 receptor
LOC486393		
FMO3	FMO3	flavin containing monooxygenase 3
DUSP27	DUSP27	dual specificity phosphatase 27 (putative)
LGI1	LGI1	leucine-rich, glioma inactivated 1
EFHC2	EFHC2	EF-hand domain (C-terminal) containing 2
TSPAN8	TSPAN8	tetraspanin 8
CD52	CD52	CD52 molecule
FMO2	FMO2	flavin containing monooxygenase 2 (non-functional)
LOC606810		
CTLA4	CTLA4	cytotoxic T-lymphocyte-associated protein 4
LOC480351		
STMN2	STMN2	stathmin-like 2
LOC609871		
LOC478556		
CD40LG	CD40LG	CD40 ligand (TNF superfamily, member 5, hyper-IgM syndrome)
LOC609053		
LOC483848		
CD28	CD28	CD28 molecule
LOC480885		
LOC486382		
LOC608959		
TMEM178	TMEM178	transmembrane protein 178
LOC491391 /// LOC491		
LOC608983		
PTGDR	PTGDR	prostaglandin D2 receptor (DP)
LOC608537		
KCNS3	KCNS3	potassium voltage-gated channel, delayed-rectifier, subfamily S, member 3
PRKCQ	PRKCQ	protein kinase C, theta
ABCB1	ABCB1	ATP-binding cassette, sub-family B (MDR/TAP), member 1
LOC609043		

GSEA Report for Dataset human_GSE12195_RMA_hg_u133_p2

Gene Set: MSigDB c2.cp.v3.1

Enrichment in phenotype: DLBCL (45 samples)

- 836 / 1091 gene sets are upregulated in phenotype **DLBCL**
- 0 gene sets are significant at FDR < 25%
- 34 gene sets are significantly enriched at nominal pvalue < 1%
- 171 gene sets are significantly enriched at nominal pvalue < 5%
- Snapshot of enrichment results
- Detailed enrichment results in html format
- Detailed enrichment results in excel format (tab delimited text)
- Guide to interpret results

Enrichment in phenotype: Healthy (10 samples)

- 255 / 1091 gene sets are upregulated in phenotype **Healthy**
- 0 gene sets are significantly enriched at FDR < 25%
- 0 gene sets are significantly enriched at nominal pvalue < 1%
- 3 gene sets are significantly enriched at nominal pvalue < 5%
- Snapshot of enrichment results
- Detailed enrichment results in html format
- Detailed enrichment results in excel format (tab delimited text)
- Guide to interpret results

Dataset details

- The dataset has 54675 native features
- After collapsing features into gene symbols, there are: 21053 genes

Gene set details

- Gene set size filters (min=15, max=500) resulted in filtering out 361 / 1452 gene sets
- The remaining 1091 gene sets were used in the analysis
- List of gene sets used and their sizes (restricted to features in the specified dataset)

Gene markers for the DLBCL *versus* Healthy comparison

- The dataset has 21053 features (genes)
- # of markers for phenotype **DLBCL**: 11842 (56.2%) with correlation area 65.2%
- # of markers for phenotype **Healthy**: 9211 (43.8%) with correlation area 34.8%
- Detailed rank ordered gene list for all features in the dataset
- Heat map and gene list correlation profile for all features in the dataset
- Buttefly plot of significant genes

Global statistics and plots

- Plot of p-values *vs.* NES
- Global ES histogram

Other

- Parameters used for this analysis

producer_class xtools.gsea.Gsea
producer_timestamp 1368382535783
param collapse true
param cls /GSEA/CEL12195QC/DLBCL_vs_Healthy.cls#DLBCL_versus_Healthy param plot_top_x 20
param norm meandiv
param save_rnd_lists false
param median false
param num 100
param scoring_scheme weighted
param make_sets true
param mode Max_probe
param gmx gseaftp.broadinstitute.org://pub/gsea/gene_sets/c2.cp.v3.1.symbols.gmt param gui false
param chip /GSEA/CEL12195QC/HG-U133_Plus_2.na33.annot.chip
param metric Signal2Noise
param rpt_label Human_c2.cp.v3.1_p10000
param help false
param order descending
param out /GSEA/CEL12195QC
param permute phenotype
param rnd_type no_balance
param set_min 15
param include_only_symbols true
param sort real
param rnd_seed timestamp
param nperm 10000
param zip_report false
param set_max 500
param res /GSEA/CEL12195QC/human_GSE12195_RMA_hg_u133_p2.txt
file /GSEA/CEL12195QC/Human_c2.cp.v3.1_p10000.Gsea.1368382535783/index.html

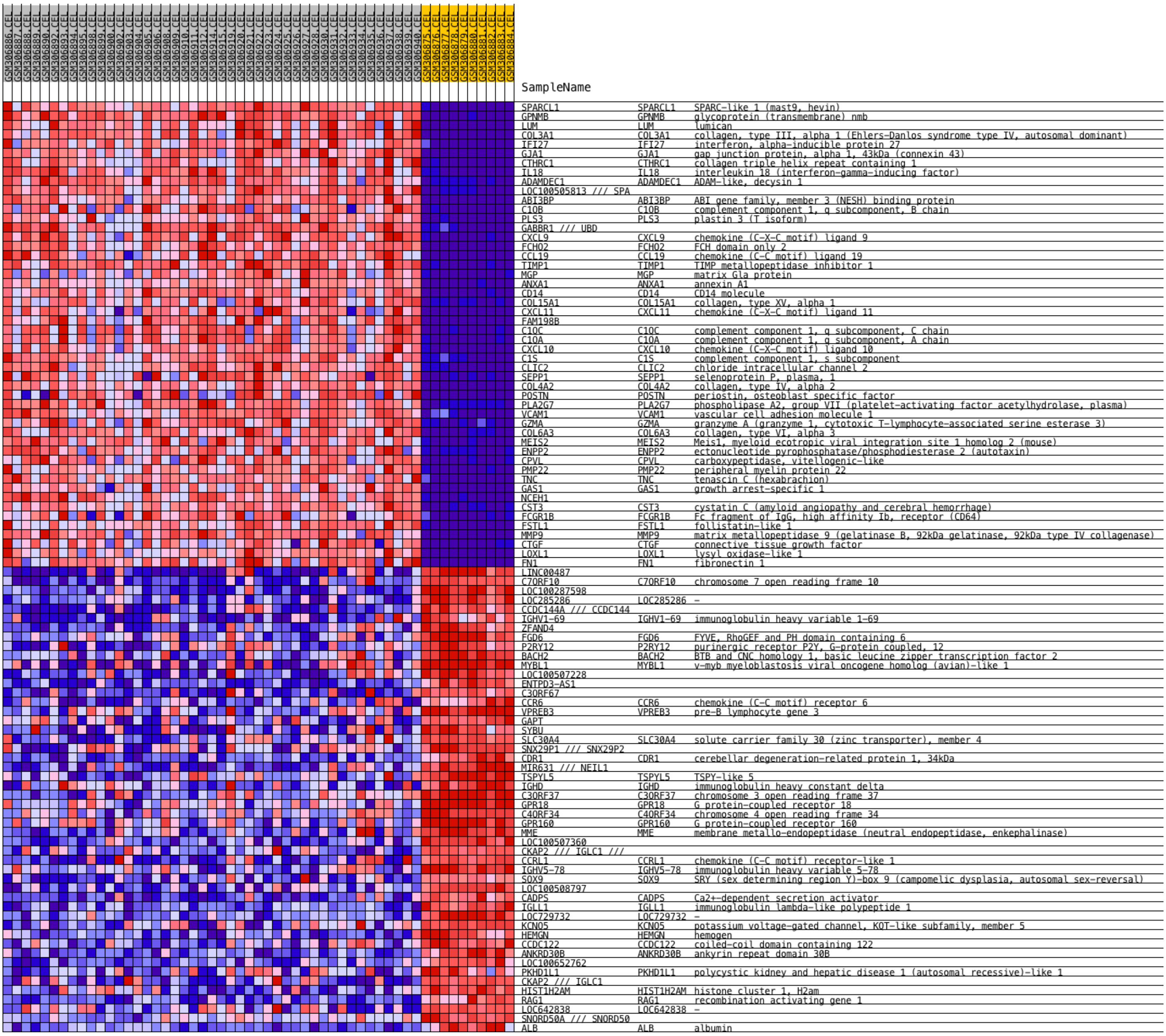
SampleName
SPARCL1 SPARCL1 SPARC−like 1 (mast9, hevin)
GPNMB GPNMB glycoprotein (transmembrane) nmb
LUM LUM lumican
COL3A1 COL3A1 collagen, type III, alpha 1 (Ehlers−Danlos syndrome type IV, autosomal dominant)
IFI27 IFI27 interferon, alpha−inducible protein 27
GJA1 GJA1 gap junction protein, alpha 1, 43kDa (connexin 43)
CTHRC1 CTHRC1 collagen triple helix repeat containing 1
IL18 IL18 interleukin 18 (interferon−gamma−inducing factor)
ADAMDEC1 ADAMDEC1 ADAM−like, decysin 1
LOC100505813 /// SPA
ABI3BP ABI3BP ABI gene family, member 3 (NESH) binding protein
C1QB C1QB complement component 1, q subcomponent, B chain
PLS3 PLS3 plastin 3 (T isoform)
GABBR1 /// UBD
CXCL9 CXCL9 chemokine (C−X−C motif) ligand 9
FCHO2 FCHO2 FCH domain only 2
CCL19 CCL19 chemokine (C−C motif) ligand 19
TIMP1 TIMP1 TIMP metallopeptidase inhibitor 1
MGP MGP matrix Gla protein
ANXA1 ANXA1 annexin A1
CD14 CD14 CD14 molecule
COL15A1 COL15A1 collagen, type XV, alpha 1
CXCL11 CXCL11 chemokine (C−X−C motif) ligand 11
FAM198B
C1QC C1QC complement component 1, q subcomponent, C chain
C1QA C1QA complement component 1, q subcomponent, A chain
CXCL10 CXCL10 chemokine (C−X−C motif) ligand 10
C1S C1S complement component 1, s subcomponent
CLIC2 CLIC2 chloride intracellular channel 2
SEPP1 SEPP1 selenoprotein P, plasma, 1
COL4A2 COL4A2 collagen, type IV, alpha 2
POSTN POSTN periostin, osteoblast specific factor
PLA2G7 PLA2G7 phospholipase A2, group VII (platelet−activating factor acetylhydrolase, plasma)
VCAM1 VCAM1 vascular cell adhesion molecule 1
GZMA GZMA granzyme A (granzyme 1, cytotoxic T−lymphocyte−associated serine esterase 3)
COL6A3 COL6A3 collagen, type VI, alpha 3
MEIS2 MEIS2 Meis1, myeloid ecotropic viral integration site 1 homolog 2 (mouse)
ENPP2 ENPP2 ectonucleotide pyrophosphatase/phosphodiesterase 2 (autotaxin)
CPVL CPVL carboxypeptidase, vitellogenic−like
PMP22 PMP22 peripheral myelin protein 22
TNC TNC tenascin C (hexabrachion)
GAS1 GAS1 growth arrest−specific 1
NCEH1
CST3 CST3 cystatin C (amyloid angiopathy and cerebral hemorrhage)
FCGR1B FCGR1B Fc fragment of IgG, high affinity Ib, receptor (CD64)
FSTL1 FSTL1 follistatin−like 1
MMP9 MMP9 matrix metallopeptidase 9 (gelatinase B, 92kDa gelatinase, 92kDa type IV collagenase)
CTGF CTGF connective tissue growth factor
LOXL1 LOXL1 lysyl oxidase−like 1
FN1 FN1 fibronectin 1
LINC00487
C7ORF10 C7ORF10 chromosome 7 open reading frame 10
LOC100287598
LOC285286 LOC285286 −
CCDC144A /// CCDC144
IGHV1−69 IGHV1−69 immunoglobulin heavy variable 1−69
ZFAND4
FGD6 FGD6 FYVE, RhoGEF and PH domain containing 6
P2RY12 P2RY12 purinergic receptor P2Y, G−protein coupled, 12
BACH2 BACH2 BTB and CNC homology 1, basic leucine zipper transcription factor 2
MYBL1 MYBL1 v−myb myeloblastosis viral oncogene homolog (avian)−like 1
LOC100507228
ENTPD3−AS1
C3ORF67
CCR6 CCR6 chemokine (C−C motif) receptor 6
VPREB3 VPREB3 pre−B lymphocyte gene 3
GAPT
SYBU
SLC30A4 SLC30A4 solute carrier family 30 (zinc transporter), member 4
SNX29P1 /// SNX29P2
CDR1 CDR1 cerebellar degeneration−related protein 1, 34kDa
MIR631 /// NEIL1
TSPYL5 TSPYL5 TSPY−like 5
IGHD IGHD immunoglobulin heavy constant delta
C3ORF37 C3ORF37 chromosome 3 open reading frame 37
GPR18 GPR18 G protein−coupled receptor 18
C4ORF34 C4ORF34 chromosome 4 open reading frame 34
GPR160 GPR160 G protein−coupled receptor 160
MME MME membrane metallo−endopeptidase (neutral endopeptidase, enkephalinase)
LOC100507360
CKAP2 /// IGLC1 ///
CCRL1 CCRL1 chemokine (C−C motif) receptor−like 1
IGHV5−78 IGHV5−78 immunoglobulin heavy variable 5−78
SOX9 SOX9 SRY (sex determining region Y)−box 9 (campomelic dysplasia, autosomal sex−reversal)
LOC100508797
CADPS CADPS Ca2+−dependent secretion activator
IGLL1 IGLL1 immunoglobulin lambda−like polypeptide 1
LOC729732 LOC729732 −
KCNQ5 KCNQ5 potassium voltage−gated channel, KQT−like subfamily, member 5
HEMGN HEMGN hemogen
CCDC122 CCDC122 coiled−coil domain containing 122
ANKRD30B ANKRD30B ankyrin repeat domain 30B
LOC100652762
PKHD1L1 PKHD1L1 polycystic kidney and hepatic disease 1 (autosomal recessive)−like 1
CKAP2 /// IGLC1
HIST1H2AM HIST1H2AM histone cluster 1, H2am
RAG1 RAG1 recombination activating gene 1
LOC642838 LOC642838 −
SNORD50A /// SNORD50
ALB ALB albumin

GSEA Report for Dataset Canine_GSE30881_RMA_cg_2

Gene Set: Top 100 gene sets enriched in the Human dataset GSE12195

Enrichment in phenotype: Cancer (23 samples)

- 19 / 90 gene sets are upregulated in phenotype **Cancer**
- 0 gene sets are significant at FDR < 25%
- 0 gene sets are significantly enriched at nominal pvalue < 1%
- 0 gene sets are significantly enriched at nominal pvalue < 5%
- Snapshot of enrichment results
- Detailed enrichment results in html format
- Detailed enrichment results in excel format (tab delimited text)
- Guide to interpret results

Enrichment in phenotype: Healthy (10 samples)

- 71 / 90 gene sets are upregulated in phenotype **Healthy**
- 0 gene sets are significantly enriched at FDR < 25%
- 3 gene sets are significantly enriched at nominal pvalue < 1%
- 10 gene sets are significantly enriched at nominal pvalue < 5%
- Snapshot of enrichment results
- Detailed enrichment results in html format
- Detailed enrichment results in excel format (tab delimited text)
- Guide to interpret results

Dataset details

- The dataset has 43035 native features
- After collapsing features into gene symbols, there are: 16794 genes

Gene set details

- Gene set size filters (min=15, max=500) resulted in filtering out 10 / 100 gene sets
- The remaining 90 gene sets were used in the analysis
- List of gene sets used and their sizes (restricted to features in the specified dataset)

Gene markers for the Cancer *versus* Healthy comparison

- The dataset has 16794 features (genes)
- # of markers for phenotype **Cancer**: 10383 (61.8%) with correlation area 50.0%
- # of markers for phenotype **Healthy**: 6411 (38.2%) with correlation area 50.0%
- Detailed rank ordered gene list for all features in the dataset
- Heat map and gene list correlation profile for all features in the dataset
- Buttefly plot of significant genes

Global statistics and plots

- Plot of p-values *vs.* NES
- Global ES histogram

Other

- Parameters used for this analysis

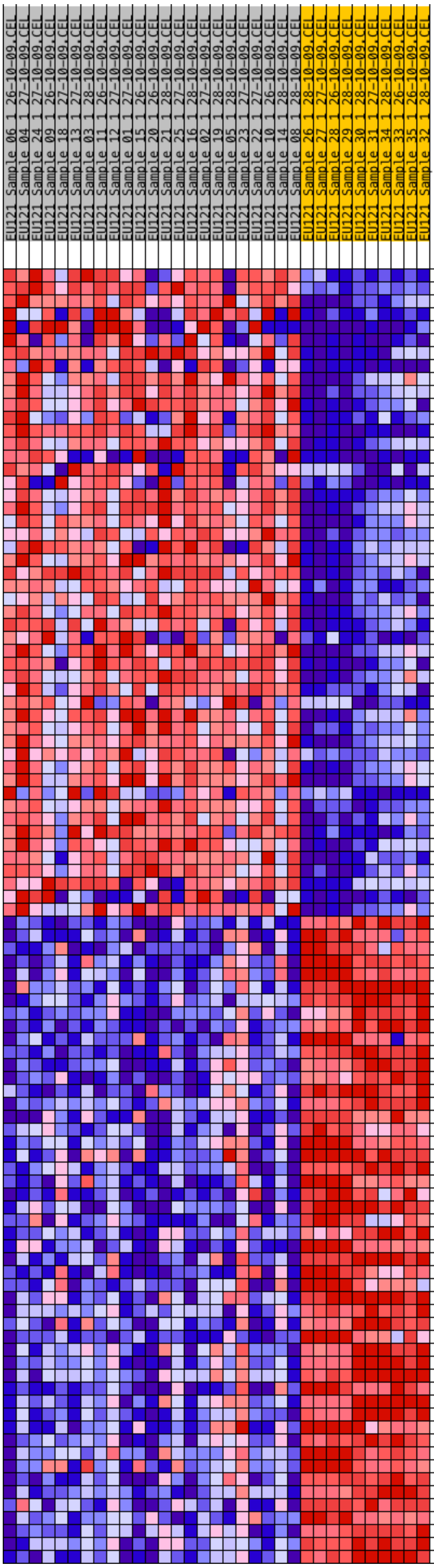

SampleName		
LOC478793		
ACCN5	ACCN5	amiloride-sensitive cation channel 5, intestinal
DNA2		
GSTA4 /// LOC606977		
LOC490493		
LONRF2	LONRF2	LON peptidase N-terminal domain and ring finger 2
SGOL1	SGOL1	shugoshin-like 1 (S. pombe)
SMPX	SMPX	small muscle protein, X-linked
LOC612464		
TTK	TTK	TTK protein kinase
CDCA5	CDCA5	cell division cycle associated 5
ELOVL4	ELOVL4	elongation of very long chain fatty acids (FEN1/Elo2, SUR4/Elo3, yeast)-like 4
ADORA2B	ADORA2B	adenosine A2b receptor
EXO1	EXO1	exonuclease 1
LRP1B	LRP1B	low density lipoprotein-related protein 1B (deleted in tumors)
SLC38A11		
LOC487888		
PLK4	PLK4	polo-like kinase 4 (Drosophila)
LOC477773		
NEK2	NEK2	NIMA (never in mitosis gene a)-related kinase 2
LOC607795 /// LOC609		
SLC6A12	SLC6A12	solute carrier family 6 (neurotransmitter transporter, betaine/GABA), member 12
LOC480027		
BCL2L14	BCL2L14	BCL2-like 14 (apoptosis facilitator)
FBXO43	FBXO43	F-box protein 43
LOC476098		
CDC45		
PRKAA2	PRKAA2	protein kinase, AMP-activated, alpha 2 catalytic subunit
SLC4A4	SLC4A4	solute carrier family 4, sodium bicarbonate cotransporter, member 4
BUB1B	BUB1B	BUB1 budding uninhibited by benzimidazoles 1 homolog beta (yeast)
CDCA7	CDCA7	cell division cycle associated 7
CDC6	CDC6	CDC6 cell division cycle 6 homolog (S. cerevisiae)
GTSE1	GTSE1	G-2 and S-phase expressed 1
SFRP2	SFRP2	secreted frizzled-related protein 2
DEPDC1	DEPDC1	DEP domain containing 1
PMCH	PMCH	pro-melanin-concentrating hormone
CENPF	CENPF	centromere protein F, 350/400ka (mitosin)
MYO5C	MYO5C	myosin VC
E2F8	E2F8	E2F transcription factor 8
ASPM	ASPM	asp (abnormal spindle) homolog, microcephaly associated (Drosophila)
ARMC2	ARMC2	armadillo repeat containing 2
DTL	DTL	denticleless homolog (Drosophila)
CENPK	CENPK	centromere protein K
SERPINB5	SERPINB5	serpin peptidase inhibitor, clade B (ovalbumin), member 5
LOC609907		
LOC609269		
CLSPN	CLSPN	claspin homolog (Xenopus laevis)
LOC607509 /// PHYH		
GTSF1		
ESCO2	ESCO2	establishment of cohesion 1 homolog 2 (S. cerevisiae)
CCR8	CCR8	chemokine (C-C motif) receptor 8
LOC491454 /// LOC606		
LOC475605		
LOC606953 /// LOC612		
LOC486389		
LOC610447		
CCL17	CCL17	chemokine (C-C motif) ligand 17
IL2	IL2	interleukin 2
LOC490595		
LOC606941 /// LOC606		
LOC482753 /// LOC609		
LEF1	LEF1	lymphoid enhancer-binding factor 1
LRRN1	LRRN1	leucine rich repeat neuronal 1
ZNF678	ZNF678	zinc finger protein 678
ZAP70	ZAP70	zeta-chain (TCR) associated protein kinase 70kDa
LOC607020 /// LOC612		
KHDRBS2	KHDRBS2	KH domain containing, RNA binding, signal transduction associated 2
LOC486386 /// LOC491		
LOC607125		
IL23R	IL23R	interleukin 23 receptor
LOC486393		
FMO3	FMO3	flavin containing monooxygenase 3
DUSP27	DUSP27	dual specificity phosphatase 27 (putative)
LGI1	LGI1	leucine-rich, glioma inactivated 1
EFHC2	EFHC2	EF-hand domain (C-terminal) containing 2
TSPAN8	TSPAN8	tetraspanin 8
CD52	CD52	CD52 molecule
FMO2	FMO2	flavin containing monooxygenase 2 (non-functional)
LOC606810		
CTLA4	CTLA4	cytotoxic T-lymphocyte-associated protein 4
LOC480351		
STMN2	STMN2	stathmin-like 2
LOC609871		
LOC478556		
CD40LG	CD40LG	CD40 ligand (TNF superfamily, member 5, hyper-IgM syndrome)
LOC609053		
LOC483848		
CD28	CD28	CD28 molecule
LOC480885		
LOC486382		
LOC608959		
TMEM178	TMEM178	transmembrane protein 178
LOC491391 /// LOC491		
LOC608983		
PTGDR	PTGDR	prostaglandin D2 receptor (DP)
LOC608537		
KCNS3	KCNS3	potassium voltage-gated channel, delayed-rectifier, subfamily S, member 3
PRKCQ	PRKCQ	protein kinase C, theta
ABCB1	ABCB1	ATP-binding cassette, sub-family B (MDR/TAP), member 1
LOC609043		

GSEA Report for Dataset human_GSE12195_RMA_hg_u133_p2

Gene Set: Top 100 gene sets enriched in the Canine dataset GSE30881

Enrichment in phenotype: DLBCL (45 samples)

- 32 / 98 gene sets are upregulated in phenotype **DLBCL**
- 0 gene sets are significant at FDR < 25%
- 0 gene sets are significantly enriched at nominal pvalue < 1%
- 1 gene sets are significantly enriched at nominal pvalue < 5%
- Snapshot of enrichment results
- Detailed enrichment results in html format
- Detailed enrichment results in excel format (tab delimited text)
- Guide to interpret results

Enrichment in phenotype: Healthy (10 samples)

- 66 / 98 gene sets are upregulated in phenotype **Healthy**
- 0 gene sets are significantly enriched at FDR < 25%
- 0 gene sets are significantly enriched at nominal pvalue < 1%
- 0 gene sets are significantly enriched at nominal pvalue < 5%
- Snapshot of enrichment results
- Detailed enrichment results in html format
- Detailed enrichment results in excel format (tab delimited text)
- Guide to interpret results

Dataset details

- The dataset has 54675 native features
- After collapsing features into gene symbols, there are: 21053 genes

Gene set details

- Gene set size filters (min=15, max=500) resulted in filtering out 2 / 100 gene sets
- The remaining 98 gene sets were used in the analysis
- List of gene sets used and their sizes (restricted to features in the specified dataset)

Gene markers for the DLBCL *versus* Healthy comparison

- The dataset has 21053 features (genes)
- # of markers for phenotype **DLBCL**: 11842 (56.2%) with correlation area 65.2%
- # of markers for phenotype **Healthy**: 9211 (43.8%) with correlation area 34.8%
- Detailed rank ordered gene list for all features in the dataset
- Heat map and gene list correlation profile for all features in the dataset
- Buttefly plot of significant genes

Global statistics and plots

- Plot of p-values *vs.* NES
- Global ES histogram

Other

- Parameters used for this analysis

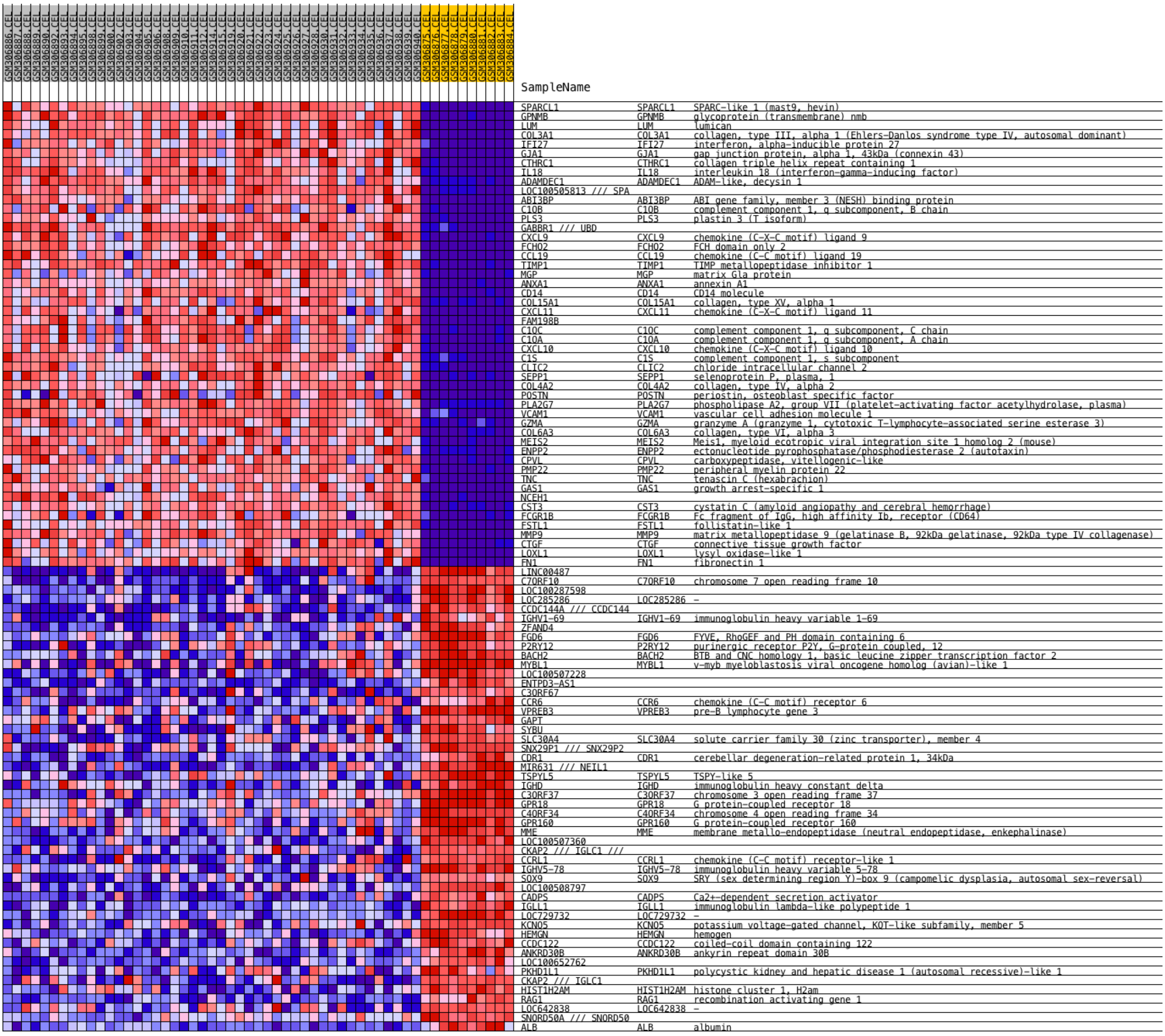

SampleName

SPARCL1	SPARCL1	SPARC-like 1 (mast9, hevin)
GPNMB	GPNMB	glycoprotein (transmembrane) nmb
LUM	LUM	lumican
COL3A1	COL3A1	collagen, type III, alpha 1 (Ehlers-Danlos syndrome type IV, autosomal dominant)
IFI27	IFI27	interferon, alpha-inducible protein 27
GJA1	GJA1	gap junction protein, alpha 1, 43kDa (connexin 43)
CTHRC1	CTHRC1	collagen triple helix repeat containing 1
IL18	IL18	interleukin 18 (interferon-gamma-inducing factor)
ADAMDEC1	ADAMDEC1	ADAM-like, decysin 1
LOC100505813 /// SPA		
ABI3BP	ABI3BP	ABI gene family, member 3 (NESH) binding protein
C1QB	C1QB	complement component 1, q subcomponent, B chain
PLS3	PLS3	plastin 3 (T isoform)
GABBR1 /// UBD		
CXCL9	CXCL9	chemokine (C-X-C motif) ligand 9
FCHO2	FCHO2	FCH domain only 2
CCL19	CCL19	chemokine (C-C motif) ligand 19
TIMP1	TIMP1	TIMP metallopeptidase inhibitor 1
MGP	MGP	matrix Gla protein
ANXA1	ANXA1	annexin A1
CD14	CD14	CD14 molecule
COL15A1	COL15A1	collagen, type XV, alpha 1
CXCL11	CXCL11	chemokine (C-X-C motif) ligand 11
FAM198B		
C1QC	C1QC	complement component 1, q subcomponent, C chain
C1QA	C1QA	complement component 1, q subcomponent, A chain
CXCL10	CXCL10	chemokine (C-X-C motif) ligand 10
C1S	C1S	complement component 1, s subcomponent
CLIC2	CLIC2	chloride intracellular channel 2
SEPP1	SEPP1	selenoprotein P, plasma, 1
COL4A2	COL4A2	collagen, type IV, alpha 2
POSTN	POSTN	periostin, osteoblast specific factor
PLA2G7	PLA2G7	phospholipase A2, group VII (platelet-activating factor acetylhydrolase, plasma)
VCAM1	VCAM1	vascular cell adhesion molecule 1
GZMA	GZMA	granzyme A (granzyme 1, cytotoxic T-lymphocyte-associated serine esterase 3)
COL6A3	COL6A3	collagen, type VI, alpha 3
MEIS2	MEIS2	Meis1, myeloid ecotropic viral integration site 1 homolog 2 (mouse)
ENPP2	ENPP2	ectonucleotide pyrophosphatase/phosphodiesterase 2 (autotaxin)
CPVL	CPVL	carboxypeptidase, vitellogenic-like
PMP22	PMP22	peripheral myelin protein 22
TNC	TNC	tenascin C (hexabrachion)
GAS1	GAS1	growth arrest-specific 1
NCEH1		
CST3	CST3	cystatin C (amyloid angiopathy and cerebral hemorrhage)
FCGR1B	FCGR1B	Fc fragment of IgG, high affinity Ib, receptor (CD64)
FSTL1	FSTL1	follistatin-like 1
MMP9	MMP9	matrix metallopeptidase 9 (gelatinase B, 92kDa gelatinase, 92kDa type IV collagenase)
CTGF	CTGF	connective tissue growth factor
LOXL1	LOXL1	lysyl oxidase-like 1
FN1	FN1	fibronectin 1
LINC00487		
C7ORF10	C7ORF10	chromosome 7 open reading frame 10
LOC100287598		
LOC285286	LOC285286	-
CCDC144A /// CCDC144		
IGHV1-69	IGHV1-69	immunoglobulin heavy variable 1-69
ZFAND4		
FGD6	FGD6	FYVE, RhoGEF and PH domain containing 6
P2RY12	P2RY12	purinergic receptor P2Y, G-protein coupled, 12
BACH2	BACH2	BTB and CNC homology 1, basic leucine zipper transcription factor 2
MYBL1	MYBL1	v-myb myeloblastosis viral oncogene homolog (avian)-like 1
LOC100507228		
ENTPD3-AS1		
C3ORF67		
CCR6	CCR6	chemokine (C-C motif) receptor 6
VPREB3	VPREB3	pre-B lymphocyte gene 3
GAPT		
SYBU		
SLC30A4	SLC30A4	solute carrier family 30 (zinc transporter), member 4
SNX29P1 /// SNX29P2		
CDR1	CDR1	cerebellar degeneration-related protein 1, 34kDa
MIR631 /// NEIL1		
TSPYL5	TSPYL5	TSPY-like 5
IGHD	IGHD	immunoglobulin heavy constant delta
C3ORF37	C3ORF37	chromosome 3 open reading frame 37
GPR18	GPR18	G protein-coupled receptor 18
C4ORF34	C4ORF34	chromosome 4 open reading frame 34
GPR160	GPR160	G protein-coupled receptor 160
MME	MME	membrane metallo-endopeptidase (neutral endopeptidase, enkephalinase)
LOC100507360		
CKAP2 /// IGLC1 ///		
CCRL1	CCRL1	chemokine (C-C motif) receptor-like 1
IGHV5-78	IGHV5-78	immunoglobulin heavy variable 5-78
SOX9	SOX9	SRY (sex determining region Y)-box 9 (campomelic dysplasia, autosomal sex-reversal)
LOC100508797		
CADPS	CADPS	Ca2+-dependent secretion activator
IGLL1	IGLL1	immunoglobulin lambda-like polypeptide 1
LOC729732	LOC729732	-
KCNQ5	KCNQ5	potassium voltage-gated channel, KQT-like subfamily, member 5
HEMGN	HEMGN	hemogen
CCDC122	CCDC122	coiled-coil domain containing 122
ANKRD30B	ANKRD30B	ankyrin repeat domain 30B
LOC100652762		
PKHD1L1	PKHD1L1	polycystic kidney and hepatic disease 1 (autosomal recessive)-like 1
CKAP2 /// IGLC1		
HIST1H2AM	HIST1H2AM	histone cluster 1, H2am
RAG1	RAG1	recombination activating gene 1
LOC642838	LOC642838	-
SNORD50A /// SNORD50		
ALB	ALB	albumin
